# Asymmetric Synthesis of 2,3‐Dihydrobenzofurans by a [4+1] Annulation Between Ammonium Ylides and In Situ Generated *o*‐Quinone Methides

**DOI:** 10.1002/chem.201700171

**Published:** 2017-03-27

**Authors:** Nicole Meisinger, Lukas Roiser, Uwe Monkowius, Markus Himmelsbach, Raphaël Robiette, Mario Waser

**Affiliations:** ^1^ Johannes Kepler University LinzInstitute of Organic ChemistryAltenbergerstrasse 694040LinzAustria; ^2^ Johannes Kepler University LinzInstitute of Inorganic ChemistryAltenbergerstrasse 694040LinzAustria; ^3^ Johannes Kepler University LinzInstitute of Analytical ChemistryAltenbergerstrasse 694040LinzAustria; ^4^ Université catholique de LouvainInstitute of Condensed Matter and NanosciencesPlace Louis Pasteur 1 box L4.01.021348Louvain-la-NeuveBelgium

**Keywords:** annulation, asymmetric synthesis, chiral auxiliaries, density functional calculations, quinone methides, ylides

## Abstract

A highly enantio‐ and diastereoselective [4+1] annulation between in situ generated ammonium ylides and *o*‐quinone methides for the synthesis of a variety of 2,3‐dihydrobenzofurans has been developed. The key factors controlling the reactivity and stereoselectivity were systematically investigated by experimental and computational means and the energy profiles obtained provide a deeper insight into the mechanistic details of this reaction.

## Introduction

The 2,3‐dihydrobenzofuran skeleton represents an important motif in a variety of natural products and biologically active molecules.^1^ Accordingly, the development of new methods for the synthesis of these important target molecules has recently attracted considerable interest and several complementary strategies have been introduced to access them in either a racemic or even in a stereoselective fashion.^2^, ^3^, ^4^, ^5^, ^6^

A highly valuable approach to accessing chiral carbo‐ or heterocycles is the use of onium ylides for [*n*+1] annulation reactions.^7^ These versatile reagents have found widespread applications in [2+1] annulations to access (chiral) epoxides, aziridines and cyclopropanes.^7^, ^8^, ^9^, ^10^ More recently, these compounds were also successfully employed in [4+1] annulations^11^ by treating them with different vinylogous acceptor molecules.^4^, ^5^, ^6^, ^12^, ^13^

**Scheme 1:**
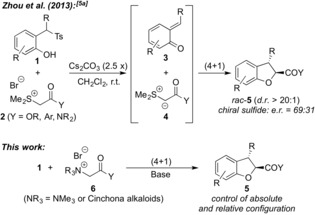
Zhou and co‐workers’ recently developed sulfur ylide (**4**) addition to *o*‐quinone methides **3** and targeted use of (chiral) ammonium salts **6** to control the absolute and relative configurations of 2,3‐dihydrobenzofurans **5**.

One particularly powerful methodology for the generation of carbo‐ and heterocyclic targets through [4+*n*] annulation approaches relies on the use of in situ generated *o*‐quinone methides or analogous aza‐*o*‐quinone methides.^14^, ^15^ Recently, the first example of the use of in situ generated *o*‐quinone methides **3** (precursor **1**) for [4+1] annulation reactions with in situ generated carbonyl‐stabilised sulfur ylides **4** (obtained from sulfonium salts **2**) to access 2,3‐dihydrobenzofurans **5** was reported by Zhou and co‐workers (Scheme 1).5a High yields and excellent diastereoselectivities were obtained for different carbonyl‐stabilised sulfur ylides. However, the use of a known camphor‐derived chiral sulfonium ylide resulted in moderate enantiocontrol only (e.r.=69:31). In addition, Yang and Xiao very recently reported the moderately enantioselective reaction of achiral sulfur ylides with in situ generated *o*‐quinone methides in the presence of chiral urea catalysts.5d

Following our recent research focused on ammonium ylide mediated asymmetric annulation reactions^10^ and [4+1] annulations using ylides,12d we became interested in testing the potential of in situ generated achiral and chiral ammonium ylides (by starting from ammonium salts **6**) to access 2,3‐dihydrobenzofurans with control of both the relative and the absolute configuration. Besides the development of the first (highly) asymmetric ammonium ylide based protocol, which will overcome the less than satisfactory enantioselectivity observed so far when using chiral sulfur ylides, we also became interested in investigating this reaction in more detail by computational means to reveal the key factors in this ylide‐mediated [4+1] annulation reaction.^16^

## Results and Discussion

### Development of an enantioselective reaction protocol

The first attempts were made with the achiral trimethylammonium salts **6 a**–**c** to investigate the influence of the nature of the carbonyl group on the reaction with *o*‐quinone methide precursor **1 a** (Table 1). Trimethylamine was chosen as the amine group because of its superior leaving group ability as compared with other achiral tertiary amines.^16^ In an initial screening of a variety of different solvent/base combinations we found that the use of 2.5 equivalents of Cs_2_CO_3_ in CH_2_Cl_2_ at room temperature gave the most satisfactory results when using the achiral trimethylamine‐containing ammonium salts **6 a**–**c**. Under these conditions, the ester‐containing ammonium salt **6 a** gave the corresponding dihydrobenzofuran **5 a** with high *trans* selectivity, but in only moderate yield (39 %, no significant improvement could be achieved by varying the conditions or stoichiometry). Interestingly, the less‐stabilised amide‐based ammonium ylide derived from **6 b** did not give any product at all (just the formation of unidentified side‐products), whereas the acetophenone‐based salt **6 c** could be used to access **5 c** in an excellent yield of 95 % and with almost complete *trans* diastereoselectivity under the optimised reaction conditions (Table 1, entries 1–3).

**Table 1:** Screening of different chiral and achiral ammonium salts **6** for the synthesis of 2,3‐dihydrobenzofurans **5**.^[a]^

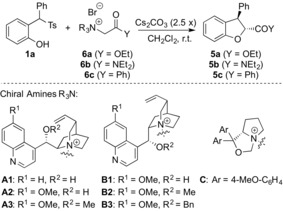						
Entry	**6**	Amine	*t* [h]	Yield [%]^[b]^	*trans*/*cis* ^[c]^	e.r. (*trans*)^[d]^
1	**6 a**	Me_3_N	20	39	>95:5	–
2	**6 b**	Me_3_N	20	n.r.^[e]^	–	–
3	**6 c**	Me_3_N	20	95	>95:5	–
4	**6 c**	**A1**	20	37^[f]^	>95:5	94:6
5	**6 c**	**A2**	20	65	>95:5	99:1
6	**6 c**	**A3**	20	53	>95:5	97:3
7	**6 c**	**B1**	20	62	>95:5	8:92
8	**6 c**	**B2**	20	52	>95:5	16:84
9	**6 c**	**B3**	20	43	>95:5	34:66
10	**6 c**	**C**	20	63	90:10	30:70
11	**6 c**	**A2**	72	85	>95:5	99:1

**Scheme 2:**
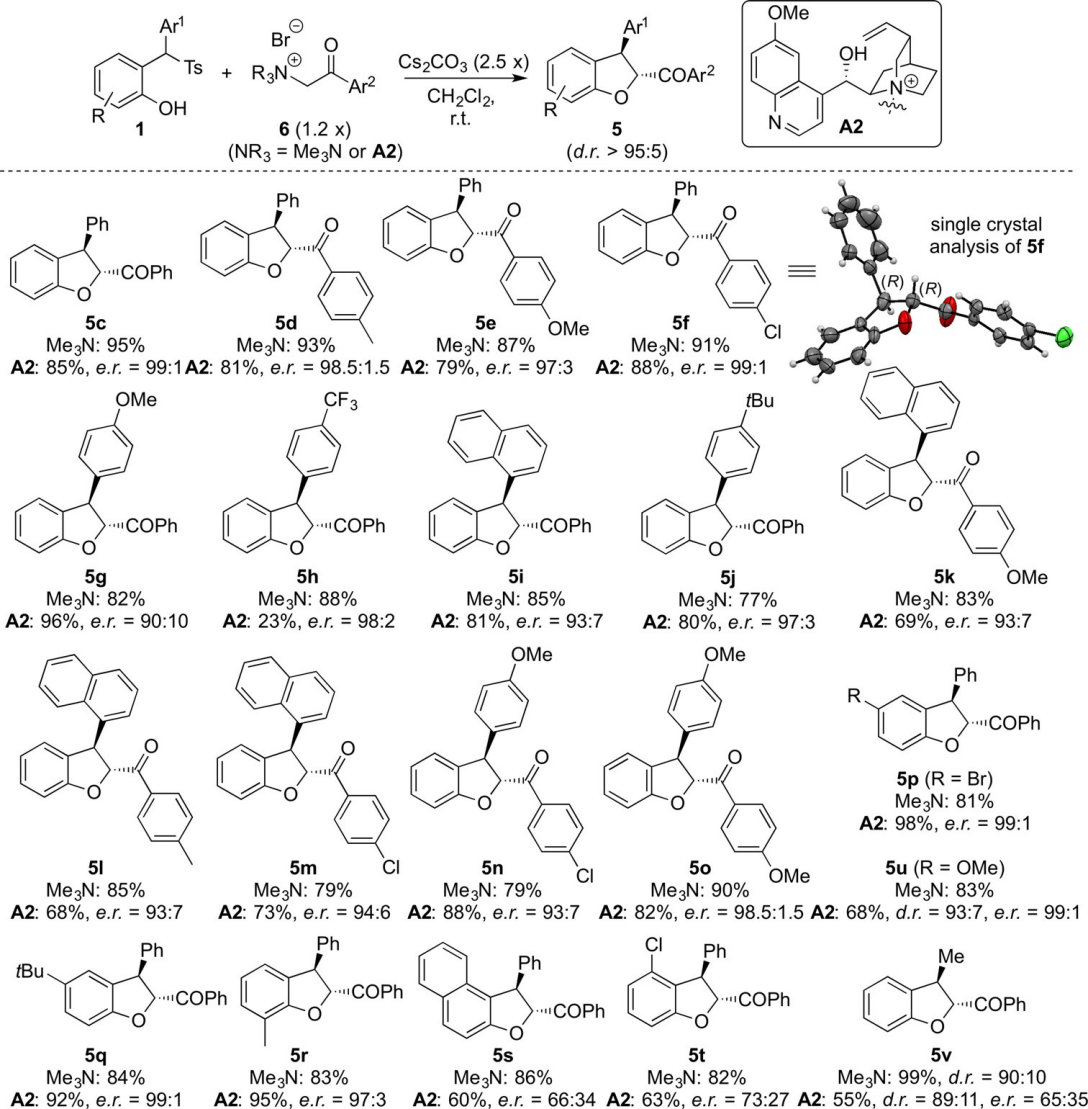
Scope of the asymmetric synthesis of 2,3‐dihydrobenzofurans **5**. All reactions were performed on a scale of 0.1–1 mmol. Racemic reactions were run for 1 day and the chiral‐amine‐based reactions for 3 days to ensure full conversion. X‐ray analysis of the chlorine‐containing product **5 f** proved a 2*R*,3*R* configuration for this derivative and the other products were assigned by analogy.^17^

[a] Typical conditions: **1 a** (0.1 mmol), **6** (0.12 mmol), Cs_2_CO_3_ (0.25 mmol), CH_2_Cl_2_, RT. [b] Isolated yield. [c] Determined by ^1^H NMR analysis of the crude product. [d] Determined by HPLC analysis using a chiral stationary phase and given as the ratio (2*R*,3*R*)/(2*S*,3*S*). X‐ray analysis of the chlorine‐containing product **5 f** (see Scheme 2) confirmed a 2*R*,3*R* configuration for this derivative and **5 c** was assigned in analogy. [e] Full consumption and formation of unidentified side‐products of **1** and **6 b**. [f] Incomplete conversion of **6**.

Having identified conditions that allowed for the high‐yielding and highly diastereoselective synthesis of racemic dihydrobenzofuran **5 c**, we next focused on the identification of a suitable chiral amine leaving group to develop an enantioselective protocol for this reaction. Cinchona alkaloids are usually the chiral tertiary amines of choice for chiral ammonium enolate based reactions and have proven their potential in ammonium ylide mediated cyclopropanations9a, 9b and [4+1] annulations to α,β‐unsaturated imines in the past.13b However, these amines were found not to be suited to ammonium ylide mediated asymmetric epoxidation reactions and it was only recently that we succeeded in developing a proline‐based chiral amine (structure **C**, Table 1) to achieve that goal.10f We were thus very pleased to see that literally the first attempt with amine **A1** gave **5 c** in high enantiopurity (Table 1, entry 4). As shown in entry 5, the yield and enantiopurity could be improved by using quinidine (**A2**) as the chiral amine leaving group (e.r.=99:1, d.r.>95:5, 65 % isolated yield) and we also soon realised that Cinchona alkaloids with a free 9‐OH group (**A2** and **B1**) allowed for higher enantioselectivities than the analogous *O*‐alkyl derivatives (see entries 4–9). Both enantiomers of *trans*‐**5 c** could readily be obtained by using either **A2** or the pseudo‐enantiomeric **B1**, but it should be pointed out that **A2** allowed for slightly higher enantiomeric ratios than **B1**. Based on our positive recent experience with compounds **C**,10f we also tested this class of auxiliaries herein (entry 10), but it was clearly shown that these amines are less suited to this [4+1] annulation than the easily available Cinchona alkaloids. Interestingly, as the conversion of the **A2**‐containing ammonium salt **6 c** was still not complete after 20 h reaction time (which is in sharp contrast to the reaction with Me_3_N), longer reaction times of up to 3 days were necessary to achieve comparable yields for the enantioselective protocol (compare entries 11 and 3 in Table 1).

Unfortunately, attempts to start from α‐bromoacetophenone and carry out in situ ammonium salt formation and ylide generation were not successful using either a catalytic or a stoichiometric amount of quinidine. As we mainly observed the decomposition of **1 a** in these attempts, it seems likely that the formation of the ammonium salt **6 c** is slow in comparison with the generation of *o*‐quinone methide **3** (which, in the absence of a suitable nucleophilic reaction partner, undergoes the aforementioned decomposition reaction). Overall, this obstacle makes a catalytic protocol unfeasible. However, it should be pointed out that the amine that is liberated during the reaction can easily be recovered after workup.

Having identified the optimum conditions and the best‐suited chiral amine leaving group for the first highly asymmetric and high‐yielding formal [4+1] annulation of onium ylides to *o*‐quinone methides, we next explored the scope of this strategy. As can be seen in Scheme 2, a variety of differently substituted acceptors **1** and ammonium salts **6** were very well tolerated either in the racemic reaction (using Me_3_N‐containing ammonium salts) or in the asymmetric protocol. In all cases the *trans* products were obtained with almost exclusive diastereoselectivity and satisfactory isolated yields. The major exception in this regard was the asymmetric synthesis of the CF_3_‐containing product **5 h**, which was achieved in a low yield of only 23 %, whereas the racemic protocol gave a yield of 88 %. We first reasoned that this could be due to a decomposition reaction between the product and the free Cinchona base that is liberated during the reaction. However, test reactions proved that the product **5 h** is stable in the presence of **A2** and therefore the exact reason for this striking difference remains unclear.

With respect to the influence of different aryl substituents on the enantioselectivity, two cases caught our attention. Although only subtle decreases in enantioselectivity were observed in most cases when we introduced substituents either on the acceptor or on the donor site, the presence of residues at the 3‐position of the quinone methide resulted in a significantly lower enantioselectivity (see products **5 s** and **t**). It has been reported that the introduction of sterically demanding groups at this position leads to the preferred formation of the (*Z*)‐quinone methides, whereas unsubstituted derivatives mainly form *E* isomers.^14^ We thus rationalised that this lower selectivity may be attributed to formation of a less selective (*Z*)‐quinone methide (or an *E*/*Z* mixture) in these two cases. We also tested one substrate **1** with a methyl group instead of the Ar^1^ substituent, which performed reasonably well in the racemic approach by giving **5 v** in a yield of 99 % but with a slightly lower d.r. However, the enantioselectivity achieved in the asymmetric protocol turned out to be lower. We also carried out a gram‐scale experiment for the asymmetric synthesis of **5 i**, which was thereby obtained with the same enantiopurity as in the experiment performed on the 0.1 mmol scale (see Scheme 2), but with a yield of 67 % (compared with the 81 % achieved from the reaction on a smaller scale).

### Computational studies

**Figure 1:**
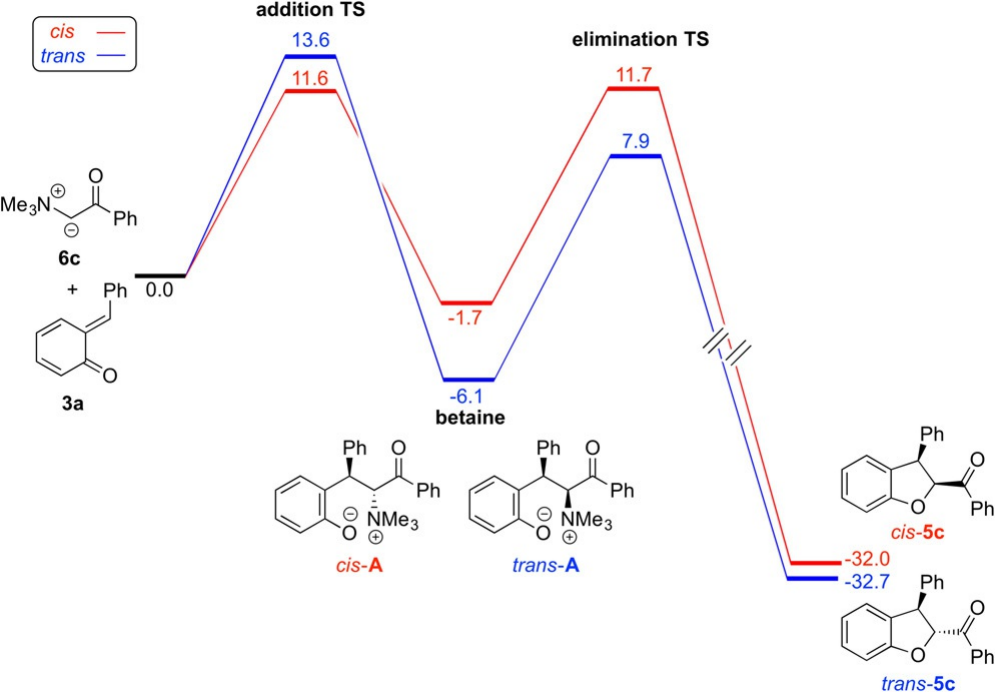
Computed free‐energy profiles [kcal mol^−1^] for the formation of *cis*‐ and *trans*‐2,3‐dihydrobenzofurans **5 c** from ammonium ylide **6 c**.

To identify the mechanism and the key factors controlling the reactivity and stereoselectivity in this [4+1] annulation, we investigated the free‐energy profile of the parent reaction between trimethylamine‐containing ylide **6 c** (R=Me_3_N; Ar^2^=Ph) and the *o*‐quinone methide generated form **1 a** (Ar^1^=Ph; Figure 1). Calculations were carried out at the B3LYP‐D3/6‐311+G**//B3LYP‐D3/6‐31G* level of theory^18^ including a continuum description of dichloromethane as solvent.^19^

The mechanistic sequence involves two key steps along each of the diastereoisomeric pathways leading to *cis*‐ and *trans*‐2,3‐dihydrobenzofurans **5**. The first is the addition of the ammonium ylide at the methylene position of the electron‐deficient *o*‐quinone methide to form a betaine intermediate (see Figure 1).^20^ This betaine can then undergo ring closure, with concomitant expulsion of the amine, to give the corresponding 2,3‐dihydrobenzofuran.^21^

Despite the sterically hindered character of the formed betaine, the initial addition step is slightly exothermic (by 2–6 kcal mol^−1^) due to the re‐aromatisation of the phenolic fragment. Two diastereomeric betaines can be formed during the addition step, *trans*‐ and *cis*‐**A** (Figure 1). *trans*‐**A** is computed to be more stable than *cis*‐**A** (−6.1 and −1.7 kcal mol^−1^, respectively), but the latter is predicted to be formed through a lower free‐energy barrier (11.6 kcal mol^−1^) than *trans*‐**A** (13.6 kcal mol^−1^).

Because the cyclisation occurs by an S_N_2‐type mechanism, this step is stereospecific such that betaines *trans*‐**A** and *cis*‐**A** lead to the *trans*‐ and *cis*‐2,3‐dihydrobenzofurans **5**, respectively. The free‐energy barrier to ring closure is found to be similar for both diastereomeric betaines (13–14 kcal mol^−1^). In the case of *trans*‐**A**, the transition state (TS) of the ring closure lies lower in free energy than the addition TS, which indicates a non‐reversible initial addition step. For the *cis* pathway, the lower stability of *cis*‐**A** results in a partially reversible betaine formation. Indeed, the *cis* addition and *cis* elimination TSs are very similar in terms of free energy (11.6 and 11.7 kcal mol^−1^, respectively). These transition states lie, however, lower than the *trans* addition TS, and thus a *cis* selectivity is predicted, which is in disagreement with the observed high *trans* selectivity (*trans*/*cis* >95:5).

We reasoned that this contrast between computational predictions and experimental findings could be explained by a faster *cis*‐2,3‐dihydrobenzofuran **5 c** formation (kinetic selectivity) followed by an epimerisation of this latter. To test this hypothesis, we isolated *cis*‐**5 c** and subjected it to the reaction conditions. A slow isomerisation to the more stable *trans*‐**5 c** isomer was observed by NMR analysis and complete epimerisation was observed after 15 h (d.r.>95:5). Monitoring of the [4+1] annulation reaction over time revealed, however, that the >95:5 diastereomeric ratio in favour of the *trans*‐2,3‐dihydrobenzofuran is observed throughout the reaction, even in the first few minutes. Thus, given its slow kinetics, epimerisation of *cis*‐2,3‐dihydrobenzofuran cannot account by itself for the observed high *trans* selectivity.

**Figure 2:**
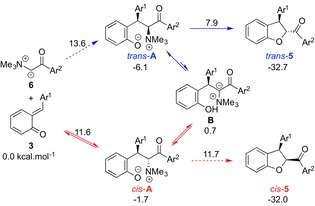
Mechanism and rationale for the observed high *trans* selectivity in [4+1] annulation reactions between ketone‐stabilised ammonium ylides and *o*‐quinone methides (relative free energies are given in kcal mol^−1^).

Another potential explanation involves the epimerisation of the betaine intermediate **A** by a deprotonation/protonation mechanism, such as previously observed by Aggarwal and co‐workers in sulfur ylide mediated cyclopropanation reactions.^22^ Indeed, the computed relative free energy of **B** (0.7 kcal mol^−1^; Figure 2) indicates a facile proton transfer, and hence epimerisation, in *cis*‐**A** (see the Supporting Information for full details). The observed high *trans* selectivity can thus be accounted for by selective *cis*‐**A** formation followed by rapid epimerisation into *trans*‐**A**, which then undergoes ring closure to give the *trans*‐2,3‐dihydrobenzofuran (Figure 2). This also rationalises why the two less enantioselective examples **5 s** and **5 t** were still obtained with very high *trans* selectivities, even though quinone methide formation proceeds only with low diastereoselectivity.

Interestingly, the computed free‐energy profile (see Figure 1) also allows a rationalisation of the low (or no) yield in **5** with ester‐ and amide‐based ammonium ylides derived from **6 a** and **6 b**, respectively (see entries 1and 2 in Table 1). As a result of a lower stabilisation of the ylide, addition should be more favoured, and more exothermic, in these cases in comparison with ketone‐based ylides. However, for the elimination step, the reverse is the case as the free‐energy barrier is expected to increase with the ester and amide derivatives.^23^ In addition, the isomerisation of betaine by deprotonation/protonation should also be less favoured and the poor yields in these cases can thus probably be explained by a longer lifetime of betaine intermediates that favours side‐reactions.

## Conclusion

We have succeeded in the development of the first highly asymmetric [4+1] annulation protocol between in situ generated ammonium ylides and *o*‐quinone methides to access chiral 2,3‐dihydrobenzofuran derivatives. Key to success was the use of an easily available Cinchona alkaloid as the chiral leaving group, which has resulted in an operationally simple and highly enantio‐ and diastereoselective synthetic strategy. Detailed computational studies support a mechanistic scenario in which the high *trans* selectivity of this procedure originates from a rapid isomerisation of the *cis*‐betaine intermediate to the *trans*‐betaine intermediate, thus resulting in high diastereoselectivities for a broad application scope.

## Experimental Section

General experimental details can be found in the Supporting Information.

**General asymmetric [4+1] annulation procedure**: Compound **1** (1 equiv), ammonium salt **6** (1.2 equiv) and Cs_2_CO_3_ (2.5 equiv) were dissolved in DCM (15 mL mmol^−1^ **1**). The reaction mixture was stirred at room temperature for 3 days and afterwards extracted with DCM and brine. The combined organic phases were dried over Na_2_SO_4_, filtered and evaporated to dryness. The crude products were purified by column chromatography (silica gel, gradient of heptane/EtOAc) gave the corresponding 2,3‐dihydrobenzofurans in the reported yields and enantiopurities.

**Product 5 c**: Obtained as a white residue in a yield of 85 % on a 1 mmol scale (e.r.=99:1). [*α*]20D =−8.8 (*c*=0.2 in DCM, e.r.=99:1); ^1^H NMR (700 MHz, CDCl_3_, 298 K): *δ*=4.99 (d, *J=*6.5 Hz, 1 H), 5.82 (d, *J=*6.4 Hz, 1 H), 6.90 (t, *J=*7.5 Hz, 1 H), 7.01–6.98 (m, 2 H), 7.24–7.20 (m, 3 H), 7.30–7.28 (m, 1 H), 7.35–7.33 (m, 2 H), 7.46 (t, *J=*7.7 Hz, 2 H), 7.60 (t, *J=*7.3 Hz, 1 H), 7.96 ppm (d, *J=*7.6 Hz, 2 H); ^13^C NMR (176 MHz, CDCl_3_, 298 K): *δ*=51.0, 90.7, 110.1, 121.8, 125.5, 127.6, 128.3, 128.8, 129.0, 129.1, 129.5, 133.9, 134.6, 142.4, 159.2, 194.8 ppm; HRMS (ESI): *m*/*z* calcd for C_21_H_16_O_2_: 323.1043 [*M*+Na]^+^; found: 323.1040. The enantioselectivity was determined by HPLC (YMC Cellulose‐SB, eluent: hexane/*i*PrOH=95:5, 0.5 mL min^−1^, 10 °C; retention times: *t*_major_ (2*R*,3*R*)=15.7, *t*_minor_ (2*S*,3*S*)=17.0 min).

## Conflict of interest

The authors declare no conflict of interest.

## Supporting information

As a service to our authors and readers, this journal provides supporting information supplied by the authors. Such materials are peer reviewed and may be re‐organized for online delivery, but are not copy‐edited or typeset. Technical support issues arising from supporting information (other than missing files) should be addressed to the authors.

Supplementary
