# Supplementary material for: Asymmetric Synthesis of 2,3‐Dihydrobenzofurans by a [4+1] Annulation Between Ammonium Ylides and In Situ Generated o‐Quinone Methides
Source: Chemistry. 2017 Mar 27;23(21):5137–42. doi: 10.1002/chem.201700171 (PMC5419452; doi:10.1002/chem.201700171)
Supplement: Supplementary file 1 — Supplementary [file CHEM-23-5137-s001.pdf]

CHEMISTRY
A European Journal

Supporting Information

Asymmetric Synthesis of 2,3-Dihydrobenzofurans by a [4+1] Annulation Between Ammonium Ylides and In Situ Generated *o*-Quinone Methides

Nicole Meisinger+,[a] Lukas Roiser+,[a] Uwe Monkowius,[b] Markus Himmelsbach,[c] Raphaël Robiette,*[d] and Mario Waser*[a]

chem_201700171_sm_miscellaneous_information.pdf

SUPPORTING INFORMATION

Asymmetric synthesis of 2,3-dihydrobenzofurans via a (4+1) annulation between ammonium ylides and in situ generated ortho-quinone methides

Nicole Meisinger,[a] Lukas Roiser,[a] Uwe Monkowius,[b] Markus Himmelsbach,[c] Raphaël Robiette,[d,*] and Mario Waser[a,*]

a) *Institute of Organic Chemistry, Johannes Kepler University Linz, Altenbergerstraße 69, 4040 Linz, Austria. Fax: +43 732 2468 8747; Tel: +43 732 2468 8748; E-mail: Mario.waser@jku.at*

b) *Institute of Inorganic Chemistry, Johannes Kepler University Linz, Altenbergerstraße 69, 4040 Linz, Austria.*

c) *Institute of Analytical Chemistry, Johannes Kepler University Linz, Altenbergerstraße 69, 4040 Linz, Austria.*

d) *Institute of Condensed Matter and Nanosciences, Université catholique de Louvain, Place Louis Pasteur 1 box L4.01.02, 1348 Louvain-la-Neuve, Belgium. E-mail: raphael.robiette@uclouvain.be*

1. General Information: 2
1.1. General Methods 2
1.2. Computational Methods 2
2. Syntheses 4
2.1 Syntheses of Starting Materials 4
2.2 (Asymmetric) (4+1) Cyclization Reaction 10
3. Computational Details, Energies and Geometries 16
3.1 Conformational study of betaines 16
3.2. Addition on the aromatic ring 17
3.3. Benchmark calculations 17
3.4. Epimerization process 18
3.5. Computational results – Energies and Geometries 19
4. Copies of HPLC Chromatograms 43
5. Copies of NMR-Spectra of new Compounds 83

1. General Information:

1.1. General Methods

^{1}H- and ^{13}C-NMR spectra were recorded on a Bruker Avance III 300 MHz spectrometer with a broad band observe probe and a sample changer for 16 samples and on a Bruker Avance III 700 MHz spectrometer with with an Ascend magnet and TCI cryoprobe, which are both property to the Austro-Czech NMR-Research Center “RERI-uasb”. All NMR spectra were referenced on the solvent peak. High resolution mass spectra were obtained using an Agilent 6520 Q-TOF mass spectrometer with an ESI source and an Agilent G1607A coaxial sprayer or a Thermo Fisher Scientific LTQ Orbitrap XL with an Ion Max API Source. Analyses were made in the positive ionization mode if not otherwise stated. Purine (exact mass for $[M+H]^+$ = 121.050873) and 1,2,3,4,5,6-hexakis(2,2,3,3-tetrafluoropropoxy)-1,3,5,2,4,6-triazatriphosphinane (exact mass for $[M+H]^+$ = 922.009798) were used for internal mass calibration.

IR spectra were recorded on a Bruker Tensor 27 FT-IR spectrometer with ATR unit.

Preparative column chromatography was carried out using Davisil LC 60A 70-200 MICRON silica gel. TLC probes were detected at 254 nm or stained with with an appropriate staining solution (compare section 3.1.3).

HPLC was performed using a Dionex Summit HPLC system consisting of a Dionex P-680 pump, an ASI-100 HPLC-autosampler, a STH-585 column oven and a PDA-100 detector or a Thermo Scientific Dionex Ultimate 3000 system with diode array detector with a Chiralcel OD-H (250 x 4.6 mm, 5 µm) or a YMC Cellulose-SB (250 x 4.6 mm, 5 µm)) chiral stationary phase.

Optical rotations were recorded on a Schmidt + Haensch Polarimeter Model UniPol L 1000 (1 dm cuvette).

Single-crystal structure analyses were carried out on a Bruker Smart X2S diffractometer operating with Mo-K_α radiation (λ= 0.71073 Å).

All chemicals were purchased from commercial suppliers and used without further purification unless otherwise stated. All reactions were carried out under Argon.

1.2. Computational Methods

Geometry optimization has been performed using the Jaguar 8.5 pseudospectral program package using the well-established B3LYP hybrid density functional with the D3 dispersion correction and the standard split valence polarized 6-31G* basis as implemented in Jaguar. All the optimization calculations include an implicit description of dichloromethane solvent using the Poisson–Boltzmann polarizable continuum method as incorporated in Jaguar, and parameters for dichloromethane.

Electronic energies were obtained by single point calculations at the B3LYP-D3/6-311+G**(dichloromethane) level of theory.

The correct nature of each stationary point as minima (zero imaginary frequencies) or transition states (one imaginary frequency) has been checked by performing frequency calculations at the B3LYP/6-31G*(dichloromethane) level of theory.

Thermal and entropic contributions to free energy (at 298.15 K) and zero-point energy have been obtained from these frequency calculations. In Jaguar, the translational partition function is computed for ideal gas standard conditions, corresponding to a pressure of 1 atmosphere at 298.15 K. For solution reactions, the standard condition is instead 1 mol/L. Accordingly, the free energy value computed in Jaguar was corrected by a concentration term, equal to RT ln (V_mol_gas_1atm / V_mol_1M), i.e. 1.89 kcal/mol at 298.15 K.

For the large reaction systems there are usually several local minima or saddle points corresponding to each intermediate or transition state. This is due to the possibility of multiple conformations of substituents. We have made a systematic attempt to locate all possible local minima and saddle points, with the data presented referring to the lowest energy form unless mentioned otherwise. All species have been fully geometry optimized, and the Cartesian coordinates are supplied in Section 3.

2. Syntheses

2.1 Syntheses of Starting Materials

2.1.1 Synthesis of Quinone Methide Precursors 1

General procedure (in analogy to literature[1]):

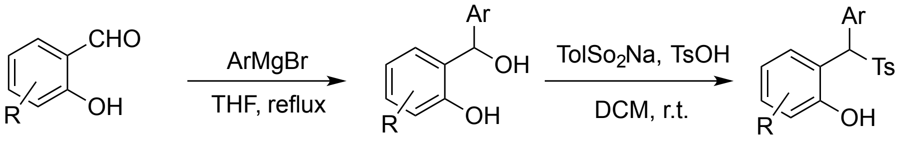

1) The arylbromide (ArBr, 2.5 equiv.) is dissolved in THF (0.7 mL per mmol ArBr) and added dropwise to a mixture of Mg turnings (2.5 equiv.) and THF (0.35 mL per mmol Mg). After formation of the Grignard reagent has started, the mixture is stirred at room temperature for 30 min and then refluxed for 1 h. After cooling with an ice bath, the aldehyde (1 equiv.), which is solved in THF (1.1 mL per mmol aldehyde), is added dropwise and the solution is stirred at room temperature for 3 h. Afterwards the reaction is quenched with saturated NH_4Cl and the mixture extracted with DCM and washed with brine. The combined organic phases are dried over Na_2SO_4, filtered, and evaporated to dryness. The crude product is purified by column chromatography (silica gel, heptane:EtOAc).

2) $TolSO_2Na$ (1.15 equiv.) and TsOH (1.75 equiv.) are dissolved in DCM (3.4 mL per mmol $TolSO_2Na$) and stirred for 5 min. Then the afore synthesised alcohol (part 1) is dissolved in DCM (2.5 mL per mmol) and added to the mixture and then stirred at room temperature for 1.5 h. Afterwards the reaction mixture is extracted with DCM and washed with 1N HCl and brine. The combined organic layers are dried over Na_2SO_4 and evaporated to dryness. The crude product is separated by column chromatography (silica gel, heptane:EtOAc) to give starting materials **1** in the reported yields.

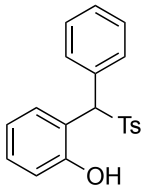

1c

Compound **1c**: Obtained as a light orange powder in 55% over 2 steps (4.5 mmol scale), R_f: 0.43 (heptane:EtOAc = 3:2). Analytical data match those reported in literature[1]. ^{1}H NMR (300 MHz, δ, $CDCl_3$, 298 K): 2.36 (s, 3H), 5.83 (s, 1H), 6.55 (s, 1H), 6.95 – 6.87 (m, 2H), 7.23 – 7.12 (m, 3H), 7.33 – 7.29 (m, 3H), 7.57 – 7.52 (m, 5H) ppm.

1) Mu-Wang, C.; Liang-Liang, C.; Zhi-Shi, Y.; Guo-Fang, J.; Yong-Gui Z,; *Chem. Commun.*, **2013**, *49*, 1660

<u>Compound **1g**</u>: Obtained as a light yellow powder in 58% over 2 steps (4.5 mmol scale), R_f: 0.28 (heptane:EtOAc = 2:1). Analytical data match those reported previously[1]. ^{1}H NMR (300 MHz, δ, $CDCl_3$, 298 K): 2.37 (s, 3H), 3.78 (s, 3H), 5.76 (s, 1H), 6.66 (s, 1H), 6.94 – 6.80 (m, 4H), 7.23 – 7.16 (m, 3H), 7.57 – 7.43 (m, 5H) ppm.

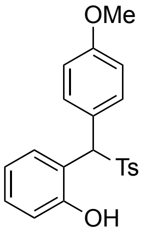

1g

<u>Compound **1h**</u>: Obtained as a light yellow powder in 43% over two steps (4.5 mmol scale), R_f: 0.48 (heptane:EtOAc = 2:1). Analytical data match those reported previously[1]. ^{1}H NMR (300 MHz, δ, $CDCl_3$, 298 K): 2.38 (s, 3H), 5.92 (s, 1H), 6.07 (s, 1H), 6.84 – 6.81 (m, 1H), 6.98 – 6.92 (m, 1H), 7.24 – 7.17 (m, 3H), 7.62 – 7.55 (m, 5H), 7.71 – 7.68 (m, 2H) ppm.

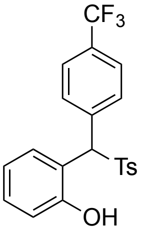

1h

<u>Compound **1i**</u>: Obtained as a dark purple powder in 80% over 2 steps (4.5 mmol scale), R_f: 0.44 (heptane:EtOAc = 2.5:2). ^{1}H NMR (300 MHz, δ, $CDCl_3$, 298 K): 2.3 (s, 3H), 6.92 – 6.82 (m, 3H), 7.20 – 7.10 (m, 3H), 7.40 – 7.37 (m, 2H), 7.57 – 7.52 (m, 2H), 7.61 (d, J = 8.3 Hz, 2H), 7.87 – 7.74 (m, 3H), 8.47 (d, J = 7.5 Hz, 1H) ppm. ^{13}C NMR (75 MHz, δ, $CDCl_3$, 298 K): 21.6, 63.3, 116.9, 120.1, 121.4, 122.5, 125.1, 125.7, 126.8, 127.2, 128.9, 129.1, 129.3, 129.5, 130.1, 131.1, 131.7, 133.9, 135.6, 144.6, 154.2 ppm. HRMS (ESI): *m/z* calculated for $C_{24}H_{20}O_3S$: 411.1025 $[M+Na]^+$; found: 411.1027.

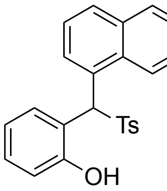

1i

<u>Compound **1j**</u>: Obtained as an orange powder in 53% over 2 steps (4.5 mmol scale), R_f: 0.45 (heptane:EtOAc = 2:1). ^{1}H NMR (300 MHz, δ, $CDCl_3$, 298 K): 1.28 (s, 9H), 2.36 (s, 3H), 5.78 (s, 1H), 6.59 (s, 2H), 6.93 – 6.86 (m, 2H), 7.22 – 7.13 (m, 3H), 7.34 – 7.31 (m, 2H), 7.55 – 7.45 (m, 5H) ppm. ^{13}C NMR (75 MHz, δ, $CDCl_3$, 298 K): 21.6, 31.2, 34.6, 70.6, 118.0, 120.3, 121.4, 125.6, 128.9, 129.0, 129.2, 130.0, 130.1, 131.1, 135.0, 144.6, 151.8, 154.3 ppm. HRMS (ESI): *m/z* calculated for $C_{24}H_{26}O_3S$: 417.1495 $[M+Na]^+$; found: 417.1495.

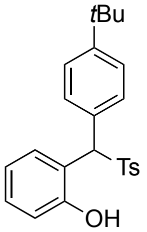

1j

<u>Compound **1p**</u>: Obtained as a white powder in 31% over 2 steps (4.5 mmol scale), R_f: 0.19 (heptane:EtOAc = 2.5:1). ^{1}H NMR (300 MHz, δ, $CDCl_3$, 298 K): 2.37 (s, 3H), 5.75 (s, 1H), 6.80 (d, J = 8.6 Hz, 1H), 7.18 (d, J = 8.0 Hz, 2H),7.34 – 7.28 (m, 4H), 7.50 – 7.47 (m, 2H), 7.56 (d, J = 8.3 Hz, 2H), 7.62 (d, J = 2.4 Hz, 1H) ppm. ^{13}C NMR (75 MHz, δ, $CDCl_3$, 298 K): 21.7, 70.2, 113.5, 119.8, 122.2, 128.8, 128.9, 129.5, 130.2, 131.7, 133.1, 133.4, 134.5, 145.1, 153.7 ppm. HRMS (ESI): *m/z* calculated for $C_{20}H_{17}BrO_3S$: 438.9979 $[M+Na]^+$; found: 438.9972.

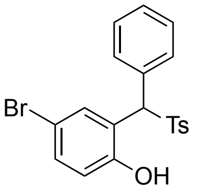

1p

Compound **1q**: Obtained as a white powder in 64% over 2 steps (4.5 mmol scale), R_f: 0.35 (heptane:EtOAc = 2:1). ^{1}H NMR (300 MHz, δ, $CDCl_3$, 298 K): 1.18 (s, 9H), 2.33 (s, 3H), 5.75 (s, 1H), 6.48 (s, 2H), 6.80 (d, J = 8.4 Hz, 1H), 7.20 – 7.11 (m, 3H), 7.32 – 7.28 (m, 3H), 7.37 – 7.36 (m, 1H), 7.58 – 7.51 (m, 4H) ppm. ^{13}C NMR (75 MHz, δ, $CDCl_3$, 298 K): 21.6, 31.3, 34.1, 71.7, 117.8, 119.2, 127.1, 128.2, 128.6, 128.7, 128.9, 129.3, 130.3, 132.0, 134.9, 144.1, 144.6, 152.1 ppm. HRMS (ESI): *m/z* calculated for $C_{24}H_{26}O_3S$: 417.1495 $[M+Na]^+$; found: 417.1495.

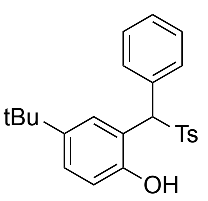

1q

Compound **1r**: Obtained as a light yellow powder in 63% over 2 steps (4.5 mmol scale), R_f: 0.50 (heptane:EtOAc = 3:2). ^{1}H NMR (300 MHz, δ, $CDCl_3$, 298 K): 2.27 (s, 3H), 2.36 (s, 3H), 5.85 (s, 1H), 6.83 (t, J = 7.7 Hz, 1H), 7.18 – 7.10 (m, 3H), 7.35 – 7.28 (m, 4H), 7.57 – 7.50 (m, 4H) ppm. ^{13}C NMR (75 MHz, δ, $CDCl_3$, 298 K): 16.4, 21.6, 30.9, 71.1, 119.9, 121.1, 126.7, 128.6, 128.7, 128.9, 129.3, 130.3, 131.5, 132.4, 134.9, 144.7, 152.8 ppm. HRMS (ESI): *m/z* calculated for $C_{21}H_{20}O_3S$: 375.1025 $[M+Na]^+$; found: 375.1023.

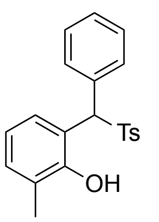

1r

Compound **1s**: Obtained as a yellow powder in 76% over 2 steps (4.5 mmol scale), R_f: 0.29 (heptane:EtOAc = 2.5:1). ^{1}H NMR (300 MHz, δ, $CDCl_3$, 298 K): 2.24 (s, 3H), 6.55 (s, 1H), 7.05 (d, J = 8.1 Hz, 2H), 7.28 – 7.20 (m, 2H), 7.39 – 7.32 (m, 4H), 7.74 – 7.63 (m, 7H), 8.89 (s, 1H) ppm. ^{13}C NMR (75 MHz, δ, $CDCl_3$, 298 K): 21.5, 30.9, 70.0, 110.8, 121.0, 123.8, 127.2, 128.6, 128.8, 128.9, 129.3, 129.4, 129.8, 130.9, 131.7, 133.0, 134.2, 145.2, 154.6 ppm. HRMS (ESI): *m/z* calculated for $C_{24}H_{20}O_3S$: 411.1025 $[M+Na]^+$; found: 411.1025.

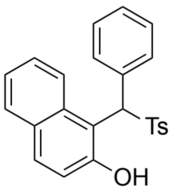

1s

Compound **1t**: Obtained as a white powder in 50% over 2 steps (3.2 mmol scale), R_f: 0.39 (heptane:EtOAc = 2.5:1). ^{1}H NMR (300 MHz, δ, $CDCl_3$, 298 K): 2.40 (s, 3H), 6.45 (s, 1H), 6.81 (dd, J = 1.4 Hz, J = 7.9 Hz, 1H), 6.93 (dd, J = 1.2 Hz, J = 8.4 Hz, 1H), 7.10 (t, J = 8.0 Hz, 1H), 7.22 (d, J = 8.1 Hz, 2H), 7.36 (t, J = 3.2 Hz, 3H), 7.67 – 7.59 (m, 4H), 8.73 (s, 1H) ppm. ^{13}C NMR (75 MHz, δ, $CDCl_3$, 298 K): 21.7, 71.7, 118.4, 119.0, 122.0, 128.7, 128.8, 129.0, 129.6, 129.8, 130.0, 130.9, 133.9, 135.8, 145.6, 157.1 ppm. HRMS (ESI): *m/z* calculated for $C_{20}H_{17}ClO_3S$: 395.0479 $[M+Na]^+$; found: 395.0481.

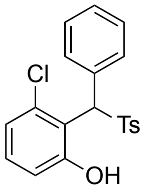

1t

2.1.2 Syntheses of Ammonium Salts 6

General procedure for achiral ammonium salts (in analogy to literature[2]):

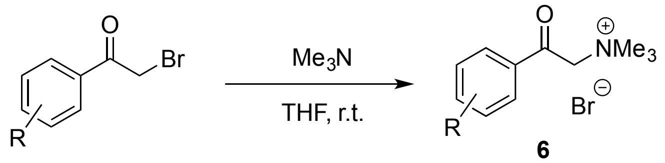

To a solution of bromoacetophenone derivative (1 equiv.) in THF (3.3 mL per mmol starting material) trimethylamine (33% in EtOH – 1 equiv.) is added and the mixture is stirred overnight. The product is filtered off and washed twice with EtOAc and dried under vacuo.

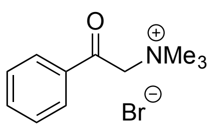

6c

Achiral ammonium salt **6c**: Isolated as a white powder in 88% (15 mmol scale), ^{1}H NMR (700 MHz, δ, $CDCl_3$, 298 K): 3.70 (s, 9H), 5.95 (s, 2H), 7.51 (t, *J* = 7.8 Hz, 2H), 7.64 (t, *J* = 7.4 Hz, 1H), 8.12 (d, *J* = 7.5 Hz, 2H) ppm. ^{13}C NMR (176 MHz, δ, $CDCl_3$, 298 K): 55.0, 68.4, 128.7, 129.5, 134.3, 135.3, 191.3 ppm. HRMS (ESI): *m/z* calculated for $C_{11}H_{16}NO$: 178.1226 $[M]^+$; found: 178.1225.

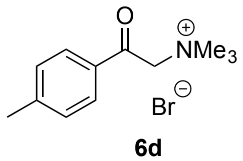

6d

Achiral ammonium salt **6d**: Obtained as a white powder in 97% (0.5 mmol scale), ^{1}H NMR (300 MHz, δ, DMSO, 298 K): 2.52 (s, 3H), 3.40 (s, 9H), 5.35 (s, 2H), 7.54 (d, *J* = 7.9 Hz, 2H), 7.99 (d, *J* = 7.9 Hz, 2H) ppm. ^{13}C NMR (75 MHz, δ, DMSO, 298 K): 21.3, 53.4, 67.0, 128.0, 129.6, 131.8, 145.5, 190.8 ppm. HRMS (ESI): *m/z* calculated for $C_{12}H_{18}NO$: 192.1383 $[M]^+$; found: 192.1381.

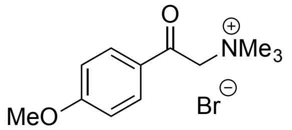

6e

Achiral ammonium salt **6e**: Isolated as a white powder in 69% (3 mmol scale), ^{1}H NMR (300 MHz, δ, $CDCl_3$, 298 K): 3.68 (s, 9H), 3.84 (s, 3H), 5.82 (s, 2H), 6.93 (d, *J* = 8.9 Hz, 2H), 8.10 (d, *J* = 8.9 Hz, 2H) ppm. ^{13}C NMR (75 MHz, δ, DMSO, 298 K): 53.4, 55.8, 66.8, 114.3, 127.2, 130.5, 164.3, 189.6 ppm. HRMS (ESI): *m/z* calculated for $C_{12}H_{18}NO_2$: 208.1332 $[M]^+$; found: 208.1333.

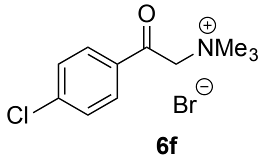

6f

Achiral ammonium salt **6f**: Obtained as a white powder in 99% (3 mmol scale), ^{1}H NMR (300 MHz, δ, DMSO, 298 K): 3.44 (s, 9H), 5.44 (s, 2H), 7.84 (d, *J* = 7.9 Hz, 2H), 8.13 (d, *J* = 8.2 Hz, 2H) ppm. ^{13}C NMR (75 MHz, δ, DMSO, 298 K): 53.5, 67.2, 129.2, 129.9, 133.1, 139.6, 190.4 ppm. HRMS (ESI): *m/z* calculated for $C_{11}H_{15}ClNO$: 212.0837 $[M]^+$; found: 212.0835.

2) Herchl, R.; Stiftinger, M.; Waser, M.; *Org. Biomol. Chem.*, **2011**, *9*, 7023.

General procedure for chiral ammonuim salts:

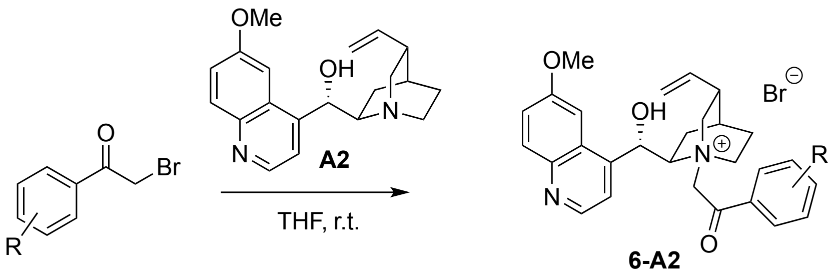

To a solution of bromoacetophenone derivative (1 equiv.) in THF (12 mL per mmol starting material) quinidine (**A2**) is added and the mixture is stirred overnight. After evaporation to dryness the crude product is purified by column chromatography (silica gel, DCM:MeOH).

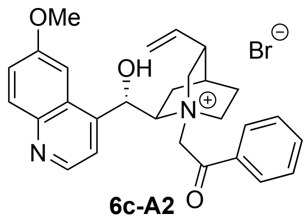

6c-A2

Chiral ammonium salt **6c-A2**: Isolated as a light yellow powder in 89 % (3 mmol scale). Analytical data match to literature[3]. ^{1}H NMR (300 MHz, δ, $CDCl_3$, 298 K): 1.01 – 0.92 (m, 1H), 2.05 – 1.94 (m, 3H), 2.30 (t, J = 12.3 Hz, 1H), 2.90 (q, J = 8.4 Hz, 1H), 4.10 (s, 3H), 4.49 – 4.33 (m, 2H), 4.79 – 4.73 (t, J = 9.5 Hz, 1H), 4.99 (t, J = 10.4 Hz, 1H), 5.36 – 5.27 (m, 2H), 5.68 – 5.61 (d, J = 18.9 Hz, 1H), 6.01 – 5.89 (m, 2H), 6.34 (d, J = 5.9 Hz, 1H), 6.725 (d, J = 18.8 Hz, 1H), 7.07 (d, J = 2.6 Hz, 1H), 7.38 – 7.34 (m, 1H), 7.49 (t, J = 7.5 Hz, 2H), 7.63 (t, J = 7.4 Hz, 1H), 7.80 (d, J = 4.5 Hz, 1H), 8.00 (d, J = 9.3 Hz, 1H), 8.35 (d, J = 8.0 Hz, 2H), 8.75 (d, J = 4.6 Hz, 1H) ppm.

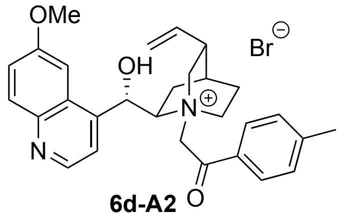

6d-A2

Chiral ammonium salt **6d-A2**: Obtained as a beige powder in 99 % (1 mmol scale), R_f: 0.40 (DCM:MeOH = 10:1). ^{1}H NMR (300 MHz, δ, $CDCl_3$, 298 K): 0.98 – 0.93 (m, 1H), 2.05 – 1.96 (m, 3H), 2.32 – 2.28 (m, 1H), 2.38 (s, 3H), 2.88 (q, J = 8.3 Hz, 1H), 3.66 (q, J = 10.7 Hz, 1H), 4.09 (s, 3H), 4.25 (t, J = 11.2 Hz, 1H), 4.53 (t, J = 10.4 Hz,1H), 4.76 (t, J = 9.6 Hz, 1H), 5.06 (t, J = 10.1 Hz, 1H), 5.46 – 5.28 (m, 3H), 6.02 – 5.91 (m, 2H), 6.37 (d, J = 5.8 Hz, 1H), 6.83 (d, J = 18.9 Hz, 1H), 7.05 (d, J = 2.6 Hz, 1H), 7.28 (d, J = 8.2 Hz, 2H), 7.36 (dd, J = 2.6 Hz, J = 7.3 Hz, 1H), 7.82 (d, J = 4.5 Hz, 1H), 8.00 (d, J = 9.2 Hz, 1H), 8.26 (d, J = 8.1 Hz, 2H), 8.76 (d, J = 4.6 Hz, 1H) ppm. ^{13}C NMR (75 MHz, δ, MeOD, 298 K): 21.8, 22.1, 24.5, 28.5, 39.3, 57.2, 58.6, 63.3, 64.7, 68.2, 101.8, 118.1, 121.4, 124.2,127.4, 129.8, 131.1, 131.7, 133.0, 137.0, 144.8, 145.5, 148.1, 148.5, 160.4, 194.1 ppm. HRMS (ESI): m/z calculated for $C_{29}H_{33}N_2O_3$: 457.2486 $[M]^+$; found: 457.2480.

3) Jian, L.; Yunbo, Q.; Tingting, L.;Yongmei, W.; *Bioorg. Med. Chem. Lett.*, **2007**, *17*, 4102.

Chiral ammonium salt **6e-A2**: Obtained as a light yellow powder in 99% (1 mmol scale). Analytical

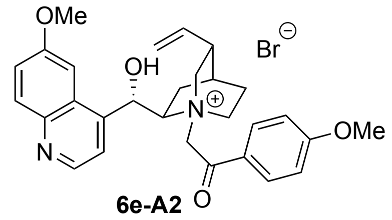

data match those reported before[3]. ^{1}H NMR (300 MHz, δ, $CDCl_3$, 298 K): 0.98 – 0.89 (m, 1H), 2.02 – 1.91 (m, 3H), 2.27 (t, *J* = 12.1 Hz, 1H), 2.90 (q, *J* = 8.4 Hz, 1H), 3.80 (s, 3H), 4.10 (s, 3H), 4.51 – 4.44 (m, 2H), 4.74 (t, *J* = 9.4 Hz, 1H), 4.95 (t, *J* = 10.4 Hz, 1H), 5.35 – 5.26 (m, 2H), 5.70 (d, *J* = 18.3 Hz, 1H), 6.00 – 5.88 (m, 2H), 6.34 (d, *J* = 5.9 Hz, 1H), 6.59 (d, *J* = 18.3 Hz, 1H), 6.90 (d, *J* = 9.0 Hz, 2H), 7.09 (d, *J* = 2.6 Hz, 1H), 7.35 (dd, *J* = 2.6 Hz, *J* = 9.3 Hz, 1H), 7.79 (d, *J* = 4.7 Hz, 1H), 7.98 (d, *J* = 9.2 Hz, 1H), 8.33 (d, *J* = 8.9 Hz, 2H), 8.72 (d, *J* = 4.6 Hz, 1H) ppm.

Chiral ammonium salt **6f-A2**: Isolated as a light pink powder in quantitative yield (1 mmol scale), R_f:

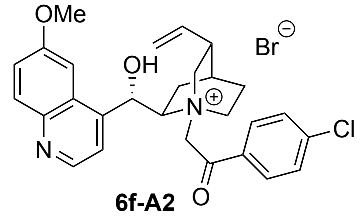

0.30 (DCM:MeOH = 10:1). Analytical data match those reported in literature[3]. ^{1}H NMR (300 MHz, δ, $CDCl_3$, 298 K): 0.99 – 0.91 (m, 2H), 2.03 – 1.93 (m, 3H), 2.28 (t, *J* = 11.7 Hz, 1H), 2.87 (q, *J* = 8.6 Hz, 1H), 3.47 (d, *J* = 5.1 Hz, 1H), 3.79 – 3.75 (m, 1H), 4.08 (s, 3H), 4.48 – 4.40 (m, 2H), 4.72 (t, *J* = 9.4 Hz, 1H), 4.95 (t, *J* = 9.8 Hz, 1H), 5.33 – 5.27 (m, 2H), 6.00 – 5.82 (m, 3H), 6.22 (d, *J* = 6.1 Hz, 1H), 6.70 (d, *J* = 18.9 Hz, 1H), 7.03 (d, *J* = 2.6 Hz, 1H), 7.36 (dd, *J* = 2.6 Hz, *J* = 9.2 Hz, 1H), 7.48 (d, *J* = 8.6 Hz, 2H), 7.79 (d, *J* = 4.7 Hz, 1H) 8.00 (d, *J* = 9.2 Hz, 1H), 8.35 (d, *J* = 8.6 Hz, 2H), 8.74 (d, *J* = 4.6 Hz, 1H) ppm.

2.2 (Asymmetric) (4+1) Cyclization Reaction

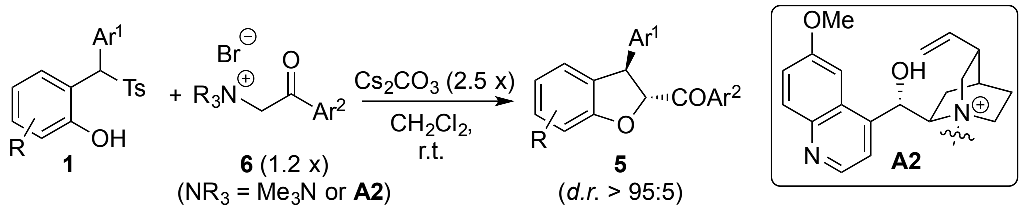

General procedure: Compound **1** (1 equiv.), ammonium salt **6** (1.2 equiv.) and Cs_2CO_3 (2.5 equiv.) are dissolved in DCM (15 mL per mmol **1**). The reaction mixture is either stirred over night (for achiral salts **6**) or for 3 days (when using chiral salts **6**) and afterwards extracted with DCM and brine. The combined organic phases are dried over Na_2SO_4, filtered and evaporated to dryness. The crude products are purified by column chromatography (silica gel, heptane:EtOAc first to obtain products **5** and DCM/MeOH then to recover the chiral amine **A2**).

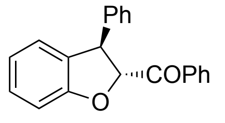

5c
Me3N: 95%
A2: 85%, *e.r.* = 99:1

Product 5c: Isolated as a white residue in the reported yields (1 mmol). $[\alpha]_D^{20}$ = -8.8 (c = 0.2, DCM, *e.r.* = 99:1), R_f: 0.53 (heptane:EtOAc = 5:1). ^{1}H NMR (700 MHz, δ, $CDCl_3$, 298 K): 4.99 (d, *J* = 6.5 Hz, 1H), 5.82 (d, *J* = 6.4 Hz, 1H), 6.90 (t, *J* = 7.5 Hz, 1H), 7.01 – 6.98 (m, 2H), 7.24 – 7.20 (m, 3H), 7.30 – 7.28 (m, 1H), 7.35 – 7.33 (m, 2H), 7.46 (t, *J* = 7.7 Hz, 2H), 7.60 (t, *J* = 7.3 Hz, 1H), 7.96 (d, *J* = 7.6 Hz, 2H) ppm. ^{13}C NMR (176 MHz, δ, $CDCl_3$, 298 K): 51.0, 90.7, 110.1, 121.8, 125.5, 127.6, 128.3, 128.8, 129.0, 129.1, 129.5, 133.9, 134.6, 142.4, 159.2, 194.8 ppm. HRMS (ESI): *m/z* calculated for $C_{21}H_{16}O_2$: 323.1043 $[M+Na]^+$; found: 323.1040. The enantioselectivity is determined by HPLC (YMC Cellulose-SB, eluent: hexane:*i*-PrOH = 95:5, 0.5 mL/min, 10 °C, retention times: t_{major} (2R, 3R) = 15.7 min, t_{minor} (2S, 3S) = 17.0 min).

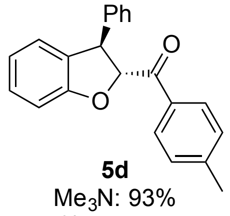

5d
Me3N: 93%
A2: 81%, *e.r.* = 98.5:1.5

Product 5d: Obtained as a yellow residue (0.1 mmol scale). $[\alpha]_D^{20}$ = -19.5 (c = 1.0, DCM, *e.r.* = 98.5:1.5), R_f: 0.35 (heptane:EtOAc = 10:1). ^{1}H NMR (700 MHz, δ, $CDCl_3$, 298 K): 4.97 (d, *J* = 6.3 Hz, 1H), 5.81 (d, *J* = 6.3 Hz, 1H), 6.89 (t, *J* = 7.4 Hz, 1H), 7.01 – 6.98 (m, 2H), 7.30 – 7.20 (m, 6H), 7.34 (t, *J* = 7.4 Hz, 2H), 7.86 (d, *J* = 8.0 Hz, 2H) ppm. ^{13}C NMR (176 MHz, δ, $CDCl_3$, 298 K): 21.9, 51.1, 90.6, 110.1, 121.7, 125.5, 127.5, 128.3, 129.0, 129.1, 129.5, 132.0, 142.5, 145.0, 159.2, 194.4 ppm. HRMS (ESI): *m/z* calculated for $C_{22}H_{18}O_2$: 337.1199 $[M+Na]^+$; found: 337.1197. The enantioselectivity is determined by HPLC (Chiralcel OD-H, eluent: hexane:*i*-PrOH = 95:5, 0.8 mL/min, 10 °C, retention times: t_{major} (2R, 3R) = 12.6 min, t_{minor} (2S, 3S) = 10.8 min).

Product **5e**: Obtained as a beige residue (0.1 mmol scale). $[\alpha]_D^{20}$ = -15.0 (c = 0.5, DCM, *e.r.* = 97:3), R_f: 0.27 (heptane:EtOAc = 10:1). ^{1}H NMR (700 MHz, δ, $CDCl_3$, 298 K): 3.87 (s, 3H), 4.99 (d, *J* = 6.6 Hz, 1H), 5.77 (d, *J* = 6.6 Hz, 1H), 6.89 (t, *J* = 7.5 Hz, 1H), 6.92 (d, *J* = 8.7 Hz, 2H), 7.01 – 6.97 (m, 2H), 7.20 (t, *J* = 7.8 Hz, 1H), 7.25 (t, *J* = 7.6 Hz, 2H), 7.29 – 7.27 (m, 1H), 7.35 – 7.33 (m, 2H), 7.94 (d, *J* = 8.9 Hz, 2H) ppm. ^{13}C NMR (176 MHz, δ, $CDCl_3$, 298 K): 51.1, 55.7, 90.7, 110.1, 114.1, 121.7, 125.5, 127.6, 128.4, 129.0, 129.2, 129.6, 131.9, 142.5, 159.3, 164.2, 193.3 ppm. HRMS (ESI): *m/z* calculated for $C_{22}H_{18}O_3$: 353.1148 $[M+Na]^+$; found: 353.1145. The enantioselectivity is determined by HPLC (Chiralcel OD-H, eluent: hexane:MeOH = 100:1, 0.5 mL/min, 10 °C, retention times: t_{major} (2R, 3R) = 41.5 min, t_{minor} (2S, 3S) = 39.6 min).

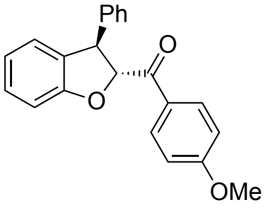

5e
Me_3N: 87%
A2: 79%, *e.r.* = 97:3

Product **5f**: Isolated as a white residue is obtained (0.1 mmol scale). $[\alpha]_D^{20}$ = -15 (c = 0.5, DCM, *e.r.* = 99:1), R_f: 0.47 (heptane:EtOAc = 10:1). ^{1}H NMR (300 MHz, δ, $CDCl_3$, 298 K): 4.99 (d, *J* = 6.8 Hz, 1H), 5.72 (d, *J* = 6.6 Hz, 1H), 7.00 – 6.85 (m, 3H), 7.23 – 7.16 (m, 3H), 7.35 – 7.26 (m, 3H), 7.42 – 7.39 (m, 2H), 7.88 (d, *J* = 8.7 Hz, 2H) ppm. ^{13}C NMR (75 MHz, δ, $CDCl_3$, 298 K): 50.7, 90.7, 110.0, 121.8, 125.4, 127.6, 128.2, 129.0, 129.1, 129.2, 130.8, 132.9, 140.4, 142.1, 158.9, 193.6 ppm. HRMS (ESI): *m/z* calculated for $C_{21}H_{15}ClO_2$: 357.0653 $[M+Na]^+$; found: 357.0650. The enantioselectivity is determined by HPLC (YMC Cellulose-SB, eluent: hexane:*i*-PrOH = 95:5, 0.5 mL/min, 10 °C, retention times: t_{major} (2R, 3R) = 17.1 min, t_{minor} (2S, 3S) = 15.8 min).

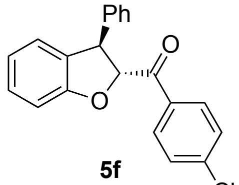

5f
Me_3N: 91%
A2: 88%, *e.r.* = 99:1

Product **5g**: Isolated as a white residue (0.1 mmol scale). $[\alpha]_D^{20}$ = -16.5 (c = 0.5, DCM, *e.r.* = 90:10), R_f: 0.48 (heptane:EtOAc = 5:1). ^{1}H NMR (700 MHz, δ, $CDCl_3$, 298 K): 3.78 (s, 3H), 4.90 (d, *J* = 6.2 Hz, 1H), 5.76 (d, *J* = 6.5 Hz, 1H), 6.88 – 6.84 (m, 3H), 6.97 (t, *J* = 7.8 Hz, 2H), 7.13 (d, *J* = 8.6 Hz, 2H), 7.19 (t, *J* = 7.8 Hz, 1H), 7.44 (t, *J* = 7.8 Hz, 2H), 7.58 (t, *J* = 7.4 Hz, 1H), 7.92 (d, *J* = 7.7 Hz, 2H) ppm. ^{13}C NMR (176 MHz, δ, $CDCl_3$, 298 K): 50.5, 55.5, 90.9, 110.1, 114.5, 121.7, 125.4, 128.8, 129.0, 129.3, 129.4, 129.6, 133.9, 134.5, 134.6, 159.1, 195.0 ppm. HRMS (ESI): *m/z* calculated for $C_{22}H_{18}O_3$: 353.1148 $[M+Na]^+$; found: 353.1147. The enantioselectivity is determined by HPLC (YMC Cellulose-SB, eluent: hexane:*i*-PrOH = 95:5, 0.5 mL/min, 10 °C, retention times: t_{major} (2R, 3R) = 21.7 min, t_{minor} (2S, 3S) = 24.7 min).

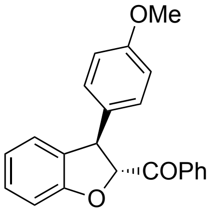

5g
Me_3N: 82%
A2: 96%, *e.r.* = 90:10

Product **5h**: Obtained as a light yellow residue (0.1 mmol scale). $[\alpha]_D^{20}$ = -12.7 (c = 0.8, DCM, *e.r.* = 98:2), R_f: 0.56 (heptane:EtOAc = 5:1). ^{1}H NMR (300 MHz, δ, $CDCl_3$, 298 K): 5.12 (d, *J* = 6.7 Hz, 1H), 5.74 (d, *J* = 6.5 Hz, 1H), 7.00 – 6.88 (m, 3H), 7.24 – 7.19 (m, 1H), 7.35 (d, *J* = 8.1 Hz, 2H), 7.46 (t, *J* = 7.6 Hz, 2H), 7.62 – 7.57 (m, 3H), 7.98 – 7.95 (m, 2H) ppm. ^{13}C NMR (75 MHz, δ, $CDCl_3$, 298 K): 50.3, 90.3, 110.2, 121.9, 125.3, 126.0, 128.5, 128.6, 128.8, 129.3, 129.4, 134.0, 134.4, 146.2, 159.0, 194.3 ppm. ^{19}F NMR (282 MHz, δ, $CDCl_3$, 298 K): -62.5 ppm. HRMS (ESI): *m/z* calculated for $C_{22}H_{15}F_3O_2$: 391.0916 $[M+Na]^+$; found: 391.0919. The enantioselectivity is determined by HPLC (YMC Cellulose-SB, eluent: hexane:*i*-

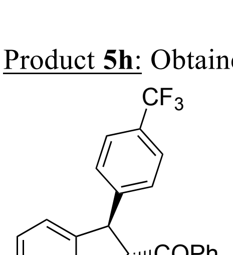

5h
Me_3N: 88%
A2: 23%, *e.r.* = 98:2

PrOH = 250:1, 0.5 mL/min, 10 °C, retention times: t_{major} (2R, 3R) = 49.8 min, t_{minor} (2S, 3S) = 56.9 min).

Product 5i: Obtained as a yellow residue (0.1 mmol scale). $[\alpha]_D^{20}$ = -15.9 (c = 0.7, DCM, *e.r.* = 93:7), R_f: 0.40 (heptane:EtOAc = 5:1). ^{1}H NMR (300 MHz, δ, $CDCl_3$, 298 K): 6.05 – 5.86 (m, 2H), 6.99 – 6.89 (m, 2H), 7.12 – 7.07 (m, 1H), 7.23 – 7.20 (m, 1H), 7.59 – 7.38 (m, 7H), 7.80 (d, *J* = 8.2 Hz, 1H), 7.91 – 7.88 (m, 1H), 7.98 (d, *J* = 7.6 Hz, 2H), 8.12 (s, 1H) ppm. ^{13}C NMR (75 MHz, δ, $CDCl_3$, 298 K): 44.3, 90.0, 110.1, 121.8, 123.2, 125.7, 125.9, 126.6, 128.1, 128.6, 128.9, 129.3, 129.6, 131.4, 133.8, 134.7, 159.0, 194.9 ppm. HRMS (ESI): *m/z* calculated for $C_{25}H_{18}O_2$: 373.1199 $[M+Na]^+$; found: 373.1198. The enantioselectivity is determined by HPLC (Chiralcel OD-H, eluent: hexane:*i*-PrOH:MeOH = 100:3:1, 0.8 mL/min, 10 °C, retention times: t_{major} (2R, 3R) = 13.4 min, t_{minor} (2S, 3S) = 12.3 min).

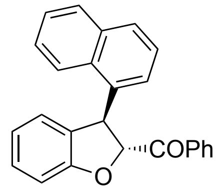

5i
Me_3N: 85%
A2: 81%, *e.r.* = 93:7

Product 5j: Obtained as a light yellow residue (0.1 mmol scale). $[\alpha]_D^{20}$ = -12.7 (c = 0.8, DCM, *e.r.* = 97:3), R_f: 0.57 (heptane:EtOAc = 10:1). ^{1}H NMR (300 MHz, δ, $CDCl_3$, 298 K): 1.32 (s, 9H), 4.98 (d, *J* = 6.4 Hz, 1H), 5.81 (d, *J* = 6.4 Hz, 1H), 6.93 – 6.87 (m, 1H), 7.05 – 6.97 (m, 2H), 7.24 – 7.14 (m, 3H), 7.37 – 7.34 (m, 2H), 7.49 – 7.44 (m, 2H), 7.63 – 7.57 (m, 1H), 7.98 – 7.95 (m, 2H) ppm. ^{13}C NMR (75 MHz, δ, $CDCl_3$, 298 K): 31.4, 34.6, 50.4, 90.7, 110.0, 121.6, 125.4, 125.9, 127.8, 128.7, 128.8, 129.4, 133.8, 134.5, 139.1, 150.4, 159.1, 194.8 ppm. HRMS (ESI): *m/z* calculated for $C_{25}H_{24}O_2$: 379.1669 $[M+Na]^+$; found: 379.1667. The enantioselectivity is determined by HPLC (YMC Cellulose-SB, eluent: hexane:*i*-PrOH = 99:1, 0.5 mL/min, 10 °C, retention times: t_{major} (2R, 3R) = 20.8 min, t_{minor} (2S, 3S) = 24.0 min).

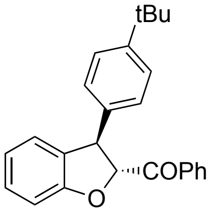

5j
Me_3N: 77%
A2: 80%, *e.r.* = 97:3

Product 5k: Obtained as a brown residue (0.1 mmol scale). $[\alpha]_D^{20}$ = -18.3 (c = 0.6, DCM, *e.r.* = 93:7), R_f: 0.45 (heptane:EtOAc = 5:1). ^{1}H NMR (300 MHz, δ, $CDCl_3$, 298 K): 3.75 (s, 3H), 5.98 – 5.74 (m, 2H), 6.90 – 6.77 (m, 4H), 6.99 (m, 1H), 7.17 – 7.11 (m, 2H), 7.32 (t, J = 7.6 Hz, 1H), 7.41 – 7.39 (m, 2H), 7.70 (d, *J* = 8.5 Hz, 1H), 7.82 – 7.79 (m, 1H), 7.90 – 7.87 (m, 2H), 8.05 (s, 1H) ppm. ^{13}C NMR (75 MHz, δ, $CDCl_3$, 298 K): 44.4, 55.5, 90.0, 110.0, 113.9, 121.7, 113.4, 125.7, 125.9, 126.6, 127.6, 128.0, 128.8, 129.0, 129.4, 131.5, 131.9, 134.1, 159.0, 164.0, 193.2 ppm. HRMS (ESI): *m/z* calculated for $C_{26}H_{20}O_3$: 403.1305 $[M+Na]^+$; found: 403.1301. The enantioselectivity is determined by HPLC (Chiralcel OD-H, eluent: hexane:*i*-PrOH = 95:5, 0.5 mL/min, 10 °C, retention times: t_{major} (2R, 3R) = 40.4 min, t_{minor} (2S, 3S) = 31.9 min).

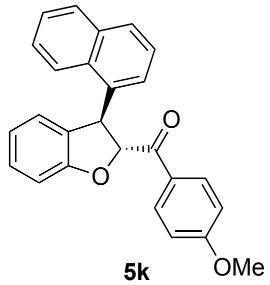

5k
Me_3N: 83%
A2: 69%, *e.r.* = 93:7

Product **5l**: Obtained as a light brown residue (0.1 mmol scale). $[\alpha]_D^{20}$ = -18.3 (c = 0.8, DCM, *e.r.* = 93:7), R_f: 0.64 (heptane:EtOAc = 5:1). ^{1}H NMR (700 MHz, δ, $CDCl_3$, 298 K): 2.38 (s, 3H), 6.08 – 5.79 (m, 2H), 6.96 – 6.88 (m, 2H), 7.22 – 7.19 (m, 4H), 7.39 (t, *J* = 7.7 Hz, 1H), 7.49 – 7.48 (m, 2H), 7.89 – 7.78 (m, 5H), 8.16 (s, 1H) ppm. ^{13}C NMR (176 MHz, δ, $CDCl_3$, 298 K): 21.9, 44.4, 90.3, 110.1, 121.8, 123.4, 125.8, 126.0, 126.7, 128.0, 129.0, 129.5, 129.8, 131.6, 132.2, 144.9, 159.1, 194.5 ppm. HRMS (ESI): *m/z* calculated for $C_{26}H_{20}O_2$: 387.1356 [M+Na]$^+$; found: 387.1355. The enantioselectivity is determined by HPLC (YMC Cellulose-SB, eluent: hexane:*i*-PrOH = 99:1, 0.5 mL/min, 10 °C, retention times: t_{major} (2R, 3R) = 43.3 min, t_{minor} (2S, 3S) = 32.4 min).

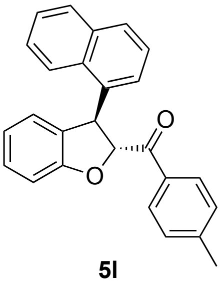

5l
Me_3N: 85%
A2: 68%, *e.r.* = 93:7

Product **5m**: Obtained as a yellow residue (0.1 mmol scale). $[\alpha]_D^{20}$ = -13.2 (c = 0.9, DCM, *e.r.* = 94:6), R_f: 0.52 (heptane:EtOAc = 5:1). ^{1}H NMR (300 MHz, δ, $CDCl_3$, 298 K): 5.92 – 5.64 (m, 2H), 6.84 – 6.76 (m, 2H), 6.96 – 6.93 (m, 1H), 7.12 – 7.06 (m, 2H), 7.30 – 7.23 (m, 2H), 7.37 – 7.34 (m, 2H), 7.60 – 7.53 (m, 1H), 7.66 (d, *J* = 8.3 Hz, 1H), 7.81 – 7.74 (m, 3H), 8.01 (s, 1H) ppm. ^{13}C NMR (75 MHz, δ, $CDCl_3$, 298 K): 44.2, 90.0, 110.1, 112.5, 121.9, 123.2, 124.1, 125.2, 125.7, 126.7, 128.1, 128.5, 129.0, 130.7, 131.0, 131.4, 133.0, 134.2, 140.3, 158.8, 193.9 ppm. HRMS (ESI): *m/z* calculated for $C_{25}H_{17}ClO_2$: 407.0809 [M+Na]$^+$; found: 407.0806. The enantioselectivity is determined by HPLC (YMC Cellulose-SB, eluent: hexane:*i*-PrOH = 99:1, 0.5 mL/min, 10 °C, retention times: t_{major} (2R, 3R) = 56.0 min, t_{minor} (2S, 3S) = 35.1 min).

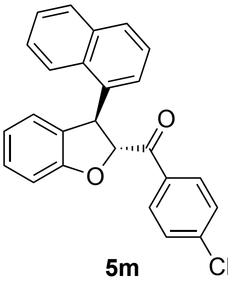

5m
Me_3N: 79%
A2: 73%, *e.r.* = 94:6

Product **5n**: Isolated as a light yellow residue. $[\alpha]_D^{20}$ = -21.2 (c = 0.3, DCM, *e.r.* = 93:7), R_f: 0.55 (heptane:EtOAc = 5:1). ^{1}H NMR (700 MHz, δ, $CDCl_3$, 298 K): 3.80 (s, 3H), 4.94 (d, *J* = 6.7 Hz, 1H), 5.70 (d, *J* = 6.7 Hz, 1H), 6.91 – 6.87 (m, 3H), 6.96 (d, *J* = 8.1 Hz, 1H), 7.00 (d, *J* = 7.6 Hz, 1H), 7.15 (d, *J* = 8.6 Hz, 2H), 7.21 (t, *J* = 7.7 Hz, 1H), 7.44 (d, *J* = 8.7 Hz, 2H)), 7.90 (d, *J* = 8.4 Hz, 2H) ppm. ^{13}C NMR (176 MHz, δ, $CDCl_3$, 298 K): 50.3, 55.5, 91.0, 110.1, 114.5, 121.9, 125.5, 129.0, 129.2, 129.4, 129.5, 130.9, 133.0, 134.2, 140.5, 158.9, 159.1, 194.0 ppm. HRMS (ESI): *m/z* calculated for $C_{22}H_{17}ClO_3$: 387.0758 [M+Na]$^+$; found: 387.0756. The enantioselectivity is determined by HPLC (Chiralcel OD-H, eluent: hexane:*i*-PrOH = 95:5, 0.5 mL/min, 10 °C, retention times: t_{major} (2R, 3R) = 23.4 min, t_{minor} (2S, 3S) = 19.9 min).

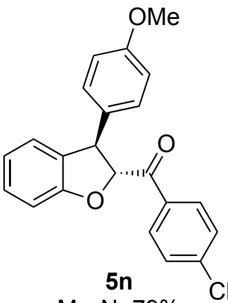

5n
Me_3N: 79%
A2: 88%, *e.r.* = 93:7

Product **5o**: Isolated as a brown residue (0.1 mmol scale). $[\alpha]_D^{20}$ = -6.5 (c = 1.0, DCM, *e.r.* = 98.5:1.5), R_f: 0.21 (heptane:EtOAc = 5:1). ^{1}H NMR (700 MHz, δ, $CDCl_3$, 298 K): 3.76 (s, 3H), 3.83 (s, 3H), 4.88 (d, *J* = 6.8 Hz, 1H), 5.69 (d, *J* = 6.6 Hz, 1H), 6.85 – 6.82 (m, 3H), 6.88 (d, *J* = 8.5 Hz, 2H), 6.95 – 6.92 (m, 2H), 7.11 (d, *J* = 8.6 Hz, 2H), 7.15 (t, *J* = 7.7 Hz, 1H), 7.89 (d, *J* = 8.7 Hz, 2H) ppm. ^{13}C NMR (176 MHz, δ, $CDCl_3$, 298 K): 50.6, 55.5, 55.7, 90.8, 110.1, 114.1, 114.5, 121.7, 125.4, 127.6, 128.9, 129.4, 129.8, 131.8, 134.6, 159.1, 159.2, 164.2, 193.5 ppm. HRMS (ESI): *m/z* calculated for $C_{23}H_{20}O_4$: 383.1254 [M+Na]$^+$; found: 383.1253. The enantioselectivity is determined by HPLC (YMC

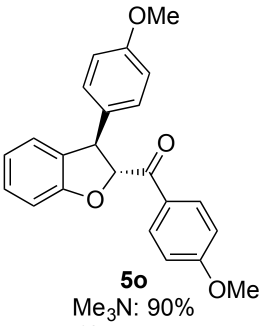

5o
Me_3N: 90%
A2: 82%, *e.r.* = 98.5:1.5

Cellulose-SB, eluent: hexane:*i*-PrOH = 98:2, 0.5 mL/min, 10 °C, retention times: t_{major} (2R, 3R) = 67.4 min, t_{minor} (2S, 3S) = 73.7 min).

Product **5p**: Obtained as a pale oil (0.1 mmol scale). $[\alpha]_D^{20}$ = -9.9 (c = 1.0, DCM, *e.r.* = 99:1), R_f: 0.54 (heptane:EtOAc = 5:1). ^{1}H NMR (300 MHz, δ, $CDCl_3$, 298 K): 4.97 (d, *J* = 6.4 Hz, 1H), 5.84 (d, *J* = 6.5 Hz, 1H), 6.86 (d, J = 8.6 Hz, 1H), 7.11 – 7.10 (m, 1H), 7.24 – 7.20 (m, 2H), 7.39 – 7.28 (m, 4H), 7.49 – 7.44 (m, 2H), 7.63 – 7.58 (m, 1H), 7.95 – 7.92 (m, 2H) ppm. ^{13}C NMR (75 MHz, δ, $CDCl_3$, 298 K): 50.6, 91.0, 111.6, 113.5, 127.8, 128.1, 128.3, 128.8, 129.2, 129.3, 131.8, 134.0, 134.2, 141.4, 158.2, 194.1 ppm. HRMS (ESI): *m/z* calculated for $C_{21}H_{15}BrO_2$: 401.0148 $[M+Na]^+$; found: 401.0147. The enantioselectivity is determined by HPLC (YMC Cellulose-SB, eluent: hexane:*i*-PrOH = 98:2, 0.5 mL/min, 10 °C, retention times: t_{major} (2R, 3R) = 24.4 min, t_{minor} (2S, 3S) = 26.2 min).

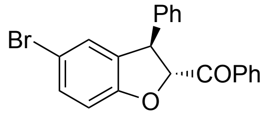

5p
Me_3N: 81%
A2: 98%, *e.r.* = 99:1

Product **5q**: Obtained as a light yellow residue (0.1 mmol scale). $[\alpha]_D^{20}$ = -8.4 (c = 0.8, DCM, *e.r.* = 99:1), R_f: 0.52 (heptane:EtOAc = 10:1). ^{1}H NMR (700 MHz, δ, $CDCl_3$, 298 K): 1.24 (s, 9H), 4.99 (d, *J* = 6.3 Hz, 1H), 5.77 (d, *J* = 6.6 Hz, 1H), 6.90 (d, *J* = 8.7 Hz, 1H), 7.03 (s, 1H), 7.26 – 7.23 (m, 3H), 7.29 (t, *J* = 7.4 Hz, 1H), 7.35 (t, *J* = 7.4 Hz, 2H), 7.45 (t, *J* = 7.8 Hz, 1H), 7.59 (t, *J* = 7.4 Hz, 1H), 7.95 (d, *J* = 7.6 Hz, 2H) ppm. ^{13}C NMR (176 MHz, δ, $CDCl_3$, 298 K): 31.8, 34.5, 51.2, 91.0, 109.2, 122.3, 125.9, 127.5, 128.4, 128.8, 129.1, 129.5, 133.9, 134.7, 142.5, 144.9, 157.1, 195.0 ppm. HRMS (ESI): *m/z* calculated for $C_{25}H_{24}O_2$: 379.1669 $[M+Na]^+$; found: 379.1667. The enantioselectivity is determined by HPLC (YMC Cellulose-SB, eluent: hexane:*i*-PrOH = 250:1, 0.5 mL/min, 10 °C, retention times: t_{major} (2R, 3R) = 33.9 min, t_{minor} (2S, 3S) = 31.2 min).

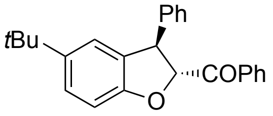

5q
Me_3N: 84%
A2: 92%, *e.r.* = 99:1

Product **5r**: Isolated as an orange residue (0.1 mmol scale). $[\alpha]_D^{20}$ = -12.7 (c = 0.4, DCM, *e.r.* = 97:3), R_f: 0.64 (heptane:EtOAc = 5:1). ^{1}H NMR (300 MHz, δ, $CDCl_3$, 298 K): 2.32 (s, 3H), 5.01 (d, *J* = 6.5 Hz, 1H), 5.79 (d, *J* = 6.5 Hz, 1H), 6.86 – 6.78 (m, 2H), 7.05 – 7.02 (m, 1H), 7.37 – 7.23 (m, 5H), 7.46 (t, *J* = 7.5 Hz, 2H), 7.63 – 7.57 (m, 1H), 8.00 – 7.97 (m, 2H) ppm. ^{13}C NMR (75 MHz, δ, $CDCl_3$, 298 K): 15.3, 51.3, 90.5, 120.2, 121.5, 122.7, 127.4, 128.2, 128.5, 128.6, 129.0, 129.3, 130.1, 133.7, 134.6, 142.4, 157.6, 195.1 ppm. HRMS (ESI): *m/z* calculated for $C_{22}H_{18}O_2$: 337.1199 $[M+Na]^+$; found: 337.1195. The enantioselectivity is determined by HPLC (YMC Cellulose-SB, eluent: hexane:*i*-PrOH = 95:5, 0.5 mL/min, 10 °C, retention times: t_{major} (2R, 3R) = 13.9 min, t_{minor} (2S, 3S) = 17.1 min).

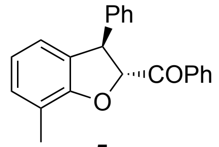

5r
Me_3N: 83%
A2: 95%, *e.r.* = 97:3

Product **5s**: Obtained as a yellow residue (0.1 mmol scale). $[\alpha]_D^{20}$ = -5.3 (c = 0.8, DCM, *e.r.* = 66:34), R_f: 0.47 (heptane:EtOAc = 5:1). ^{1}H NMR (300 MHz, δ, $CDCl_3$, 298 K): 5.29 (d, *J* = 5.3 Hz, 1H), 5.91 (d, *J* = 5.4 Hz, 1H), 7.29 – 7.20 (m, 9H), 7.45 (t, *J* = 7.6 Hz, 2H), 7.59 (t, *J* = 7.4 Hz, 1H), 7.77 – 7.74 (m, 2H), 7.97 – 7.94 (m, 2H) ppm. ^{13}C NMR (75 MHz, δ, $CDCl_3$, 298 K): 50.7, 91.6, 112.1, 119.9, 122.8, 123.2, 126.9, 127.5, 128.0, 128.8, 128.9, 129.1, 129.4, 130.1, 130.4, 130.6, 133.8, 134.3, 142.4, 157.2, 194.7 ppm. HRMS (ESI): *m/z* calculated for $C_{25}H_{18}O_2$: 373.1199 $[M+Na]^+$; found: 373.1198. The enantioselectivity is determined by HPLC (YMC Cellulose-SB, eluent: hexane:*i*-PrOH = 95:5, 0.5 mL/min, 10 °C, retention times: t_{major} (2R, 3R) = 27.9 min, t_{minor} (2S, 3S) = 25.6 min).

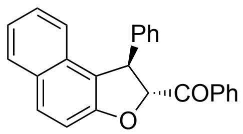

5s
Me_3N: 86%
A2: 60%, *e.r.* = 66:34

Product **5t**: Obtained as a white residue (0.1 mmol scale). $[\alpha]_D^{20}$ = 2.3 (c = 0.8, DCM, *e.r.* = 73:27), R_f: 0.44 (heptane:EtOAc = 5:1). ^{1}H NMR (300 MHz, δ, $CDCl_3$, 298 K): 4.94 (d, *J* = 4.5 Hz, 1H), 5.89 (d, *J* = 4.5 Hz, 1H), 6.86 (d, *J* = 8.1 Hz, 1H), 6.94 (d, *J* = 8.1 Hz, 1H), 7.17 (t, *J* = 8.0 Hz, 1H), 7.24 – 7.21 (m, 1H), 7.39 – 7.28 (m, 3H), 7.49 (t, *J* = 7.5 Hz, 2H), 7.63 (t, *J* = 7.5 Hz, 1H), 7.96 – 7.93 (m, 2H) ppm. ^{13}C NMR (75 MHz, δ, $CDCl_3$, 298 K): 50.8, 90.6, 108.6, 122.2, 127.6, 128.0, 128.9, 129.0, 129.3, 130.4, 131.4, 133.8, 134.0, 140.9, 160.3, 194.1 ppm. HRMS (ESI): *m/z* calculated for $C_{21}H_{15}ClO_2$: 357.0653 $[M+Na]^+$; found: 357.0651. The enantioselectivity is determined by HPLC (YMC Cellulose-SB, eluent: hexane:*i*-PrOH = 95:5, 0.5 mL/min, 10 °C, retention times: t_{major} (2R, 3R) = 22.5 min, t_{minor} (2S, 3S) = 18.9 min).

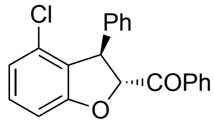

5t
Me_3N: 82%
A2: 63%, *e.r.* = 73:27

Product **5u**: Obtained as a white residue (0.1 mmol scale). R_f: 0.44 (heptane:EtOAc = 5:1). ^{1}H NMR (300 MHz, δ, $CDCl_3$, 298 K): 3.69 (s, 3), 4.99 (d, *J* = 6.5 Hz, 1H), 5.78 (d, *J* = 6.5 Hz, 1H), 6.57 (d, *J* = 2.2 Hz, 1H), 6.76 (dd, *J* = 2.2, 8.7 Hz, 1H), 6.89 (d, *J* = 8.7 Hz, 1H), 7.22 – 7.28 (m, 2H), 7.29 – 7.39 (m, 3H), 7.41 – 7.50 (m, 2H), 7.55 – 7.63 (m, 1H), 7.92 – 7.99 (m, 2H) ppm. ^{13}C NMR (75 MHz, δ, $CDCl_3$, 298 K): 51.1, 55.9, 90.8, 110.0, 110.8, 114.4, 127.4, 128.1, 128.6, 128.9, 129.3, 130.0, 133.7, 134.4, 141.9, 153.1, 154.9, 194.7 ppm. LRMS (ESI): *m/z* calculated for $C_{22}H_{18}O_3$: 331.13 $[M+H]^+$; found: 331.2. The enantioselectivity is determined by HPLC (YMC Cellulose-SB, eluent: hexane:*i*-PrOH = 100:1, 0.5 mL/min, 10 °C, retention times: t_{major} (2R, 3R) = 34.0 min, t_{minor} (2S, 3S) = 45.9 min).

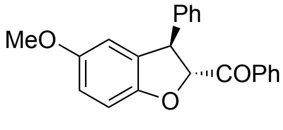

5u
Me_3N: 83%
A2: 68%, *d.r.* = 93:7, *e.r.* = 99:1

Product **5v**: Obtained as a colourless residue (0.1 mmol scale). R_f: 0.49 (heptane:EtOAc = 5:1). ^{1}H NMR (300 MHz, δ, $CDCl_3$, 298 K): 1.50 (d, *J* = 6.9 Hz, 3H), 3.84 (dq, *J* = 6.9, 6.9 Hz, 1H), 5.42 (d, *J* = 6.9 Hz, 1H), 6.86 – 6.97 (m, 2H), 7.12 – 7.22 (m, 2H), 7.45 – 7.56 (m, 2H), 7.58 – 7.66 (m, 1H), 8.01 – 8.07 (m, 2H) ppm. ^{13}C NMR (75 MHz, δ, $CDCl_3$, 298 K): 20.0, 39.8, 90.4, 109.8, 121.2, 123.9, 128.4, 128.6, 129.1, 130.5, 133.6, 134.8, 158.4, 195.9 ppm. HRMS (ESI): *m/z* calculated for $C_{16}H_{14}O_2$: 239.11 $[M+H]^+$; found: 239.1. The enantioselectivity is determined by HPLC (YMC Cellulose-SB, eluent: hexane:*i*-PrOH = 100:1, 0.5 mL/min, 10 °C, retention times: t_{major} (2R, 3R) = 27.1 min, t_{minor} (2S, 3S) = 31.6 min).

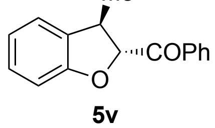

5v
Me_3N: 99%, *d.r.* = 90:10
A2: 55%, *d.r.* = 89:11, *e.r.* = 65:35

3. Computational Details, Energies and Geometries

3.1 Conformational study of betaines

For *trans* pathway, addition occurs via a *cisoid* approach of reactants. The betaine formed initially is therefore in a *cisoid* conformation. In order for the second key step to occur, this betaine needs to undergo rotation around the newly formed carbon-carbon bond to give the corresponding *transoid* conformer (Figure S1). The computed free energy of the transition state for this rotational equilibrium is 6.3 kcal.mol^{-1}, i.e. lower than TS to ring closure. It is thus not relevant for the reactivity and selectivity.
Addition to form *cis* betaine occurs via a *transoid* approach. The betaine formed is thus in a conformation allowing cyclization.

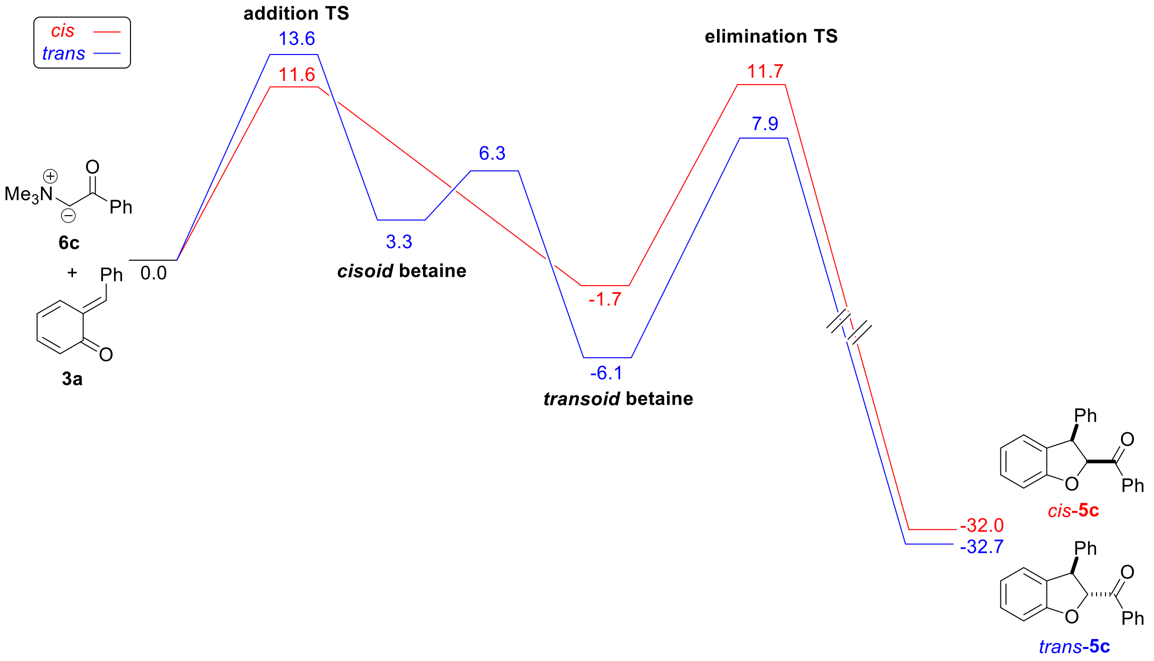

Figure S1. Computed free energy profile including conformational equilibrium for betaines.

3.2. Addition on the aromatic ring

Addition can potentially not only occur on the methylene position of the electrondeficient o-quinone methide but also onto electrophilic C3 and C5 carbon atoms of the quinone ring. Free energy barrier for these different addition mode are reported in Table S1. These data are in good agreement with the observed exclusive addition onto the methylene group.

Table S1: Free energy barrier to addition (kcal.mol^{-1})

**Addition mode**	**TSadd-*trans***	**TSadd-*cis***
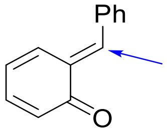	13.6	11.6
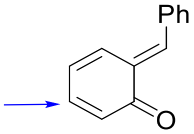	30.2	26.8
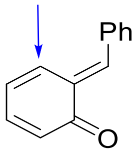	30.5	30.6

3.3. Benchmark calculations

Free energy of key transition states has been obtained at different levels of theory (Table S2).

Table S2: Relative free energy (kcal.mol^{-1}) of key TSs.

	**B3LYP-3/ 6-31G***	**B3LYP-D3/ 6-311+G****	**B3LYP-D3/ cc-pVTZ**	**M06-2X-D3/ 6-311+G****	**SCS-MP2/ 6-31+G****
trans addition TS	20.6	13.6	14.3	14.0	13.3
cis addition TS	9.0	11.6	12.5	12.3	11.3
trans elimination TS	3.1	7.9	8.8	6.4	2.5
cis alimination TS	6.7	11.7	12.2	11.2	7.8

3.4. Epimerization process

The full computed free energy profile for epimerization of *cis*-**A** via a fully intramolecular mechanism is depicted in Figure S2. It should be noted that protonation and/or deprotonation steps may also well occur via a pathway involving an intermolecular process (through *cis*-**C** or *trans*-**C** for instance).

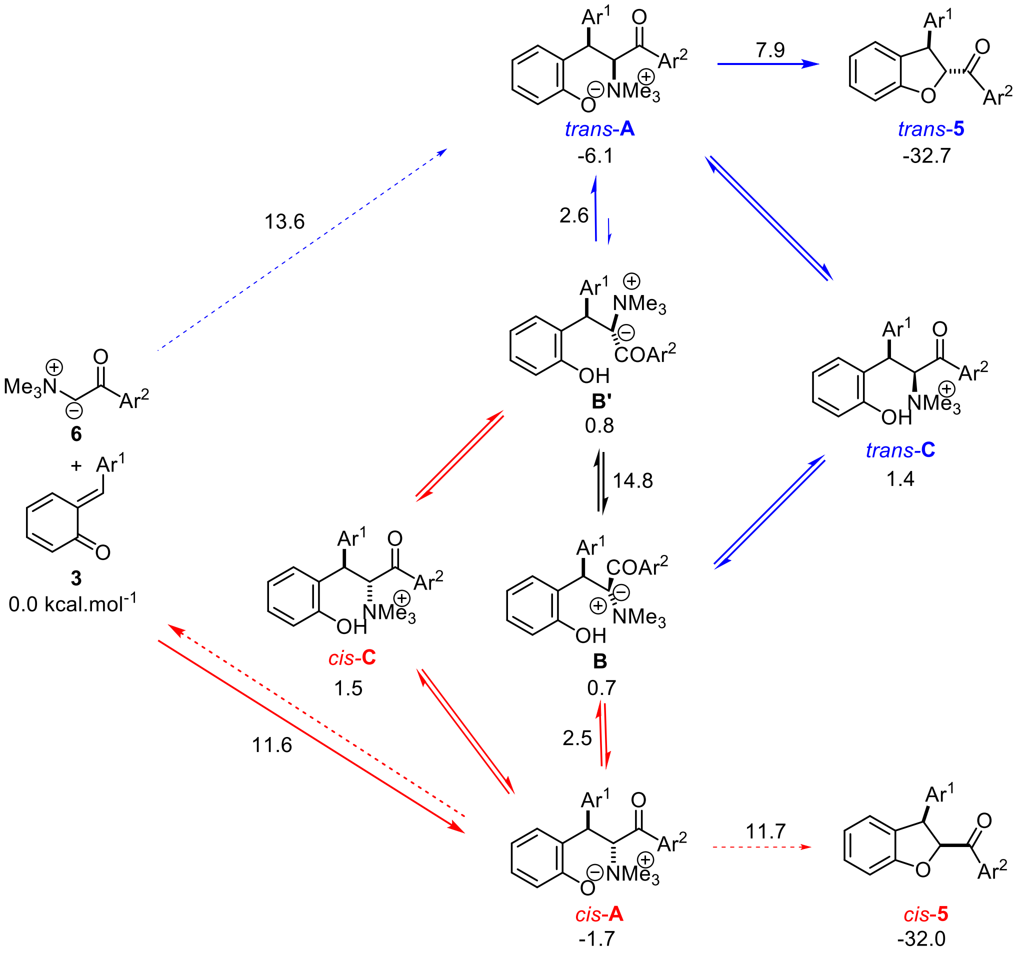

Figure S2. Some potential pathways for the isomerization of *cis*-**A** into *trans*-**A** (free energies in kcal.mol^{-1}).[4]

[4] The free energy of *cis*-**C** and *trans*-**C** was computed taking **1a** as proton source.

3.5. Computational results – Energies and Geometries

o-quinone methide

E(B3LYP-D3/6-31G*(dichloromethane)) = -576.628033
E(B3LYP-D3/6-311+G**(dichloromethane)) = -576.779513
Gtot(B3LYP/6-31G*(dichloromethane)) = -576.473748

C	0.145308	-0.190484	6.4747511177
C	-0.669118	1.021686	6.4888561200
C	-2.161579	0.821820	6.5478346086
C	-2.677254	-0.530285	6.6534864429
C	-1.846578	-1.605962	6.6533960343
C	-0.416669	-1.427504	6.5431426692
H	1.221620	-0.050343	6.4140790187
O	-0.147982	2.149577	6.4323940367
H	-3.744194	-0.673441	6.7897195482
H	-2.246044	-2.611144	6.7616688608
H	0.217307	-2.312640	6.5371977932
C	-2.931434	1.955751	6.5454242708
H	-2.369066	2.887242	6.6162881429
C	-7.161652	2.543655	6.4604986778
C	-6.321472	3.442723	7.1262752277
C	-4.944046	3.239500	7.1249237486
C	-4.378838	2.111054	6.4896877416
C	-5.238650	1.227084	5.8007041372
C	-6.614538	1.445230	5.7880359281
H	-8.236644	2.709296	6.4494955823
H	-6.741229	4.306680	7.6363041668
H	-4.290509	3.945040	7.6346900757
H	-4.823526	0.399228	5.2346445469
H	-7.262504	0.762898	5.2429271641

Ylide

E(B3LYP-D3/6-31G*(dichloromethane)) = -558.160579
E(B3LYP-D3/6-311+G**(dichloromethane)) = -558.320133
Gtot(B3LYP/6-31G*(dichloromethane)) = -557.955608

C	0.525316	0.193593	6.893280
C	-0.172952	1.375168	6.589294
C	-1.515428	1.270064	6.196551
C	-2.132732	0.023946	6.072465
C	-1.420436	-1.145428	6.356264
C	-0.089798	-1.054197	6.774715
H	1.551489	0.251274	7.246471
H	-2.063680	2.184233	5.985951
H	-3.171297	-0.037238	5.752938
H	-1.899949	-2.117576	6.261293
H	0.467855	-1.956690	7.017988
C	0.451136	2.757919	6.662126
O	-0.315788	3.759409	6.896600

C	1.813151	2.795506	6.426592
H	2.403598	1.951657	6.106129
N	2.569126	4.079894	6.421814
C	2.053088	4.995549	5.332272
H	2.606195	5.939225	5.355807
H	2.196268	4.489980	4.375713
H	0.992455	5.153549	5.526198
C	2.438952	4.778273	7.757085
H	2.982192	5.727010	7.722041
H	1.375753	4.935848	7.933723
H	2.859308	4.120339	8.519251
C	4.022380	3.789395	6.169814
H	4.580453	4.728592	6.174645
H	4.386810	3.126487	6.958106
H	4.118750	3.299475	5.197974

***Trans* addition TS**

E(B3LYP-D3/6-31G*(dichloromethane)) = -1134.799245
E(B3LYP-D3/6-311+G**(dichloromethane)) = -1134.803474
Gtot(B3LYP/6-31G*(dichloromethane)) = -1134.415533

C	-1.079249	5.155557	5.715079
C	-2.276070	4.455666	5.829085
C	-2.297409	3.061108	6.048085
C	-1.058719	2.403079	6.185425
C	0.140632	3.102380	6.066957
C	0.139093	4.477600	5.824750
H	-1.094231	6.230132	5.547443
H	-3.220050	4.992577	5.755024
H	-1.026247	1.346531	6.416731
H	1.082461	2.569147	6.174935
H	1.078188	5.019919	5.736196
C	-3.607818	2.416954	6.227389
H	-4.383614	3.118062	6.525010
C	-4.397127	2.662587	3.924400
H	-3.996768	3.663971	3.862598
C	-3.707562	1.587273	3.288504
O	-4.230263	0.474267	3.023201
N	-5.897829	2.703816	3.833329
C	-6.392378	3.986603	4.448302
H	-7.482058	4.014382	4.370563
H	-6.098528	4.006018	5.498609
H	-5.957787	4.833038	3.910653
C	-6.559744	1.552957	4.565206
H	-7.620882	1.554951	4.305004
H	-6.078775	0.632865	4.243071
H	-6.435091	1.713839	5.633998
C	-6.322571	2.671346	2.377386
H	-7.409806	2.772291	2.318816
H	-5.834849	3.500613	1.860806
H	-6.003986	1.718038	1.957788
C	0.511446	1.993354	2.456348
C	-0.063411	0.740105	2.688360

C	-1.428701	0.636788	2.948235
C	-2.241188	1.781684	3.000275
C	-1.654877	3.032130	2.749264
C	-0.290188	3.136636	2.480469
H	1.577043	2.077808	2.252723
H	0.554065	-0.155355	2.664900
H	-1.889243	-0.332063	3.117624
H	-2.258490	3.933962	2.752895
H	0.147916	4.113833	2.291841
C	-4.429017	-1.457024	7.716764
C	-5.236296	-0.404948	8.074897
C	-5.052676	0.925618	7.537867
C	-3.883317	1.098184	6.652209
C	-3.107994	-0.040107	6.279074
C	-3.360337	-1.289582	6.792475
H	-4.618700	-2.445987	8.133072
H	-6.071008	-0.544738	8.758530
O	-5.847296	1.871214	7.827119
H	-2.326177	0.082228	5.537209
H	-2.764918	-2.145206	6.483797

Cis **addition TS**

E(B3LYP-D3/6-31G*(dichloromethane)) = -1134.803474
E(B3LYP-D3/6-311+G**(dichloromethane)) = -1135.108222
Gtot(B3LYP/6-31G*(dichloromethane)) = -1134.417990

C	-1.240343	5.418226	5.775009
C	-2.490073	4.689822	5.797970
C	-2.394501	3.247218	6.104897
C	-1.112506	2.667961	6.362216
C	0.035284	3.418044	6.341922
C	-0.039181	4.806778	6.033596
H	-1.296324	6.480564	5.547297
O	-3.593422	5.254737	5.543457
H	-1.055348	1.616085	6.621941
H	0.996295	2.962128	6.567188
H	0.878687	5.393905	6.013042
C	-3.606718	2.536989	6.176509
H	-4.462690	3.179294	6.352333
C	-4.429010	2.629503	3.836128
H	-3.980032	3.610867	3.840698
C	-3.818703	1.534310	3.166506
O	-4.434147	0.497094	2.791524
N	-5.929168	2.757032	3.772040
C	-6.350650	4.056157	4.411521
H	-7.434366	4.151990	4.310146
H	-6.070407	4.050633	5.463415
H	-5.842965	4.880424	3.909431
C	-6.615988	1.619798	4.492020
H	-7.697288	1.741480	4.387050
H	-6.273682	0.690041	4.043603
H	-6.330356	1.657515	5.541742
C	-6.391562	2.778466	2.327035

H	-7.476011	2.918490	2.302043
H	-5.889680	3.609073	1.826694
H	-6.113582	1.829969	1.871080
C	0.466886	1.524449	2.599755
C	-0.277731	0.340483	2.552328
C	-1.658882	0.380258	2.723749
C	-2.325572	1.595470	2.961603
C	-1.568740	2.777891	2.989668
C	-0.184348	2.740223	2.811553
H	1.546980	1.496872	2.470068
H	0.221043	-0.612111	2.384849
H	-2.248719	-0.530794	2.691432
H	-2.045665	3.739039	3.146766
H	0.385563	3.665077	2.849668
C	-4.128653	-1.488846	7.557762
C	-4.916809	-0.446991	8.056114
C	-4.747618	0.849506	7.572153
C	-3.772982	1.137493	6.595690
C	-2.994338	0.076206	6.097442
C	-3.172731	-1.221604	6.573221
H	-4.261030	-2.501793	7.931652
H	-5.662625	-0.645026	8.822910
H	-5.358235	1.658402	7.970648
H	-2.261815	0.266613	5.322576
H	-2.563415	-2.028643	6.172032

***Cisoid Trans*-A**

E(B3LYP-D3/6-31G*(dichloromethane)) = -1134.819257
E(B3LYP-D3/6-311+G**(dichloromethane)) = -1135.120907
Gtot(B3LYP/6-31G*(dichloromethane)) = -1134.432203

C	-0.729642	-0.973301	7.355740
C	-1.901900	-0.486658	6.687069
C	-2.164643	0.926869	6.838346
C	-1.267772	1.719621	7.576061
C	-0.125225	1.213526	8.194626
C	0.129689	-0.160830	8.081489
H	-0.532492	-2.039926	7.258829
O	-2.622432	-1.297314	5.975183
H	-1.485704	2.783427	7.685453
H	0.532900	1.867307	8.762444
H	1.004133	-0.595344	8.565850
C	-3.411723	1.660681	6.330247
H	-3.269376	2.665198	6.742957
C	-3.433604	2.112887	4.796598
C	-4.574021	3.139460	4.655289
O	-5.682418	2.789372	4.265730
H	-2.475304	2.610501	4.646676
C	-4.068889	7.204550	5.961208
C	-5.337796	6.680679	5.688226
C	-5.467259	5.364273	5.258239
C	-4.331873	4.545807	5.097961
C	-3.060866	5.083109	5.373019

C -2.933737 6.405251 5.799994
H -3.966026 8.233299 6.299685
H -6.223855 7.298554 5.814719
H -6.447147 4.945144 5.050819
H -2.158229 4.491358 5.253375
H -1.946943 6.811790 6.007251
N -3.458461 1.088939 3.635903
C -4.500805 0.009115 3.825458
H -4.533406 -0.583852 2.907779
H -4.168746 -0.601581 4.664707
H -5.459404 0.486148 4.012379
C -3.700723 1.810656 2.323459
H -3.505670 1.105885 1.513425
H -4.731123 2.151931 2.273081
H -3.008371 2.654102 2.252397
C -2.099788 0.424672 3.518395
H -2.105017 -0.174640 2.604862
H -1.330932 1.197470 3.449430
H -1.964891 -0.226439 4.378790
C -7.159014 0.623931 8.310267
C -6.768425 1.953316 8.138899
C -5.574975 2.248651 7.479390
C -4.748864 1.236112 6.959880
C -5.146304 -0.094376 7.153954
C -6.337505 -0.393836 7.820322
H -8.082850 0.384342 8.833248
H -7.381799 2.762107 8.531377
H -5.276377 3.290237 7.386063
H -4.498012 -0.891408 6.806895
H -6.617644 -1.435319 7.966928

***Transoid trans*-A**

E(B3LYP-D3/6-31G*(dichloromethane)) = -1134.826741
E(B3LYP-D3/6-311+G**(dichloromethane)) = -1135.133223
Gtot(B3LYP/6-31G*(dichloromethane)) = -1134.438629

C -5.763899 -0.378264 6.789704
C -5.141202 0.852197 6.409691
C -3.726844 0.939396 6.703043
C -3.055510 -0.127159 7.309171
C -3.703253 -1.310016 7.668259
C -5.074266 -1.419443 7.398077
H -6.826311 -0.472022 6.569427
O -5.818949 1.791733 5.817000
H -1.988162 -0.022828 7.509532
H -3.155388 -2.122243 8.140795
H -5.607257 -2.332603 7.664561
C -2.925694 2.212002 6.397590
H -1.862802 1.961894 6.482645
C -3.152801 2.524527 4.876286
C -2.309841 1.462957 4.125309
O -1.087679 1.612428 4.090689
H -4.222937 2.437624 4.690194

C	-3.983612	-2.085066	2.406549
C	-2.597006	-1.908807	2.494120
C	-2.083520	-0.741748	3.045387
C	-2.946131	0.266288	3.523015
C	-4.336928	0.082248	3.430171
C	-4.846174	-1.090185	2.872153
H	-4.388295	-3.001460	1.982164
H	-1.922527	-2.684718	2.139144
H	-1.010843	-0.596084	3.128815
H	-5.028651	0.819721	3.823907
H	-5.922318	-1.232339	2.814678
N	-2.799720	3.948040	4.342341
C	-1.490308	4.493942	4.862457
H	-1.300423	5.450751	4.370111
H	-1.577816	4.640838	5.936921
H	-0.704370	3.777230	4.637242
C	-2.747842	3.911211	2.829665
H	-2.707485	4.938427	2.460460
H	-1.859178	3.370225	2.509083
H	-3.651388	3.418657	2.462881
C	-3.913431	4.899945	4.720473
H	-3.677535	5.884858	4.310367
H	-4.852211	4.529469	4.304203
H	-3.983940	4.950229	5.803291
C	-3.539408	4.972417	9.698195
C	-2.261250	4.523631	9.363695
C	-2.084620	3.674460	8.267534
C	-3.174113	3.259054	7.485532
C	-4.457519	3.708540	7.839239
C	-4.633921	4.559570	8.930682
H	-3.684877	5.626954	10.554965
H	-1.400526	4.821577	9.959351
H	-1.085441	3.311276	8.031154
H	-5.305563	3.358862	7.257803
H	-5.636556	4.891435	9.192938

TS rotation *trans*-A

E(B3LYP-D3/6-31G*(dichloromethane)) = -1134.815779
E(B3LYP-D3/6-311+G**(dichloromethane)) = -1135.120329
Gtot(B3LYP/6-31G*(dichloromethane)) = -1134.426275

C	-0.668079	-0.940915	7.060191
C	-1.903783	-0.431044	6.535333
C	-2.190831	0.950155	6.862756
C	-1.298320	1.673740	7.670493
C	-0.116209	1.134946	8.175281
C	0.191586	-0.193469	7.851655
H	-0.436946	-1.977406	6.818634
O	-2.674109	-1.200535	5.838712
H	-1.540797	2.709268	7.911090
H	0.543661	1.734791	8.797593
H	1.109511	-0.646338	8.227355
C	-3.432293	1.714016	6.399390

H	-3.273011	2.700034	6.845929
C	-3.389273	2.103274	4.830742
C	-3.449326	3.642551	4.831437
O	-4.527156	4.220199	4.705597
H	-2.432300	1.753814	4.447659
C	0.040688	5.990746	5.691425
C	-1.232562	6.572462	5.653328
C	-2.343082	5.788293	5.364038
C	-2.201326	4.408907	5.110888
C	-0.918555	3.834033	5.150992
C	0.194007	4.625508	5.438929
H	0.910622	6.602328	5.921032
H	-1.355660	7.634310	5.854087
H	-3.336311	6.226229	5.337608
H	-0.770752	2.773540	4.979871
H	1.181195	4.172279	5.473242
N	-4.392421	1.520818	3.802255
C	-5.856837	1.589979	4.182767
H	-6.433485	1.357712	3.284446
H	-6.056266	0.851953	4.953510
H	-6.080882	2.592324	4.534333
C	-4.218346	2.247595	2.480336
H	-4.711454	1.654290	1.707674
H	-4.671970	3.234561	2.541192
H	-3.151036	2.326083	2.258638
C	-4.026419	0.070477	3.566130
H	-4.833165	-0.393289	2.994421
H	-3.098849	0.042711	2.988481
H	-3.846798	-0.422916	4.527828
C	-7.186896	0.689446	8.322675
C	-6.825449	2.022310	8.110508
C	-5.623193	2.323366	7.469560
C	-4.766657	1.304987	7.018410
C	-5.133310	-0.029343	7.241389
C	-6.335083	-0.330119	7.888700
H	-8.117297	0.448776	8.833003
H	-7.470857	2.827652	8.455635
H	-5.344786	3.365233	7.321276
H	-4.466831	-0.816931	6.902214
H	-6.601745	-1.370517	8.063861

***Cis*-A**

E(B3LYP-D3/6-31G*(dichloromethane)) = -1134.832488
E(B3LYP-D3/6-311+G**(dichloromethane)) = -1135.137778
Gtot(B3LYP/6-31G*(dichloromethane)) = -1134.444481

C	-1.073518	5.243341	6.104739
C	-2.334507	4.595269	5.916676
C	-2.370680	3.187780	6.256442
C	-1.230303	2.542454	6.738786
C	-0.015879	3.211681	6.904746
C	0.047909	4.572714	6.578650

H -1.020893 6.300248 5.847442
O -3.360865 5.227053 5.434024
H -1.285329 1.487865 6.995472
H 0.857535 2.682334 7.279017
H 0.985886 5.115532 6.700120
C -3.716101 2.482617 6.084810
H -4.418904 3.156264 6.580782
C -4.169402 2.545077 4.564200
H -3.857113 3.550170 4.263666
C -3.529051 1.492810 3.657524
O -4.162464 0.527422 3.227961
N -5.716042 2.622524 4.377442
C -6.265091 3.783233 5.193273
H -7.234712 4.058935 4.774461
H -6.401729 3.462982 6.225910
H -5.553192 4.609182 5.151556
C -6.471580 1.375609 4.782791
H -7.538965 1.587049 4.681996
H -6.170417 0.554909 4.139166
H -6.232295 1.146895 5.819712
C -6.003890 2.931117 2.922085
H -7.084574 3.031569 2.799923
H -5.511032 3.871596 2.662907
H -5.637796 2.117779 2.299418
C 0.669329 1.720070 2.815090
C -0.003261 0.491803 2.803958
C -1.370054 0.450814 3.056070
C -2.079499 1.632624 3.348861
C -1.400852 2.862039 3.335584
C -0.032170 2.900471 3.067953
H 1.740039 1.753822 2.623995
H 0.540531 -0.428479 2.602811
H -1.903468 -0.495877 3.050086
H -1.926038 3.790619 3.523989
H 0.487036 3.854673 3.069595
C -4.036732 -1.291614 8.223527
C -4.662418 -0.135654 8.696094
C -4.558226 1.059807 7.980833
C -3.831942 1.132510 6.781493
C -3.197226 -0.035112 6.329898
C -3.300876 -1.233974 7.038012
H -4.114082 -2.225083 8.777191
H -5.229313 -0.162116 9.624389
H -5.041053 1.956410 8.367131
H -2.586030 -0.013916 5.436679
H -2.794669 -2.122289 6.664986

TS proton transfer in *cis*-A

E(B3LYP-D3/6-31G*(dichloromethane)) = -1134.81879
E(B3LYP-D3/6-311+G**(dichloromethane)) = -1135.120907
Gtot(B3LYP/6-31G*(dichloromethane)) = -1134.434101

C -7.371799 0.740237 5.63117

C	-6.076479	1.220131	5.34846
C	-5.135913	1.337639	6.41519
C	-5.523921	0.902984	7.68944
C	-6.805234	0.417884	7.95674
C	-7.734972	0.346910	6.91573
H	-8.074784	0.673049	4.80373
H	-4.799029	0.931922	8.49842
H	-7.067366	0.097059	8.96255
H	-8.740950	-0.025352	7.10255
C	-3.679442	1.730733	6.13492
H	-3.185022	0.768145	5.97849
C	-3.452615	2.416837	4.73870
C	-2.289310	1.946073	3.97820
O	-1.532305	2.667971	3.29950
C	-1.383891	-2.274382	3.90980
C	-0.365913	-1.318851	3.98970
C	-0.682964	0.039190	4.00237
C	-2.022248	0.458467	3.96467
C	-3.039140	-0.505827	3.87134
C	-2.718150	-1.864237	3.83920
H	-1.137643	-3.334064	3.89887
H	0.674377	-1.632948	4.04007
H	0.103460	0.788205	4.04775
H	-4.078406	-0.193972	3.81875
H	-3.512720	-2.603172	3.76268
N	-3.680666	3.945340	4.63477
C	-2.515655	4.775108	5.13713
H	-2.744863	5.830349	4.96616
H	-2.388504	4.586673	6.20044
H	-1.627033	4.481855	4.58420
C	-3.949127	4.302341	3.18382
H	-4.175460	5.370001	3.13177
H	-3.064838	4.071301	2.59751
H	-4.805937	3.717690	2.84561
C	-4.916022	4.361444	5.39551
H	-5.062124	5.433478	5.24443
H	-5.766447	3.804656	5.00806
H	-4.776534	4.160553	6.45209
C	-1.242131	3.467412	9.30723
C	-0.685265	2.650991	8.31816
C	-1.498364	2.101388	7.32813
C	-2.880752	2.351047	7.29214
C	-3.427009	3.157375	8.30124
C	-2.615982	3.713832	9.29581
H	-0.612612	3.896549	10.08369
H	0.381543	2.436660	8.32172
H	-1.054448	1.463676	6.56645
H	-4.497678	3.337851	8.33542
H	-3.065122	4.335005	10.06796
O	-5.768064	1.545537	4.08157
H	-4.593375	1.929186	4.17752

B (from *cis*-A)

E(B3LYP-D3/6-31G*(dichloromethane)) = -1134.82399

E(B3LYP-D3/6-311+G**(dichloromethane)) = -1135.128090
Gtot(B3LYP/6-31G*(dichloromethane)) = -1134.435462

C	-7.394214	0.731241	5.653845
C	-6.114562	1.226726	5.369071
C	-5.137429	1.347790	6.385599
C	-5.491861	0.877501	7.661115
C	-6.760009	0.374384	7.950686
C	-7.722130	0.312127	6.939826
H	-8.111692	0.670041	4.838832
H	-4.748152	0.896706	8.452055
H	-6.988755	0.029059	8.956532
H	-8.717817	-0.074706	7.147106
C	-3.682111	1.760414	6.090599
H	-3.191911	0.796145	5.934321
C	-3.378939	2.467006	4.745222
C	-2.339509	1.966728	3.908434
O	-1.645462	2.641028	3.092387
C	-1.350544	-2.248823	4.008974
C	-0.354624	-1.268786	4.072353
C	-0.698675	0.082882	4.030709
C	-2.042998	0.478150	3.955021
C	-3.033742	-0.512765	3.873906
C	-2.689682	-1.866441	3.898348
H	-1.083755	-3.303231	4.040169
H	0.690836	-1.559452	4.152798
H	0.072793	0.848312	4.062487
H	-4.078162	-0.227650	3.788945
H	-3.469949	-2.621972	3.833161
N	-3.638095	3.976877	4.617396
C	-2.431742	4.791304	5.043170
H	-2.650766	5.853406	4.901654
H	-2.234531	4.581632	6.092992
H	-1.592425	4.482625	4.422942
C	-3.983002	4.324751	3.179455
H	-4.144701	5.403945	3.116461
H	-3.156093	4.017908	2.545964
H	-4.897979	3.793769	2.912976
C	-4.822159	4.419483	5.436385
H	-4.987632	5.483221	5.249705
H	-5.695673	3.847673	5.130445
H	-4.614666	4.267348	6.489417
C	-1.284710	3.388191	9.360718
C	-0.719268	2.597106	8.355368
C	-1.519230	2.089108	7.333052
C	-2.898435	2.351491	7.282643
C	-3.452342	3.135218	8.304674
C	-2.654346	3.653146	9.330386
H	-0.664861	3.784193	10.162240
H	0.344813	2.369866	8.371823
H	-1.069948	1.476287	6.554565
H	-4.521622	3.325189	8.327909
H	-3.112228	4.256004	10.112042
O	-5.871116	1.570914	4.065378
H	-4.900182	1.845441	4.028661

TS rotation B

E(B3LYP-D3/6-31G*(dichloromethane)) = -1134.79831
E(B3LYP-D3/6-311+G**(dichloromethane)) = -1135.1064475
Gtot(B3LYP/6-31G*(dichloromethane)) = -1134.409009

C	-6.694940	-0.332999	7.364158
C	-5.606614	0.098260	6.597271
C	-5.374171	1.470038	6.379591
C	-6.288342	2.375760	6.934568
C	-7.371233	1.956715	7.704360
C	-7.570134	0.591068	7.929814
H	-6.837002	-1.402688	7.499459
H	-6.150070	3.434567	6.733656
H	-8.058095	2.690769	8.119446
H	-8.410499	0.244857	8.527728
C	-4.080226	1.952088	5.686840
H	-3.676390	1.104630	5.116638
C	-4.318404	3.123477	4.704707
C	-5.479029	3.219629	3.925729
O	-5.908806	4.251256	3.295529
C	-7.825972	-0.362933	3.312622
C	-6.456149	-0.371174	3.040943
C	-5.698937	0.784664	3.247606
C	-6.295564	1.958099	3.730558
C	-7.679369	1.970037	3.946437
C	-8.436720	0.815278	3.756926
H	-8.417254	-1.265552	3.172769
H	-5.977355	-1.276437	2.672702
H	-4.632358	0.782509	3.033010
H	-8.149161	2.885314	4.296798
H	-9.505259	0.828520	3.962172
N	-3.303558	4.250893	4.488842
C	-3.093302	4.458377	2.995019
H	-2.419304	5.307168	2.853971
H	-2.645339	3.545153	2.600082
H	-4.059735	4.648556	2.537069
C	-3.814901	5.537660	5.100238
H	-3.104148	6.346573	4.902003
H	-4.783345	5.743395	4.646111
H	-3.915402	5.380095	6.174656
C	-1.916271	3.992843	5.022467
H	-1.257290	4.760938	4.611802
H	-1.909037	4.050899	6.104910
H	-1.585098	3.007043	4.694644
C	-1.316426	2.226853	9.049540
C	-1.118237	1.291663	8.033364
C	-2.000510	1.234948	6.950046
C	-3.093050	2.105409	6.859372
C	-3.295185	3.024708	7.901391
C	-2.412249	3.094063	8.978012
H	-0.633160	2.275541	9.894550
H	-0.278424	0.601316	8.080919

H	-1.833764	0.501807	6.162722
H	-4.154544	3.687503	7.877657
H	-2.586639	3.818799	9.770751
O	-4.785433	-0.898177	6.109435
H	-4.361817	-0.607723	5.283879

B (from *trans*-A)

E(B3LYP-D3/6-31G*(dichloromethane)) = -1134.821801
E(B3LYP-D3/6-311+G**(dichloromethane)) = -1135.12720
Gtot(B3LYP/6-31G*(dichloromethane)) = -1134.434253

C	-7.468530	0.485220	6.15631
C	-6.311123	1.143904	5.71158
C	-5.080818	0.964828	6.37912
C	-5.063058	0.076602	7.46709
C	-6.204220	-0.585947	7.91162
C	-7.419896	-0.369188	7.25267
H	-8.396799	0.662631	5.61802
H	-4.119902	-0.078890	7.98841
H	-6.148573	-1.258639	8.76462
H	-8.325598	-0.869357	7.58985
C	-3.763333	1.658500	5.99185
H	-3.039023	0.839972	5.88365
C	-3.746398	2.409310	4.63335
C	-3.469739	3.800451	4.49729
O	-2.839500	4.343915	3.54203
C	-4.933254	6.616064	7.43346
C	-5.814486	5.683331	6.88564
C	-5.359176	4.749121	5.95134
C	-4.018691	4.746737	5.54352
C	-3.149916	5.713922	6.07030
C	-3.596689	6.629165	7.02053
H	-5.284383	7.332636	8.17351
H	-6.859248	5.675266	7.19037
H	-6.055989	4.026361	5.54156
H	-2.114362	5.724415	5.74221
H	-2.904003	7.354806	7.44187
N	-3.259167	1.533814	3.45527
C	-3.834441	2.050856	2.14904
H	-3.469586	1.417052	1.33677
H	-4.922003	1.988796	2.21341
H	-3.512791	3.079231	2.01685
C	-1.749473	1.524866	3.36950
H	-1.435965	0.918483	2.51474
H	-1.422351	2.557252	3.25596
H	-1.357394	1.091790	4.29186
C	-3.742095	0.105384	3.56126
H	-3.458789	-0.413981	2.64337
H	-3.280168	-0.396830	4.40930
H	-4.826036	0.111019	3.66881
C	-1.937687	3.855603	9.28613
C	-1.162756	3.345449	8.24102
C	-1.771517	2.652083	7.19520

C	-3.159055	2.455305	7.16581
C	-3.922095	2.960526	8.22312
C	-3.319245	3.660303	9.27040
H	-1.468061	4.397453	10.10460
H	-0.083285	3.483765	8.24268
H	-1.158980	2.264594	6.38302
H	-4.997663	2.820595	8.22341
H	-3.935628	4.055717	10.07505
O	-6.440920	1.931520	4.59998
H	-5.517938	2.314870	4.43157

TS proton transfer in *trans*-A

E(B3LYP-D3/6-31G*(dichloromethane)) = -1134.818765
E(B3LYP-D3/6-311+G**(dichloromethane)) = -1135.120876
Gtot(B3LYP/6-31G*(dichloromethane)) = -1134.434053

C	-7.334784	0.654675	5.684041
C	-6.056987	1.168600	5.380126
C	-5.099487	1.301272	6.429743
C	-5.450873	0.845198	7.707014
C	-6.714360	0.326644	7.995183
C	-7.662646	0.241240	6.971883
H	-8.051789	0.576332	4.869606
O	-5.779975	1.511299	4.111277
H	-4.711154	0.885644	8.501992
H	-6.948393	-0.009985	9.002830
H	-8.654989	-0.158324	7.175061
C	-3.656494	1.726072	6.126763
H	-3.143364	0.774505	5.964369
C	-3.462447	2.414453	4.727239
C	-2.307656	1.959711	3.943719
O	-1.577725	2.691405	3.246377
H	-4.603086	1.913901	4.190141
C	-1.334410	-2.245641	3.882008
C	-0.330814	-1.273140	3.935973
C	-0.669736	0.079844	3.945438
C	-2.016109	0.476963	3.929889
C	-3.018808	-0.504320	3.861942
C	-2.676282	-1.857531	3.833211
H	-1.071064	-3.301313	3.874447
H	0.715262	-1.570111	3.969143
H	0.105214	0.841745	3.971988
H	-4.063680	-0.209835	3.824116
H	-3.459724	-2.610048	3.776565
N	-3.717915	3.938386	4.624302
C	-2.554892	4.786404	5.098878
H	-2.805268	5.837951	4.934828
H	-2.399284	4.598844	6.158701
H	-1.675147	4.508068	4.524366
C	-4.023000	4.286581	3.177863
H	-4.268345	5.350138	3.127527
H	-3.148045	4.067900	2.573023
H	-4.877407	3.687160	2.860027

C	-4.943158	4.337186	5.410175
H	-5.110700	5.405834	5.257038
H	-5.791982	3.763826	5.044074
H	-4.776551	4.144786	6.464337
C	-1.213533	3.518095	9.263100
C	-0.651933	2.716716	8.264164
C	-1.466671	2.147635	7.286331
C	-2.854840	2.363759	7.272171
C	-3.405502	3.153682	8.291436
C	-2.593411	3.729561	9.273935
H	-0.582602	3.962866	10.029715
H	0.419962	2.529413	8.250613
H	-1.019468	1.522149	6.516433
H	-4.479837	3.306247	8.342144
H	-3.046162	4.338129	10.054029

***Trans* elimination TS**

E(B3LYP-D3/6-31G*(dichloromethane)) = -1134.813656
E(B3LYP-D3/6-311+G**(dichloromethane)) = -1135.113974
Gtot(B3LYP/6-31G*(dichloromethane)) = -1134.427576

C	0.051899	3.089592	8.481111
C	-1.224106	3.185397	7.877235
C	-1.700407	2.065356	7.153993
C	-0.945770	0.904862	7.034026
C	0.319043	0.823732	7.631199
C	0.806590	1.923488	8.348069
H	0.423534	3.939397	9.049544
O	-2.000790	4.239479	7.969069
H	-1.338425	0.060078	6.468322
H	0.911388	-0.084090	7.542256
H	1.787602	1.867336	8.818466
C	-3.051991	2.265642	6.463574
H	-3.573762	1.309810	6.532920
C	-3.885073	3.320935	7.193460
C	-4.016039	3.161422	8.684730
O	-3.949232	2.017685	9.143359
H	-3.976115	4.302533	6.758094
C	-5.015360	6.415510	11.313794
C	-5.078734	5.088618	11.747932
C	-4.737024	4.054598	10.877811
C	-4.326742	4.330907	9.564435
C	-4.257172	5.668076	9.138357
C	-4.601791	6.700971	10.007927
H	-5.280498	7.224677	11.990817
H	-5.394301	4.859688	12.763500
H	-4.783762	3.019429	11.202970
H	-3.895610	5.907873	8.144775
H	-4.534854	7.732974	9.671277
N	-5.779013	2.903559	6.646272
C	-6.033904	1.445874	6.593376
H	-7.104556	1.259825	6.438894
H	-5.481716	1.001475	5.764082

H	-5.712705	0.996857	7.536081
C	-6.707927	3.529907	7.621469
H	-7.739969	3.439143	7.259361
H	-6.624721	3.028506	8.586851
H	-6.457672	4.585451	7.739145
C	-5.981430	3.531110	5.316154
H	-7.015857	3.374229	4.983626
H	-5.791502	4.605876	5.388306
H	-5.294205	3.092034	4.594425
C	-2.259738	3.201694	2.305438
C	-2.778104	1.953910	2.656273
C	-3.050921	1.658823	3.996139
C	-2.812513	2.606915	4.998049
C	-2.277937	3.853715	4.638091
C	-2.007294	4.149789	3.303119
H	-2.046177	3.433080	1.264208
H	-2.969964	1.206502	1.889210
H	-3.447485	0.679710	4.261600
H	-2.054991	4.577709	5.417928
H	-1.590708	5.119999	3.039983

***Cis* elimination TS**

E(B3LYP-D3/6-31G*(dichloromethane)) = -1134.805131
E(B3LYP-D3/6-311+G**(dichloromethane)) = -1135.104716
Gtot(B3LYP/6-31G*(dichloromethane)) = -1134.421755

C	-0.517448	4.414857	6.374076
C	-1.861888	4.054087	6.121868
C	-2.270711	2.741527	6.463174
C	-1.392748	1.828333	7.033806
C	-0.065165	2.199243	7.287571
C	0.357580	3.491199	6.949606
H	-0.186810	5.418985	6.117314
O	-2.756207	4.864823	5.602831
H	-1.738064	0.823583	7.276437
H	0.626155	1.493057	7.741472
H	1.387979	3.786899	7.143860
C	-3.711900	2.436997	6.057945
H	-4.090043	1.697809	6.769284
C	-4.500592	3.728111	6.269740
H	-4.291823	4.223521	7.206611
C	-5.201353	4.527973	5.204756
O	-5.722119	3.951293	4.253354
N	-6.272137	3.023189	7.173577
C	-5.872550	2.545259	8.514585
H	-6.755042	2.212411	9.077706
H	-5.181740	1.702848	8.428277
H	-5.386491	3.355911	9.065946
C	-6.894829	1.941696	6.381135
H	-7.838843	1.624565	6.845917
H	-7.078911	2.303609	5.368599
H	-6.221961	1.085172	6.333227
C	-7.215430	4.156320	7.317009

H	-8.080064	3.854818	7.923743
H	-6.709779	4.994594	7.802541
H	-7.567291	4.470474	6.333049
C	-5.781133	8.777237	5.591611
C	-6.366147	8.056298	4.545546
C	-6.151890	6.683759	4.441674
C	-5.346646	6.010233	5.375510
C	-4.755674	6.745438	6.417451
C	-4.977042	8.117622	6.525583
H	-5.947108	9.849214	5.675447
H	-6.988015	8.565121	3.812460
H	-6.604122	6.113750	3.635595
H	-4.091037	6.262937	7.122978
H	-4.509464	8.675288	7.333900
C	-3.602630	0.459958	2.194286
C	-4.058040	-0.217404	3.325004
C	-4.121643	0.442306	4.557202
C	-3.741987	1.783134	4.676678
C	-3.269440	2.451046	3.536051
C	-3.205283	1.797477	2.308100
H	-3.548003	-0.048562	1.233937
H	-4.358664	-1.260957	3.255232
H	-4.456918	-0.106768	5.436051
H	-2.952412	3.484852	3.622563
H	-2.838440	2.332648	1.434381

***Trans* 2,3-dihydroquinone**

E(B3LYP-D3/6-31G*(dichloromethane)) = -960.364263
E(B3LYP-D3/6-311+G**(dichloromethane)) = -960.611433
Gtot(B3LYP/6-31G*(dichloromethane)) = -960.098006

C	-0.693038	0.508840	5.811992
C	-1.292567	1.532737	6.544227
C	-2.552588	1.342766	7.100195
C	-3.197512	0.113469	6.903252
C	-2.581075	-0.899452	6.158298
C	-1.309839	-0.718305	5.596571
H	-3.023116	2.132161	7.682362
H	-4.181551	-0.055807	7.333875
H	-3.092705	-1.848907	6.016163
H	-0.817744	-1.499916	5.024081
C	-0.375432	2.741883	6.549121
H	-0.771817	3.498412	5.859471
C	0.928055	2.106836	5.932206
H	1.649436	1.890632	6.727105
O	0.547080	0.848330	5.321289
C	0.293894	4.632207	10.382207
C	0.433953	3.246315	10.248774
C	0.213398	2.636258	9.014528
C	-0.148179	3.400663	7.894776
C	-0.285782	4.785691	8.036968
C	-0.067046	5.400198	9.273674
H	0.463716	5.108116	11.345629

H	0.711840	2.640830	11.108911
H	0.312657	1.556678	8.919097
H	-0.568814	5.389253	7.176222
H	-0.180225	6.478128	9.368657
C	1.628176	3.042673	4.944623
O	2.619915	3.642901	5.348448
C	0.169574	3.841571	0.979260
C	1.403385	4.342205	1.411195
C	1.855756	4.063041	2.697697
C	1.088966	3.270191	3.571348
C	-0.148938	2.770638	3.127649
C	-0.604925	3.060075	1.841379
H	-0.186712	4.061102	-0.024728
H	2.008674	4.951256	0.743858
H	2.806947	4.454050	3.047062
H	-0.763901	2.157079	3.772751
H	-1.565518	2.672159	1.510808

***Cis* 2,3-dihydroquinone**

E(B3LYP-D3/6-31G*(dichloromethane)) = -960.364109
E(B3LYP-D3/6-311+G**(dichloromethane)) = -960.61051
Gtot(B3LYP/6-31G*(dichloromethane)) = -960.097744

C	-0.863780	0.315585	5.803133
C	-1.271546	1.447430	6.505811
C	-2.502296	1.460423	7.149536
C	-3.319523	0.323410	7.067316
C	-2.896394	-0.799487	6.346943
C	-1.652819	-0.823435	5.699361
H	-2.819453	2.335431	7.712220
H	-4.284556	0.313890	7.568287
H	-3.538373	-1.676036	6.292688
H	-1.309260	-1.694497	5.148039
C	-0.208930	2.520071	6.365614
H	-0.519936	3.233928	5.591386
C	0.973388	1.661719	5.748561
O	0.366828	0.464174	5.214160
C	0.770410	4.764452	9.937068
C	0.522488	3.391630	10.020710
C	0.187849	2.668295	8.875990
C	0.104140	3.303179	7.628296
C	0.343592	4.680703	7.557000
C	0.676408	5.408433	8.701703
H	1.030752	5.328862	10.829979
H	0.588747	2.881812	10.979581
H	0.001431	1.600557	8.947664
H	0.275054	5.187240	6.595732
H	0.861754	6.477980	8.628560
H	1.440705	2.175795	4.907230
C	2.028053	1.252324	6.794119
O	1.923436	0.166413	7.352441
C	5.200393	3.926663	7.900910
C	4.992918	2.728741	8.594759

C	3.964499	1.874478	8.211411
C	3.126462	2.202535	7.130965
C	3.343242	3.406885	6.440520
C	4.375881	4.262306	6.824592
H	6.003502	4.596847	8.200304
H	5.632540	2.466491	9.434524
H	3.786367	0.945277	8.744458
H	2.711765	3.694993	5.606308
H	4.535436	5.192675	6.284740

Trans cyclization TS toward cyclopropane

E(B3LYP-D3/6-31G*(dichloromethane)) = -1134.794688
E(B3LYP-D3/6-311+G**(dichloromethane)) = -1135.0981663
Gtot(B3LYP/6-31G*(dichloromethane)) = -1134.411163

C	-6.159517	0.653559	6.685179
C	-5.226549	1.744488	6.479143
C	-3.810624	1.362143	6.527342
C	-3.444700	-0.000918	6.690013
C	-4.381731	-0.994194	6.868505
C	-5.758914	-0.647111	6.866133
H	-7.213944	0.920697	6.671225
O	-5.651778	2.920218	6.260774
H	-2.387041	-0.255579	6.705581
H	-4.071109	-2.024164	7.024922
H	-6.505496	-1.427715	7.007082
C	-2.668800	2.393152	6.448508
H	-1.743923	1.825259	6.537915
C	-2.965805	2.688505	5.014042
C	-2.743228	1.670274	3.932460
O	-3.485231	1.712295	2.948695
H	-3.882007	3.243956	4.854968
C	0.326586	-1.339458	4.015068
C	-0.904465	-1.580627	3.394398
C	-1.885205	-0.591590	3.378699
C	-1.656686	0.645312	4.004544
C	-0.418238	0.878097	4.623291
C	0.572090	-0.104760	4.621231
H	1.093512	-2.110877	4.022360
H	-1.096545	-2.540696	2.921104
H	-2.841561	-0.767766	2.894482
H	-0.222977	1.833485	5.095153
H	1.532466	0.092778	5.091988
N	-1.873893	4.309245	4.187495
C	-0.741672	4.686988	5.055851
H	-0.265093	5.604753	4.680347
H	-1.095657	4.855816	6.071681
H	0.006991	3.891114	5.060491
C	-1.396865	4.000385	2.824565
H	-0.913702	4.884399	2.383532
H	-0.673241	3.182214	2.864176
H	-2.238731	3.703458	2.197691
C	-2.889029	5.380927	4.148668

H	-2.462792	6.299574	3.720097
H	-3.734143	5.061584	3.532269
H	-3.237369	5.587837	5.161716
C	-2.357056	5.393218	9.559319
C	-1.438881	4.345104	9.463764
C	-1.567141	3.398333	8.445902
C	-2.609898	3.475650	7.509026
C	-3.530977	4.527475	7.617812
C	-3.400705	5.477445	8.633736
H	-2.262420	6.134007	10.350363
H	-0.622472	4.262795	10.178593
H	-0.843954	2.586490	8.379855
H	-4.367551	4.567371	6.931293
H	-4.127206	6.284074	8.705440

***Cis* cyclization TS toward cyclopropane**

E(B3LYP-D3/6-31G*(dichloromethane)) = -1134.799895
E(B3LYP-D3/6-311+G**(dichloromethane)) = -1135.101919
Gtot(B3LYP/6-31G*(dichloromethane)) = -1134.418274

C	-6.234965	1.184578	6.696222
C	-5.050193	2.007707	6.613449
C	-3.818923	1.296728	6.231332
C	-3.834186	-0.097333	5.967094
C	-4.999839	-0.823913	6.059916
C	-6.204357	-0.163839	6.424999
H	-7.160992	1.681770	6.976579
O	-5.068674	3.256048	6.828600
H	-2.908923	-0.599787	5.700194
H	-5.000830	-1.892678	5.861829
H	-7.123759	-0.744577	6.490614
C	-2.546791	2.157194	6.185149
C	-2.949607	2.433433	4.789049
C	-2.624051	1.610714	3.569975
O	-1.463344	1.276430	3.334210
H	-3.817668	3.075105	4.712597
C	-5.667839	0.516633	0.748076
C	-4.312422	0.470871	0.399680
C	-3.345183	0.838508	1.329035
C	-3.718443	1.273059	2.615171
C	-5.080553	1.311836	2.957893
C	-6.047510	0.933080	2.026590
H	-6.424674	0.221647	0.024474
H	-4.014048	0.144355	-0.593941
H	-2.289841	0.798277	1.075230
H	-5.389626	1.598274	3.956985
H	-7.098707	0.953258	2.303671
N	-1.827230	4.267413	4.132184
C	-0.377174	4.044132	4.177345
H	0.171924	4.969201	3.939326
H	-0.096518	3.706411	5.177981
H	-0.107890	3.266051	3.461236
C	-2.294285	4.619909	2.785645
H	-1.882424	5.588419	2.460457

H	-1.983023	3.849872	2.074777
H	-3.387490	4.685630	2.783757
C	-2.260399	5.260247	5.124667
H	-1.918588	6.270097	4.849859
H	-3.352823	5.262552	5.200114
H	-1.844213	5.006721	6.102820
H	-2.773076	3.041225	6.783934
C	1.163145	0.552039	7.756974
C	0.627265	-0.007025	6.594792
C	-0.553810	0.492738	6.042914
C	-1.229675	1.567870	6.643405
C	-0.678835	2.117899	7.813524
C	0.502988	1.620488	8.366348
H	2.083908	0.158474	8.182400
H	1.130975	-0.839383	6.107502
H	-0.924584	0.047854	5.129886
H	-1.189093	2.943788	8.306470
H	0.903629	2.066938	9.274017

***Trans* addition TS onto C3**

E(B3LYP-D3/6-31G*(dichloromethane)) = -1134.772215
E(B3LYP-D3/6-311+G**(dichloromethane)) = -1135.080923
Gtot(B3LYP/6-31G*(dichloromethane)) = -1134.387157

C	0.634544	1.545473	4.181650
C	-0.745385	1.685421	4.321984
C	-1.396072	1.351653	5.527985
C	-0.595941	0.927539	6.608329
C	0.786382	0.793980	6.470678
C	1.407827	1.090219	5.254081
H	1.109675	1.796428	3.235330
H	-1.339626	2.043688	3.483463
H	-1.057736	0.729901	7.571478
H	1.382755	0.466758	7.320229
H	2.485475	0.983372	5.147561
C	-2.858966	1.486981	5.592952
H	-3.281419	2.328097	5.044215
C	-6.236765	-2.707960	5.875424
H	-5.528035	-3.475885	6.170813
N	-7.626062	-3.268943	6.176117
C	-8.053769	-4.210969	5.066628
H	-8.985229	-4.701081	5.362601
H	-8.200387	-3.631091	4.157087
H	-7.265606	-4.953389	4.923369
C	-8.683338	-2.192509	6.324144
H	-9.654741	-2.685843	6.406481
H	-8.485488	-1.617299	7.227727
H	-8.636796	-1.555370	5.443751
C	-7.557219	-4.072705	7.453729
H	-8.557581	-4.445107	7.687136
H	-6.871071	-4.911047	7.309093
H	-7.202414	-3.437108	8.266000
C	-5.802217	-1.214275	7.053865

C -6.166552 0.088386 6.566188
C -5.245142 1.034460 6.072309
C -3.772610 0.660276 6.167176
C -3.424445 -0.595137 6.822848
C -4.356647 -1.460194 7.259813
H -6.415749 -1.584640 7.879019
H -7.214802 0.375138 6.555982
O -5.571123 2.178194 5.626977
H -2.376944 -0.859510 6.918317
H -4.056385 -2.403382 7.713032
C -6.038743 -2.163994 4.529287
O -6.944278 -1.579080 3.911681
C -2.035276 -1.972860 2.983858
C -2.954517 -0.950458 2.733778
C -4.251701 -1.040941 3.231733
C -4.639923 -2.146495 4.005942
C -3.711201 -3.171871 4.249538
C -2.419000 -3.088904 3.732773
H -1.021419 -1.899458 2.597079
H -2.654708 -0.077840 2.159293
H -4.970776 -0.246927 3.055449
H -3.995566 -4.048283 4.826325
H -1.708984 -3.891919 3.916612

***Cis* addition TS onto C3**

E(B3LYP-D3/6-31G*(dichloromethane)) = -1134.776230
E(B3LYP-D3/6-311+G**(dichloromethane)) = -1135.084280
Gtot(B3LYP/6-31G*(dichloromethane)) = -1134.392764

C 0.790827 1.281490 4.155727
C -0.594378 1.386507 4.268872
C -1.251813 1.119611 5.487698
C -0.455751 0.795846 6.604572
C 0.932152 0.696017 6.493609
C 1.562042 0.927711 5.267649
H 1.271911 1.479815 3.199943
H -1.187819 1.662411 3.399755
H -0.925101 0.653162 7.573821
H 1.525613 0.446839 7.371086
H 2.643902 0.848796 5.182527
C -2.717437 1.234290 5.533029
H -3.146687 2.032103 4.928410
C -6.167025 -2.882637 5.928011
H -6.936147 -2.381230 5.350981
C -4.966508 -3.264417 5.166720
O -4.296968 -4.286900 5.426967
N -6.869194 -4.023937 6.664551
C -7.148234 -5.171866 5.709939
H -7.743490 -5.924929 6.233241
H -7.702788 -4.776882 4.855952
H -6.197487 -5.590644 5.388237
C -8.201912 -3.529256 7.177380
H -8.697166 -4.349132 7.702710

H	-8.043566	-2.694697	7.860024
H	-8.811280	-3.208887	6.328777
C	-6.059417	-4.547472	7.832191
H	-6.597264	-5.392020	8.269542
H	-5.088684	-4.857085	7.452407
H	-5.952911	-3.756114	8.574207
C	-3.366457	-0.428599	2.363198
C	-2.574069	-1.459455	2.885111
C	-3.126931	-2.379443	3.765395
C	-4.478438	-2.285585	4.153356
C	-5.267192	-1.256615	3.609994
C	-4.711066	-0.336390	2.718525
H	-2.932793	0.296971	1.678123
H	-1.523274	-1.529849	2.615144
H	-2.519083	-3.170063	4.193874
H	-6.315778	-1.159758	3.866484
H	-5.329544	0.461259	2.315673
C	-5.655128	-1.398438	7.116074
C	-6.017028	-0.134350	6.542080
C	-5.095519	0.805574	6.031037
C	-3.626485	0.444170	6.162578
C	-3.282568	-0.765857	6.904061
C	-4.215061	-1.612198	7.380440
H	-6.300391	-1.726719	7.933751
H	-7.071814	0.126698	6.474317
O	-5.430683	1.921230	5.531909
H	-2.234159	-1.008400	7.045195
H	-3.911349	-2.518543	7.898219

***Trans* addition TS onto C5**

E(B3LYP-D3/6-31G*(dichloromethane)) = -1134.773050
E(B3LYP-D3/6-311+G**(dichloromethane)) = -1135.0782364
Gtot(B3LYP/6-31G*(dichloromethane)) = -1134.387869

C	0.775831	0.939405	4.767889
C	-0.590957	1.216529	4.830146
C	-1.295566	1.100496	6.042795
C	-0.577226	0.749278	7.201750
C	0.791852	0.485075	7.141806
C	1.471573	0.567435	5.922536
H	1.300479	1.022922	3.818390
H	-1.123901	1.520345	3.930827
H	-1.094055	0.713573	8.157806
H	1.330164	0.223738	8.050482
H	2.538333	0.360248	5.876181
C	-2.741852	1.377079	6.073779
H	-3.078793	2.293952	5.589382
C	-3.306591	-1.847508	5.451343
H	-2.734669	-1.168672	4.830918
N	-2.420897	-3.077263	5.668636
C	-2.579575	-4.038087	4.502817
H	-1.833542	-4.831662	4.598609
H	-3.584970	-4.453643	4.531694

H -2.421843 -3.480847 3.577204
C -2.724463 -3.828483 6.947780
H -2.174739 -4.772572 6.923642
H -2.390735 -3.231910 7.795897
H -3.798053 -3.994468 6.987147
C -0.975663 -2.642282 5.699093
H -0.353555 -3.515880 5.908202
H -0.711071 -2.218779 4.727965
H -0.837950 -1.891271 6.475158
C -5.758880 -0.750246 7.991108
C -6.107142 0.380945 7.243668
C -5.139326 1.134032 6.525860
C -3.718189 0.594034 6.583847
C -3.475810 -0.773503 7.150849
C -4.481288 -1.300597 8.017882
H -6.532712 -1.214865 8.603765
H -7.127035 0.757365 7.246328
O -5.384016 2.209971 5.922153
H -2.448294 -0.953242 7.449081
H -4.271743 -2.169427 8.632051
C -4.678497 -2.125891 5.042779
O -5.299753 -3.091411 5.536226
C -6.787441 0.443386 2.294186
C -7.436500 -0.603235 2.960308
C -6.731568 -1.410065 3.846609
C -5.363609 -1.192052 4.091220
C -4.720100 -0.146847 3.405662
C -5.428762 0.667220 2.522411
H -7.337197 1.078999 1.603398
H -8.495507 -0.785132 2.789654
H -7.228359 -2.216408 4.376625
H -3.666549 0.056682 3.563511
H -4.916475 1.479357 2.011780

***Cis* addition TS onto C5**

E(B3LYP-D3/6-31G*(dichloromethane)) = -1134.774517
E(B3LYP-D3/6-311+G**(dichloromethane)) = -1135.078692
Gtot(B3LYP/6-31G*(dichloromethane)) = -1134.388563

C 0.217438 2.536388 4.331373
C -1.128900 2.221057 4.502684
C -1.585715 1.614599 5.688609
C -0.654500 1.379682 6.718202
C 0.691874 1.705378 6.550433
C 1.135079 2.276543 5.354201
H 0.551906 2.992275 3.401855
H -1.839188 2.422182 3.704499
H -0.996640 0.978704 7.668422
H 1.394708 1.525202 7.361223
H 2.184732 2.532488 5.225582
C -3.019210 1.317001 5.818516
H -3.692236 2.042300 5.361036
C -3.212290 -2.268418 5.488492

H	-4.272263	-2.160924	5.283876
C	-2.298277	-1.849790	4.415472
O	-1.119511	-2.261161	4.378553
N	-3.000902	-3.716935	5.943089
C	-2.897908	-4.639156	4.740826
H	-2.907164	-5.672096	5.098736
H	-3.759587	-4.456935	4.095216
H	-1.971643	-4.429281	4.211000
C	-4.206951	-4.164282	6.736210
H	-4.020199	-5.175140	7.106572
H	-4.353526	-3.472736	7.561870
H	-5.078895	-4.162293	6.077970
C	-1.757679	-3.899323	6.788176
H	-1.638519	-4.965660	6.993952
H	-0.913096	-3.513521	6.223127
H	-1.883761	-3.355103	7.720396
C	-3.486710	0.924244	1.338315
C	-2.181273	0.422364	1.404468
C	-1.828109	-0.466441	2.414531
C	-2.770456	-0.883506	3.373506
C	-4.080210	-0.378663	3.291172
C	-4.431250	0.522589	2.285360
H	-3.763341	1.626986	0.554938
H	-1.437923	0.735236	0.674259
H	-0.814050	-0.845876	2.486859
H	-4.836005	-0.645734	4.019059
H	-5.443733	0.917529	2.252525
C	-4.835012	-1.118546	8.501847
C	-5.672508	-0.388291	7.645899
C	-5.144553	0.332889	6.538340
C	-3.632854	0.264295	6.404936
C	-2.939523	-0.921949	6.982436
C	-3.493954	-1.373517	8.230746
H	-5.264831	-1.526366	9.418106
H	-6.731404	-0.272204	7.864472
O	-5.831697	1.055973	5.766763
H	-1.857011	-0.908740	6.906863
H	-2.895714	-1.967600	8.915885

4. Copies of HPLC Chromatograms

Operator:Admin Timebase:U-3000_DAD Sequence:WAS_20160621_NIK_YMC

Page 1-2
11.1.2017 10:18 AM

2 NIK-066-02 (95/5) flow 0,5

Sample Name:	**NIK-066-02 (95/5) flow 0,5**	*Injection Volume:*	**10,0**
Vial Number:	**GA6**	*Channel:*	**UV_VIS_1**
Sample Type:	**unknown**	*Wavelength:*	**220**
Control Program:	**YMC_60Min_95_5_flow0_5**	*Bandwidth:*	**4**
Quantif. Method:	**AD_H**	*Temperature/Column:*	**10**
Recording Time:	**21.6.2016 10:37**	*Flow ml/min:*	**0,500**
Run Time (min):	**60,00**	*Sample Amount:*	**1,0000**

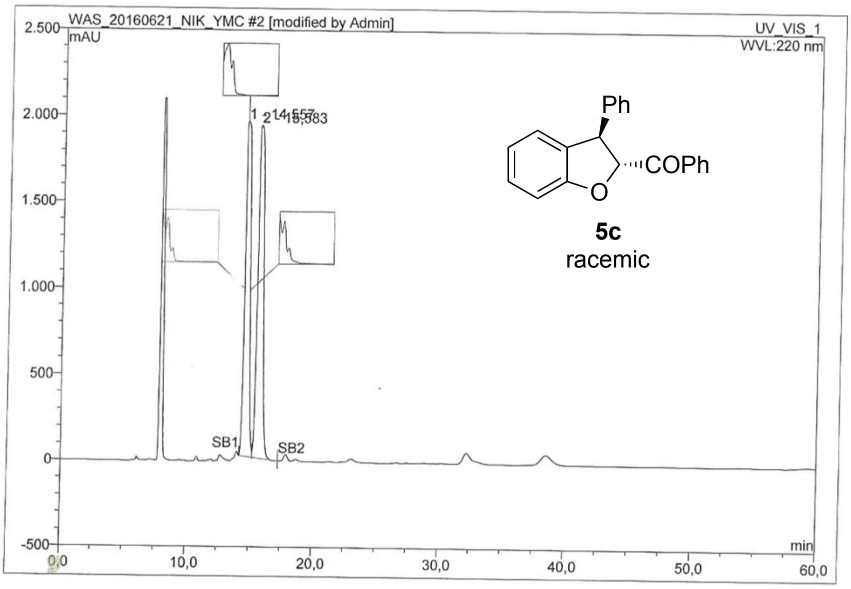

No.	Ret.Time min	Peak Name	Height mAU	Area mAU*min	Rel.Area %	Amount	Type
1	14,56	n.a.	1940,083	944,441	47,99	n.a.	BM *
2	15,58	n.a.	1930,108	1023,533	52,01	n.a.	MB*
Total:			3870,191	1967,974	100,00	0,000	

default/Integration

Chromeleon (c) Dionex 1996-2006
Version 6.80 SR12 Build 3578 (207169)

3 NIK-137-02 (95/5) flow 0,5

Sample Name:	**NIK-137-02 (95/5) flow 0,5**	*Injection Volume:*	**10,0**
Vial Number:	**GC3**	*Channel:*	**UV_VIS_2**
Sample Type:	**unknown**	*Wavelength:*	**250**
Control Program:	**YMC_60Min_95_5_flow0_5**	*Bandwidth:*	**4**
Quantif. Method:	**AD_H**	*Temperature/Column:*	**10**
Recording Time:	**26.7.2016 12:27**	*Flow ml/min:*	**0,500**
Run Time (min):	**60,00**	*Sample Amount:*	**1,0000**

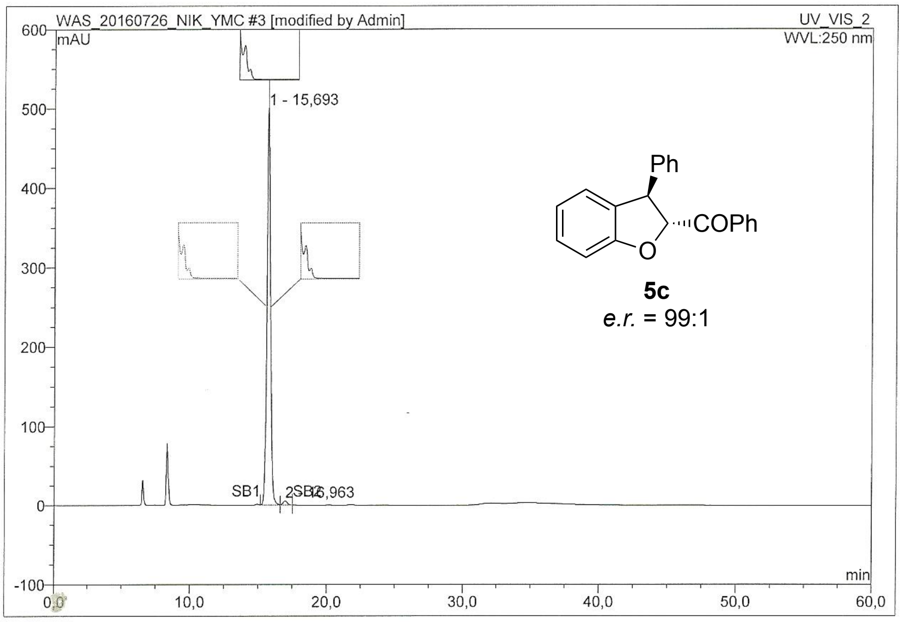

5c
e.r. = 99:1

No.	Ret.Time min	Peak Name	Height mAU	Area mAU*min	Rel.Area %	Amount	Type
1	15,69	n.a.	500,487	154,281	98,75	n.a.	BM
2	16,96	n.a.	5,479	1,952	1,25	n.a.	MB
**Total:**			505,966	156,233	100,00	0,000	

Chromeleon (c) Dionex 1996-2006
Version 6.80 SR12 Build 3578 (207169)

32 NIK-078-02

Sample Name:	**NIK-078-02**	*Injection Volume:*	**10,0**
Vial Number:	**BA3**	*Channel:*	**UV_VIS_2**
Sample Type:	**unknown**	*Wavelength:*	**290**
Control Program:	**OD_H_90Min_95_5_flow08**	*Bandwidth:*	**4**
Quantif. Method:	**default**	*Temperature/Column:*	**10**
Recording Time:	**10.8.2016 23:30**	*Flow ml/min:*	**0,800**
Run Time (min):	**90,00**	*Sample Amount:*	**1,0000**

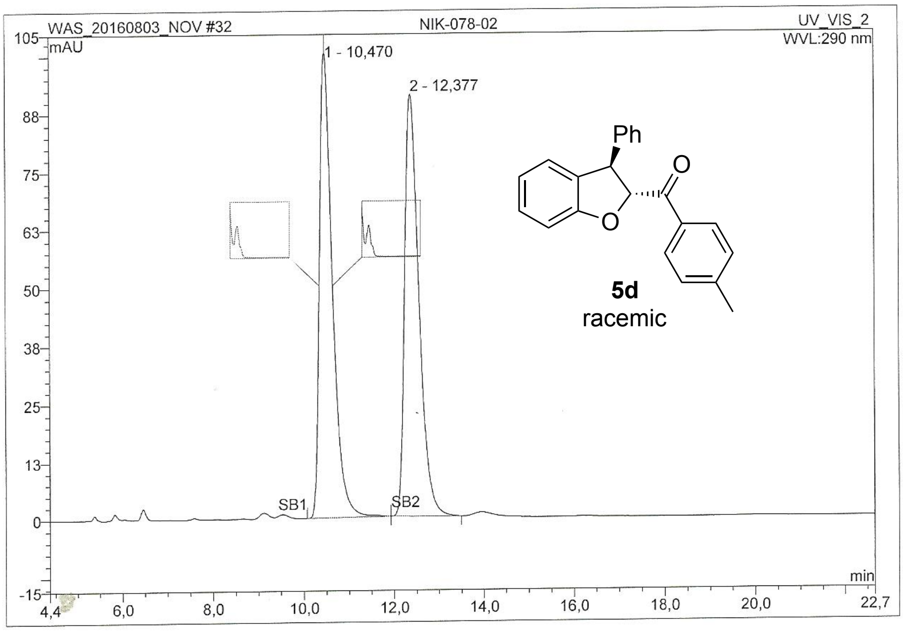

No.	Ret.Time min	Peak Name	Height mAU	Area mAU*min	Rel.Area %	Amount	Type
1	10,47	n.a.	100,261	33,302	49,81	n.a.	BMB
2	12,38	n.a.	91,017	33,556	50,19	n.a.	bMB
**Total:**			191,278	66,859	100,00	0,000	

Chromeleon (c) Dionex 1996-2006
Version 6.80 SR12 Build 3578 (207169)

9 NIK-144-02

Sample Name:	**NIK-144-02**	*Injection Volume:*	**10,0**
Vial Number:	**BA4**	*Channel:*	**UV_VIS_2**
Sample Type:	**unknown**	*Wavelength:*	**250**
Control Program:	**OD_H_45Min_95_5_flow08**	*Bandwidth:*	**4**
Quantif. Method:	**default**	*Temperature/Column:*	**10**
Recording Time:	**12.8.2016 12:03**	*Flow ml/min:*	**0,800**
Run Time (min):	**29,06**	*Sample Amount:*	**1,0000**

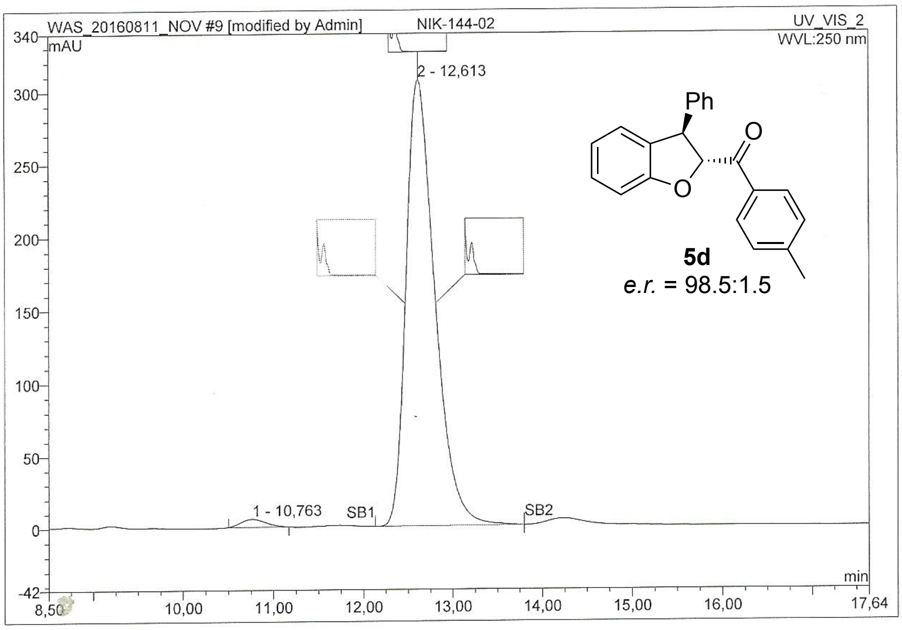

No.	Ret.Time min	Peak Name	Height mAU	Area mAU*min	Rel.Area %	Amount	Type
1	10,76	n.a.	5,451	1,745	1,49	n.a.	BMB*
2	12,61	n.a.	306,701	115,230	98,51	n.a.	BMB
**Total:**			312,152	116,975	100,00	0,000	

6 NIK-077-02 (100/1MeOH) flow 0,5

Sample Name:	**NIK-077-02 (100/1MeOH) flow 0,5**	*Injection Volume:*	**10,0**
Vial Number:	**GD1**	*Channel:*	**UV_VIS_1**
Sample Type:	**unknown**	*Wavelength:*	**220**
Control Program:	**OD_H_90Min_95_5_flow05**	*Bandwidth:*	**4**
Quantif. Method:	**default**	*Temperature/Column:*	**10**
Recording Time:	**17.8.2016 15:10**	*Flow ml/min:*	**0,500**
Run Time (min):	**66,33**	*Sample Amount:*	**1,0000**

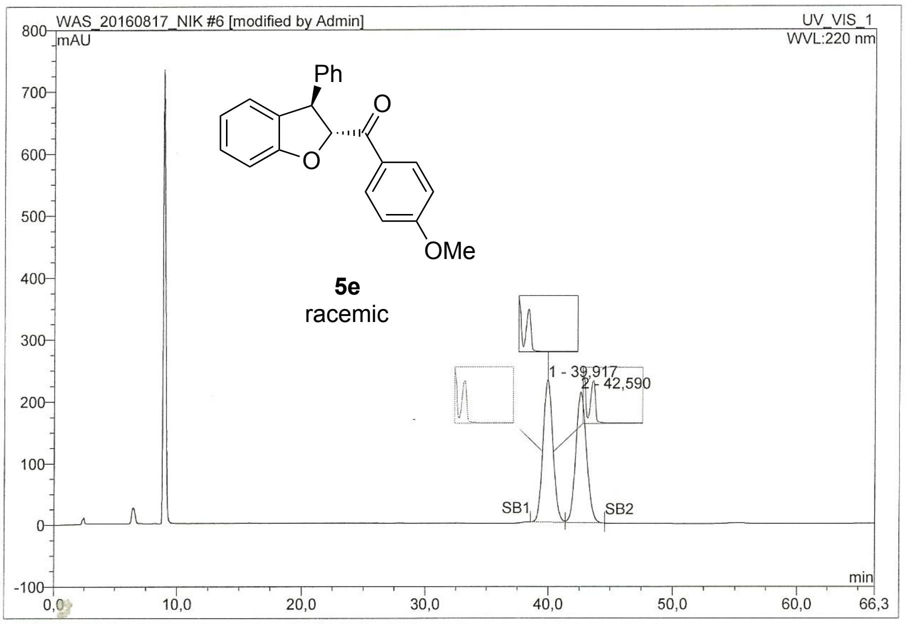

No.	Ret.Time min	Peak Name	Height mAU	Area mAU*min	Rel.Area %	Amount	Type
1	39,92	n.a.	230,659	215,981	49,90	n.a.	BM
2	42,59	n.a.	212,404	216,846	50,10	n.a.	MB
**Total:**			443,062	432,827	100,00	0,000	

7 NIK-149-02 (100/1MeOH) flow 0,5

Sample Name:	**NIK-149-02 (100/1MeOH) flow 0,5**	*Injection Volume:*	**10,0**
Vial Number:	**GD2**	*Channel:*	**UV_VIS_1**
Sample Type:	**unknown**	*Wavelength:*	**220**
Control Program:	**OD_H_90Min_95_5_flow05**	*Bandwidth:*	**4**
Quantif. Method:	**default**	*Temperature/Column:*	**10**
Recording Time:	**17.8.2016 16:17**	*Flow ml/min:*	**0,500**
Run Time (min):	**48,82**	*Sample Amount:*	**1,0000**

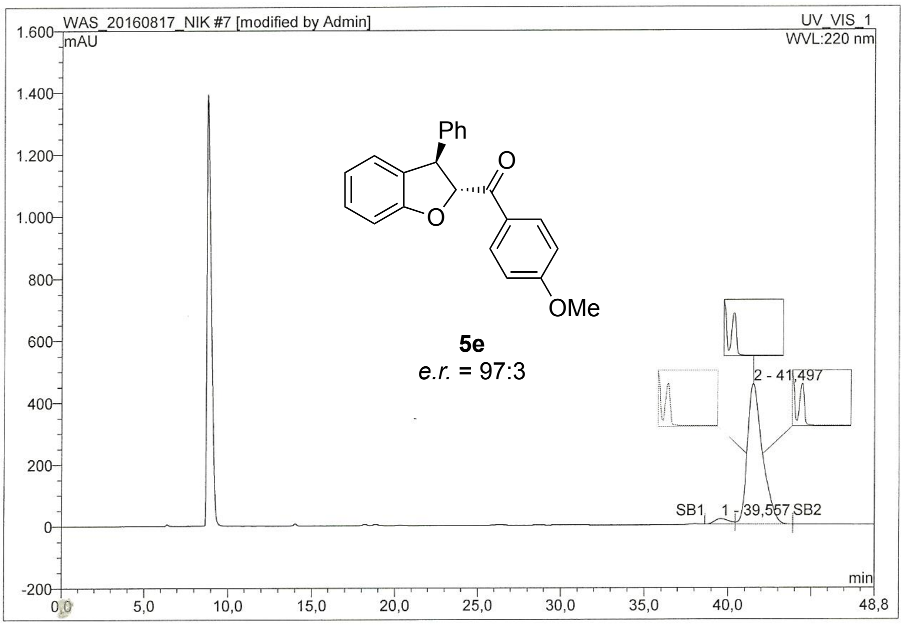

No.	Ret.Time min	Peak Name	Height mAU	Area mAU*min	Rel.Area %	Amount	Type
1	39,56	n.a.	17,785	17,591	3,54	n.a.	BM
2	41,50	n.a.	454,857	478,709	96,46	n.a.	MB
**Total:**			472,642	496,300	100,00	0,000	

Chromeleon (c) Dionex 1996-2006
Version 6.80 SR12 Build 3578 (207169)

5 NIK-079-02 (95/5) flow 0,5

Sample Name:	NIK-079-02 (95/5) flow 0,5	*Injection Volume:*	10,0
Vial Number:	GD5	*Channel:*	UV_VIS_1
Sample Type:	unknown	*Wavelength:*	220
Control Program:	YMC_60Min_95_5_flow0_5	*Bandwidth:*	4
Quantif. Method:	AD_H	*Temperature/Column:*	10
Recording Time:	2.8.2016 14:06	*Flow ml/min:*	0,500
Run Time (min):	60,00	*Sample Amount:*	1,0000

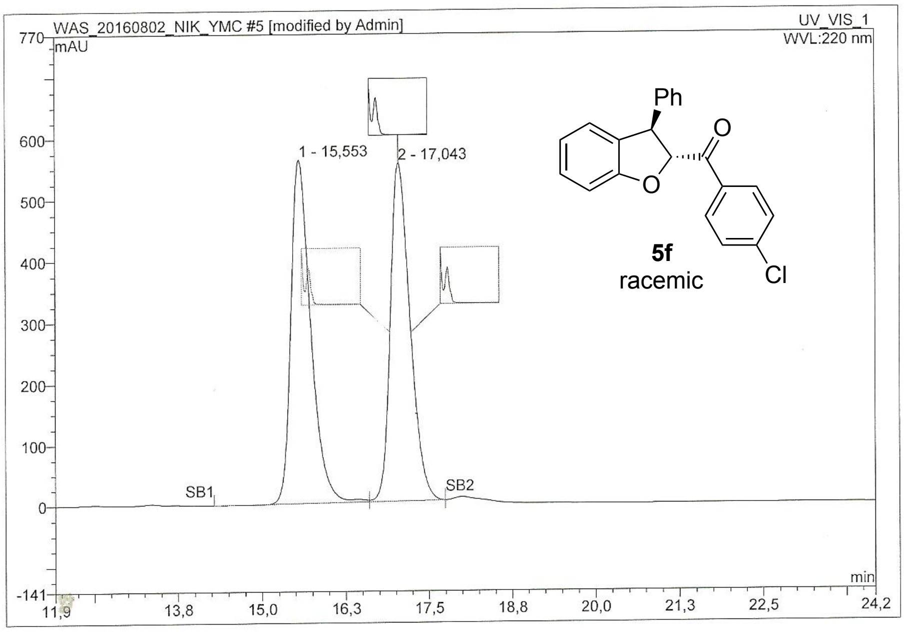

No.	Ret.Time min	Peak Name	Height mAU	Area mAU*min	Rel.Area %	Amount	Type
1	15,55	n.a.	562,224	192,856	50,30	n.a.	BM *
2	17,04	n.a.	553,002	190,529	49,70	n.a.	MB*
Total:			1115,225	383,384	100,00	0,000	

6 NIK-141-02 (95/5) flow 0,5

Sample Name:	**NIK-141-02 (95/5) flow 0,5**	*Injection Volume:*	**10,0**
Vial Number:	**GD6**	*Channel:*	**UV_VIS_1**
Sample Type:	**unknown**	*Wavelength:*	**220**
Control Program:	**YMC_60Min_95_5_flow0_5**	*Bandwidth:*	**4**
Quantif. Method:	**AD_H**	*Temperature/Column:*	**10**
Recording Time:	**2.8.2016 15:07**	*Flow ml/min:*	**0,500**
Run Time (min):	**60,00**	*Sample Amount:*	**1,0000**

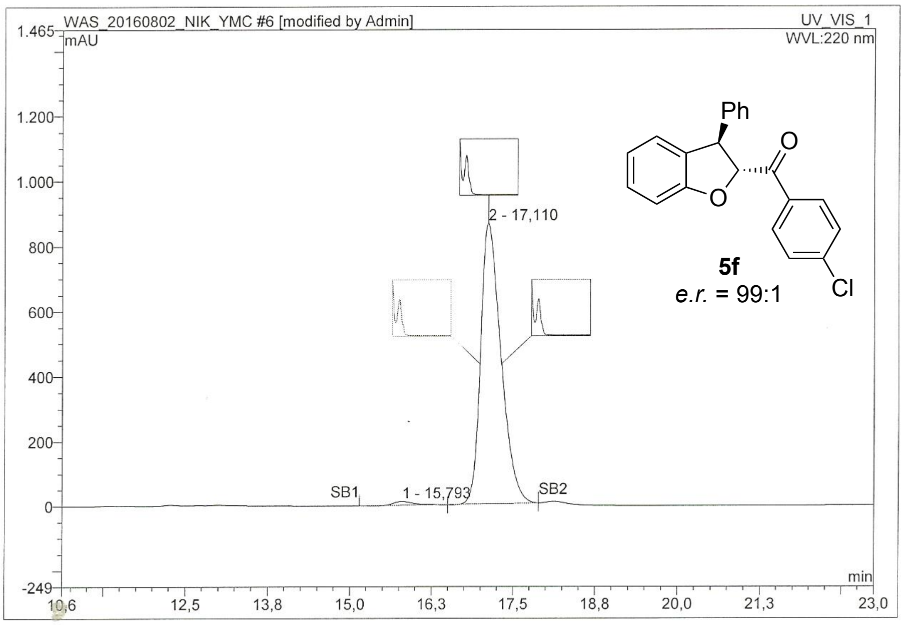

No.	Ret.Time min	Peak Name	Height mAU	Area mAU*min	Rel.Area %	Amount	Type
1	15,79	n.a.	12,069	4,048	1,27	n.a.	BM *
2	17,11	n.a.	863,301	313,963	98,73	n.a.	MB*
Total:			875,371	318,012	100,00	0,000	

default/Integration

Chromeleon (c) Dionex 1996-2006
Version 6.80 SR12 Build 3578 (207169)

4 NIK-111-02 (95/5) flow 0,5

Sample Name:	**NIK-111-02 (95/5) flow 0,5**	*Injection Volume:*	**10,0**
Vial Number:	**GC4**	*Channel:*	**UV_VIS_1**
Sample Type:	**unknown**	*Wavelength:*	**220**
Control Program:	**YMC_60Min_95_5_flow0_5**	*Bandwidth:*	**4**
Quantif. Method:	**AD_H**	*Temperature/Column:*	**10**
Recording Time:	**26.7.2016 13:27**	*Flow ml/min:*	**0,500**
Run Time (min):	**60,00**	*Sample Amount:*	**1,0000**

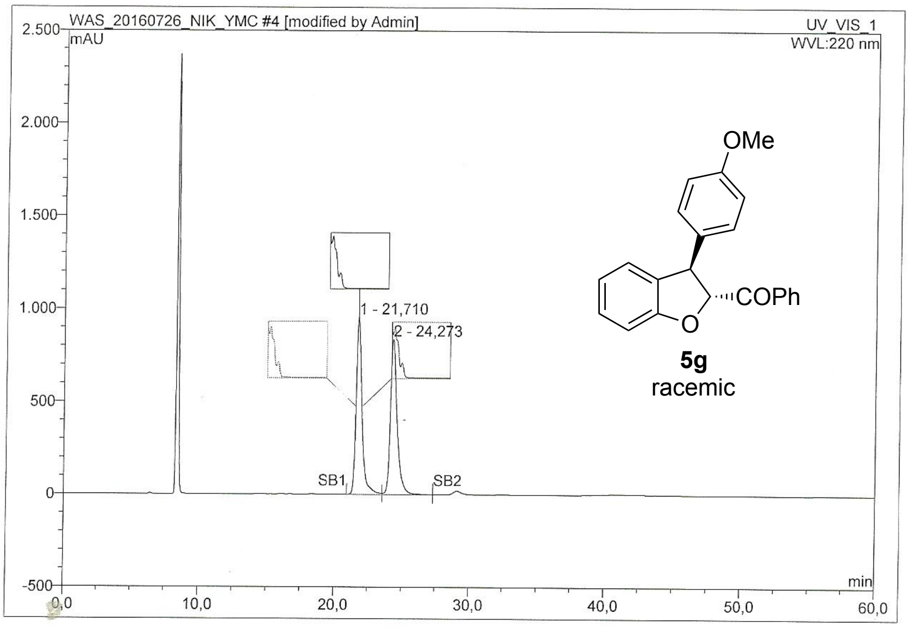

No.	Ret.Time min	Peak Name	Height mAU	Area mAU*min	Rel.Area %	Amount	Type
1	21,71	n.a.	956,505	487,347	50,00	n.a.	BM
2	24,27	n.a.	838,057	487,266	50,00	n.a.	MB
**Total:**			1794,562	974,613	100,00	0,000	

5 NIK-136-02 (95/5) flow 0,5

Sample Name:	**NIK-136-02 (95/5) flow 0,5**	*Injection Volume:*	**10,0**
Vial Number:	**GC5**	*Channel:*	**UV_VIS_2**
Sample Type:	**unknown**	*Wavelength:*	**250**
Control Program:	**YMC_60Min_95_5_flow0_5**	*Bandwidth:*	**4**
Quantif. Method:	**AD_H**	*Temperature/Column:*	**10**
Recording Time:	**26.7.2016 14:28**	*Flow ml/min:*	**0,500**
Run Time (min):	**60,00**	*Sample Amount:*	**1,0000**

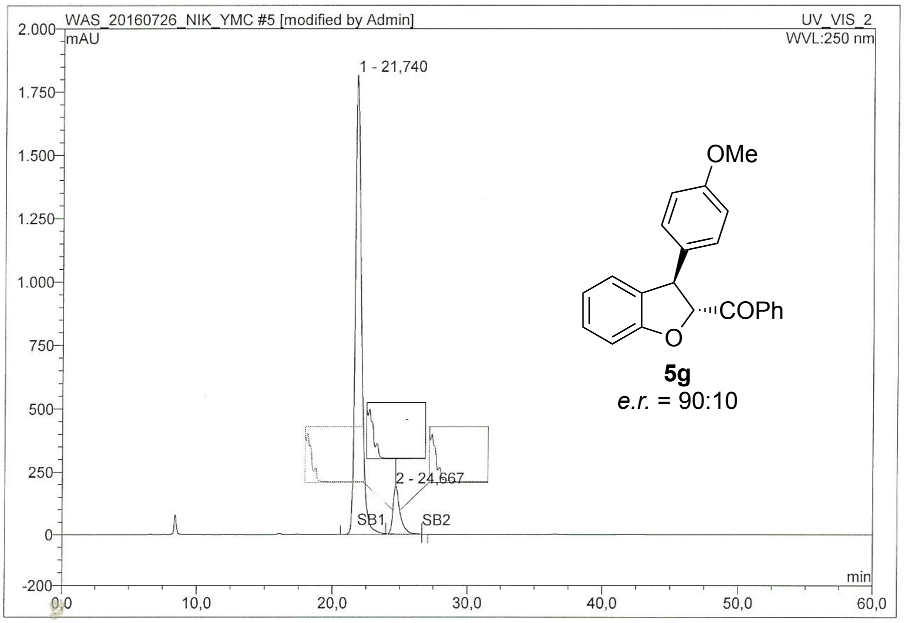

No.	Ret.Time min	Peak Name	Height mAU	Area mAU*min	Rel.Area %	Amount	Type
1	21,74	n.a.	1816,810	974,850	89,65	n.a.	BMB
2	24,67	n.a.	188,993	112,597	10,35	n.a.	Rd
**Total:**			2005,803	1087,447	100,00	0,000	

1 NIK-140-02 (250/1) flow 0,5

Sample Name:	**NIK-140-02 (250/1) flow 0,5**	*Injection Volume:*	**10,0**
Vial Number:	**BA6**	*Channel:*	**UV_VIS_1**
Sample Type:	**unknown**	*Wavelength:*	**220**
Control Program:	**YMC_90Min_95_5_flow0_5**	*Bandwidth:*	**4**
Quantif. Method:	**AD_H**	*Temperature/Column:*	**10**
Recording Time:	**11.10.2016 13:35**	*Flow ml/min:*	**0,500**
Run Time (min):	**90,00**	*Sample Amount:*	**1,0000**

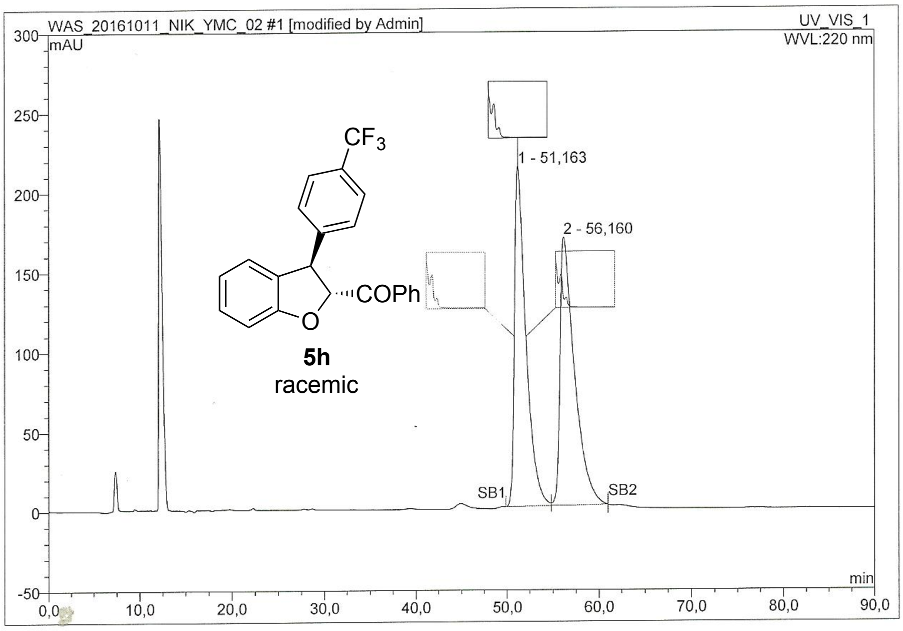

No.	Ret.Time min	Peak Name	Height mAU	Area mAU*min	Rel.Area %	Amount	Type
1	51,16	n.a.	213,592	307,402	49,99	n.a.	BM
2	56,16	n.a.	168,204	307,522	50,01	n.a.	MB
**Total:**			381,796	614,925	100,00	0,000	

3 NIK-161-02 (250/1) flow 0,5

Sample Name:	**NIK-161-02 (250/1) flow 0,5**	*Injection Volume:*	**10,0**
Vial Number:	**BB3**	*Channel:*	**UV_VIS_1**
Sample Type:	**unknown**	*Wavelength:*	**220**
Control Program:	**YMC_90Min_95_5_flow0_5**	*Bandwidth:*	**4**
Quantif. Method:	**AD_H**	*Temperature/Column:*	**10**
Recording Time:	**11.10.2016 15:53**	*Flow ml/min:*	**0,500**
Run Time (min):	**77,81**	*Sample Amount:*	**1,0000**

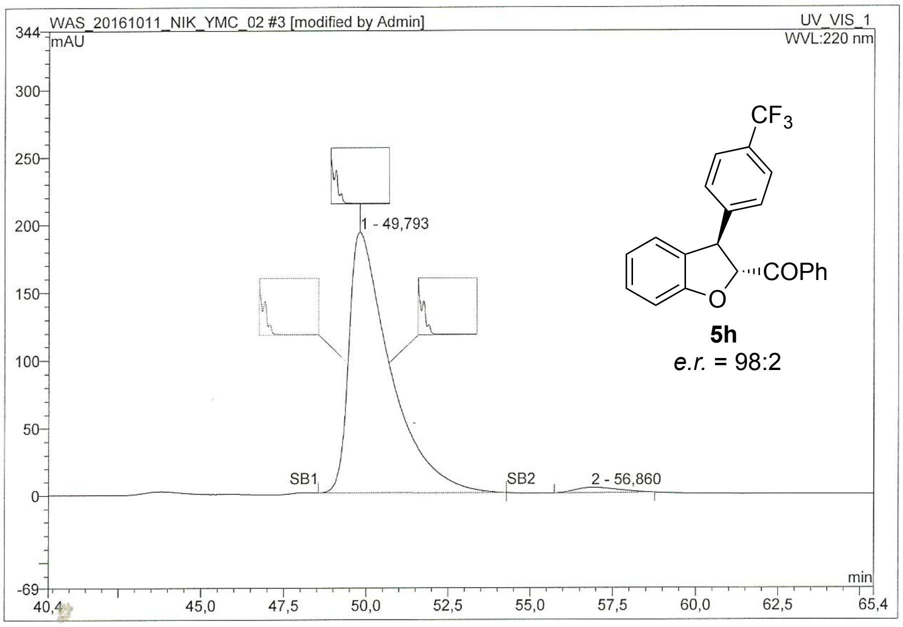

No.	Ret.Time min	Peak Name	Height mAU	Area mAU*min	Rel.Area %	Amount	Type
1	49,79	n.a.	192,848	281,931	97,91	n.a.	BMB
2	56,86	n.a.	3,955	6,009	2,09	n.a.	BMB*
**Total:**			196,803	287,940	100,00	0,000	

default/Integration

Chromeleon (c) Dionex 1996-2006
Version 6.80 SR12 Build 3578 (207169)

3 NIK-123-02 (hexan/iPrOH/MeOH 100/3/1)

Sample Name:	**NIK-123-02 (hexan/iPrOH/MeOH 100/3/1)**	*Injection Volume:*	**10,0**
Vial Number:	**GD1**	*Channel:*	**UV_VIS_2**
Sample Type:	**unknown**	*Wavelength:*	**290**
Control Program:	**OD_H_90Min_95_5_flow08**	*Bandwidth:*	**4**
Quantif. Method:	**default**	*Temperature/Column:*	**10**
Recording Time:	**11.8.2016 15:17**	*Flow ml/min:*	**0,800**
Run Time (min):	**15,06**	*Sample Amount:*	**1,0000**

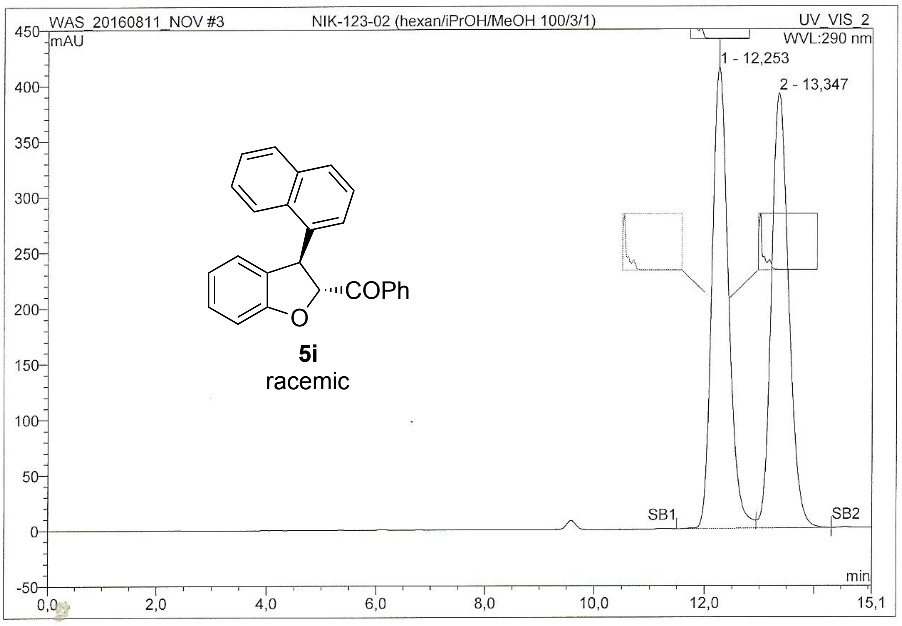

No.	Ret.Time min	Peak Name	Height mAU	Area mAU*min	Rel.Area %	Amount	Type
1	12,25	n.a.	414,906	144,266	49,94	n.a.	BM
2	13,35	n.a.	390,919	144,611	50,06	n.a.	MB
**Total:**			805,825	288,877	100,00	0,000	

5 NIK-139-02 (verdünnter, hexan/iPrOH/MeOH 100/3/1)

Sample Name:	**NIK-139-02 (verdünnter, hexan/iPrOH/MeOI**	*Injection Volume:*	**10,0**
Vial Number:	**GD2**	*Channel:*	**UV_VIS_2**
Sample Type:	**unknown**	*Wavelength:*	**290**
Control Program:	**OD_H_25Min_95_5_flow08**	*Bandwidth:*	**4**
Quantif. Method:	**default**	*Temperature/Column:*	**10**
Recording Time:	**11.8.2016 15:49**	*Flow ml/min:*	**0,800**
Run Time (min):	**17,65**	*Sample Amount:*	**1,0000**

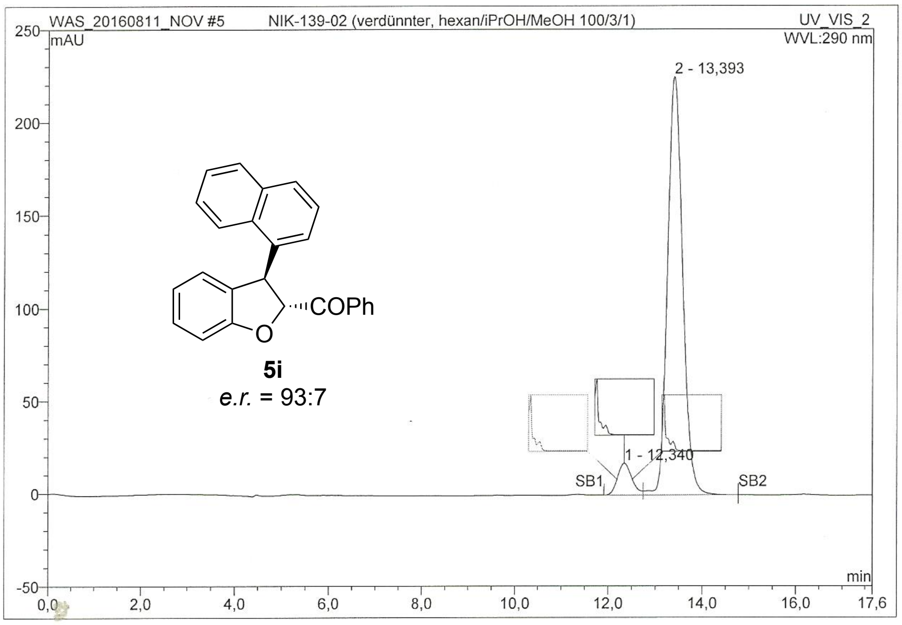

No.	Ret.Time min	Peak Name	Height mAU	Area mAU*min	Rel.Area %	Amount	Type
1	12,34	n.a.	17,358	6,115	6,78	n.a.	BM
2	13,39	n.a.	225,296	84,097	93,22	n.a.	MB
**Total:**			242,654	90,212	100,00	0,000	

3 NIK-157-02 (99/1) flow 0,5

Sample Name:	**NIK-157-02 (99/1) flow 0,5**	*Injection Volume:*	**10,0**
Vial Number:	**GE3**	*Channel:*	**UV_VIS_1**
Sample Type:	**unknown**	*Wavelength:*	**220**
Control Program:	**YMC_60Min_95_5_flow0_5**	*Bandwidth:*	**4**
Quantif. Method:	**AD_H**	*Temperature/Column:*	**10**
Recording Time:	**14.9.2016 15:04**	*Flow ml/min:*	**0,500**
Run Time (min):	**60,00**	*Sample Amount:*	**1,0000**

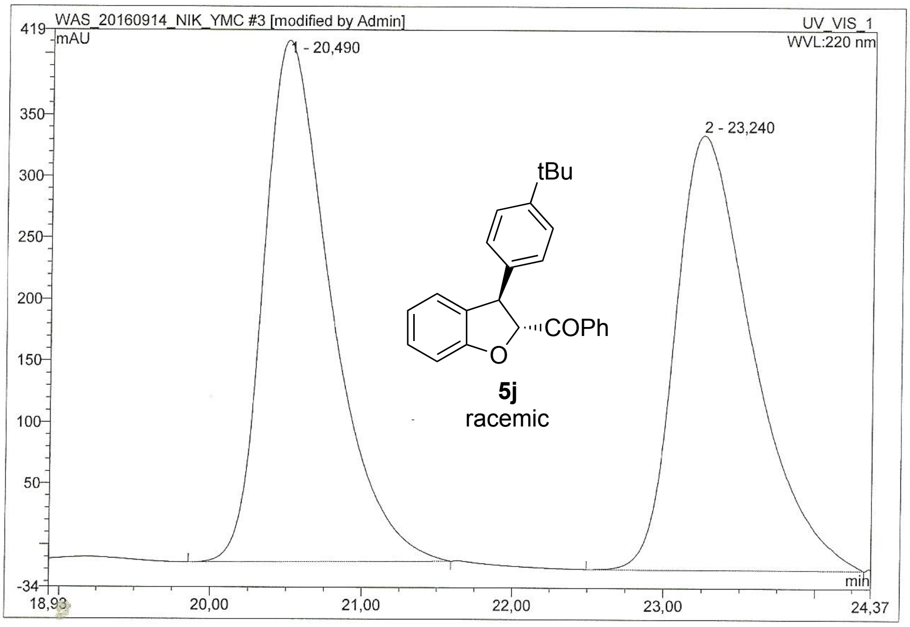

No.	Ret.Time min	Peak Name	Height mAU	Area mAU*min	Rel.Area %	Amount	Type
1	20,49	n.a.	424,489	218,818	51,07	n.a.	BMB
2	23,24	n.a.	353,796	209,670	48,93	n.a.	BMB
**Total:**			778,284	428,488	100,00	0,000	

Chromeleon (c) Dionex 1996-2006
Version 6.80 SR12 Build 3578 (207169)

4 NIK-158-02 (99/1) flow 0,5

Sample Name:	**NIK-158-02 (99/1) flow 0,5**	*Injection Volume:*	**10,0**
Vial Number:	**GE4**	*Channel:*	**UV_VIS_2**
Sample Type:	**unknown**	*Wavelength:*	**250**
Control Program:	**YMC_60Min_95_5_flow0_5**	*Bandwidth:*	**4**
Quantif. Method:	**AD_H**	*Temperature/Column:*	**10**
Recording Time:	**14.9.2016 16:05**	*Flow ml/min:*	**0,500**
Run Time (min):	**60,00**	*Sample Amount:*	**1,0000**

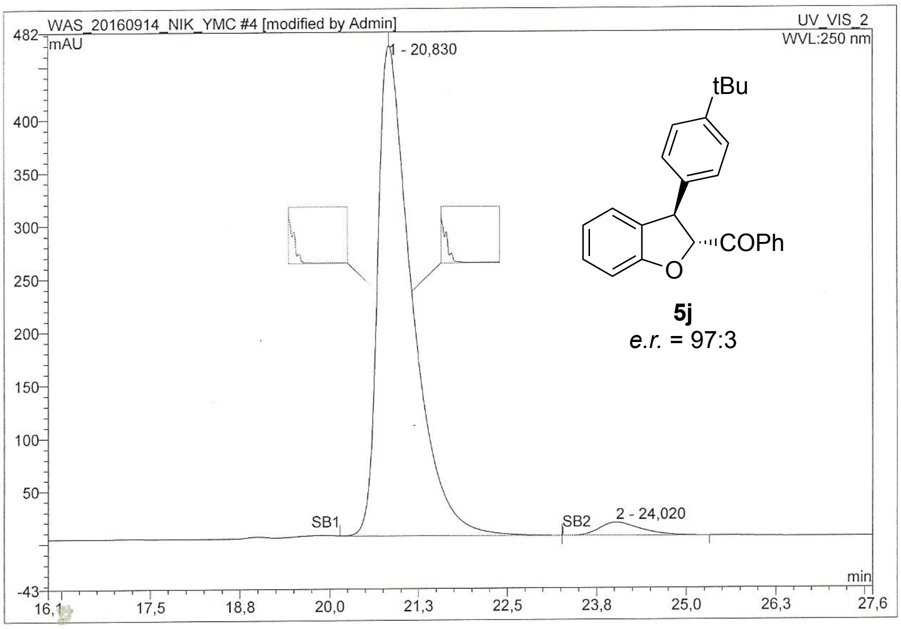

No.	Ret.Time min	Peak Name	Height mAU	Area mAU*min	Rel.Area %	Amount	Type
1	20,83	n.a.	462,756	259,502	96,91	n.a.	BMB
2	24,02	n.a.	12,390	8,265	3,09	n.a.	BMB
**Total:**			475,146	267,767	100,00	0,000	

default/Integration

Chromeleon (c) Dionex 1996-2006
Version 6.80 SR12 Build 3578 (207169)

2 NIK-121-02 (95/5) flow 0,5

Sample Name:	NIK-121-02 (95/5) flow 0,5	*Injection Volume:*	10,0
Vial Number:	GE2	*Channel:*	UV_VIS_2
Sample Type:	unknown	*Wavelength:*	250
Control Program:	OD_H_90Min_95_5_flow05	*Bandwidth:*	4
Quantif. Method:	default	*Temperature/Column:*	10
Recording Time:	15.9.2016 11:52	*Flow ml/min:*	0,500
Run Time (min):	56,06	*Sample Amount:*	1,0000

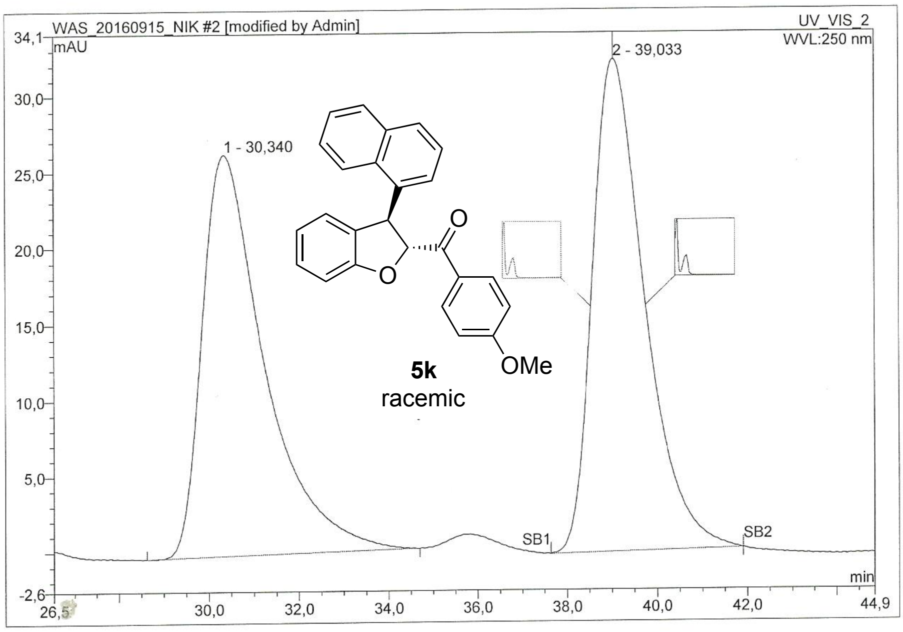

No.	Ret.Time min	Peak Name	Height mAU	Area mAU*min	Rel.Area %	Amount	Type
1	30,34	n.a.	26,439	42,359	49,33	n.a.	BMB*
2	39,03	n.a.	32,462	43,510	50,67	n.a.	BMB
Total:			58,901	85,869	100,00	0,000	

3 NIK-151-02 (95/5) flow 0,5

Sample Name:	**NIK-151-02 (95/5) flow 0,5**	*Injection Volume:*	**10,0**
Vial Number:	**GE6**	*Channel:*	**UV_VIS_1**
Sample Type:	**unknown**	*Wavelength:*	**220**
Control Program:	**OD_H_90Min_95_5_flow05**	*Bandwidth:*	**4**
Quantif. Method:	**default**	*Temperature/Column:*	**10**
Recording Time:	**15.9.2016 12:49**	*Flow ml/min:*	**0,500**
Run Time (min):	**64,71**	*Sample Amount:*	**1,0000**

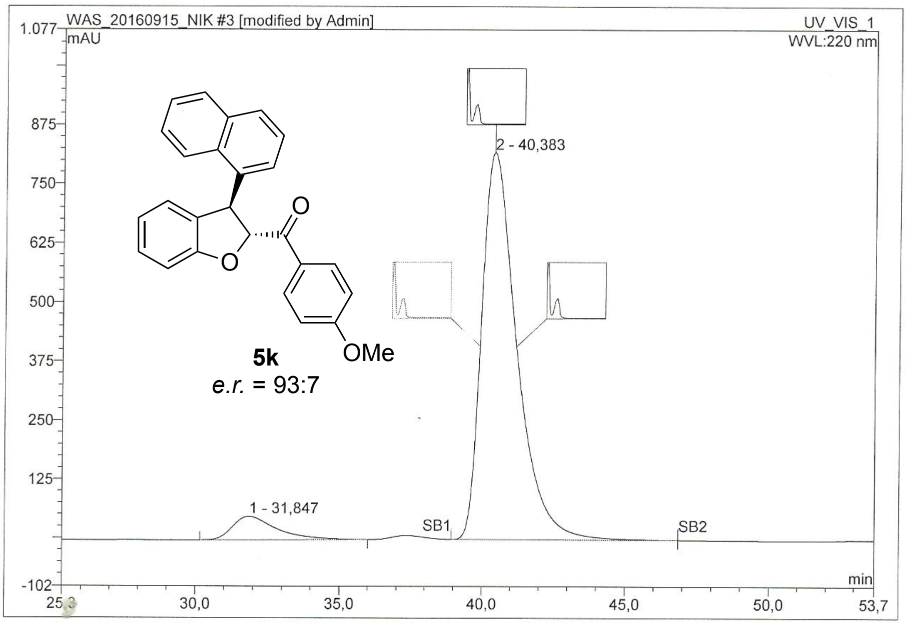

No.	Ret.Time min	Peak Name	Height mAU	Area mAU*min	Rel.Area %	Amount	Type
1	31,85	n.a.	49,867	88,060	6,94	n.a.	BMB*
2	40,38	n.a.	819,825	1180,062	93,06	n.a.	BMB*
**Total:**			869,692	1268,122	100,00	0,000	

11 NIK-122-02 (99/1) flow 0,5

Sample Name:	**NIK-122-02 (99/1) flow 0,5**	*Injection Volume:*	**10,0**
Vial Number:	**BA5**	*Channel:*	**UV_VIS_1**
Sample Type:	**unknown**	*Wavelength:*	**220**
Control Program:	**YMC_60Min_95_5_flow0_5**	*Bandwidth:*	**4**
Quantif. Method:	**AD_H**	*Temperature/Column:*	**10**
Recording Time:	**9.8.2016 0:54**	*Flow ml/min:*	**0,500**
Run Time (min):	**60,00**	*Sample Amount:*	**1,0000**

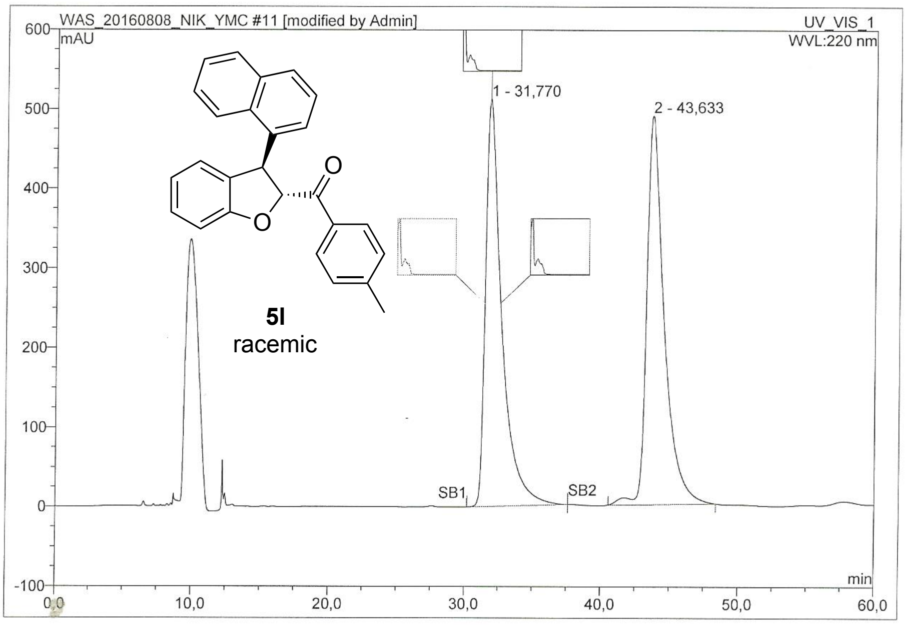

No.	Ret.Time min	Peak Name	Height mAU	Area mAU*min	Rel.Area %	Amount	Type
1	31,77	n.a.	511,889	716,836	49,72	n.a.	BMB
2	43,63	n.a.	488,546	724,844	50,28	n.a.	BMB*
**Total:**			1000,435	1441,681	100,00	0,000	

default/Integration

Chromeleon (c) Dionex 1996-2006
Version 6.80 SR12 Build 3578 (207169)

12 NIK-145-02 (99/1) flow 0,5

Sample Name:	**NIK-145-02 (99/1) flow 0,5**	*Injection Volume:*	**10,0**
Vial Number:	**BA6**	*Channel:*	**UV_VIS_2**
Sample Type:	**unknown**	*Wavelength:*	**250**
Control Program:	**YMC_60Min_95_5_flow0_5**	*Bandwidth:*	**4**
Quantif. Method:	**AD_H**	*Temperature/Column:*	**10**
Recording Time:	**9.8.2016 1:55**	*Flow ml/min:*	**0,500**
Run Time (min):	**60,00**	*Sample Amount:*	**1,0000**

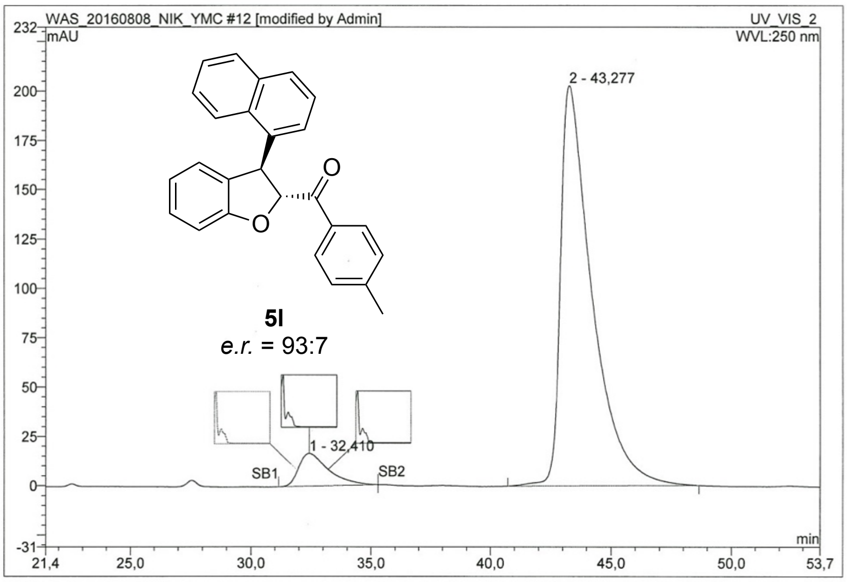

No.	Ret.Time min	Peak Name	Height mAU	Area mAU*min	Rel.Area %	Amount	Type
1	32,41	n.a.	16,623	23,736	7,18	n.a.	BMB
2	43,28	n.a.	202,538	306,824	92,82	n.a.	BMB
Total:			219,161	330,561	100,00	0,000	

7 NIK-124-02 (99/1) flow 0,5

Sample Name:	**NIK-124-02 (99/1) flow 0,5**	*Injection Volume:*	**10,0**
Vial Number:	**BA1**	*Channel:*	**UV_VIS_2**
Sample Type:	**unknown**	*Wavelength:*	**250**
Control Program:	**YMC_60Min_95_5_flow0_5**	*Bandwidth:*	**4**
Quantif. Method:	**AD_H**	*Temperature/Column:*	**10**
Recording Time:	**8.8.2016 20:52**	*Flow ml/min:*	**0,500**
Run Time (min):	**60,00**	*Sample Amount:*	**1,0000**

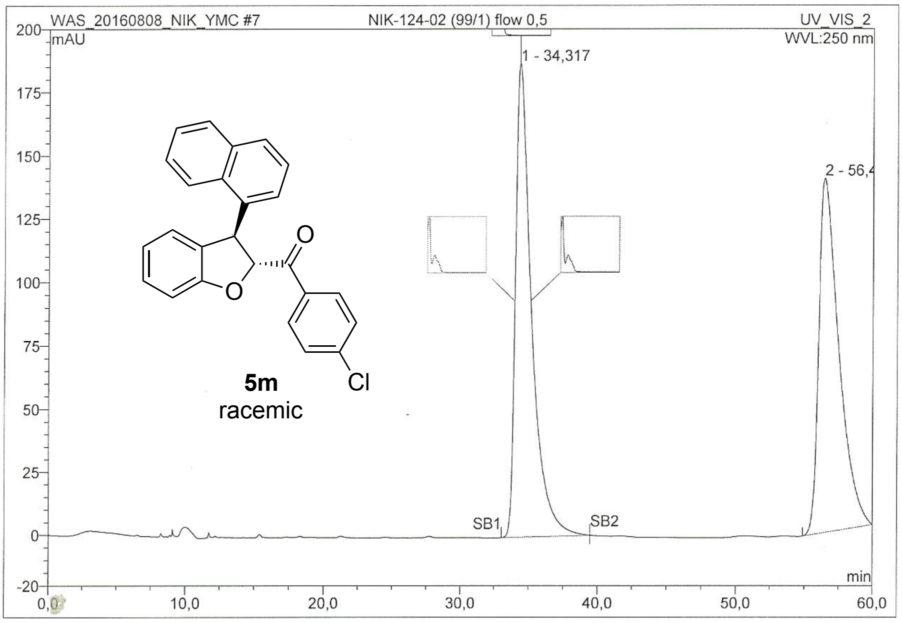

No.	Ret.Time min	Peak Name	Height mAU	Area mAU*min	Rel.Area %	Amount	Type
1	34,32	n.a.	187,036	254,176	51,43	n.a.	BMB
2	56,45	n.a.	139,999	240,052	48,57	n.a.	BMB
**Total:**			327,035	494,228	100,00	0,000	

8 NIK-143-02 (99/1) flow 0,5

Sample Name:	**NIK-143-02 (99/1) flow 0,5**	*Injection Volume:*	**10,0**
Vial Number:	**BA2**	*Channel:*	**UV_VIS_2**
Sample Type:	**unknown**	*Wavelength:*	**250**
Control Program:	**YMC_60Min_95_5_flow0_5**	*Bandwidth:*	**4**
Quantif. Method:	**AD_H**	*Temperature/Column:*	**10**
Recording Time:	**8.8.2016 21:52**	*Flow ml/min:*	**0,500**
Run Time (min):	**60,00**	*Sample Amount:*	**1,0000**

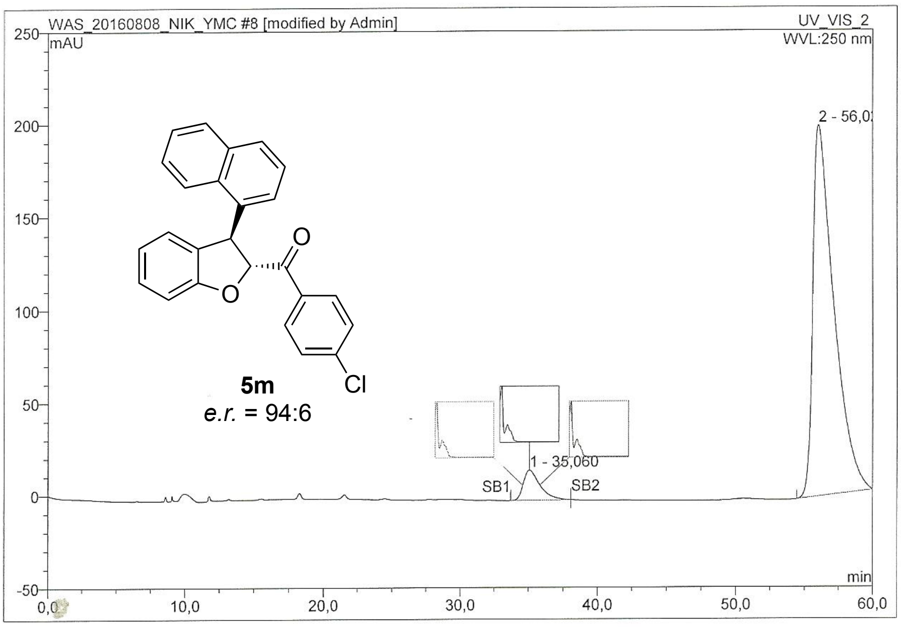

No.	Ret.Time min	Peak Name	Height mAU	Area mAU*min	Rel.Area %	Amount	Type
1	35,06	n.a.	16,505	23,138	6,07	n.a.	BMB
2	56,03	n.a.	199,785	358,177	93,93	n.a.	BMB
Total:			216,290	381,315	100,00	0,000	

3 NIK-110-02 (95/5) flow 0,5

Sample Name:	NIK-110-02 (95/5) flow 0,5	*Injection Volume:*	10,0
Vial Number:	GD7	*Channel:*	UV_VIS_1
Sample Type:	unknown	*Wavelength:*	220
Control Program:	OD_H_60Min_95_5_flow05_25grad	*Bandwidth:*	4
Quantif. Method:	default	*Temperature/Column:*	25
Recording Time:	17.8.2016 12:01	*Flow ml/min:*	0,500
Run Time (min):	33,97	*Sample Amount:*	1,0000

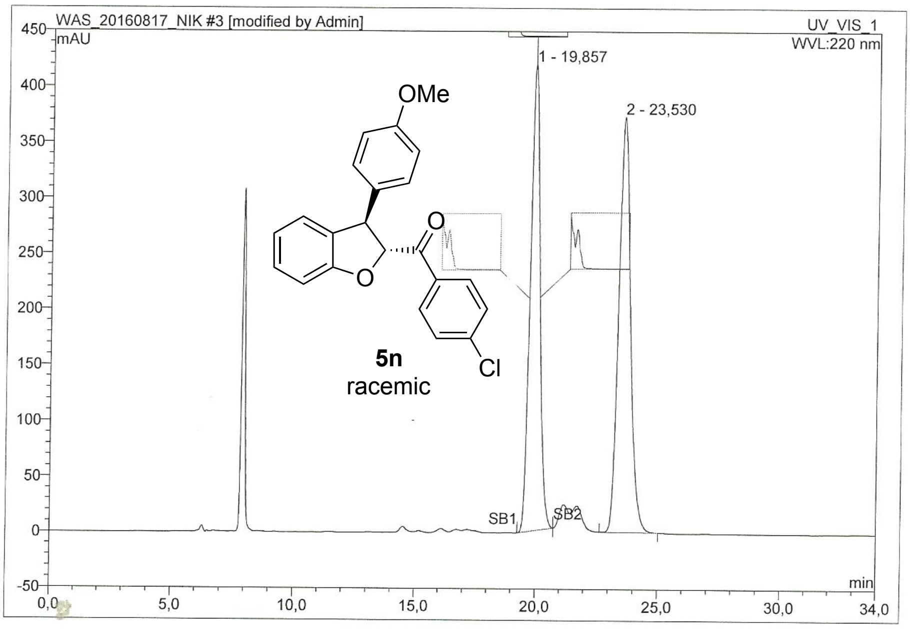

No.	Ret.Time min	Peak Name	Height mAU	Area mAU*min	Rel.Area %	Amount	Type
1	19,86	n.a.	418,034	194,094	48,26	n.a.	BMB
2	23,53	n.a.	372,204	208,092	51,74	n.a.	BMB
Total:			790,238	402,186	100,00	0,000	

Chromeleon (c) Dionex 1996-2006
Version 6.80 SR12 Build 3578 (207169)

4 NIK-142-02 (95/5) flow 0,5

Sample Name:	**NIK-142-02 (95/5) flow 0,5**	*Injection Volume:*	**10,0**
Vial Number:	**GD8**	*Channel:*	**UV_VIS_1**
Sample Type:	**unknown**	*Wavelength:*	**220**
Control Program:	**OD_H_60Min_95_5_flow05_25grad**	*Bandwidth:*	**4**
Quantif. Method:	**default**	*Temperature/Column:*	**25**
Recording Time:	**17.8.2016 12:36**	*Flow ml/min:*	**0,500**
Run Time (min):	**49,68**	*Sample Amount:*	**1,0000**

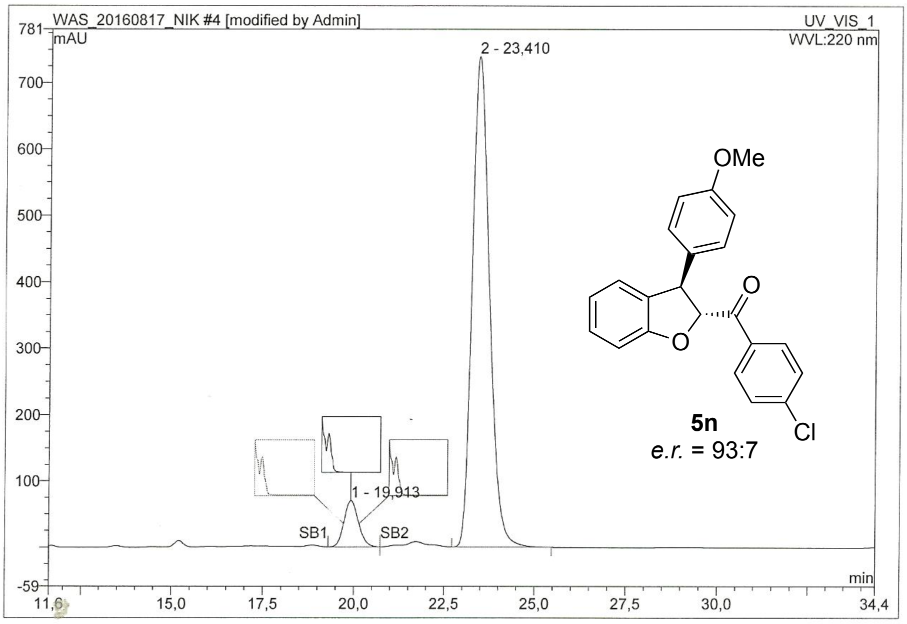

No.	Ret.Time min	Peak Name	Height mAU	Area mAU*min	Rel.Area %	Amount	Type
1	19,91	n.a.	70,079	32,127	7,26	n.a.	BMB
2	23,41	n.a.	739,086	410,303	92,74	n.a.	BMB
**Total:**			809,165	442,430	100,00	0,000	

Chromeleon (c) Dionex 1996-2006
Version 6.80 SR12 Build 3578 (207169)

8 NIK-113-02 (98/2) flow 0,5

Sample Name:	**NIK-113-02 (98/2) flow 0,5**	*Injection Volume:*	**10,0**
Vial Number:	**BD1**	*Channel:*	**UV_VIS_1**
Sample Type:	**unknown**	*Wavelength:*	**220**
Control Program:	**YMC_90Min_95_5_flow0_5**	*Bandwidth:*	**4**
Quantif. Method:	**AD_H**	*Temperature/Column:*	**10**
Recording Time:	**21.12.2016 16:00**	*Flow ml/min:*	**0,500**
Run Time (min):	**90,00**	*Sample Amount:*	**1,0000**

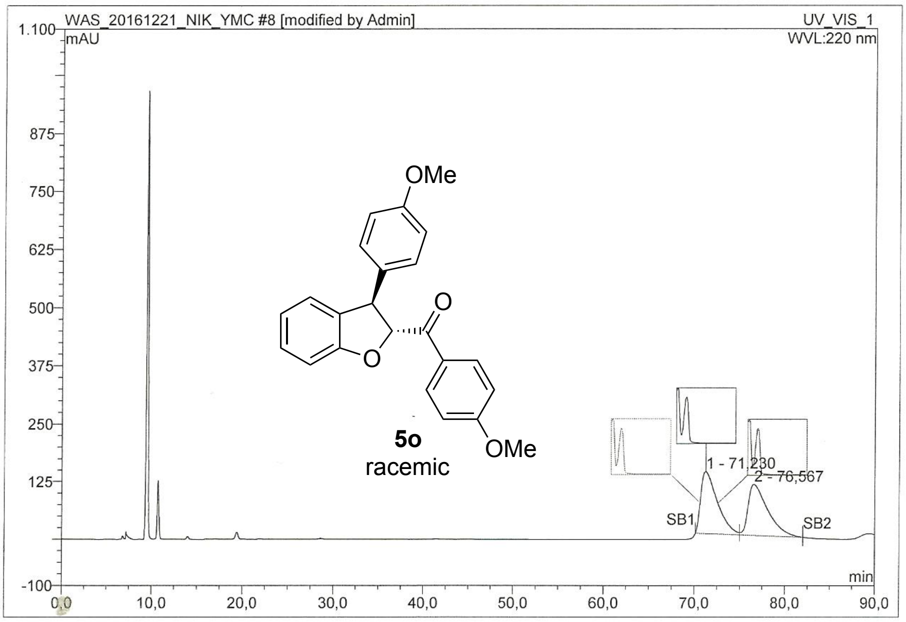

No.	Ret.Time min	Peak Name	Height mAU	Area mAU*min	Rel.Area %	Amount	Type
1	71,23	n.a.	135,349	287,273	50,70	n.a.	BM *
2	76,57	n.a.	111,011	279,327	49,30	n.a.	MB*
Total:			246,360	566,600	100,00	0,000	

9 NIK-150-02 (98/2) flow 0,5

Sample Name:	**NIK-150-02 (98/2) flow 0,5**	*Injection Volume:*	**10,0**
Vial Number:	**BE1**	*Channel:*	**UV_VIS_1**
Sample Type:	**unknown**	*Wavelength:*	**220**
Control Program:	**YMC_90Min_95_5_flow0_5**	*Bandwidth:*	**4**
Quantif. Method:	**AD_H**	*Temperature/Column:*	**10**
Recording Time:	**21.12.2016 17:30**	*Flow ml/min:*	**0,500**
Run Time (min):	**90,00**	*Sample Amount:*	**1,0000**

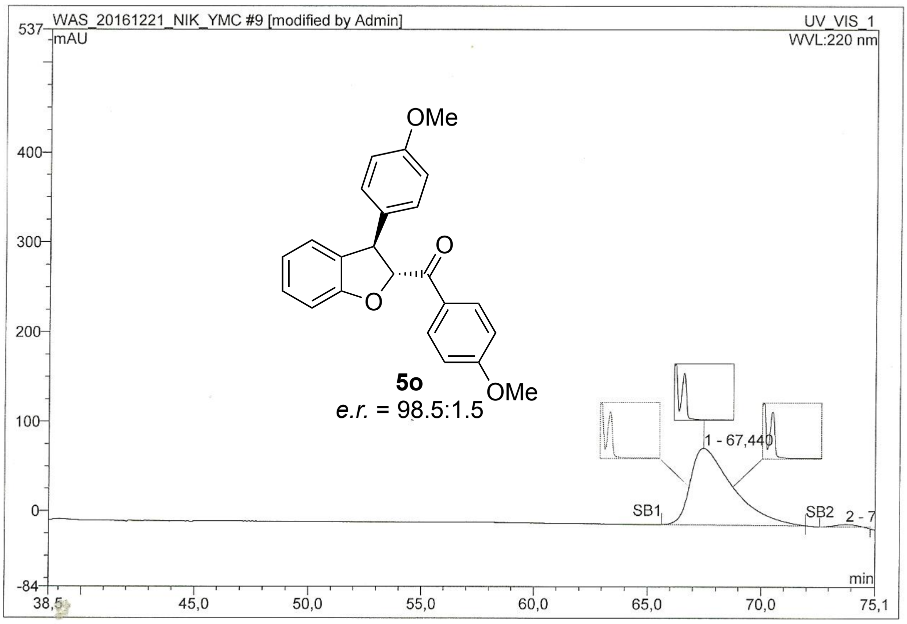

No.	Ret.Time min	Peak Name	Height mAU	Area mAU*min	Rel.Area %	Amount	Type
1	67,44	n.a.	85,692	185,342	98,78	n.a.	BMB
2	73,74	n.a.	2,556	2,293	1,22	n.a.	BM *
**Total:**			88,249	187,634	100,00	0,000	

Chromeleon (c) Dionex 1996-2006
Version 6.80 SR12 Build 3578 (207169)

2 NIK-186-02 (98/2) flow 0,5

Sample Name:	**NIK-186-02 (98/2) flow 0,5**	*Injection Volume:*	**10,0**
Vial Number:	**BA1**	*Channel:*	**UV_VIS_2**
Sample Type:	**unknown**	*Wavelength:*	**250**
Control Program:	**YMC_60Min_95_5_flow0_5**	*Bandwidth:*	**4**
Quantif. Method:	**AD_H**	*Temperature/Column:*	**10**
Recording Time:	**5.10.2016 13:26**	*Flow ml/min:*	**0,500**
Run Time (min):	**60,00**	*Sample Amount:*	**1,0000**

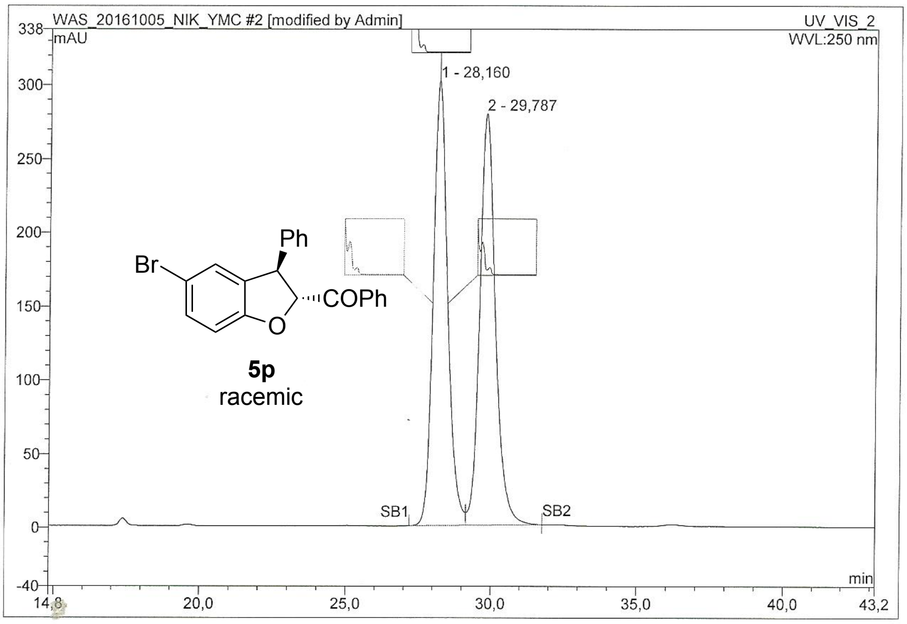

No.	Ret.Time min	Peak Name	Height mAU	Area mAU*min	Rel.Area %	Amount	Type
1	28,16	n.a.	301,414	173,979	49,68	n.a.	BM
2	29,79	n.a.	278,842	176,210	50,32	n.a.	MB
**Total:**			580,256	350,189	100,00	0,000	

3 NIK-187-02 (98/2) flow 0,5

Sample Name:	**NIK-187-02 (98/2) flow 0,5**	*Injection Volume:*	**10,0**
Vial Number:	**BA2**	*Channel:*	**UV_VIS_2**
Sample Type:	**unknown**	*Wavelength:*	**250**
Control Program:	**YMC_60Min_95_5_flow0_5**	*Bandwidth:*	**4**
Quantif. Method:	**AD_H**	*Temperature/Column:*	**10**
Recording Time:	**5.10.2016 15:22**	*Flow ml/min:*	**0,500**
Run Time (min):	**60,00**	*Sample Amount:*	**1,0000**

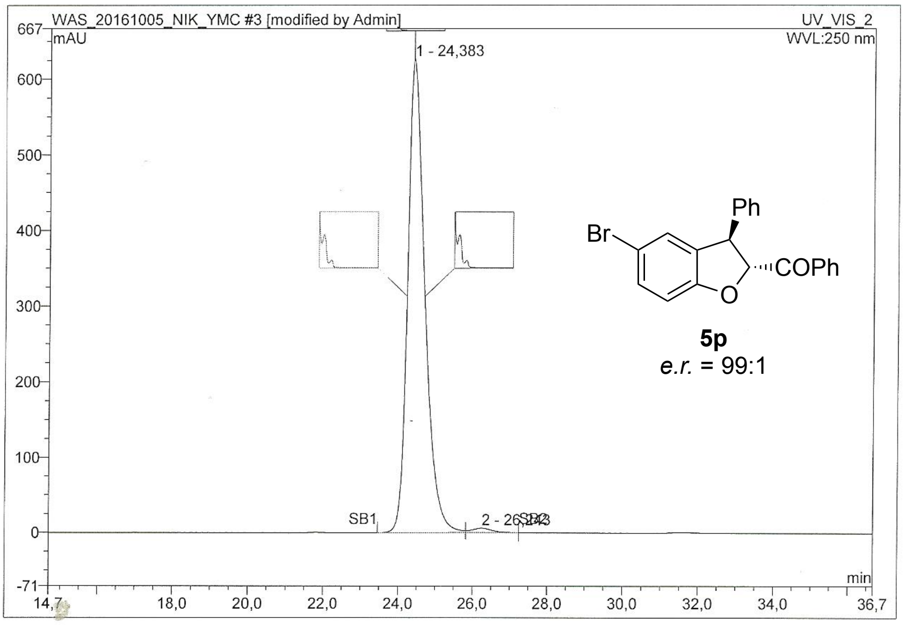

No.	Ret.Time min	Peak Name	Height mAU	Area mAU*min	Rel.Area %	Amount	Type
1	24,38	n.a.	626,669	338,631	98,89	n.a.	BM
2	26,24	n.a.	6,071	3,804	1,11	n.a.	MB
**Total:**			632,740	342,435	100,00	0,000	

2 NIK-171-02 (250/1) flow 0,5

Sample Name:	**NIK-171-02 (250/1) flow 0,5**	*Injection Volume:*	**10,0**
Vial Number:	**BA3**	*Channel:*	**UV_VIS_1**
Sample Type:	**unknown**	*Wavelength:*	**220**
Control Program:	**YMC_90Min_95_5_flow0_5**	*Bandwidth:*	**4**
Quantif. Method:	**AD_H**	*Temperature/Column:*	**10**
Recording Time:	**11.10.2016 15:06**	*Flow ml/min:*	**0,500**
Run Time (min):	**45,66**	*Sample Amount:*	**1,0000**

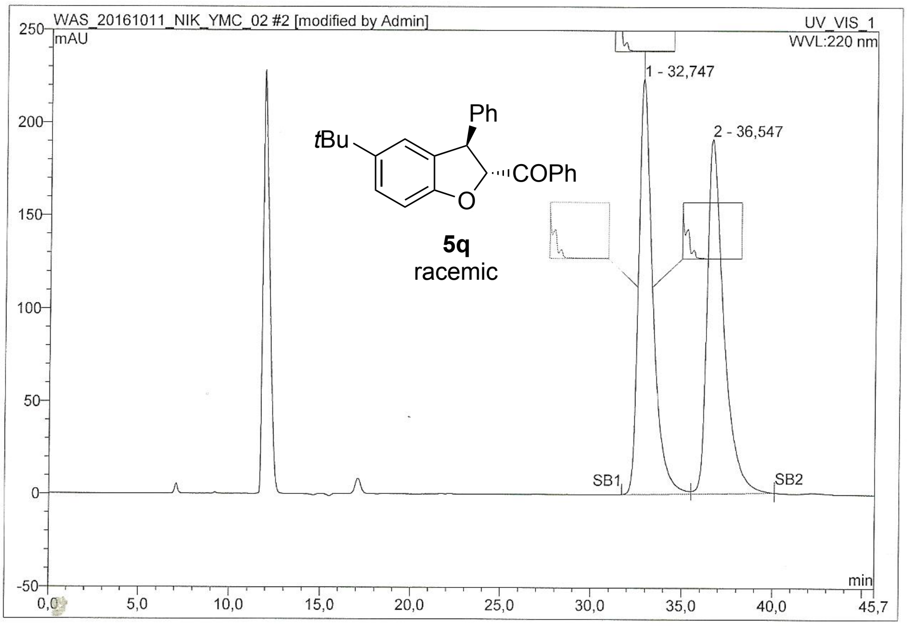

No.	Ret.Time min	Peak Name	Height mAU	Area mAU*min	Rel.Area %	Amount	Type
1	32,75	n.a.	223,450	202,189	49,89	n.a.	BM
2	36,55	n.a.	190,929	203,117	50,11	n.a.	MB
**Total:**			414,379	405,306	100,00	0,000	

4 NIK-172-02 (250/1) flow 0,5

Sample Name:	**NIK-172-02 (250/1) flow 0,5**	*Injection Volume:*	**10,0**
Vial Number:	**BA4**	*Channel:*	**UV_VIS_2**
Sample Type:	**unknown**	*Wavelength:*	**250**
Control Program:	**YMC_90Min_95_5_flow0_5**	*Bandwidth:*	**4**
Quantif. Method:	**AD_H**	*Temperature/Column:*	**10**
Recording Time:	**11.10.2016 17:11**	*Flow ml/min:*	**0,500**
Run Time (min):	**42,70**	*Sample Amount:*	**1,0000**

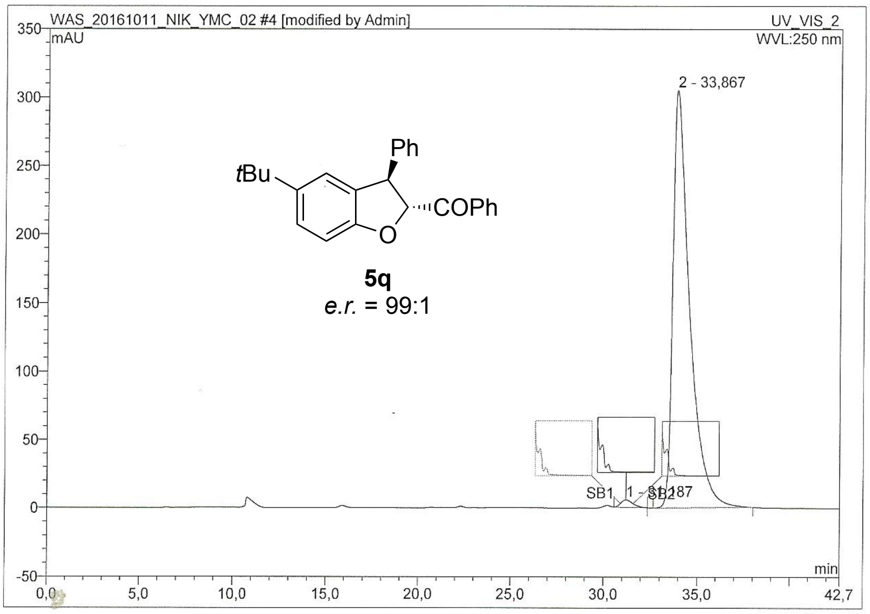

No.	Ret.Time min	Peak Name	Height mAU	Area mAU*min	Rel.Area %	Amount	Type
1	31,19	n.a.	5,489	4,068	1,27	n.a.	BMB
2	33,87	n.a.	304,804	316,491	98,73	n.a.	BMB
Total:			310,293	320,559	100,00	0,000	

6 NIK-197-02 (95/5) flow 0,5

Sample Name:	**NIK-197-02 (95/5) flow 0,5**	*Injection Volume:*	**10,0**
Vial Number:	**BD4**	*Channel:*	**UV_VIS_2**
Sample Type:	**unknown**	*Wavelength:*	**250**
Control Program:	**YMC_60Min_95_5_flow0_5**	*Bandwidth:*	**4**
Quantif. Method:	**AD_H**	*Temperature/Column:*	**10**
Recording Time:	**21.12.2016 14:16**	*Flow ml/min:*	**0,500**
Run Time (min):	**20,86**	*Sample Amount:*	**1,0000**

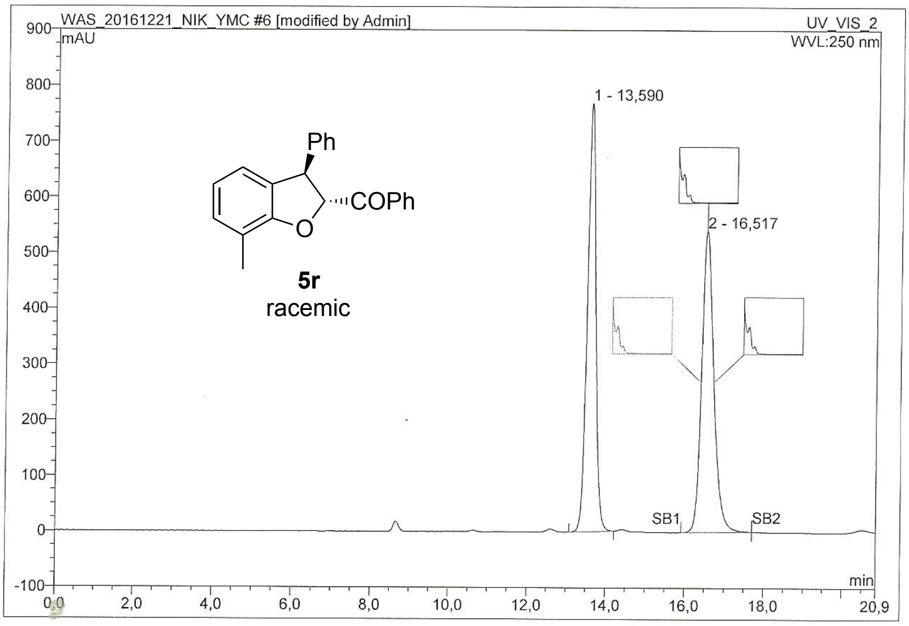

No.	Ret.Time min	Peak Name	Height mAU	Area mAU*min	Rel.Area %	Amount	Type
1	13,59	n.a.	768,366	191,662	49,98	n.a.	BMB*
2	16,52	n.a.	540,119	191,808	50,02	n.a.	BMB
**Total:**			1308,485	383,470	100,00	0,000	

7 NIK-196-02 (95/5) flow 0,5

Sample Name:	**NIK-196-02 (95/5) flow 0,5**	*Injection Volume:*	**10,0**
Vial Number:	**BE4**	*Channel:*	**UV_VIS_1**
Sample Type:	**unknown**	*Wavelength:*	**220**
Control Program:	**YMC_60Min_95_5_flow0_5**	*Bandwidth:*	**4**
Quantif. Method:	**AD_H**	*Temperature/Column:*	**10**
Recording Time:	**21.12.2016 14:37**	*Flow ml/min:*	**0,500**
Run Time (min):	**33,93**	*Sample Amount:*	**1,0000**

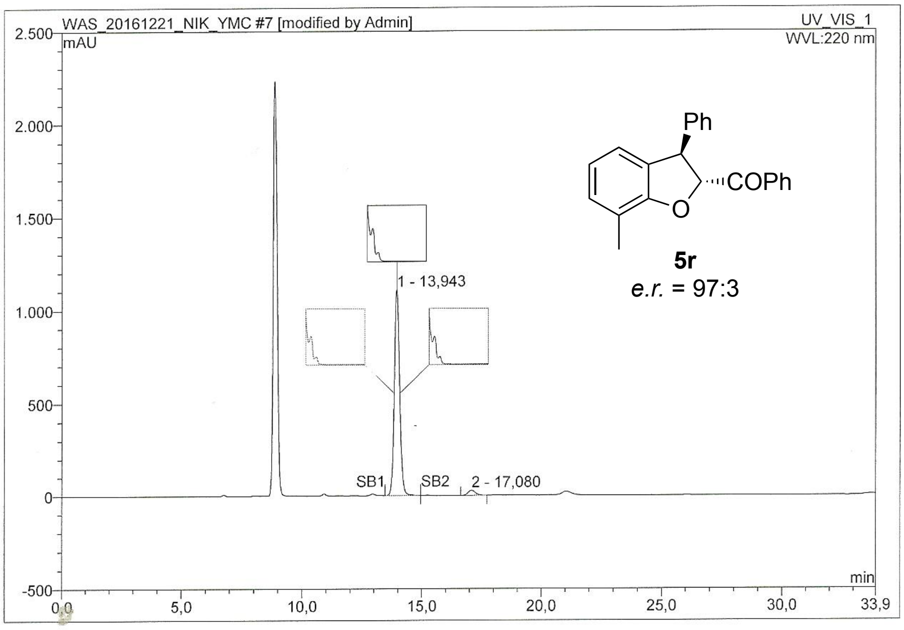

No.	Ret.Time min	Peak Name	Height mAU	Area mAU*min	Rel.Area %	Amount	Type
1	13,94	n.a.	1107,759	288,218	96,68	n.a.	BMB*
2	17,08	n.a.	28,202	9,890	3,32	n.a.	BMB*
Total:			1135,961	298,108	100,00	0,000	

2 NIK-191-02 (95/5) flow 0,5

Sample Name:	**NIK-191-02 (95/5) flow 0,5**	*Injection Volume:*	**10,0**
Vial Number:	**BD2**	*Channel:*	**UV_VIS_1**
Sample Type:	**unknown**	*Wavelength:*	**220**
Control Program:	**YMC_60Min_95_5_flow0_5**	*Bandwidth:*	**4**
Quantif. Method:	**AD_H**	*Temperature/Column:*	**10**
Recording Time:	**21.12.2016 12:03**	*Flow ml/min:*	**0,500**
Run Time (min):	**37,79**	*Sample Amount:*	**1,0000**

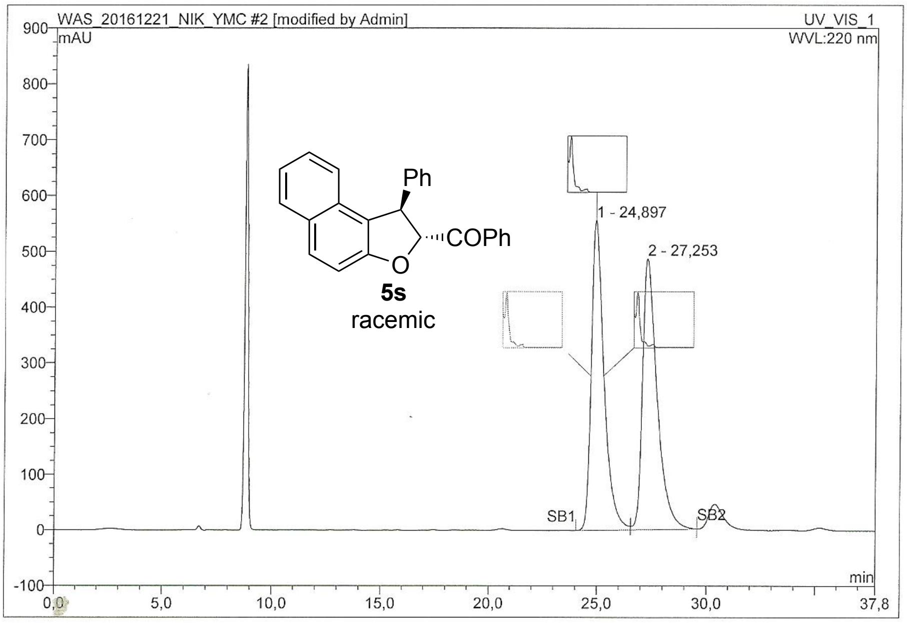

No.	Ret.Time min	Peak Name	Height mAU	Area mAU*min	Rel.Area %	Amount	Type
1	24,90	n.a.	556,539	375,300	49,98	n.a.	BM *
2	27,25	n.a.	486,941	375,635	50,02	n.a.	MB*
**Total:**			1043,480	750,936	100,00	0,000	

3 NIK-190-02 (95/5) flow 0,5

Sample Name:	**NIK-190-02 (95/5) flow 0,5**	*Injection Volume:*	**10,0**
Vial Number:	**BE2**	*Channel:*	**UV_VIS_2**
Sample Type:	**unknown**	*Wavelength:*	**250**
Control Program:	**YMC_60Min_95_5_flow0_5**	*Bandwidth:*	**4**
Quantif. Method:	**AD_H**	*Temperature/Column:*	**10**
Recording Time:	**21.12.2016 12:42**	*Flow ml/min:*	**0,500**
Run Time (min):	**37,46**	*Sample Amount:*	**1,0000**

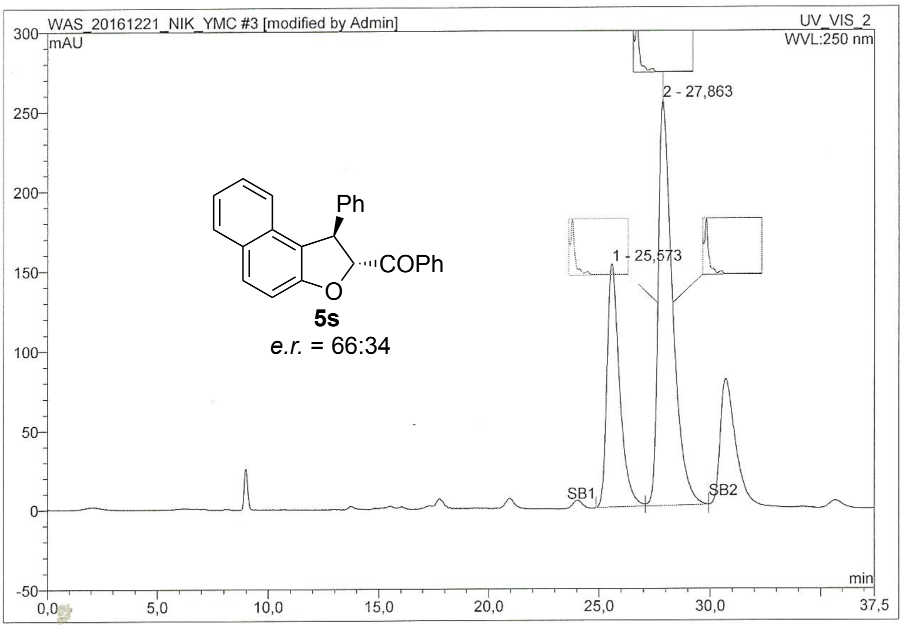

No.	Ret.Time min	Peak Name	Height mAU	Area mAU*min	Rel.Area %	Amount	Type
1	25,57	n.a.	152,557	100,848	34,29	n.a.	BM *
2	27,86	n.a.	254,579	193,233	65,71	n.a.	MB*
**Total:**			407,136	294,081	100,00	0,000	

4 NIK-201-02 (95/5) flow 0,5

Sample Name:	**NIK-201-02 (95/5) flow 0,5**	*Injection Volume:*	**10,0**
Vial Number:	**BD3**	*Channel:*	**UV_VIS_1**
Sample Type:	**unknown**	*Wavelength:*	**220**
Control Program:	**YMC_60Min_95_5_flow0_5**	*Bandwidth:*	**4**
Quantif. Method:	**AD_H**	*Temperature/Column:*	**10**
Recording Time:	**21.12.2016 13:20**	*Flow ml/min:*	**0,500**
Run Time (min):	**27,67**	*Sample Amount:*	**1,0000**

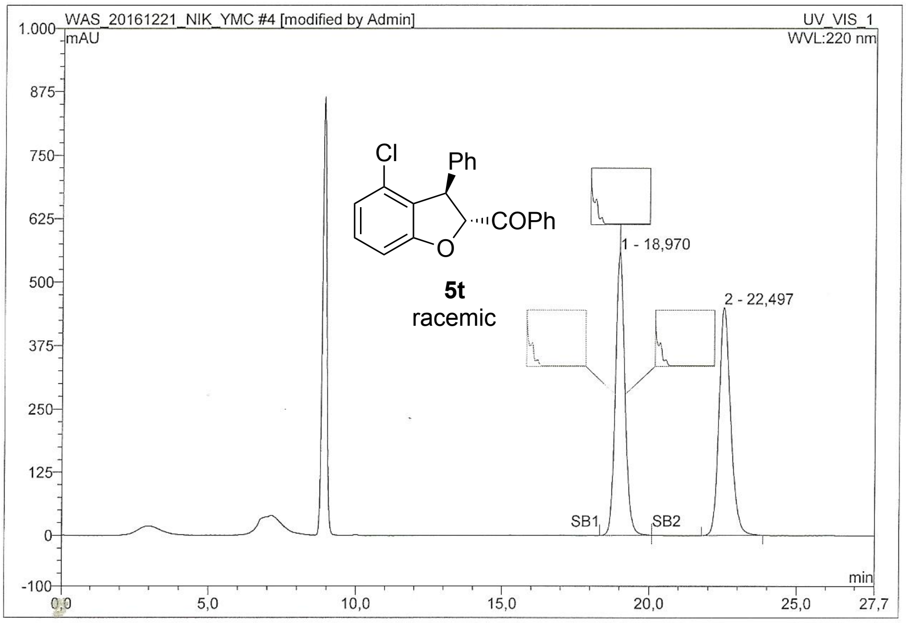

No.	Ret.Time min	Peak Name	Height mAU	Area mAU*min	Rel.Area %	Amount	Type
1	18,97	n.a.	558,663	207,683	50,06	n.a.	BMB
2	22,50	n.a.	451,122	207,203	49,94	n.a.	BMB
Total:			1009,785	414,887	100,00	0,000	

5 NIK-202-02 (95/5) flow 0,5

Sample Name:	**NIK-202-02 (95/5) flow 0,5**	*Injection Volume:*	**10,0**
Vial Number:	**BE3**	*Channel:*	**UV_VIS_2**
Sample Type:	**unknown**	*Wavelength:*	**250**
Control Program:	**YMC_60Min_95_5_flow0_5**	*Bandwidth:*	**4**
Quantif. Method:	**AD_H**	*Temperature/Column:*	**10**
Recording Time:	**21.12.2016 13:49**	*Flow ml/min:*	**0,500**
Run Time (min):	**25,98**	*Sample Amount:*	**1,0000**

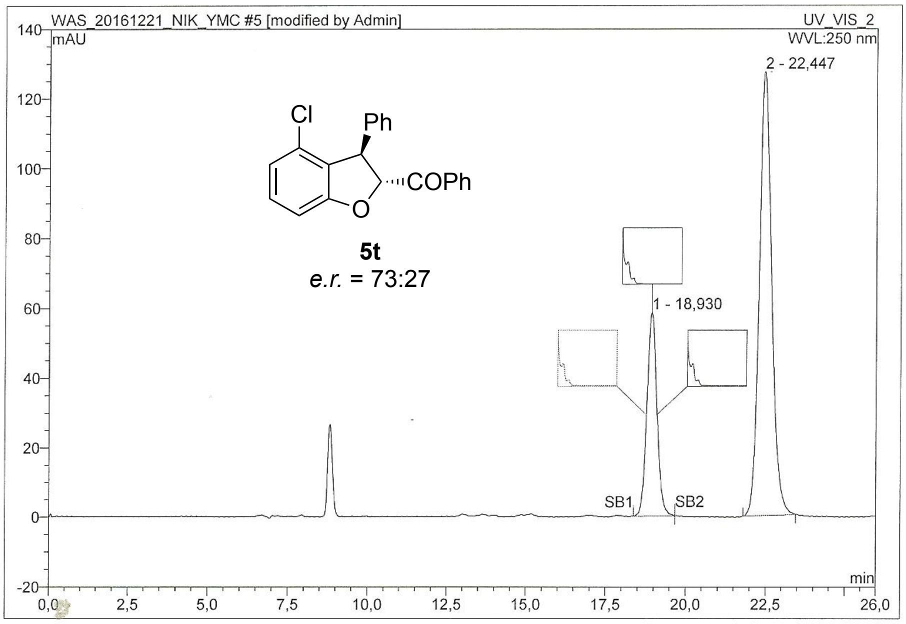

No.	Ret.Time min	Peak Name	Height mAU	Area mAU*min	Rel.Area %	Amount	Type
1	18,93	n.a.	58,515	21,224	26,99	n.a.	BMB
2	22,45	n.a.	127,182	57,415	73,01	n.a.	BMB
**Total:**			185,697	78,639	100,00	0,000	

3 ROI-1244-02_100_1_flo5

Sample Name:	**ROI-1244-02_100_1_flo5**	*Injection Volume:*	**10,0**
Vial Number:	**BD8**	*Channel:*	**UV_VIS_1**
Sample Type:	**unknown**	*Wavelength:*	**220**
Control Program:	**AD_H_90Min_100A_flow0_5**	*Bandwidth:*	**4**
Quantif. Method:	**default**	*Temperature/Column:*	**10**
Recording Time:	**31.1.2017 13:41**	*Flow ml/min:*	**0,500**
Run Time (min):	**60,14**	*Sample Amount:*	**1,0000**

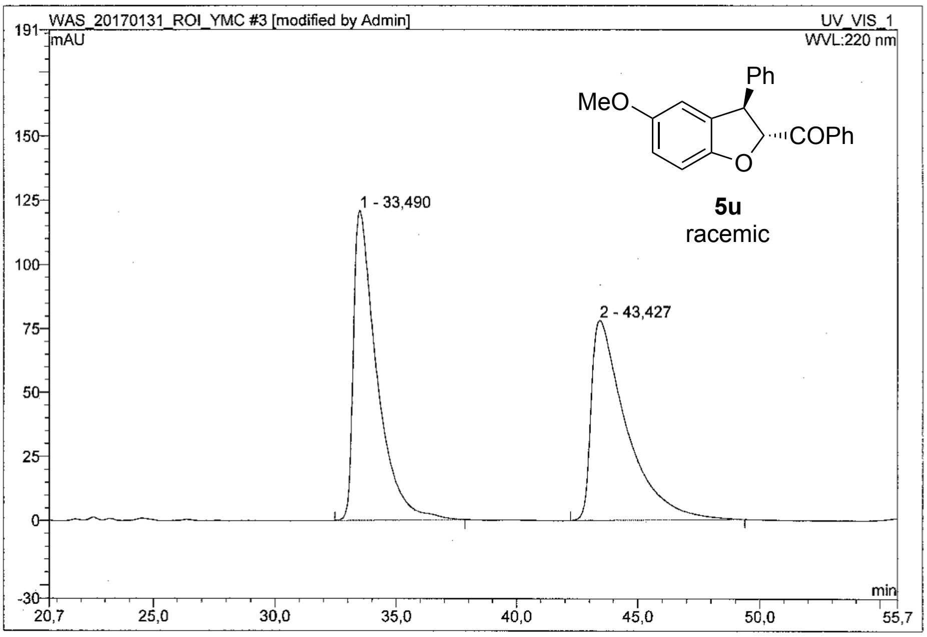

No.	Ret.Time min	Peak Name	Height mAU	Area mAU*min	Rel.Area %	Amount	Type
1	33,49	n.a.	120,784	134,800	50,52	n.a.	BMB
2	43,43	n.a.	78,120	132,009	49,48	n.a.	BMB*
Total:			198,905	266,809	100,00	0,000	

1 ROI-1243-02_100_1_flo5

Sample Name:	**ROI-1243-02_100_1_flo5**	*Injection Volume:*	**10,0**
Vial Number:	**BE8**	*Channel:*	**UV_VIS_1**
Sample Type:	**unknown**	*Wavelength:*	**220**
Control Program:	**AD_H_90Min_100A_flow0_5**	*Bandwidth:*	**4**
Quantif. Method:	**default**	*Temperature/Column:*	**10**
Recording Time:	**31.1.2017 15:25**	*Flow ml/min:*	**0,500**
Run Time (min):	**80,02**	*Sample Amount:*	**1,0000**

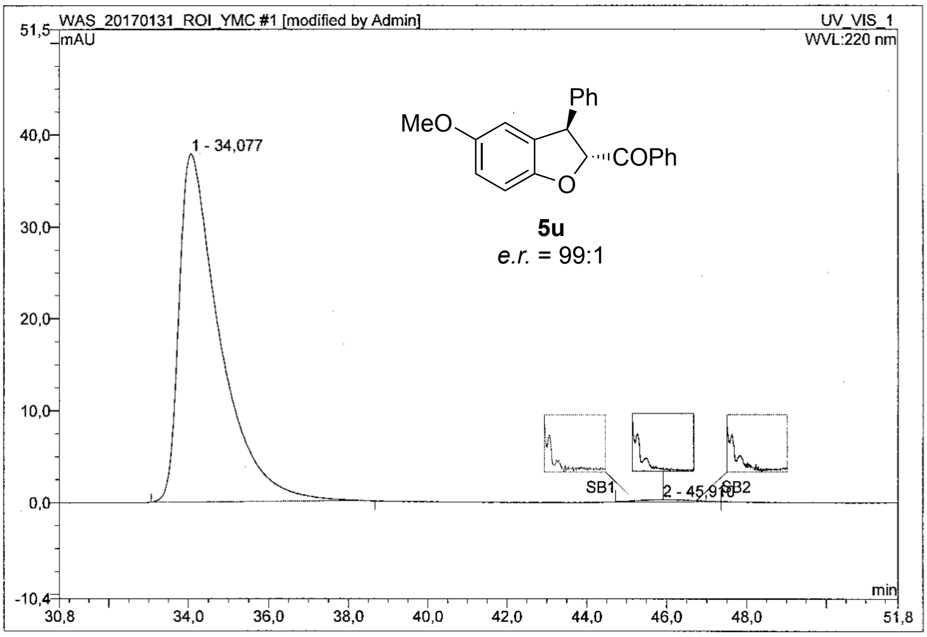

No.	Ret.Time min	Peak Name	Height mAU	Area mAU*min	Rel.Area %	Amount	Type
1	34,08	n.a.	37,865	44,488	99,24	n.a.	BMB*
2	45,91	n.a.	0,228	0,339	0,76	n.a.	BMB*
**Total:**			38,094	44,827	100,00	0,000	

2 ROI-1236-02_100_1_flo5

Sample Name:	**ROI-1236-02_100_1_flo5**	*Injection Volume:*	**10,0**
Vial Number:	**BD7**	*Channel:*	**UV_VIS_1**
Sample Type:	**unknown**	*Wavelength:*	**220**
Control Program:	**AD_H_90Min_100A_flow0_5**	*Bandwidth:*	**4**
Quantif. Method:	**default**	*Temperature/Column:*	**10**
Recording Time:	**31.1.2017 12:24**	*Flow ml/min:*	**0,500**
Run Time (min):	**76,50**	*Sample Amount:*	**1,0000**

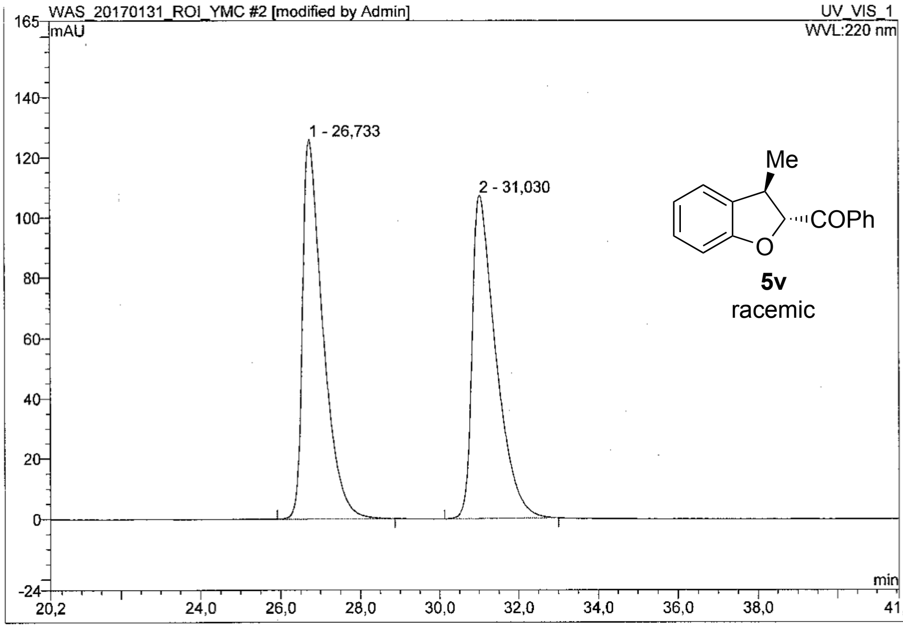

No.	Ret.Time min	Peak Name	Height mAU	Area mAU*min	Rel.Area %	Amount	Type
1	26,73	n.a.	125,901	74,849	50,09	n.a.	BMB
2	31,03	n.a.	107,107	74,583	49,91	n.a.	BMB
Total:			233,007	149,432	100,00	0,000	

1 ROI-1235-02_100_1_flo5

Sample Name:	**ROI-1235-02_100_1_flo5**	*Injection Volume:*	**10,0**
Vial Number:	**BE7**	*Channel:*	**UV_VIS_1**
Sample Type:	**unknown**	*Wavelength:*	**220**
Control Program:	**AD_H_60Min_100A_flow0_5**	*Bandwidth:*	**4**
Quantif. Method:	**default**	*Temperature/Column:*	**10**
Recording Time:	**31.1.2017 14:44**	*Flow ml/min:*	**0,500**
Run Time (min):	**36,66**	*Sample Amount:*	**1,0000**

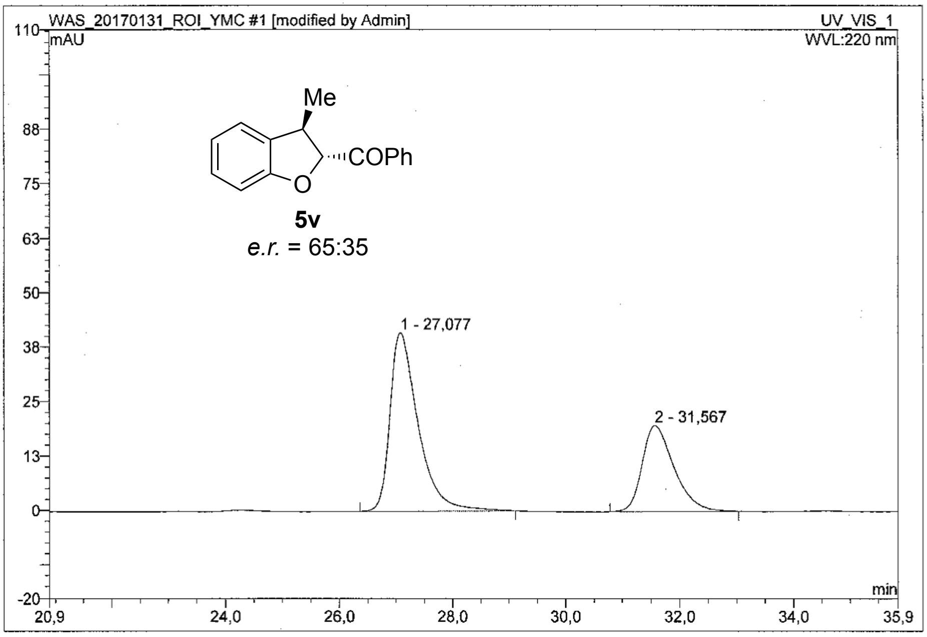

No.	Ret.Time min	Peak Name	Height mAU	Area mAU*min	Rel.Area %	Amount	Type
1	27,08	n.a.	40,979	23,748	64,99	n.a.	BMB
2	31,57	n.a.	19,773	12,793	35,01	n.a.	BMB
**Total:**			60,753	36,541	100,00	0,000	

5. Copies of NMR-Spectra of new Compounds

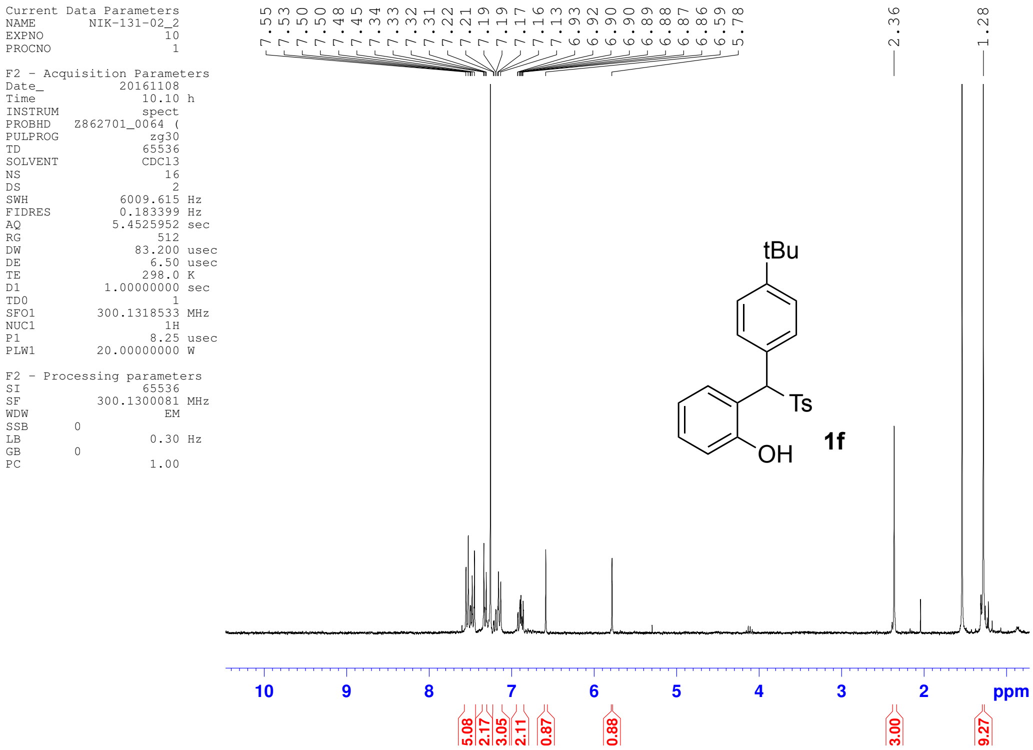

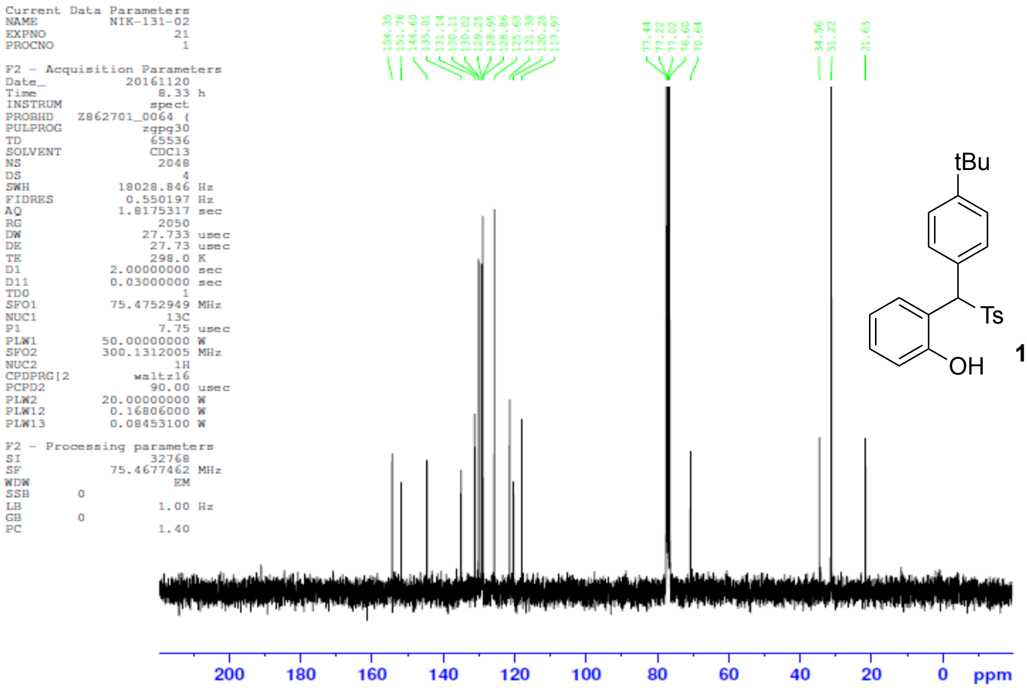

```
Current Data Parameters
NAME          NIK-203-02
EXPNO                 40
PROCNO                 1

F2 - Acquisition Parameters
Date_           20170110
Time               18.22 h
INSTRUM            spect
PROBHD   Z862701_0064 (
PULPROG             zg30
TD                 65536
SOLVENT            CDCl3
NS                    16
DS                     2
SWH             6009.615 Hz
FIDRES          0.183399 Hz
AQ             5.4525952 sec
RG                   575
DW                83.200 usec
DE                  6.50 usec
TE                 298.0 K
D1            1.00000000 sec
TD0                    1
SFO1         300.1318533 MHz
NUC1                  1H
P1                  8.25 usec
PLW1         20.00000000 W

F2 - Processing parameters
SI                 65536
SF           300.1300109 MHz
WDW                   EM
SSB      0
LB                  0.30 Hz
GB       0
PC                  1.00
```

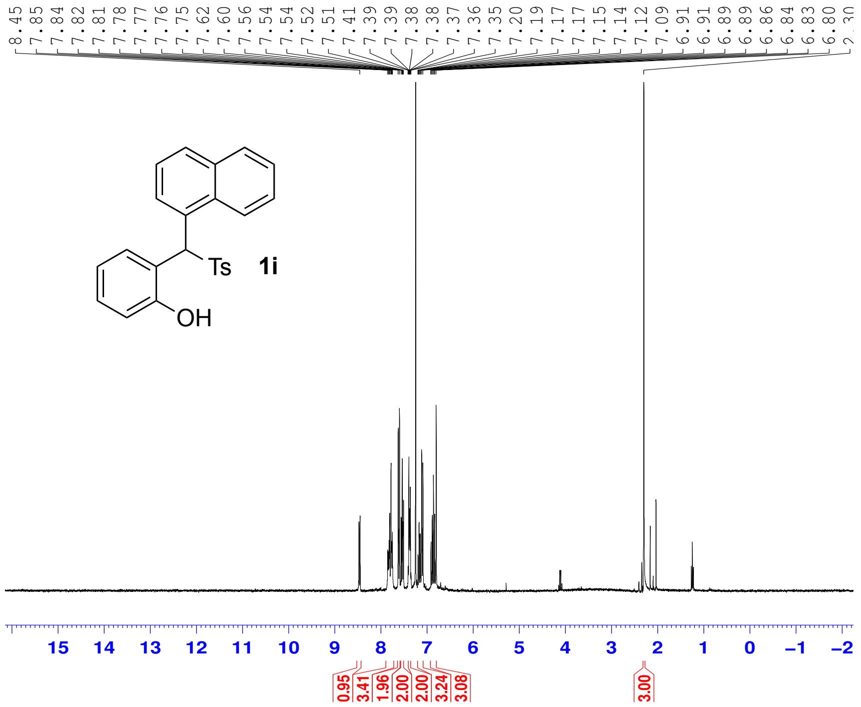


```
Current Data Parameters
NAME          NIK-203-02
EXPNO                 51
PROCNO                 1

F2 - Acquisition Parameters
Date_           20170114
Time               10.33 h
INSTRUM            spect
PROBHD   Z862701_0064 (
PULPROG           zgpg30
TD                 65536
SOLVENT            CDCl3
NS                  2048
DS                     4
SWH            18028.846 Hz
FIDRES          0.550197 Hz
AQ             1.8175317 sec
RG                  2050
DW                27.733 usec
DE                 27.73 usec
TE                 298.0 K
D1            4.00000000 sec
D11           0.03000000 sec
TD0                    1
SFO1          75.4752949 MHz
NUC1                 13C
P1                  7.75 usec
PLW1         50.00000000 W
SFO2         300.1312005 MHz
NUC2                  1H
CPDPRG[2         waltz16
PCPD2              90.00 usec
PLW2         20.00000000 W
PLW12         0.16806000 W
PLW13         0.08453100 W

F2 - Processing parameters
SI                 32768
SF            75.4677486 MHz
WDW                   EM
SSB      0
LB                  1.00 Hz
GB       0
PC                  1.40
```

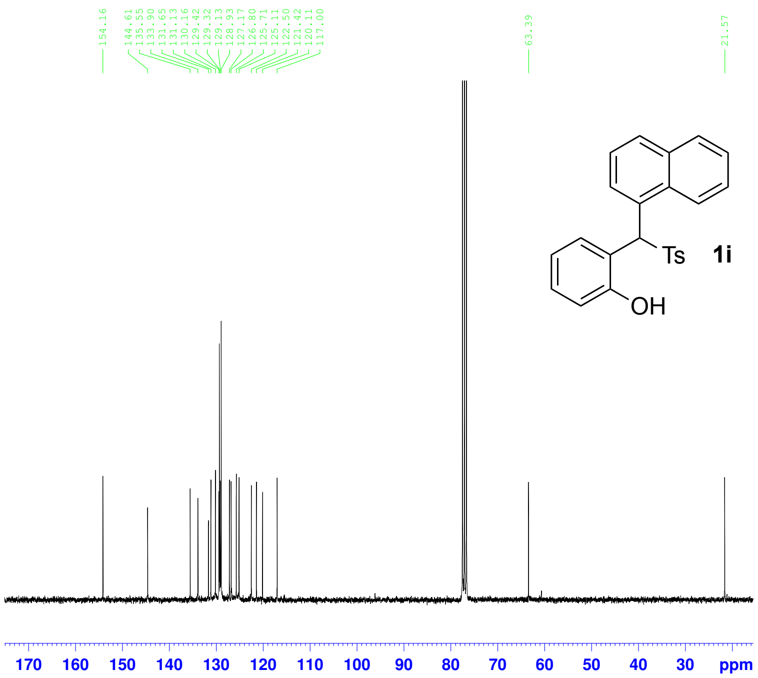

Current Data Parameters
NAME NIK-185-02
EXPNO 20
PROCNO 1

F2 - Acquisition Parameters
Date_ 20161108
Time 10.14 h
INSTRUM spect
PROBHD Z862701_0064 (
PULPROG zg30
TD 65536
SOLVENT CDCl3
NS 16
DS 2
SWH 6009.615 Hz
FIDRES 0.183399 Hz
AQ 5.4525952 sec
RG 456
DW 83.200 usec
DE 6.50 usec
TE 298.0 K
D1 1.00000000 sec
TD0 1
SFO1 300.1318533 MHz
NUC1 1H
P1 8.25 usec
PLW1 20.00000000 W

F2 - Processing parameters
SI 65536
SF 300.1300087 MHz
WDW EM
SSB 0
LB 0.30 Hz
GB 0
PC 1.00

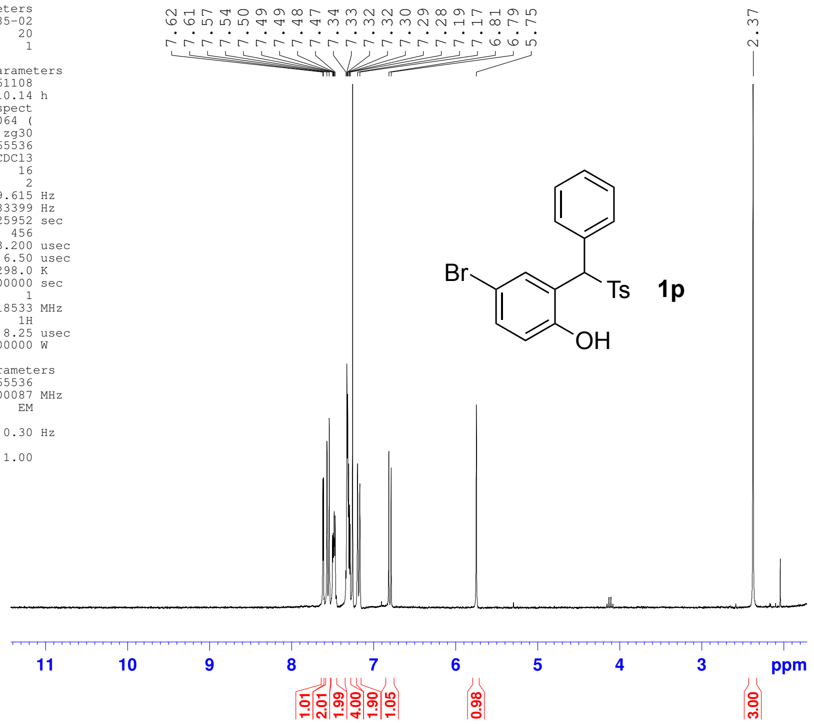

Current Data Parameters
NAME NIK-185-02
EXPNO 31
PROCNO 1

F2 - Acquisition Parameters
Date_ 20161118
Time 2.16 h
INSTRUM spect
PROBHD Z862701_0064 (
PULPROG zgpg30
TD 65536
SOLVENT CDCl3
NS 2048
DS 4
SWH 18028.846 Hz
FIDRES 0.550197 Hz
AQ 1.8175317 sec
RG 2050
DW 27.733 usec
DE 27.73 usec
TE 297.8 K
D1 2.00000000 sec
D11 0.03000000 sec
TD0 1
SFO1 75.4752949 MHz
NUC1 13C
P1 7.75 usec
PLW1 50.00000000 W
SFO2 300.1312005 MHz
NUC2 1H
CPDPRG[2 waltz16
PCPD2 90.00 usec
PLW2 20.00000000 W
PLW12 0.16806000 W
PLW13 0.08453100 W

F2 - Processing parameters
SI 32768
SF 75.4677461 MHz
WDW EM
SSB 0
LB 1.00 Hz
GB 0
PC 1.40

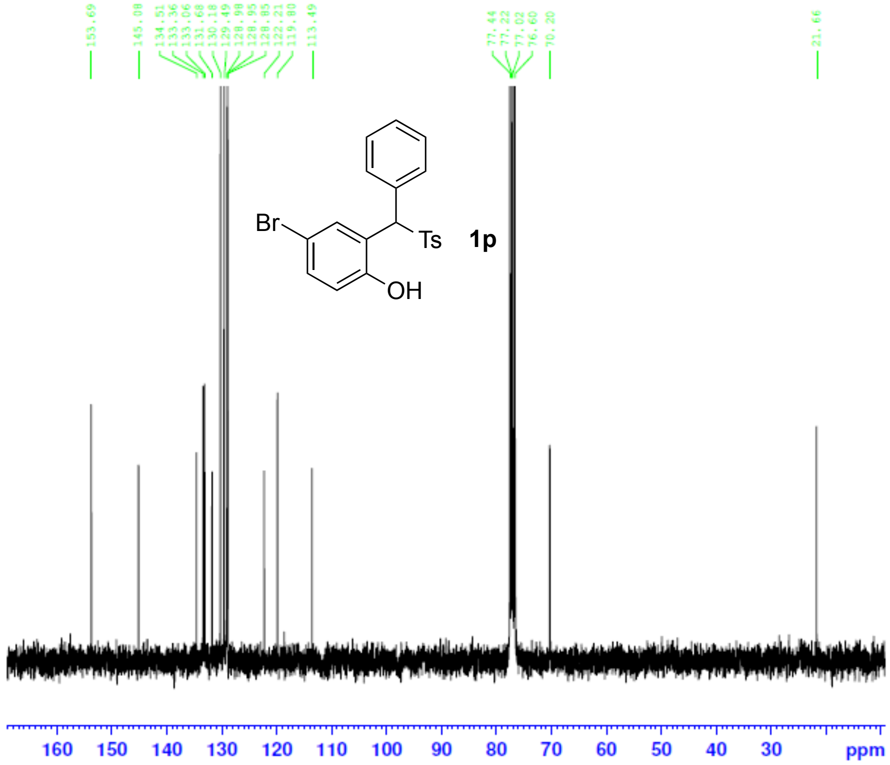

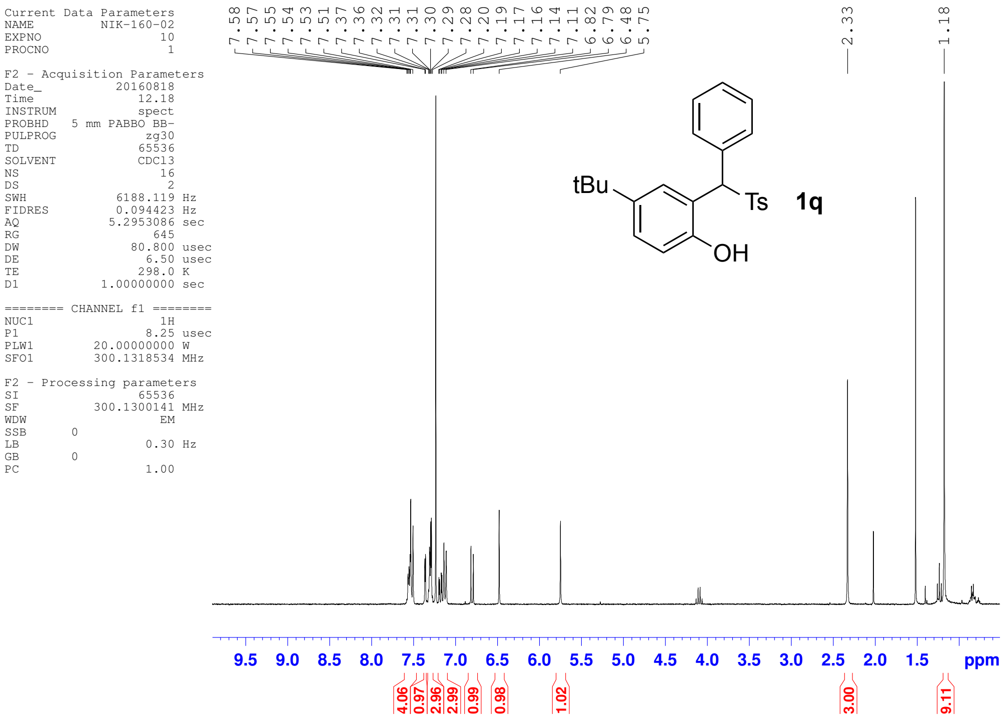
Current Data Parameters
NAME NIK-160-02
EXPNO 10
PROCNO 1
F2 - Acquisition Parameters
Date_ 20160818
Time 12.18
INSTRUM spect
PROBHD 5 mm PABBO BB-
PULPROG zg30
TD 65536
SOLVENT CDCl3
NS 16
DS 2
SWH 6188.119 Hz
FIDRES 0.094423 Hz
AQ 5.2953086 sec
RG 645
DW 80.800 usec
DE 6.50 usec
TE 298.0 K
D1 1.00000000 sec
======== CHANNEL f1 ========
NUC1 1H
P1 8.25 usec
PLW1 20.00000000 W
SFO1 300.1318534 MHz
F2 - Processing parameters
SI 65536
SF 300.1300141 MHz
WDW EM
SSB 0
LB 0.30 Hz
GB 0
PC 1.00
7.58 7.57 7.55 7.54 7.53 7.51 7.37 7.36 7.32 7.31 7.31 7.30 7.29 7.28 7.20 7.19 7.17 7.16 7.14 7.11 6.82 6.79 6.48 5.75
2.33
1.18
tBu
Ts
1q
OH
9.5 9.0 8.5 8.0 7.5 7.0 6.5 6.0 5.5 5.0 4.5 4.0 3.5 3.0 2.5 2.0 1.5 ppm
4.06 0.97 2.96 2.99 0.99 0.98 1.02 3.00 9.11

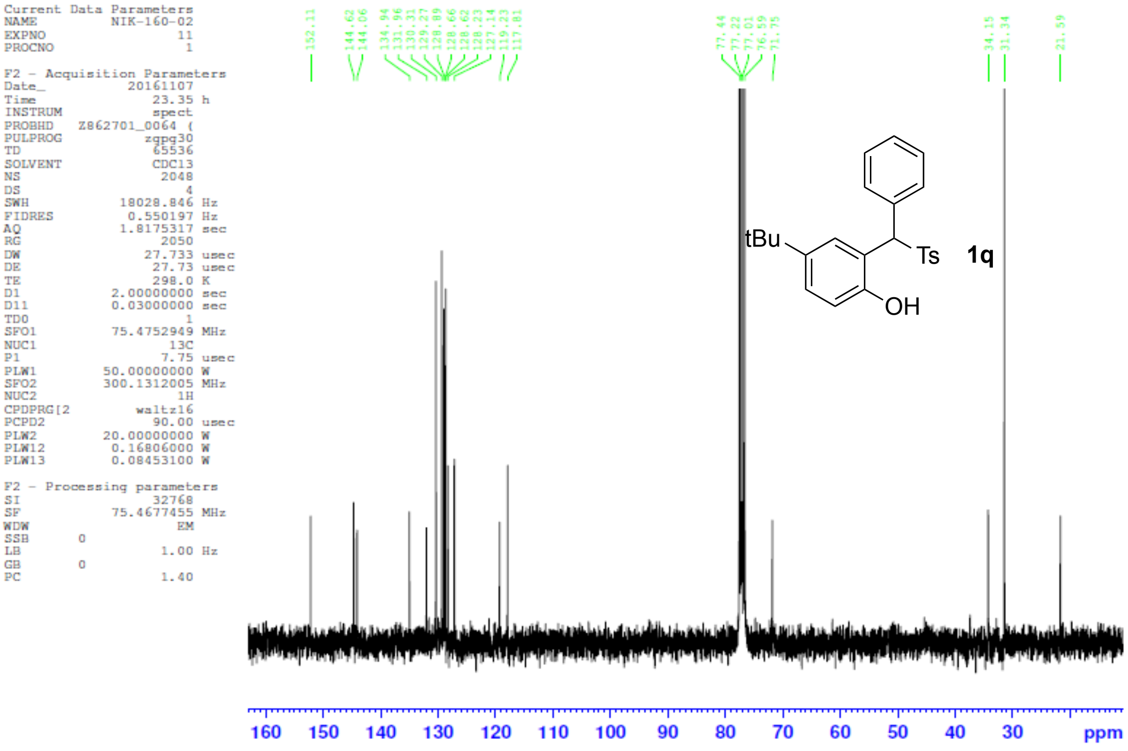
Current Data Parameters
NAME NIK-160-02
EXPNO 11
PROCNO 1
F2 - Acquisition Parameters
Date_ 20161107
Time 23.35 h
INSTRUM spect
PROBHD Z862701_0064 (
PULPROG zgpg30
TD 65536
SOLVENT CDCl3
NS 2048
DS 4
SWH 18028.846 Hz
FIDRES 0.550197 Hz
AQ 1.8175317 sec
RG 2050
DW 27.733 usec
DE 27.73 usec
TE 298.0 K
D1 2.00000000 sec
D11 0.03000000 sec
TD0 1
SFO1 75.4752949 MHz
NUC1 13C
P1 7.75 usec
PLW1 50.00000000 W
SFO2 300.1312005 MHz
NUC2 1H
CPDPRG[2 waltz16
PCPD2 90.00 usec
PLW2 20.00000000 W
PLW12 0.16806000 W
PLW13 0.08453100 W
F2 - Processing parameters
SI 32768
SF 75.4677455 MHz
WDW EM
SSB 0
LB 1.00 Hz
GB 0
PC 1.40
152.11
144.62 144.06
134.94 131.96 130.31 129.27 128.89 128.66 128.62 128.23 127.14 119.23 117.81
77.44 77.22 77.01 76.59 71.75
34.15 31.34
21.59
tBu
Ts
1q
OH
160 150 140 130 120 110 100 90 80 70 60 50 40 30 ppm

Current Data Parameters
NAME NIK-195-02
EXPNO 10
PROCNO 1

F2 - Acquisition Parameters
Date_ 20161020
Time 13.42 h
INSTRUM spect
PROBHD Z862701_0064 (
PULPROG zg30
TD 65536
SOLVENT CDCl3
NS 16
DS 2
SWH 6009.615 Hz
FIDRES 0.183399 Hz
AQ 5.4525952 sec
RG 575
DW 83.200 usec
DE 6.50 usec
TE 297.9 K
D1 1.00000000 sec
TD0 1
SFO1 300.1318533 MHz
NUC1 1H
P1 8.25 usec
PLW1 20.00000000 W

F2 - Processing parameters
SI 65536
SF 300.1300073 MHz
WDW EM
SSB 0
LB 0.30 Hz
GB 0
PC 1.00

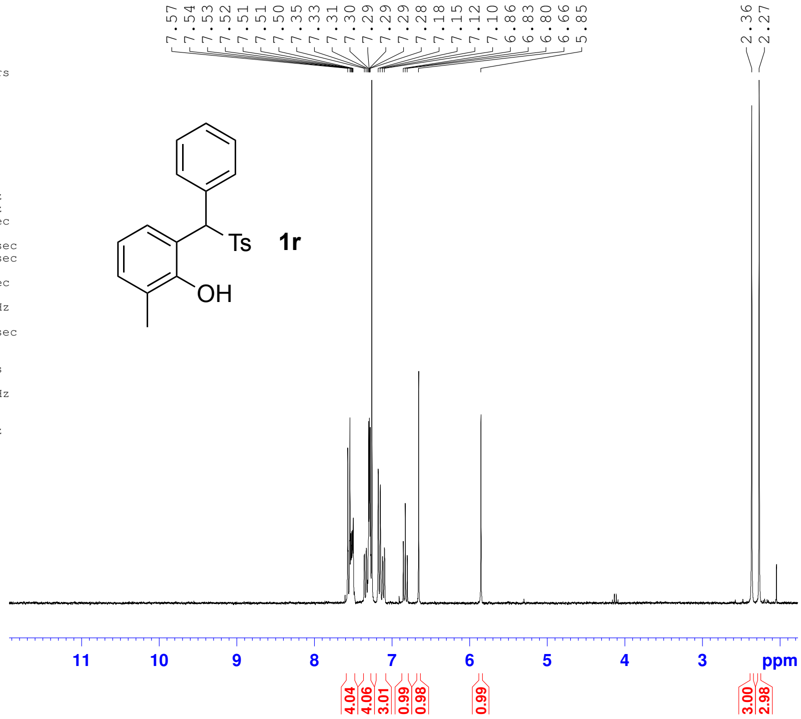

Current Data Parameters
NAME NIK-195-02
EXPNO 21
PROCNO 1

F2 - Acquisition Parameters
Date_ 20161030
Time 6.49 h
INSTRUM spect
PROBHD Z862701_0064 (
PULPROG zgpg30
TD 65536
SOLVENT CDCl3
NS 4096
DS 4
SWH 18028.846 Hz
FIDRES 0.550197 Hz
AQ 1.8175317 sec
RG 2050
DW 27.733 usec
DE 27.73 usec
TE 298.0 K
D1 2.00000000 sec
D11 0.03000000 sec
TD0 1
SFO1 75.4752949 MHz
NUC1 13C
P1 7.75 usec
PLW1 50.00000000 W
SFO2 300.1312005 MHz
NUC2 1H
CPDPRG[2 waltz16
PCPD2 90.00 usec
PLW2 20.00000000 W
PLW12 0.16806000 W
PLW13 0.08453100 W

F2 - Processing parameters
SI 32768
SF 75.4677459 MHz
WDW EM
SSB 0
LB 1.00 Hz
GB 0
PC 1.40

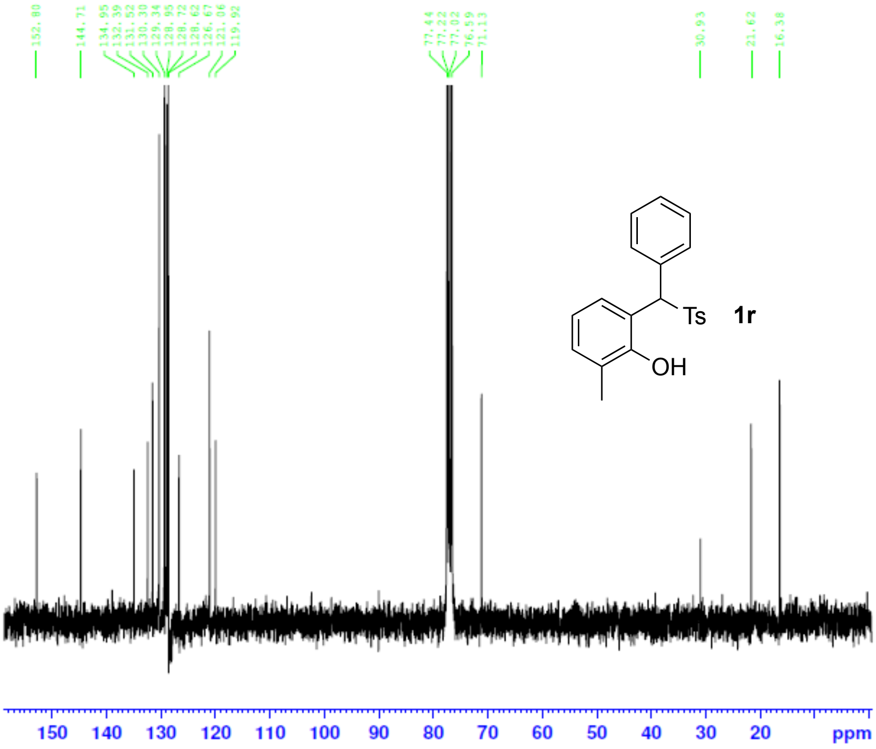

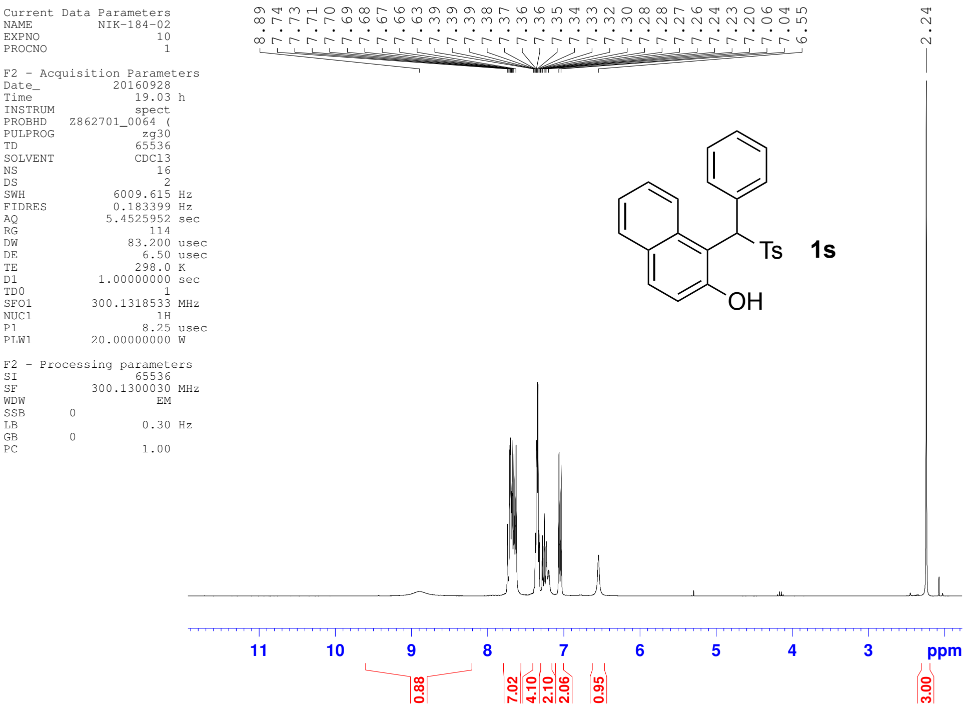
Current Data Parameters
NAME NIK-184-02
EXPNO 10
PROCNO 1
F2 - Acquisition Parameters
Date_ 20160928
Time 19.03 h
INSTRUM spect
PROBHD Z862701_0064 (
PULPROG zg30
TD 65536
SOLVENT CDCl3
NS 16
DS 2
SWH 6009.615 Hz
FIDRES 0.183399 Hz
AQ 5.4525952 sec
RG 114
DW 83.200 usec
DE 6.50 usec
TE 298.0 K
D1 1.00000000 sec
TD0 1
SFO1 300.1318533 MHz
NUC1 1H
P1 8.25 usec
PLW1 20.00000000 W
F2 - Processing parameters
SI 65536
SF 300.1300030 MHz
WDW EM
SSB 0
LB 0.30 Hz
GB 0
PC 1.00
8.89 7.74 7.73 7.71 7.70 7.69 7.68 7.67 7.66 7.63 7.39 7.39 7.39 7.38 7.37 7.36 7.36 7.35 7.34 7.33 7.32 7.30 7.28 7.28 7.27 7.26 7.24 7.23 7.20 7.06 7.04 6.55
2.24
Ts
OH
1s
11 10 9 8 7 6 5 4 3 ppm
0.88 7.02 4.10 2.10 2.06 0.95 3.00

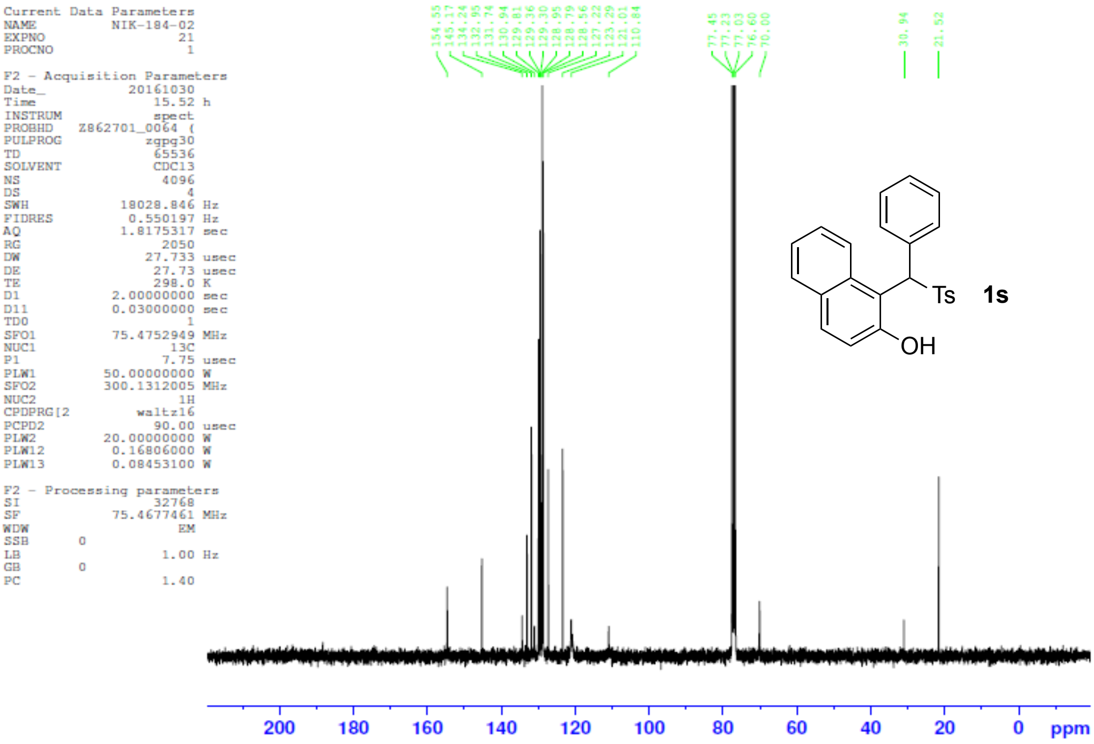
Current Data Parameters
NAME NIK-184-02
EXPNO 21
PROCNO 1
F2 - Acquisition Parameters
Date_ 20161030
Time 15.52 h
INSTRUM spect
PROBHD Z862701_0064 (
PULPROG zgpg30
TD 65536
SOLVENT CDCl3
NS 4096
DS 4
SWH 18028.846 Hz
FIDRES 0.550197 Hz
AQ 1.8175317 sec
RG 2050
DW 27.733 usec
DE 27.73 usec
TE 298.0 K
D1 2.00000000 sec
D11 0.03000000 sec
TD0 1
SFO1 75.4752949 MHz
NUC1 13C
P1 7.75 usec
PLW1 50.00000000 W
SFO2 300.1312005 MHz
NUC2 1H
CPDPRG[2 waltz16
PCPD2 90.00 usec
PLW2 20.00000000 W
PLW12 0.16806000 W
PLW13 0.08453100 W
F2 - Processing parameters
SI 32768
SF 75.4677461 MHz
WDW EM
SSB 0
LB 1.00 Hz
GB 0
PC 1.40
154.55 145.17 134.24 132.95 131.74 130.94 129.81 129.36 129.30 128.95 128.79 128.56 127.22 123.29 121.01 110.84
77.45 77.23 77.03 76.60 70.80
30.94
21.52
Ts
OH
1s
200 180 160 140 120 100 80 60 40 20 0 ppm

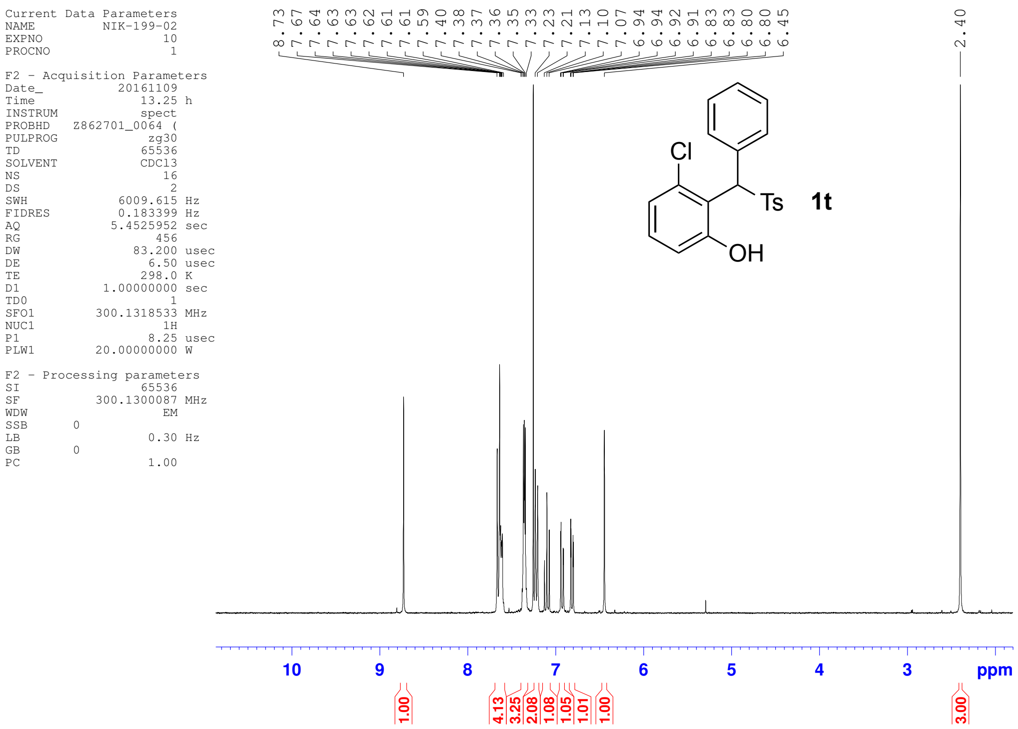
Current Data Parameters
NAME NIK-199-02
EXPNO 10
PROCNO 1
F2 - Acquisition Parameters
Date_ 20161109
Time 13.25 h
INSTRUM spect
PROBHD Z862701_0064 (
PULPROG zg30
TD 65536
SOLVENT CDCl3
NS 16
DS 2
SWH 6009.615 Hz
FIDRES 0.183399 Hz
AQ 5.4525952 sec
RG 456
DW 83.200 usec
DE 6.50 usec
TE 298.0 K
D1 1.00000000 sec
TD0 1
SFO1 300.1318533 MHz
NUC1 1H
P1 8.25 usec
PLW1 20.00000000 W
F2 - Processing parameters
SI 65536
SF 300.1300087 MHz
WDW EM
SSB 0
LB 0.30 Hz
GB 0
PC 1.00
8.73 7.67 7.64 7.63 7.63 7.62 7.61 7.61 7.59 7.40 7.38 7.37 7.36 7.35 7.33 7.23 7.21 7.13 7.10 7.07 6.94 6.94 6.92 6.91 6.83 6.83 6.80 6.80 6.45
2.40
Cl
Ts
1t
OH
1.00 4.13 3.25 2.08 1.08 1.05 1.01 1.00 3.00
10 9 8 7 6 5 4 3 ppm

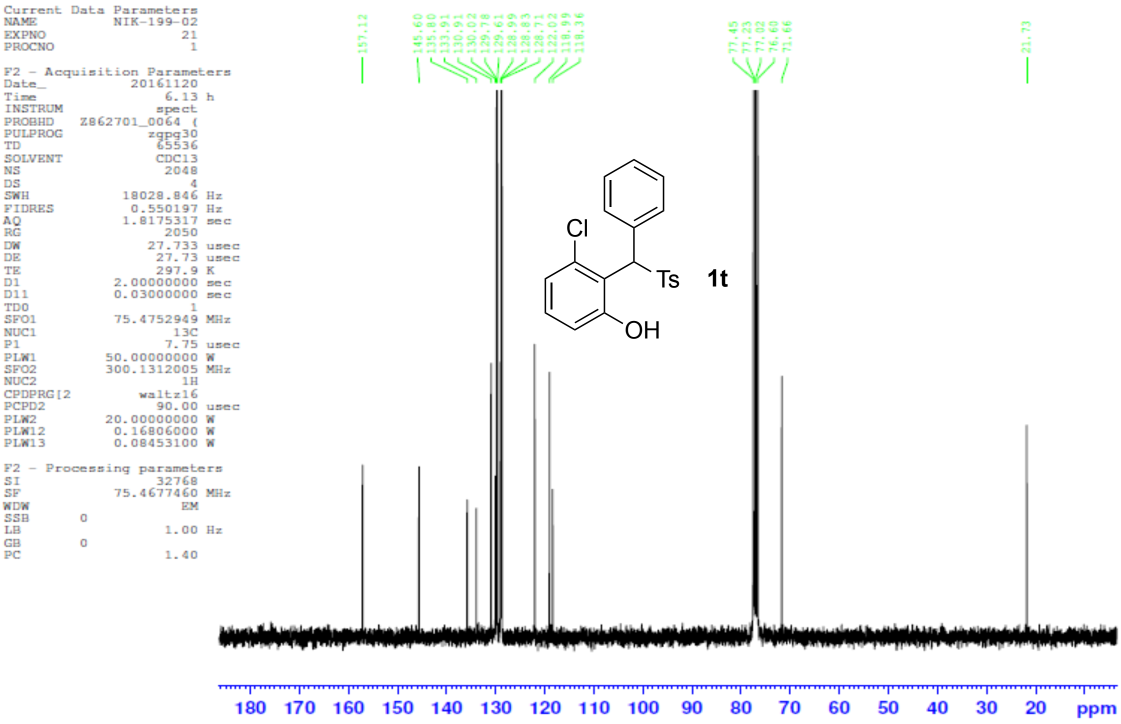
Current Data Parameters
NAME NIK-199-02
EXPNO 21
PROCNO 1
F2 - Acquisition Parameters
Date_ 20161120
Time 6.13 h
INSTRUM spect
PROBHD Z862701_0064 (
PULPROG zgpg30
TD 65536
SOLVENT CDCl3
NS 2048
DS 4
SWH 18028.846 Hz
FIDRES 0.550197 Hz
AQ 1.8175317 sec
RG 2050
DW 27.733 usec
DE 27.73 usec
TE 297.9 K
D1 2.00000000 sec
D11 0.03000000 sec
TD0 1
SFO1 75.4752949 MHz
NUC1 13C
P1 7.75 usec
PLW1 50.00000000 W
SFO2 300.1312005 MHz
NUC2 1H
CPDPRG[2 waltz16
PCPD2 90.00 usec
PLW2 20.00000000 W
PLW12 0.16806000 W
PLW13 0.08453100 W
F2 - Processing parameters
SI 32768
SF 75.4677460 MHz
WDW EM
SSB 0
LB 1.00 Hz
GB 0
PC 1.40
157.12
145.60 135.80 133.91 130.91 130.02 129.78 129.61 128.99 128.83 128.71 122.02 118.99 118.36
77.45 77.23 77.02 76.60 71.66
21.73
Cl
Ts
1t
OH
180 170 160 150 140 130 120 110 100 90 80 70 60 50 40 30 20 ppm

```
Current Data Parameters
NAME          NIK-198-01
EXPNO                  1
PROCNO                 1

F2 - Acquisition Parameters
Date_           20161024
Time               12.20 h
INSTRUM            spect
PROBHD   Z126715_0001 (
PULPROG             zg30
TD                 65536
SOLVENT            CDCl3
NS                     4
DS                     2
SWH             9803.922 Hz
FIDRES          0.299192 Hz
AQ             3.3423359 sec
RG                  28.5
DW                51.000 usec
DE                 18.00 usec
TE                 298.0 K
D1            2.00000000 sec
TD0                    1
SFO1         700.3335017 MHz
NUC1                  1H
P1                  8.90 usec
PLW1          8.69999981 W

F2 - Processing parameters
SI                 65536
SF           700.3300185 MHz
WDW                   EM
SSB      0
LB                  0.20 Hz
GB       0
PC                  1.00
```

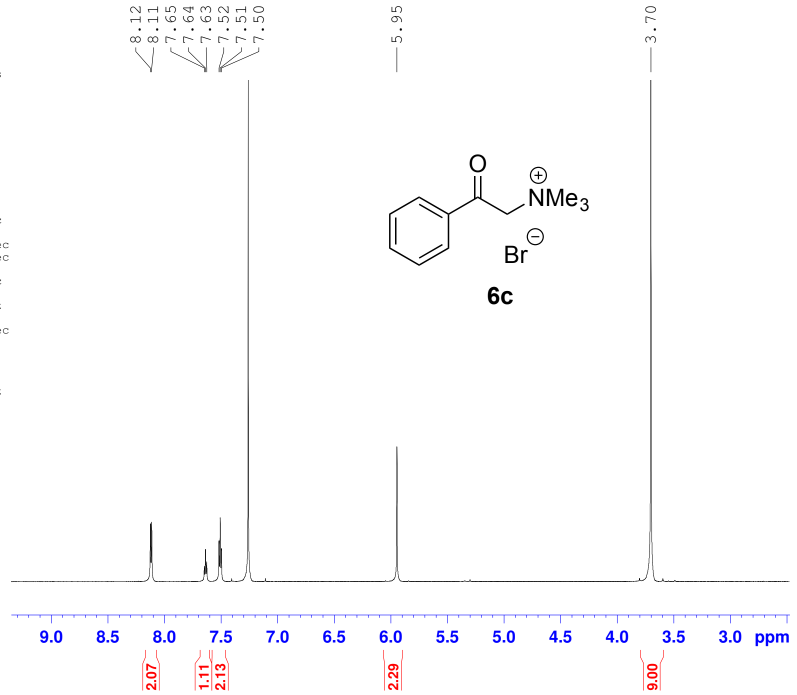


```
Current Data Parameters
NAME          NIK-198-01
EXPNO                  2
PROCNO                 1

F2 - Acquisition Parameters
Date_           20161024
Time               13.11 h
INSTRUM            spect
PROBHD   Z126715_0001 (
PULPROG           zgpg30
TD                 65536
SOLVENT            CDCl3
NS                  1023
DS                     4
SWH            40760.871 Hz
FIDRES          1.243923 Hz
AQ             0.8039083 sec
RG                  2050
DW                12.267 usec
DE                 18.00 usec
TE                 298.0 K
D1            2.00000000 sec
D11           0.03000000 sec
TD0                    1
SFO1         176.1183703 MHz
NUC1                 13C
P1                 12.00 usec
PLW1        129.00000000 W
SFO2         700.3328013 MHz
NUC2                  1H
CPDPRG[2         waltz16
PCPD2              65.00 usec
PLW2          8.69999981 W
PLW12         0.16311000 W
PLW13         0.08213100 W

F2 - Processing parameters
SI                 65536
SF           176.0980943 MHz
WDW                   EM
SSB      0
LB                  4.00 Hz
GB       0
PC                  1.40
```

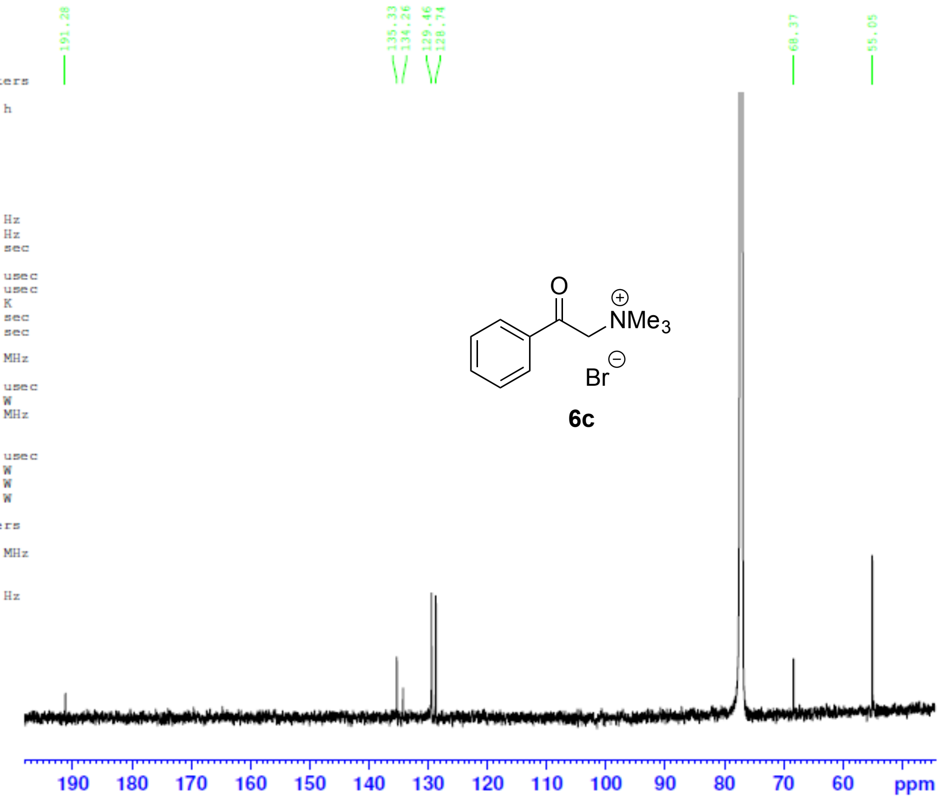

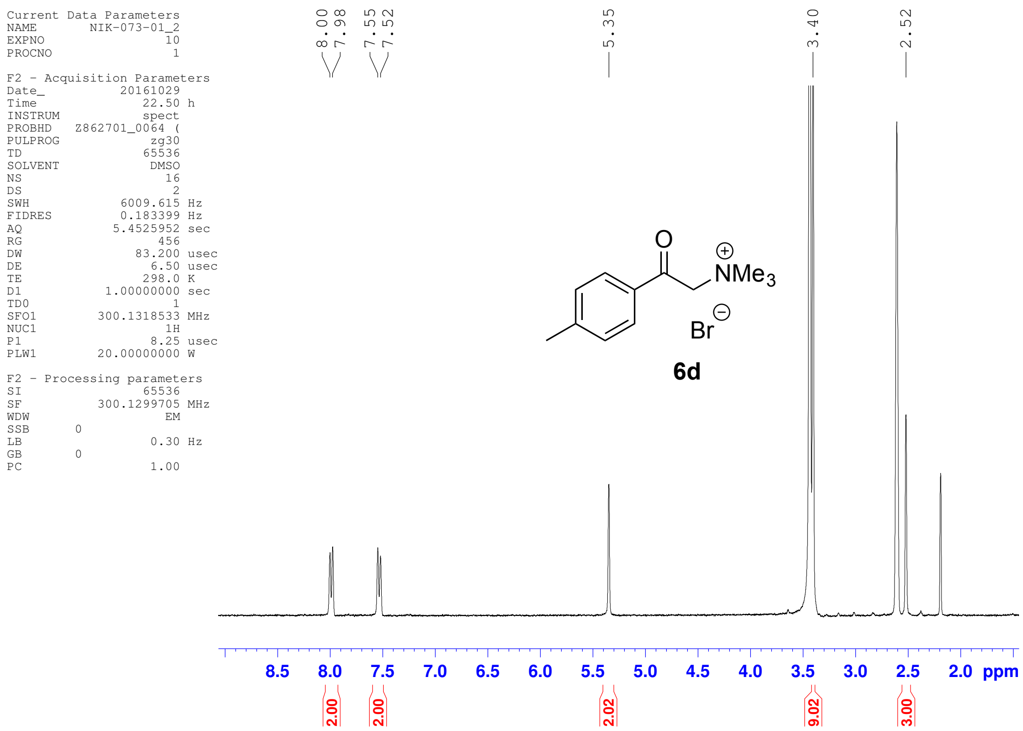
Current Data Parameters
NAME NIK-073-01_2
EXPNO 10
PROCNO 1
F2 - Acquisition Parameters
Date_ 20161029
Time 22.50 h
INSTRUM spect
PROBHD Z862701_0064 (
PULPROG zg30
TD 65536
SOLVENT DMSO
NS 16
DS 2
SWH 6009.615 Hz
FIDRES 0.183399 Hz
AQ 5.4525952 sec
RG 456
DW 83.200 usec
DE 6.50 usec
TE 298.0 K
D1 1.00000000 sec
TD0 1
SFO1 300.1318533 MHz
NUC1 1H
P1 8.25 usec
PLW1 20.00000000 W
F2 - Processing parameters
SI 65536
SF 300.1299705 MHz
WDW EM
SSB 0
LB 0.30 Hz
GB 0
PC 1.00
8.00
7.98
7.55
7.52
5.35
3.40
2.52
O
NMe3
Br
6d
8.5
8.0
7.5
7.0
6.5
6.0
5.5
5.0
4.5
4.0
3.5
3.0
2.5
2.0 ppm
2.00
2.00
2.02
9.02
3.00

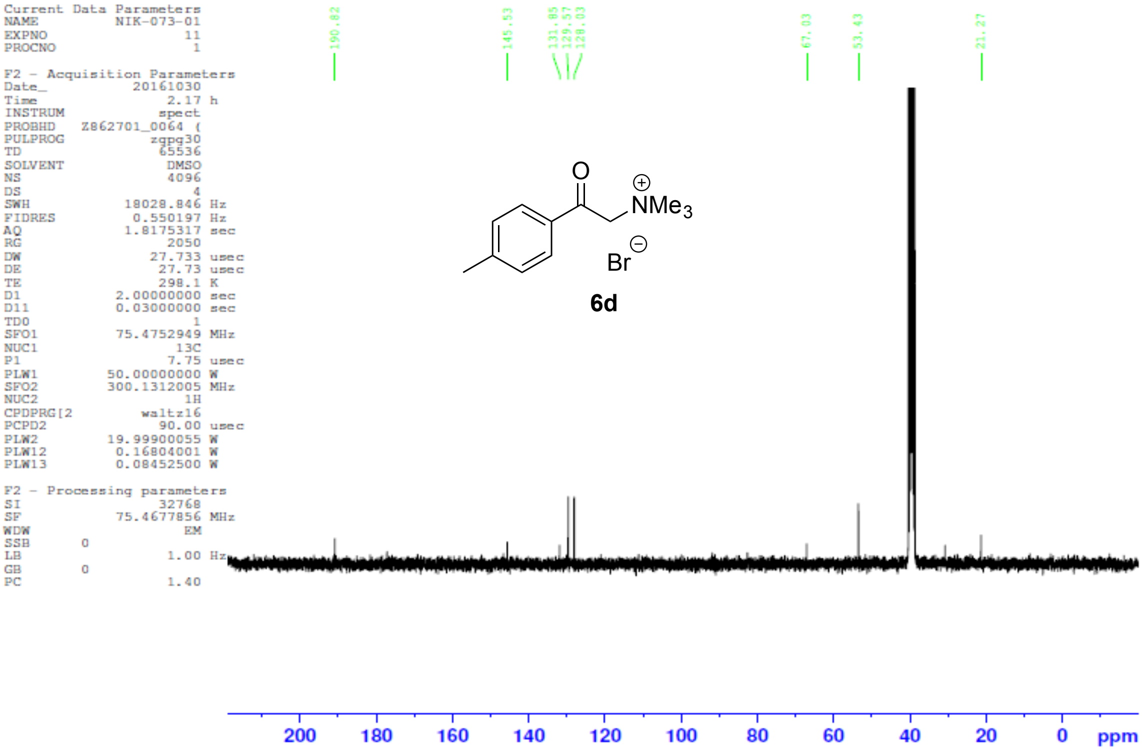
Current Data Parameters
NAME NIK-073-01
EXPNO 11
PROCNO 1
F2 - Acquisition Parameters
Date_ 20161030
Time 2.17 h
INSTRUM spect
PROBHD Z862701_0064 (
PULPROG zgpg30
TD 65536
SOLVENT DMSO
NS 4096
DS 4
SWH 18028.846 Hz
FIDRES 0.550197 Hz
AQ 1.8175317 sec
RG 2050
DW 27.733 usec
DE 27.73 usec
TE 298.1 K
D1 2.00000000 sec
D11 0.03000000 sec
TD0 1
SFO1 75.4752949 MHz
NUC1 13C
P1 7.75 usec
PLW1 50.00000000 W
SFO2 300.1312005 MHz
NUC2 1H
CPDPRG[2 waltz16
PCPD2 90.00 usec
PLW2 19.99900055 W
PLW12 0.16804001 W
PLW13 0.08452500 W
F2 - Processing parameters
SI 32768
SF 75.4677856 MHz
WDW EM
SSB 0
LB 1.00 Hz
GB 0
PC 1.40
190.82
145.53
131.85
129.57
128.03
67.03
53.43
21.27
O
NMe3
Br
6d
200
180
160
140
120
100
80
60
40
20
0
ppm

```
Current Data Parameters
NAME         NIK-091-01_2
EXPNO                  10
PROCNO                  1

F2 - Acquisition Parameters
Date_            20161029
Time                18.20 h
INSTRUM             spect
PROBHD   Z862701_0064 (
PULPROG              zg30
TD                  65536
SOLVENT              DMSO
NS                     16
DS                      2
SWH              6009.615 Hz
FIDRES           0.183399 Hz
AQ              5.4525952 sec
RG                    406
DW                 83.200 usec
DE                   6.50 usec
TE                  297.9 K
D1             1.00000000 sec
TD0                     1
SFO1          300.1318533 MHz
NUC1                   1H
P1                   8.25 usec
PLW1          20.00000000 W

F2 - Processing parameters
SI                  65536
SF            300.1299638 MHz
WDW                    EM
SSB      0
LB                   0.30 Hz
GB       0
PC                   1.00
```

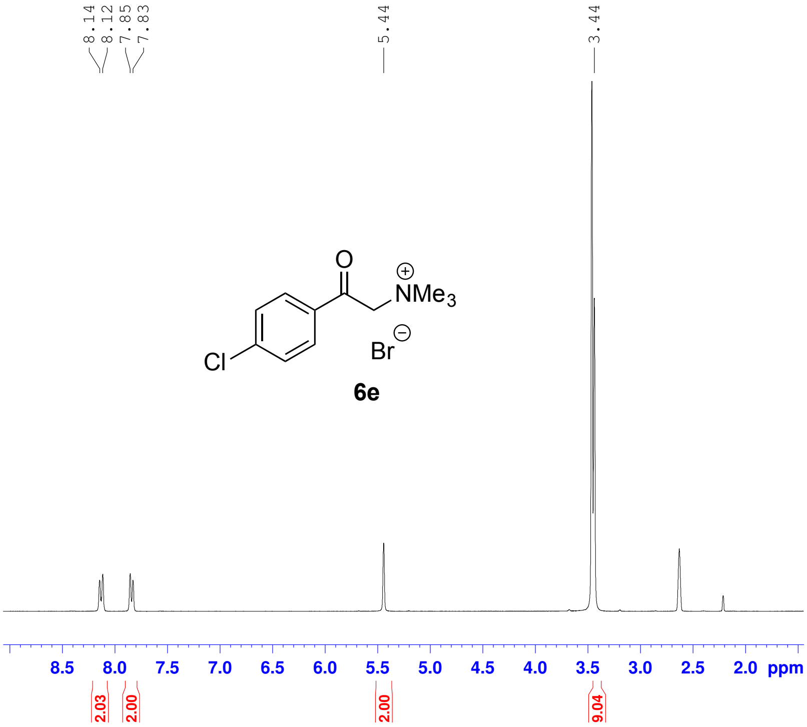


```
Current Data Parameters
NAME           NIK-091-01
EXPNO                  11
PROCNO                  1

F2 - Acquisition Parameters
Date_            20161029
Time                22.47 h
INSTRUM             spect
PROBHD   Z862701_0064 (
PULPROG            zgpg30
TD                  65536
SOLVENT              DMSO
NS                   4096
DS                      4
SWH             18028.846 Hz
FIDRES           0.550197 Hz
AQ              1.8175317 sec
RG                   2050
DW                 27.733 usec
DE                  27.73 usec
TE                  298.0 K
D1             2.00000000 sec
D11            0.03000000 sec
TD0                     1
SFO1           75.4752949 MHz
NUC1                  13C
P1                   7.75 usec
PLW1          50.00000000 W
SFO2          300.1312005 MHz
NUC2                   1H
CPDPRG[2          waltz16
PCPD2               90.00 usec
PLW2          19.99900055 W
PLW12          0.16804001 W
PLW13          0.08452500 W

F2 - Processing parameters
SI                  32768
SF             75.4677853 MHz
WDW                    EM
SSB      0
LB                   1.00 Hz
GB       0
PC                   1.40
```

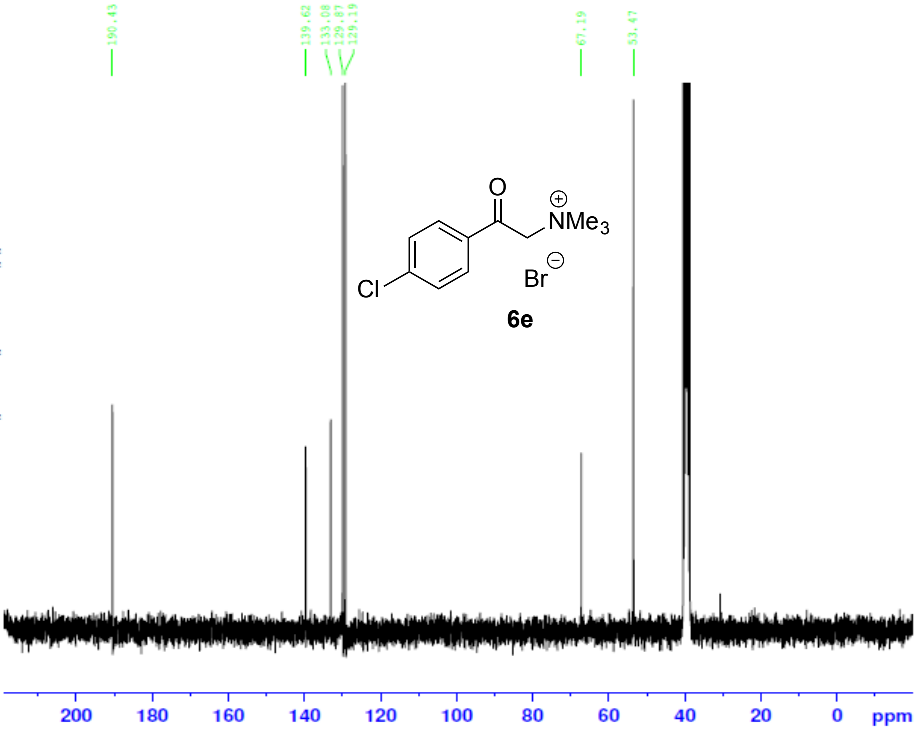

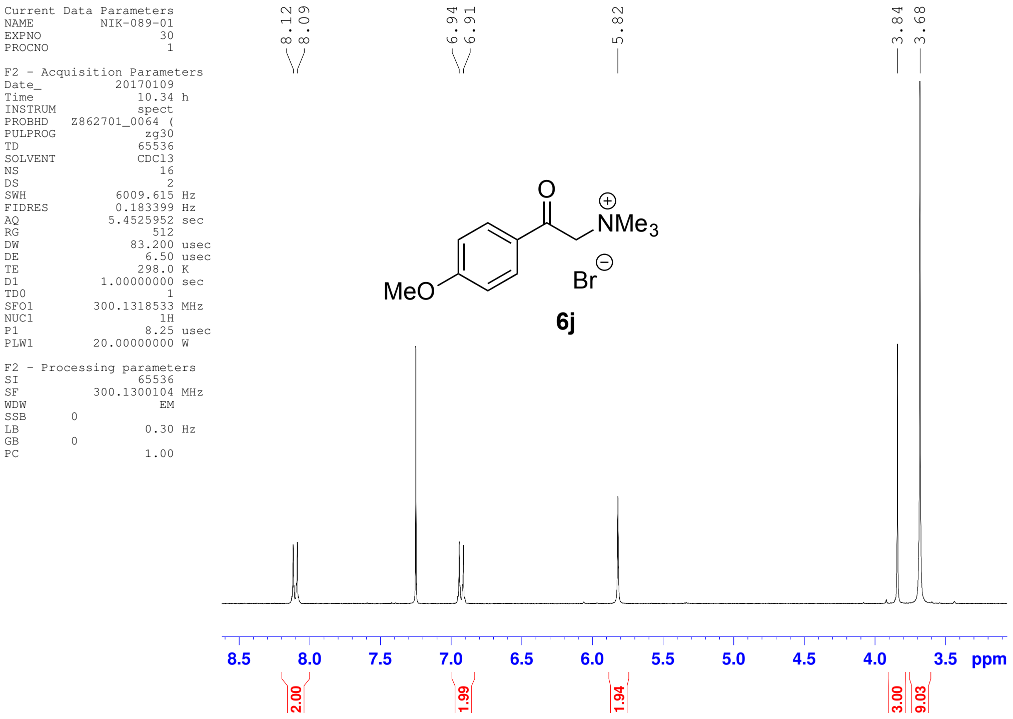
Current Data Parameters
NAME NIK-089-01
EXPNO 30
PROCNO 1
F2 - Acquisition Parameters
Date_ 20170109
Time 10.34 h
INSTRUM spect
PROBHD Z862701_0064 (
PULPROG zg30
TD 65536
SOLVENT CDCl3
NS 16
DS 2
SWH 6009.615 Hz
FIDRES 0.183399 Hz
AQ 5.4525952 sec
RG 512
DW 83.200 usec
DE 6.50 usec
TE 298.0 K
D1 1.00000000 sec
TD0 1
SFO1 300.1318533 MHz
NUC1 1H
P1 8.25 usec
PLW1 20.00000000 W
F2 - Processing parameters
SI 65536
SF 300.1300104 MHz
WDW EM
SSB 0
LB 0.30 Hz
GB 0
PC 1.00
8.12
8.09
6.94
6.91
5.82
3.84
3.68
O
NMe3
MeO
Br
6j
8.5
8.0
7.5
7.0
6.5
6.0
5.5
5.0
4.5
4.0
3.5
ppm
2.00
1.99
1.94
3.00
9.03

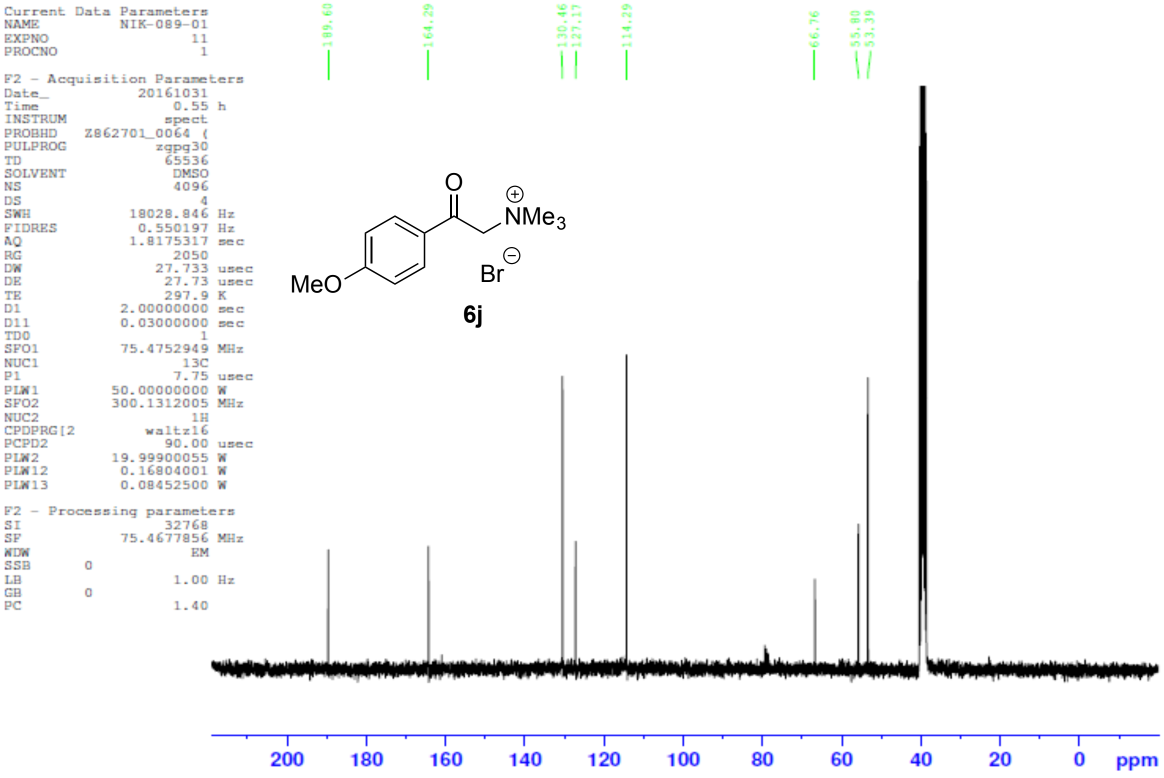
Current Data Parameters
NAME NIK-089-01
EXPNO 11
PROCNO 1
F2 - Acquisition Parameters
Date_ 20161031
Time 0.55 h
INSTRUM spect
PROBHD Z862701_0064 (
PULPROG zgpg30
TD 65536
SOLVENT DMSO
NS 4096
DS 4
SWH 18028.846 Hz
FIDRES 0.550197 Hz
AQ 1.8175317 sec
RG 2050
DW 27.733 usec
DE 27.73 usec
TE 297.9 K
D1 2.00000000 sec
D11 0.03000000 sec
TD0 1
SFO1 75.4752949 MHz
NUC1 13C
P1 7.75 usec
PLW1 50.00000000 W
SFO2 300.1312005 MHz
NUC2 1H
CPDPRG[2 waltz16
PCPD2 90.00 usec
PLW2 19.99900055 W
PLW12 0.16804001 W
PLW13 0.08452500 W
F2 - Processing parameters
SI 32768
SF 75.4677856 MHz
WDW EM
SSB 0
LB 1.00 Hz
GB 0
PC 1.40
189.60
164.29
130.46
127.17
114.29
66.76
55.80
53.39
O
NMe3
MeO
Br
6j
200
180
160
140
120
100
80
60
40
20
0
ppm

Current Data Parameters
NAME NIK-128-05
EXPNO 10
PROCNO 1

F2 - Acquisition Parameters
Date_ 20160726
Time 14.36
INSTRUM spect
PROBHD 5 mm PABBO BB-
PULPROG zg30
TD 65536
SOLVENT CDCl3
NS 16
DS 2
SWH 6188.119 Hz
FIDRES 0.094423 Hz
AQ 5.2953086 sec
RG 645
DW 80.800 usec
DE 6.50 usec
TE 298.0 K
D1 1.00000000 sec

======== CHANNEL f1 ========
NUC1 1H
P1 8.25 usec
PLW1 20.00000000 W
SFO1 300.1318534 MHz

F2 - Processing parameters
SI 65536
SF 300.1300083 MHz
WDW EM
SSB 0
LB 0.30 Hz
GB 0
PC 1.00

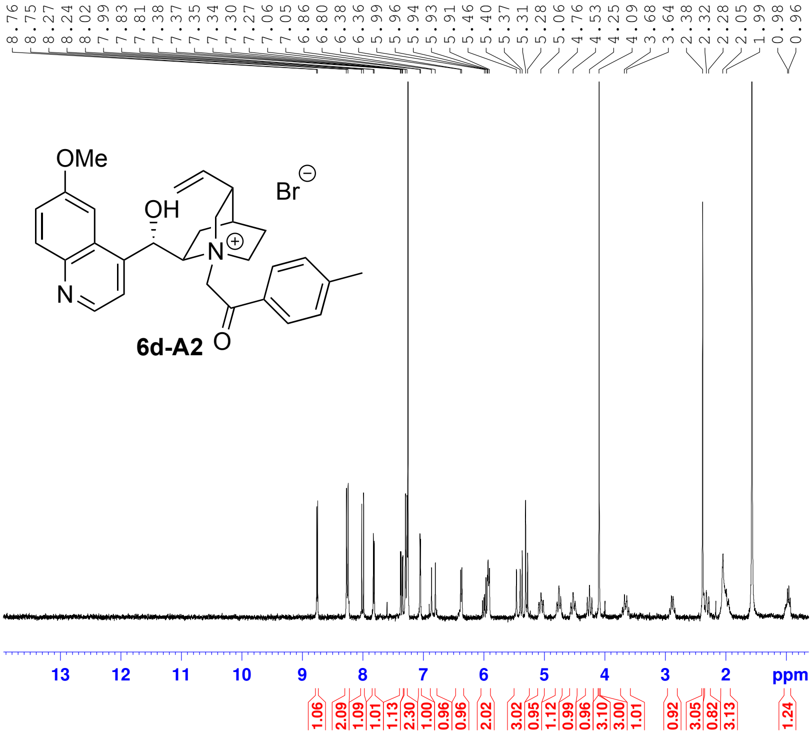

Current Data Parameters
NAME NIK-128-05
EXPNO 30
PROCNO 1

F2 - Acquisition Parameters
Date_ 20161110
Time 1.51 h
INSTRUM spect
PROBHD Z862701_0064 (
PULPROG zgpg30
TD 65536
SOLVENT MeOD
NS 2048
DS 4
SWH 18028.846 Hz
FIDRES 0.550197 Hz
AQ 1.8175317 sec
RG 2050
DW 27.733 usec
DE 27.73 usec
TE 298.0 K
D1 2.00000000 sec
D11 0.03000000 sec
TD0 1
SFO1 75.4752949 MHz
NUC1 13C
P1 7.75 usec
PLW1 50.00000000 W
SFO2 300.1312005 MHz
NUC2 1H
CPDPRG[2 waltz16
PCPD2 90.00 usec
PLW2 19.99900055 W
PLW12 0.16804001 W
PLW13 0.08452500 W

F2 - Processing parameters
SI 32768
SF 75.4676438 MHz
WDW EM
SSB 0
LB 1.00 Hz
GB 0
PC 1.40

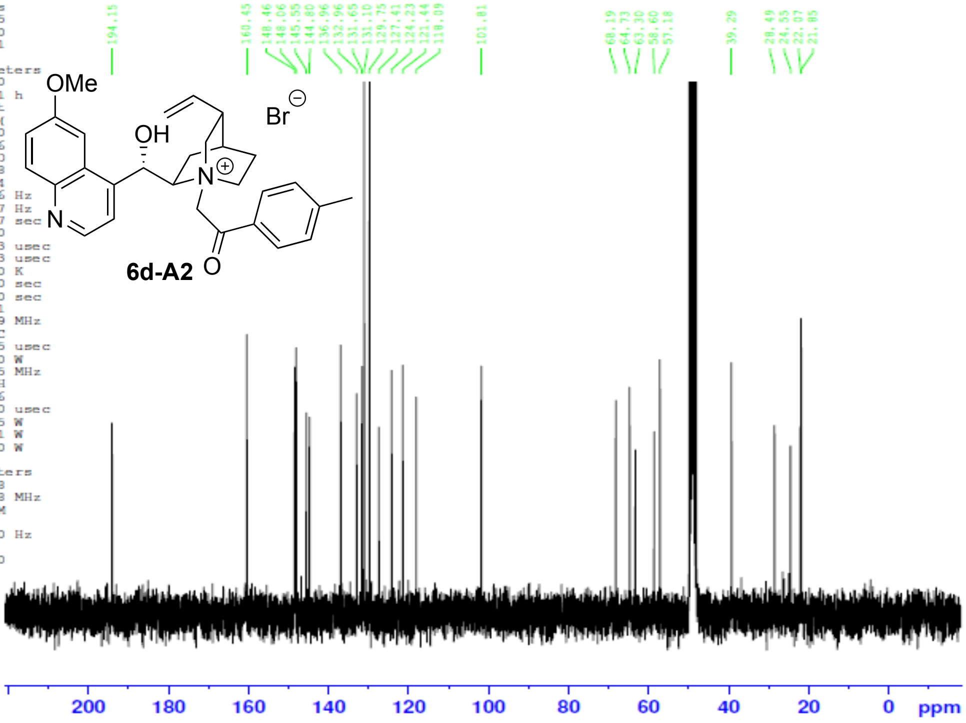

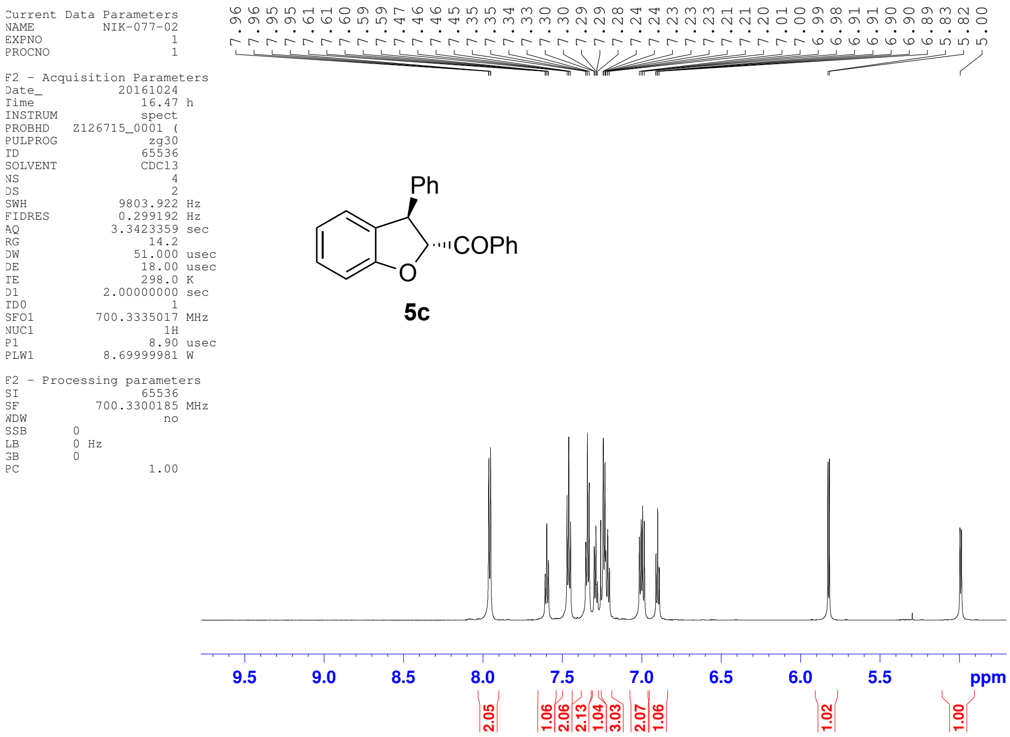
Current Data Parameters
NAME NIK-077-02
EXPNO 1
PROCNO 1
F2 - Acquisition Parameters
Date_ 20161024
Time 16.47 h
INSTRUM spect
PROBHD Z126715_0001 (
PULPROG zg30
TD 65536
SOLVENT CDCl3
NS 4
DS 2
SWH 9803.922 Hz
FIDRES 0.299192 Hz
AQ 3.3423359 sec
RG 14.2
DW 51.000 usec
DE 18.00 usec
TE 298.0 K
D1 2.00000000 sec
TD0 1
SFO1 700.3335017 MHz
NUC1 1H
P1 8.90 usec
PLW1 8.69999981 W
F2 - Processing parameters
SI 65536
SF 700.3300185 MHz
WDW no
SSB 0
LB 0 Hz
GB 0
PC 1.00
7.96 7.96 7.95 7.61 7.61 7.60 7.59 7.59 7.47 7.46 7.46 7.45 7.35 7.35 7.34 7.33 7.30 7.30 7.29 7.29 7.28 7.24 7.24 7.23 7.23 7.23 7.21 7.21 7.20 7.01 7.00 6.99 6.98 6.91 6.91 6.90 6.90 6.89 5.83 5.82 5.00
Ph
COPh
O
5c
9.5 9.0 8.5 8.0 7.5 7.0 6.5 6.0 5.5 ppm
2.05 1.06 2.06 2.13 1.04 3.03 2.07 1.06 1.02 1.00

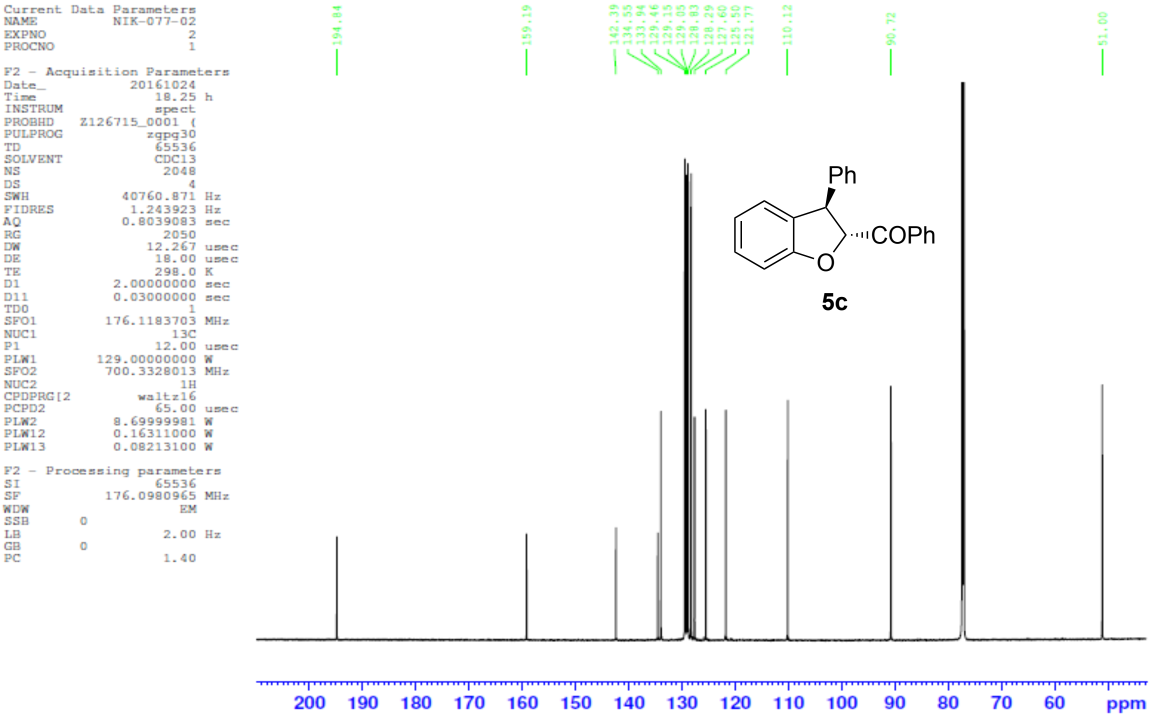
Current Data Parameters
NAME NIK-077-02
EXPNO 2
PROCNO 1
F2 - Acquisition Parameters
Date_ 20161024
Time 18.25 h
INSTRUM spect
PROBHD Z126715_0001 (
PULPROG zgpg30
TD 65536
SOLVENT CDCl3
NS 2048
DS 4
SWH 40760.871 Hz
FIDRES 1.243923 Hz
AQ 0.8039083 sec
RG 2050
DW 12.267 usec
DE 18.00 usec
TE 298.0 K
D1 2.00000000 sec
D11 0.03000000 sec
TD0 1
SFO1 176.1183703 MHz
NUC1 13C
P1 12.00 usec
PLW1 129.00000000 W
SFO2 700.3328013 MHz
NUC2 1H
CPDPRG[2 waltz16
PCPD2 65.00 usec
PLW2 8.69999981 W
PLW12 0.16311000 W
PLW13 0.08213100 W
F2 - Processing parameters
SI 65536
SF 176.0980965 MHz
WDW EM
SSB 0
LB 2.00 Hz
GB 0
PC 1.40
194.84 159.19 142.39 134.55 133.94 129.46 129.15 129.05 128.93 128.29 127.60 125.50 121.77 110.12 90.72 51.00
Ph
COPh
O
5c
200 190 180 170 160 150 140 130 120 110 100 90 80 70 60 ppm

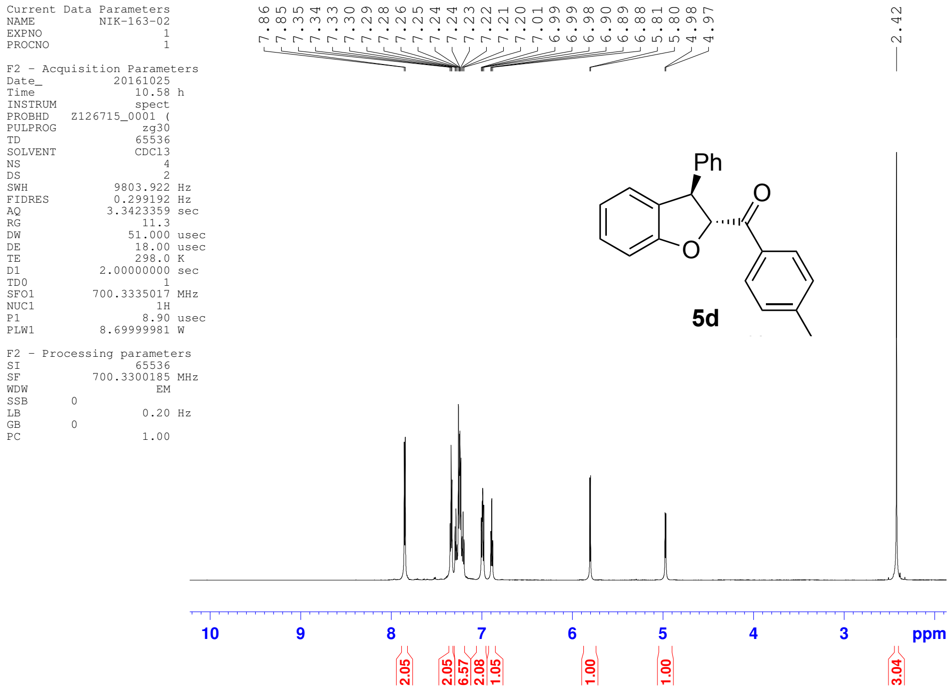
Current Data Parameters
NAME NIK-163-02
EXPNO 1
PROCNO 1
F2 - Acquisition Parameters
Date_ 20161025
Time 10.58 h
INSTRUM spect
PROBHD Z126715_0001 (
PULPROG zg30
TD 65536
SOLVENT CDCl3
NS 4
DS 2
SWH 9803.922 Hz
FIDRES 0.299192 Hz
AQ 3.3423359 sec
RG 11.3
DW 51.000 usec
DE 18.00 usec
TE 298.0 K
D1 2.00000000 sec
TD0 1
SFO1 700.3335017 MHz
NUC1 1H
P1 8.90 usec
PLW1 8.69999981 W
F2 - Processing parameters
SI 65536
SF 700.3300185 MHz
WDW EM
SSB 0
LB 0.20 Hz
GB 0
PC 1.00
7.86 7.85 7.35 7.34 7.33 7.30 7.29 7.28 7.26 7.25 7.24 7.24 7.23 7.22 7.21 7.20 7.01 6.99 6.99 6.98 6.90 6.89 6.88 5.81 5.80 4.98 4.97
2.42
Ph
O
O
5d
10
9
8
7
6
5
4
3
ppm
2.05
2.05
6.57
2.08
1.05
1.00
1.00
3.04

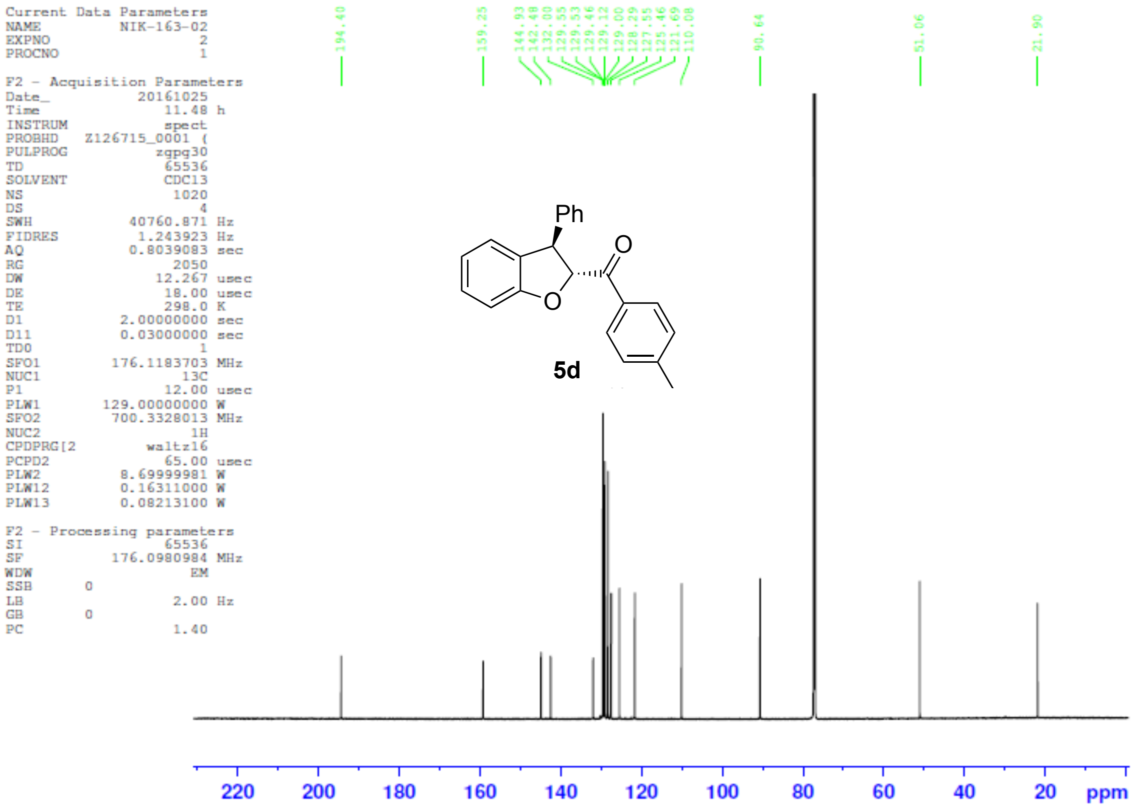
Current Data Parameters
NAME NIK-163-02
EXPNO 2
PROCNO 1
F2 - Acquisition Parameters
Date_ 20161025
Time 11.48 h
INSTRUM spect
PROBHD Z126715_0001 (
PULPROG zgpg30
TD 65536
SOLVENT CDCl3
NS 1020
DS 4
SWH 40760.871 Hz
FIDRES 1.243923 Hz
AQ 0.8039083 sec
RG 2050
DW 12.267 usec
DE 18.00 usec
TE 298.0 K
D1 2.00000000 sec
D11 0.03000000 sec
TD0 1
SFO1 176.1183703 MHz
NUC1 13C
P1 12.00 usec
PLW1 129.00000000 W
SFO2 700.3328013 MHz
NUC2 1H
CPDPRG[2 waltz16
PCPD2 65.00 usec
PLW2 8.69999981 W
PLW12 0.16311000 W
PLW13 0.08213100 W
F2 - Processing parameters
SI 65536
SF 176.0980984 MHz
WDW EM
SSB 0
LB 2.00 Hz
GB 0
PC 1.40
194.40
159.25
144.93 142.48 132.00 129.55 129.53 129.46 129.12 129.00 128.29 127.55 125.46 121.69 110.08
90.64
51.06
21.90
Ph
O
O
5d
220
200
180
160
140
120
100
80
60
40
20
ppm

```
Current Data Parameters
NAME          NIK-147-02
EXPNO                  1
PROCNO                 1

F2 - Acquisition Parameters
Date_           20161025
Time               14.54 h
INSTRUM            spect
PROBHD   Z126715_0001 (
PULPROG             zg30
TD                 65536
SOLVENT            CDCl3
NS                     4
DS                     2
SWH             9803.922 Hz
FIDRES          0.299192 Hz
AQ             3.3423359 sec
RG                    16
DW                51.000 usec
DE                 18.00 usec
TE                 298.0 K
D1            2.00000000 sec
TD0                    1
SFO1         700.3335017 MHz
NUC1                  1H
P1                  8.90 usec
PLW1          8.69999981 W

F2 - Processing parameters
SI                 65536
SF           700.3300185 MHz
WDW                   EM
SSB      0
LB                  0.20 Hz
GB       0
PC                  1.00
```

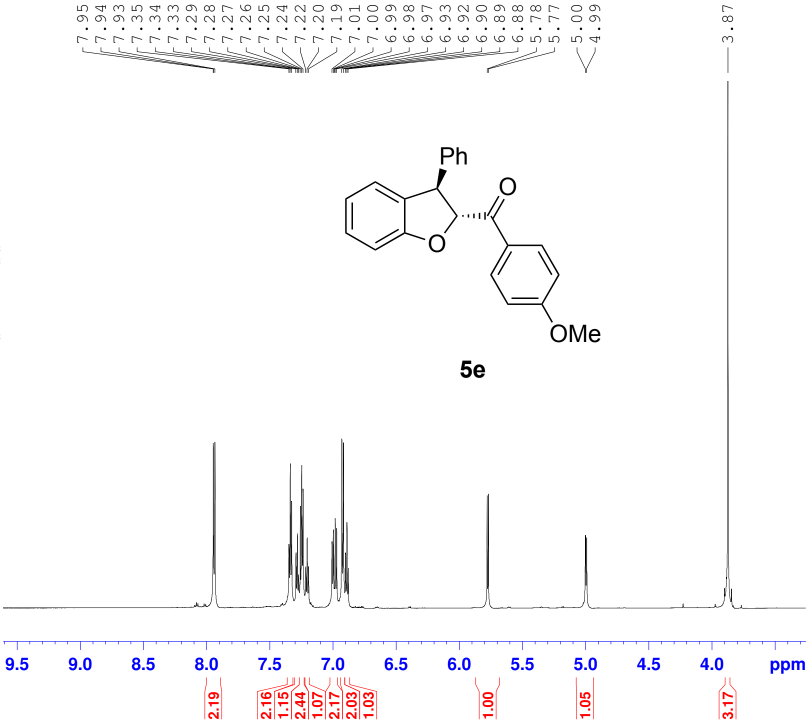


```
Current Data Parameters
NAME          NIK-147-02
EXPNO                  2
PROCNO                 1

F2 - Acquisition Parameters
Date_           20161025
Time               16.33 h
INSTRUM            spect
PROBHD   Z126715_0001 (
PULPROG           zgpg30
TD                 65536
SOLVENT            CDCl3
NS                  2048
DS                     4
SWH            40760.871 Hz
FIDRES          1.243923 Hz
AQ             0.8039083 sec
RG                  2050
DW                12.267 usec
DE                 18.00 usec
TE                 298.0 K
D1            2.00000000 sec
D11           0.03000000 sec
TD0                    1
SFO1         176.1183703 MHz
NUC1                 13C
P1                 12.00 usec
PLW1        129.00000000 W
SFO2         700.3328013 MHz
NUC2                  1H
CPDPRG[2         waltz16
PCPD2              65.00 usec
PLW2          8.69999981 W
PLW12         0.16311000 W
PLW13         0.08213100 W

F2 - Processing parameters
SI                 65536
SF           176.0980893 MHz
WDW                   EM
SSB      0
LB                  2.00 Hz
GB       0
PC                  1.40
```

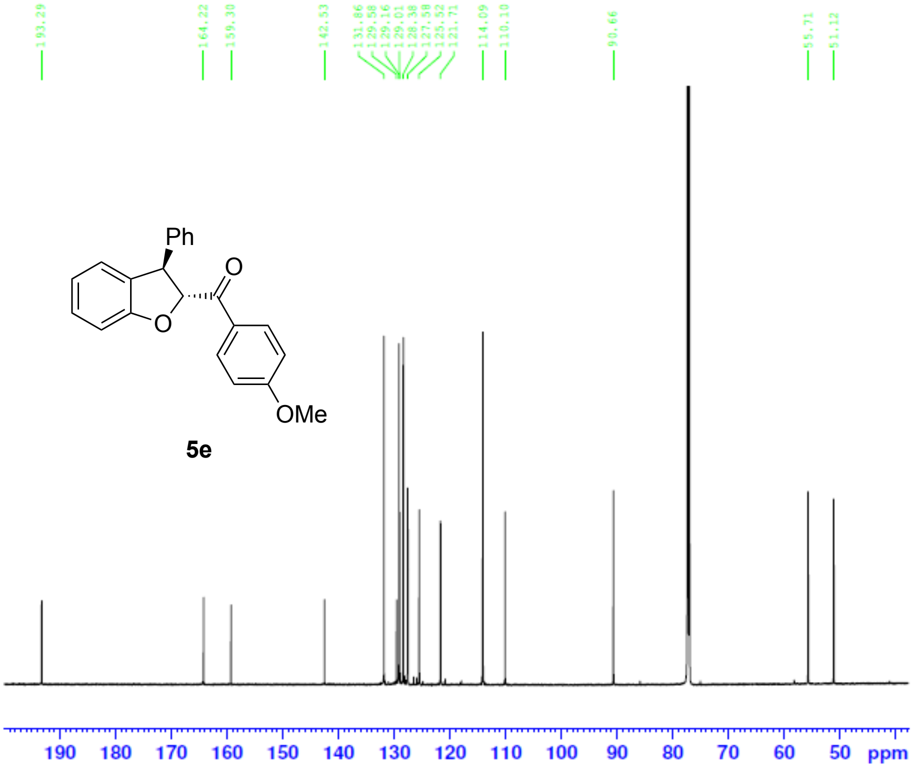

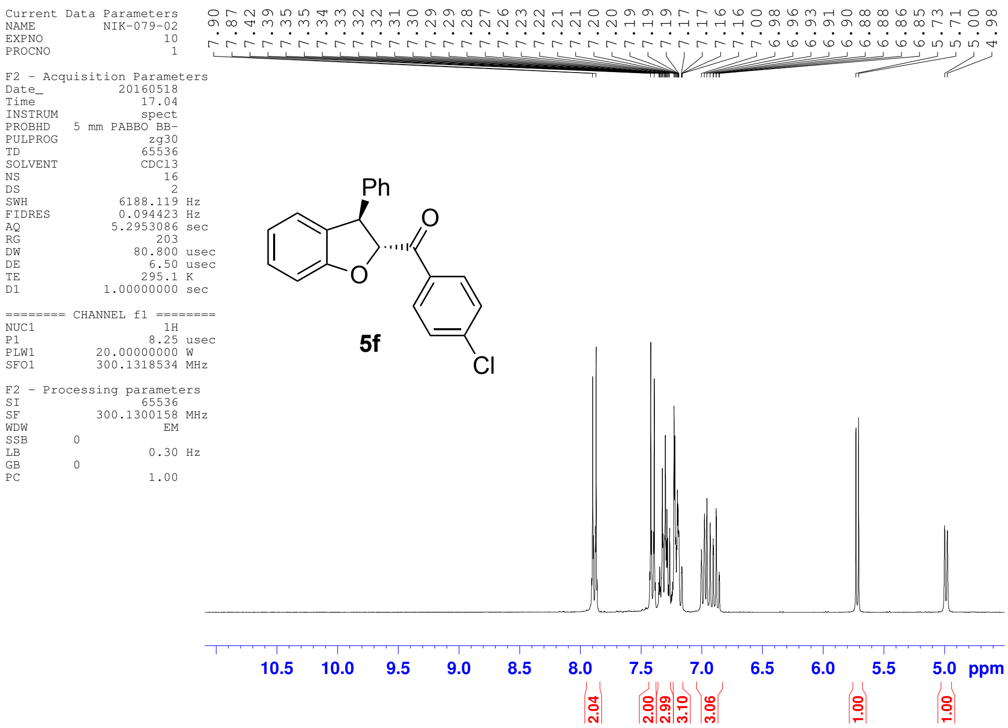
Current Data Parameters
NAME NIK-079-02
EXPNO 10
PROCNO 1
F2 - Acquisition Parameters
Date_ 20160518
Time 17.04
INSTRUM spect
PROBHD 5 mm PABBO BB-
PULPROG zg30
TD 65536
SOLVENT CDCl3
NS 16
DS 2
SWH 6188.119 Hz
FIDRES 0.094423 Hz
AQ 5.2953086 sec
RG 203
DW 80.800 usec
DE 6.50 usec
TE 295.1 K
D1 1.00000000 sec
======== CHANNEL f1 ========
NUC1 1H
P1 8.25 usec
PLW1 20.00000000 W
SFO1 300.1318534 MHz
F2 - Processing parameters
SI 65536
SF 300.1300158 MHz
WDW EM
SSB 0
LB 0.30 Hz
GB 0
PC 1.00
7.90 7.87 7.42 7.39 7.35 7.35 7.34 7.33 7.32 7.32 7.31 7.30 7.29 7.29 7.28 7.27 7.26 7.23 7.22 7.21 7.21 7.20 7.20 7.19 7.19 7.19 7.17 7.17 7.16 7.16 7.00 6.98 6.96 6.93 6.91 6.90 6.88 6.88 6.86 6.85 5.73 5.71 5.00 4.98
Ph
O
O
Cl
5f
10.5 10.0 9.5 9.0 8.5 8.0 7.5 7.0 6.5 6.0 5.5 5.0 ppm
2.04 2.00 2.99 3.10 3.06 1.00 1.00

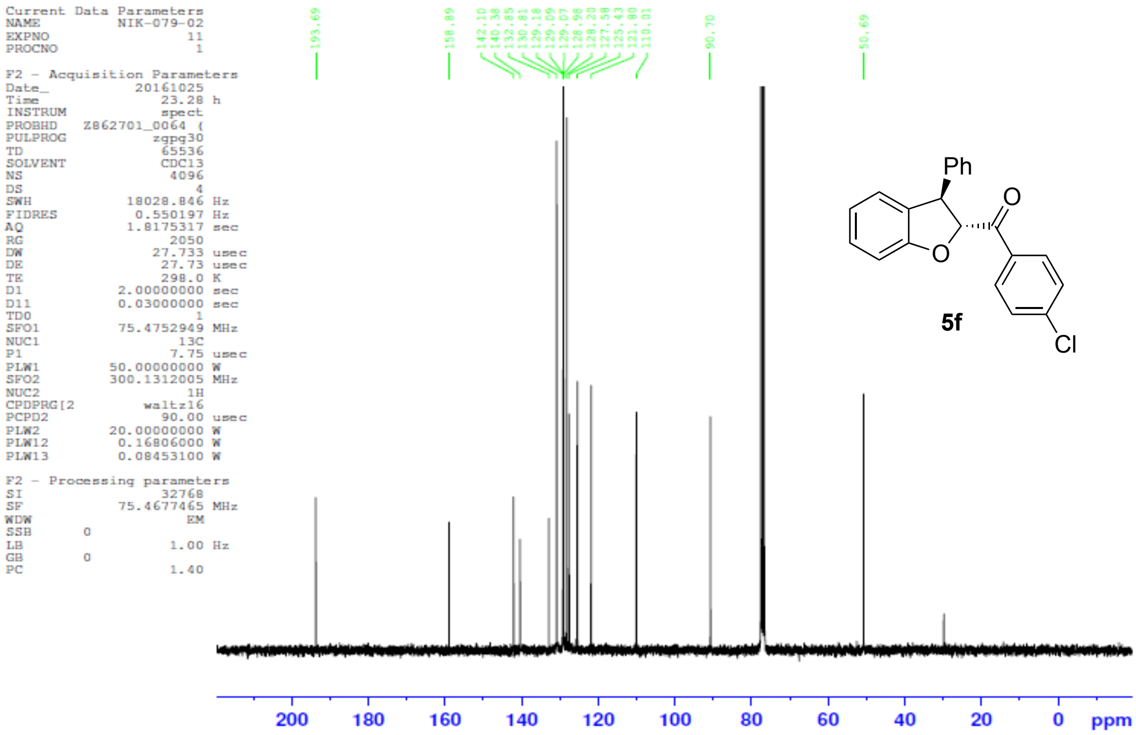
Current Data Parameters
NAME NIK-079-02
EXPNO 11
PROCNO 1
F2 - Acquisition Parameters
Date_ 20161025
Time 23.28 h
INSTRUM spect
PROBHD Z862701_0064 (
PULPROG zgpg30
TD 65536
SOLVENT CDCl3
NS 4096
DS 4
SWH 18028.846 Hz
FIDRES 0.550197 Hz
AQ 1.8175317 sec
RG 2050
DW 27.733 usec
DE 27.73 usec
TE 298.0 K
D1 2.00000000 sec
D11 0.03000000 sec
TD0 1
SFO1 75.4752949 MHz
NUC1 13C
P1 7.75 usec
PLW1 50.00000000 W
SFO2 300.1312005 MHz
NUC2 1H
CPDPRG[2 waltz16
PCPD2 90.00 usec
PLW2 20.00000000 W
PLW12 0.16806000 W
PLW13 0.08453100 W
F2 - Processing parameters
SI 32768
SF 75.4677465 MHz
WDW EM
SSB 0
LB 1.00 Hz
GB 0
PC 1.40
193.69 158.89 142.10 140.38 132.85 130.81 129.18 129.09 129.07 128.98 128.20 127.58 125.43 121.80 110.01 90.70 50.69
Ph
O
O
Cl
5f
200 180 160 140 120 100 80 60 40 20 0 ppm

Current Data Parameters
NAME NIK-136-02
EXPNO 1
PROCNO 1

F2 - Acquisition Parameters
Date_ 20161024
Time 14.04 h
INSTRUM spect
PROBHD Z126715_0001 (
PULPROG zg30
TD 65536
SOLVENT CDCl3
NS 4
DS 2
SWH 9803.922 Hz
FIDRES 0.299192 Hz
AQ 3.3423359 sec
RG 12.7
DW 51.000 usec
DE 18.00 usec
TE 298.0 K
D1 2.00000000 sec
TD0 1
SFO1 700.3335017 MHz
NUC1 1H
P1 8.90 usec
PLW1 8.69999981 W

F2 - Processing parameters
SI 65536
SF 700.3300310 MHz
WDW EM
SSB 0
LB 0.20 Hz
GB 0
PC 1.00

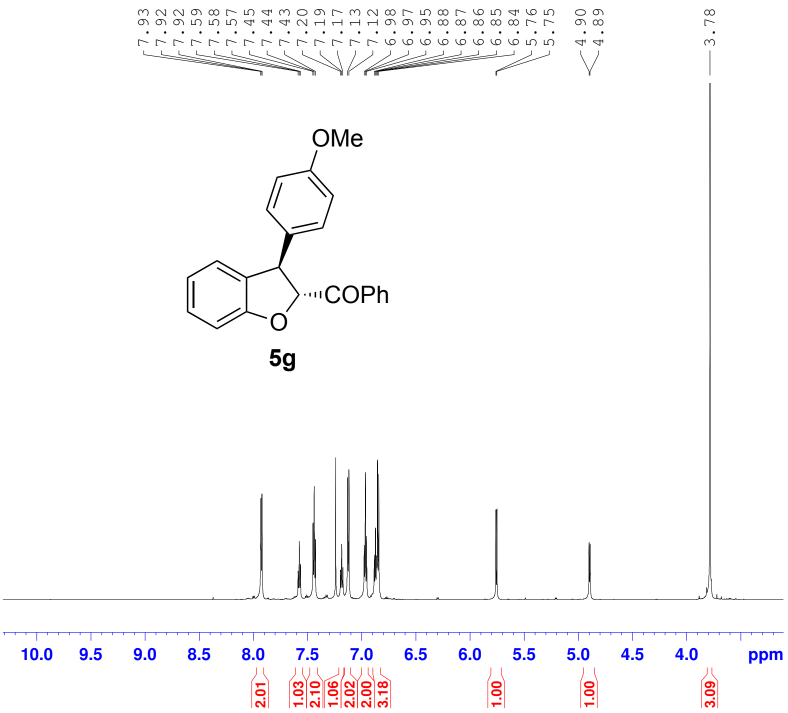

Current Data Parameters
NAME NIK-136-02
EXPNO 2
PROCNO 1

F2 - Acquisition Parameters
Date_ 20161024
Time 14.50 h
INSTRUM spect
PROBHD Z126715_0001 (
PULPROG zgpg30
TD 65536
SOLVENT CDCl3
NS 963
DS 4
SWH 40760.871 Hz
FIDRES 1.243923 Hz
AQ 0.8039083 sec
RG 2050
DW 12.267 usec
DE 18.00 usec
TE 298.0 K
D1 2.00000000 sec
D11 0.03000000 sec
TD0 1
SFO1 176.1183703 MHz
NUC1 13C
P1 12.00 usec
PLW1 129.00000000 W
SFO2 700.3328013 MHz
NUC2 1H
CPDPRG[2 waltz16
PCPD2 65.00 usec
PLW2 8.69999981 W
PLW12 0.16311000 W
PLW13 0.08213100 W

F2 - Processing parameters
SI 65536
SF 176.0980954 MHz
WDW EM
SSB 0
LB 2.00 Hz
GB 0
PC 1.40

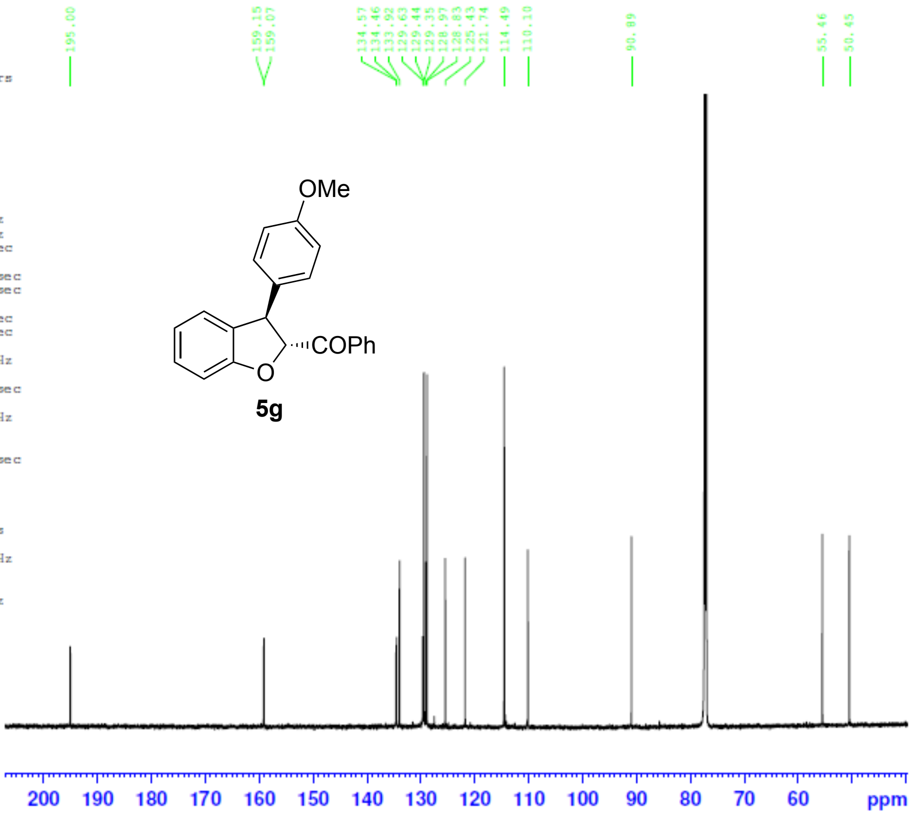

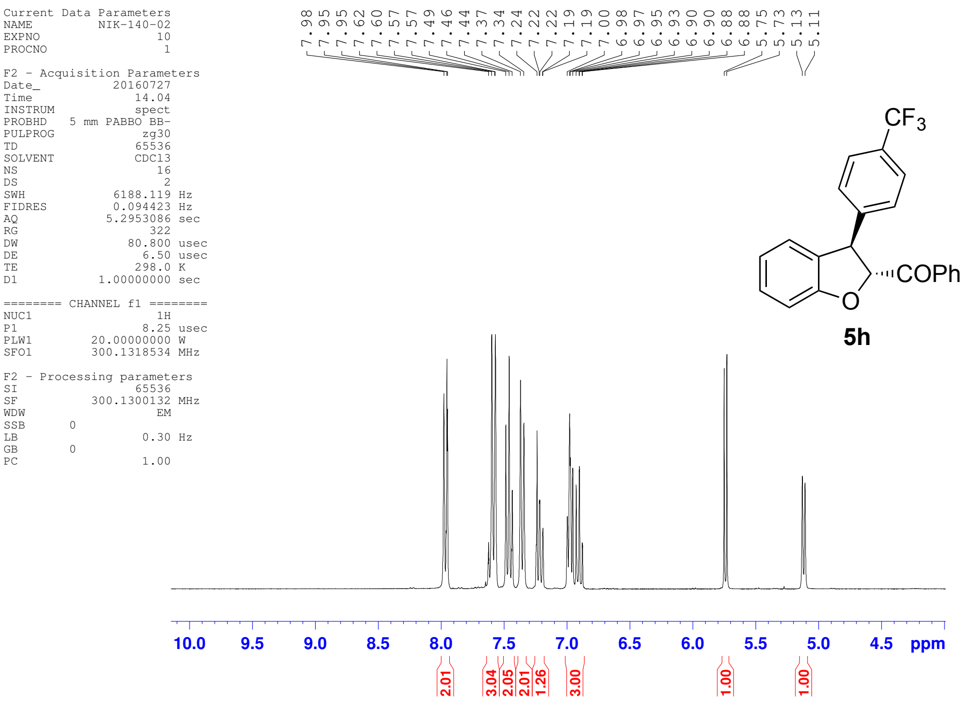
Current Data Parameters
NAME NIK-140-02
EXPNO 10
PROCNO 1
F2 - Acquisition Parameters
Date_ 20160727
Time 14.04
INSTRUM spect
PROBHD 5 mm PABBO BB-
PULPROG zg30
TD 65536
SOLVENT CDCl3
NS 16
DS 2
SWH 6188.119 Hz
FIDRES 0.094423 Hz
AQ 5.2953086 sec
RG 322
DW 80.800 usec
DE 6.50 usec
TE 298.0 K
D1 1.00000000 sec
======== CHANNEL f1 ========
NUC1 1H
P1 8.25 usec
PLW1 20.00000000 W
SFO1 300.1318534 MHz
F2 - Processing parameters
SI 65536
SF 300.1300132 MHz
WDW EM
SSB 0
LB 0.30 Hz
GB 0
PC 1.00
7.98 7.95 7.95 7.62 7.60 7.57 7.57 7.49 7.46 7.44 7.37 7.34 7.24 7.22 7.22 7.19 7.19 7.00 6.98 6.97 6.95 6.93 6.90 6.90 6.88 6.88 5.75 5.73 5.13 5.11
CF3
COPh
O
5h
10.0 9.5 9.0 8.5 8.0 7.5 7.0 6.5 6.0 5.5 5.0 4.5 ppm
2.01 3.04 2.05 2.01 1.26 3.00 1.00 1.00

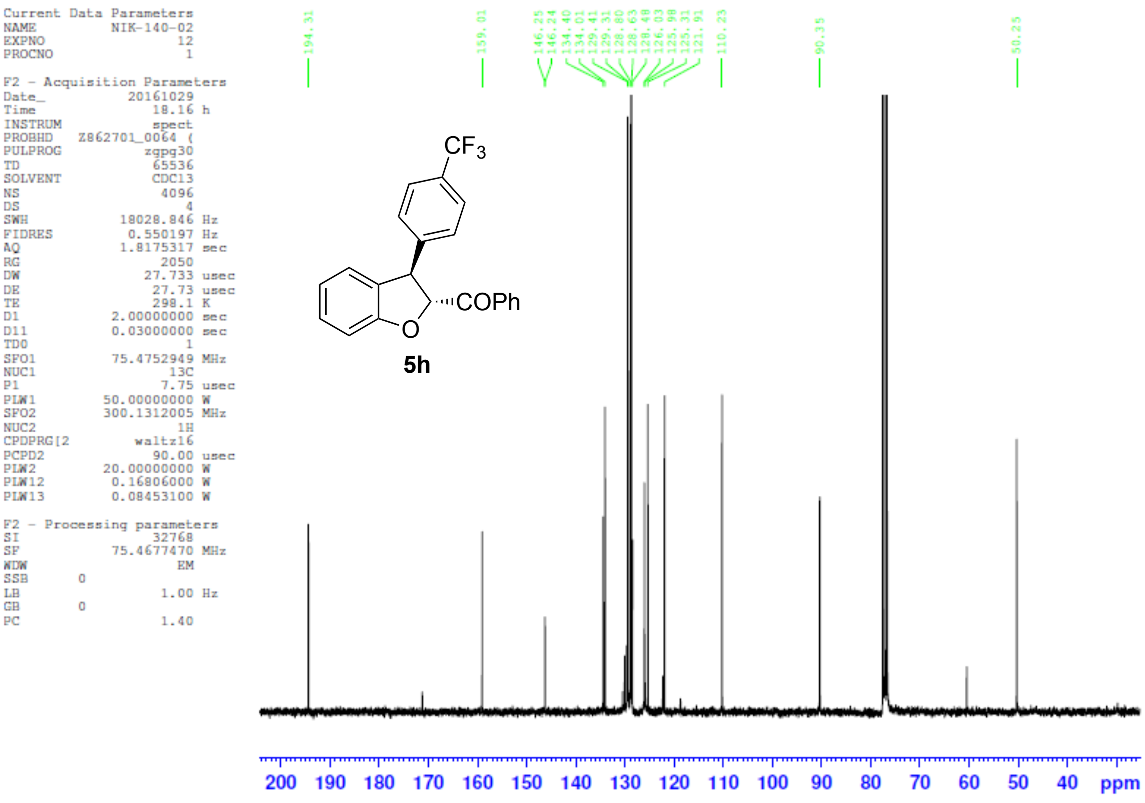
Current Data Parameters
NAME NIK-140-02
EXPNO 12
PROCNO 1
F2 - Acquisition Parameters
Date_ 20161029
Time 18.16 h
INSTRUM spect
PROBHD Z862701_0064 (
PULPROG zgpg30
TD 65536
SOLVENT CDCl3
NS 4096
DS 4
SWH 18028.846 Hz
FIDRES 0.550197 Hz
AQ 1.8175317 sec
RG 2050
DW 27.733 usec
DE 27.73 usec
TE 298.1 K
D1 2.00000000 sec
D11 0.03000000 sec
TD0 1
SFO1 75.4752949 MHz
NUC1 13C
P1 7.75 usec
PLW1 50.00000000 W
SFO2 300.1312005 MHz
NUC2 1H
CPDPRG[2 waltz16
PCPD2 90.00 usec
PLW2 20.00000000 W
PLW12 0.16806000 W
PLW13 0.08453100 W
F2 - Processing parameters
SI 32768
SF 75.4677470 MHz
WDW EM
SSB 0
LB 1.00 Hz
GB 0
PC 1.40
194.31 159.01 146.25 146.24 134.40 134.01 129.41 129.31 128.80 128.63 128.49 126.03 125.98 125.31 121.91 110.23 90.35 50.25
CF3
COPh
O
5h
200 190 180 170 160 150 140 130 120 110 100 90 80 70 60 50 40 ppm

Current Data Parameters
NAME NIK-140-02
EXPNO 20
PROCNO 1

F2 - Acquisition Parameters
Date_ 20161107
Time 13.47 h
INSTRUM spect
PROBHD Z862701_0064 (
PULPROG zgflqn
TD 131072
SOLVENT CDCl3
NS 16
DS 4
SWH 66964.289 Hz
FIDRES 1.021794 Hz
AQ 0.9786710 sec
RG 724
DW 7.467 usec
DE 6.50 usec
TE 298.0 K
D1 1.00000000 sec
TD0 1
SFO1 282.3761148 MHz
NUC1 19F
P1 8.60 usec
PLW1 19.99900055 W

F2 - Processing parameters
SI 65536
SF 282.4043550 MHz
WDW EM
SSB 0
LB 0.30 Hz
GB 0
PC 1.00

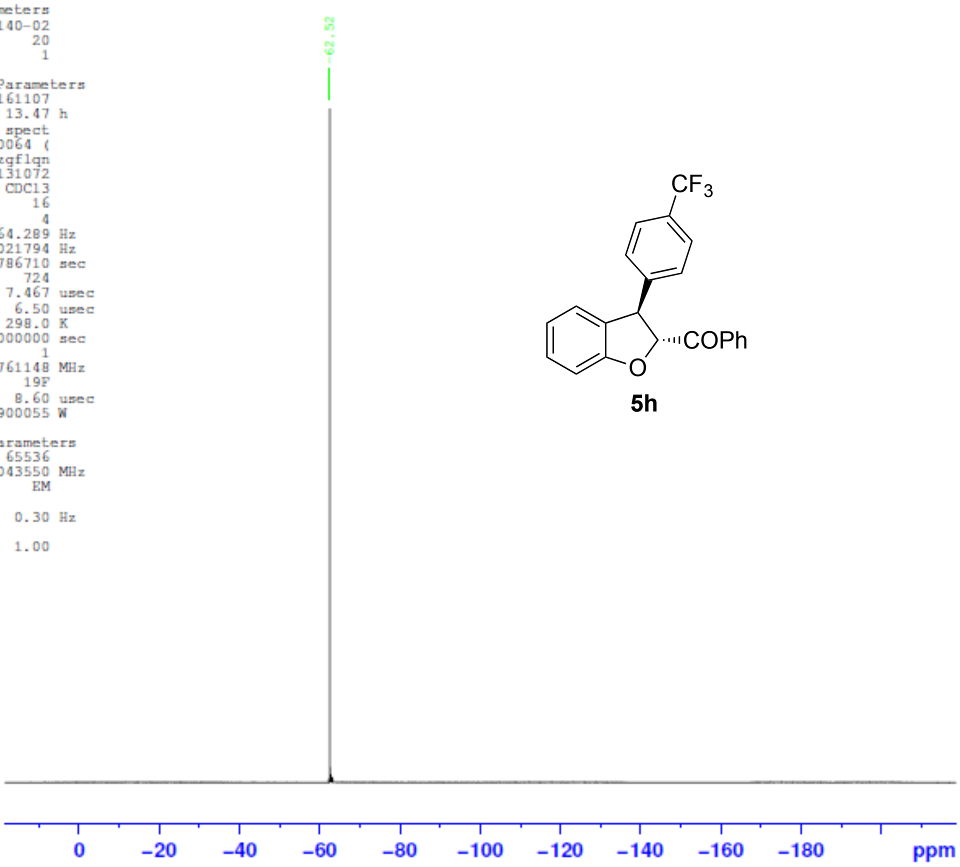

5h

Current Data Parameters
NAME NIK-176-02
EXPNO 10
PROCNO 1

F2 - Acquisition Parameters
Date_ 20161005
Time 15.50 h
INSTRUM spect
PROBHD Z862701_0064 (
PULPROG zg30
TD 65536
SOLVENT CDCl3
NS 16
DS 2
SWH 6009.615 Hz
FIDRES 0.183399 Hz
AQ 5.4525952 sec
RG 406
DW 83.200 usec
DE 6.50 usec
TE 298.0 K
D1 1.00000000 sec
TD0 1
SFO1 300.1318533 MHz
NUC1 1H
P1 8.25 usec
PLW1 20.00000000 W

F2 - Processing parameters
SI 65536
SF 300.1300091 MHz
WDW EM
SSB 0
LB 0.30 Hz
GB 0
PC 1.00

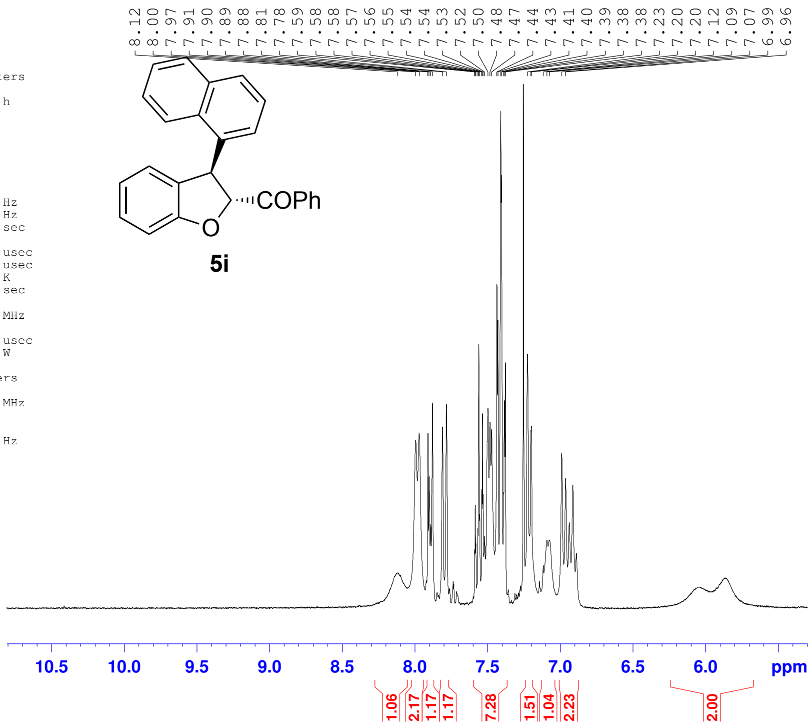

Current Data Parameters
NAME NIK-176-02
EXPNO 21
PROCNO 1

F2 - Acquisition Parameters
Date_ 20161030
Time 20.24 h
INSTRUM spect
PROBHD Z862701_0064 (
PULPROG zgpg30
TD 65536
SOLVENT CDCl3
NS 4096
DS 4
SWH 18028.846 Hz
FIDRES 0.550197 Hz
AQ 1.8175317 sec
RG 2050
DW 27.733 usec
DE 27.73 usec
TE 297.9 K
D1 2.00000000 sec
D11 0.03000000 sec
TD0 1
SFO1 75.4752949 MHz
NUC1 13C
P1 7.75 usec
PLW1 50.00000000 W
SFO2 300.1312005 MHz
NUC2 1H
CPDPRG[2 waltz16
PCPD2 90.00 usec
PLW2 20.00000000 W
PLW12 0.16806000 W
PLW13 0.08453100 W

F2 - Processing parameters
SI 32768
SF 75.4677478 MHz
WDW EM
SSB 0
LB 4.50 Hz
GB 0
PC 1.40

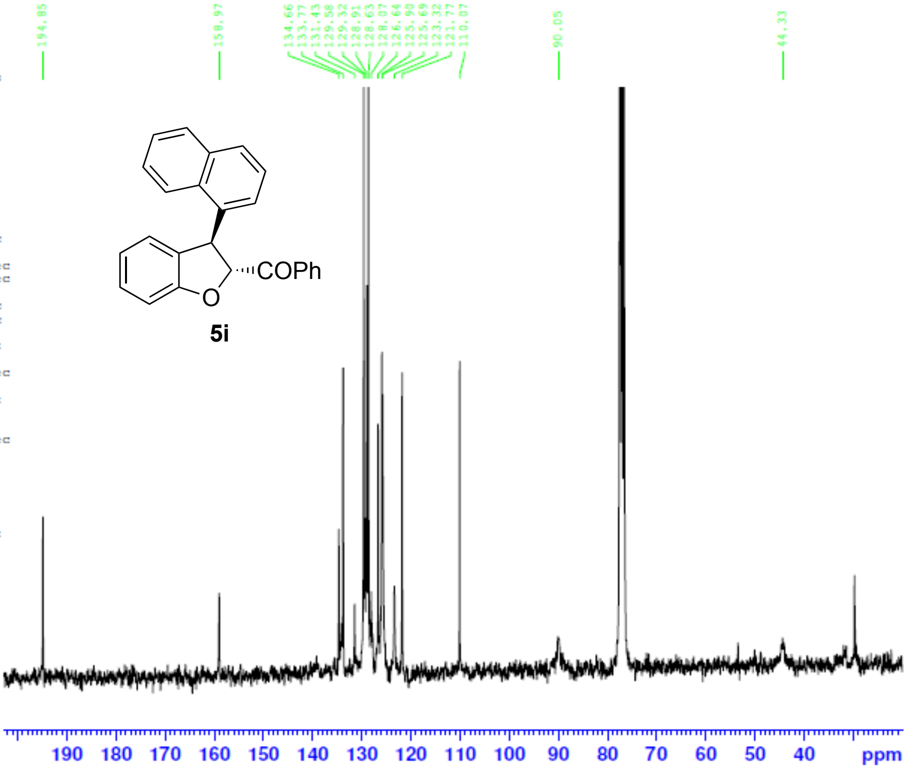

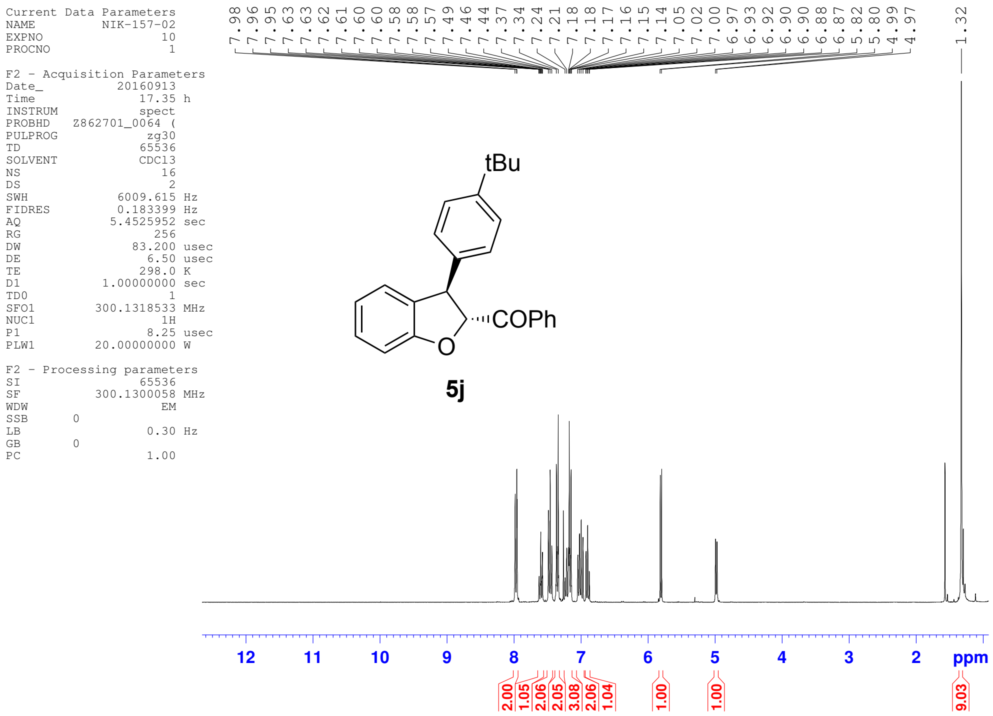
Current Data Parameters
NAME NIK-157-02
EXPNO 10
PROCNO 1
F2 - Acquisition Parameters
Date_ 20160913
Time 17.35 h
INSTRUM spect
PROBHD Z862701_0064 (
PULPROG zg30
TD 65536
SOLVENT CDCl3
NS 16
DS 2
SWH 6009.615 Hz
FIDRES 0.183399 Hz
AQ 5.4525952 sec
RG 256
DW 83.200 usec
DE 6.50 usec
TE 298.0 K
D1 1.00000000 sec
TD0 1
SFO1 300.1318533 MHz
NUC1 1H
P1 8.25 usec
PLW1 20.00000000 W
F2 - Processing parameters
SI 65536
SF 300.1300058 MHz
WDW EM
SSB 0
LB 0.30 Hz
GB 0
PC 1.00
7.98 7.96 7.95 7.63 7.63 7.62 7.61 7.60 7.60 7.58 7.58 7.57 7.49 7.46 7.44 7.37 7.34 7.24 7.21 7.18 7.18 7.17 7.16 7.15 7.14 7.05 7.02 7.00 6.97 6.93 6.92 6.90 6.88 6.87 5.82 5.80 4.99 4.97 1.32
tBu
COPh
O
5j
12 11 10 9 8 7 6 5 4 3 2 ppm
2.00 1.05 2.06 2.05 3.08 2.06 1.04 1.00 1.00 9.03

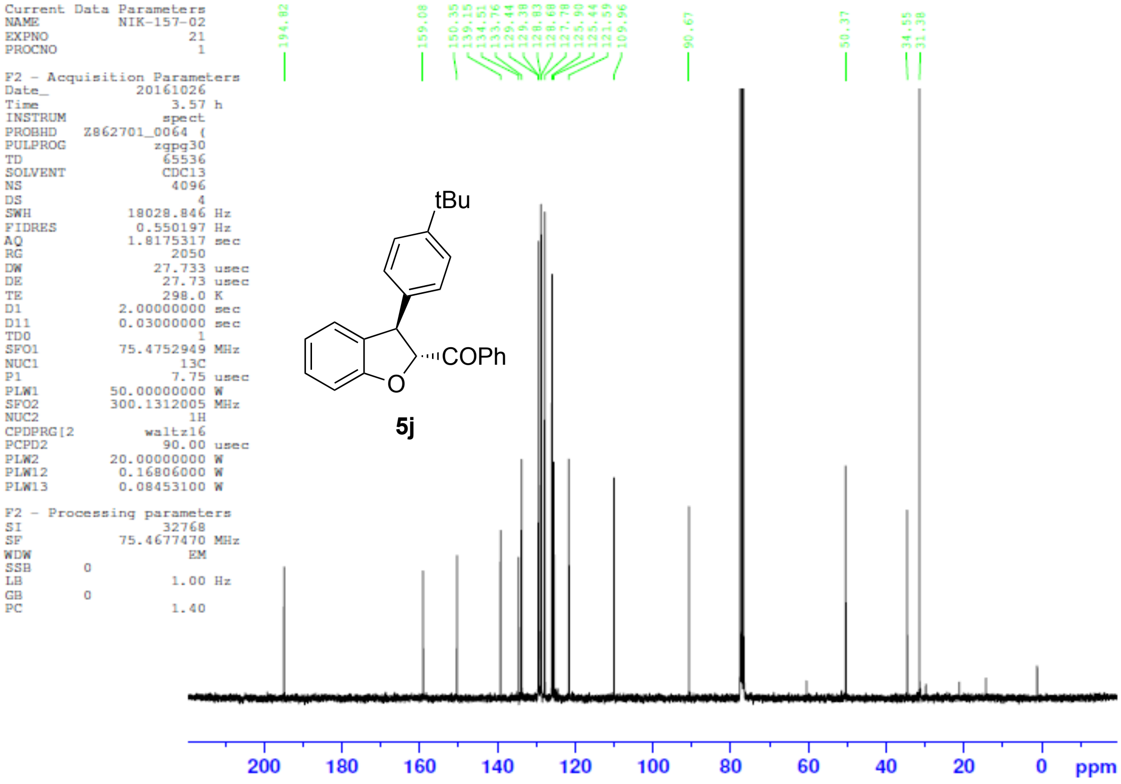
Current Data Parameters
NAME NIK-157-02
EXPNO 21
PROCNO 1
F2 - Acquisition Parameters
Date_ 20161026
Time 3.57 h
INSTRUM spect
PROBHD Z862701_0064 (
PULPROG zgpg30
TD 65536
SOLVENT CDCl3
NS 4096
DS 4
SWH 18028.846 Hz
FIDRES 0.550197 Hz
AQ 1.8175317 sec
RG 2050
DW 27.733 usec
DE 27.73 usec
TE 298.0 K
D1 2.00000000 sec
D11 0.03000000 sec
TD0 1
SFO1 75.4752949 MHz
NUC1 13C
P1 7.75 usec
PLW1 50.00000000 W
SFO2 300.1312005 MHz
NUC2 1H
CPDPRG[2 waltz16
PCPD2 90.00 usec
PLW2 20.00000000 W
PLW12 0.16806000 W
PLW13 0.08453100 W
F2 - Processing parameters
SI 32768
SF 75.4677470 MHz
WDW EM
SSB 0
LB 1.00 Hz
GB 0
PC 1.40
194.82 159.08 150.35 139.15 134.51 133.76 129.44 129.38 128.83 128.68 127.78 125.90 125.44 121.59 109.96 90.67 50.37 34.55 31.38
tBu
COPh
O
5j
200 180 160 140 120 100 80 60 40 20 0 ppm

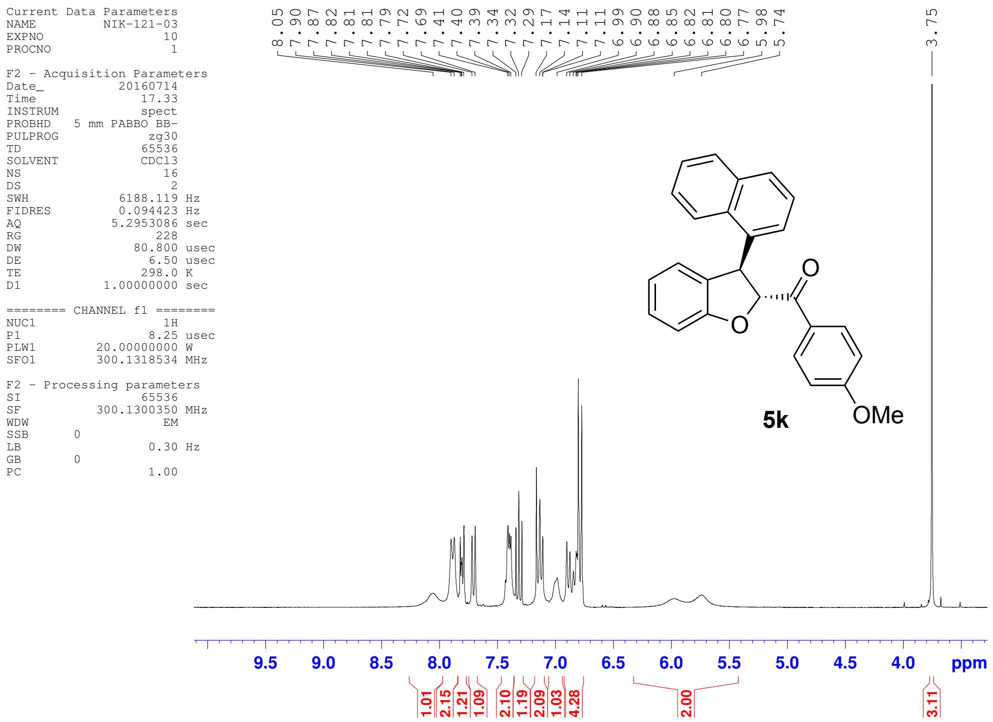
Current Data Parameters
NAME NIK-121-03
EXPNO 10
PROCNO 1
F2 - Acquisition Parameters
Date_ 20160714
Time 17.33
INSTRUM spect
PROBHD 5 mm PABBO BB-
PULPROG zg30
TD 65536
SOLVENT CDCl3
NS 16
DS 2
SWH 6188.119 Hz
FIDRES 0.094423 Hz
AQ 5.2953086 sec
RG 228
DW 80.800 usec
DE 6.50 usec
TE 298.0 K
D1 1.00000000 sec
======== CHANNEL f1 ========
NUC1 1H
P1 8.25 usec
PLW1 20.00000000 W
SFO1 300.1318534 MHz
F2 - Processing parameters
SI 65536
SF 300.1300350 MHz
WDW EM
SSB 0
LB 0.30 Hz
GB 0
PC 1.00
8.05 7.90 7.87 7.82 7.81 7.81 7.79 7.72 7.69 7.41 7.40 7.39 7.34 7.32 7.29 7.17 7.14 7.11 7.11 6.99 6.90 6.88 6.85 6.82 6.81 6.80 6.77 5.98 5.74
3.75
O
O
5k
OMe
9.5 9.0 8.5 8.0 7.5 7.0 6.5 6.0 5.5 5.0 4.5 4.0 ppm
1.01 2.15 1.21 1.09 2.10 1.19 2.09 1.03 4.28 2.00 3.11

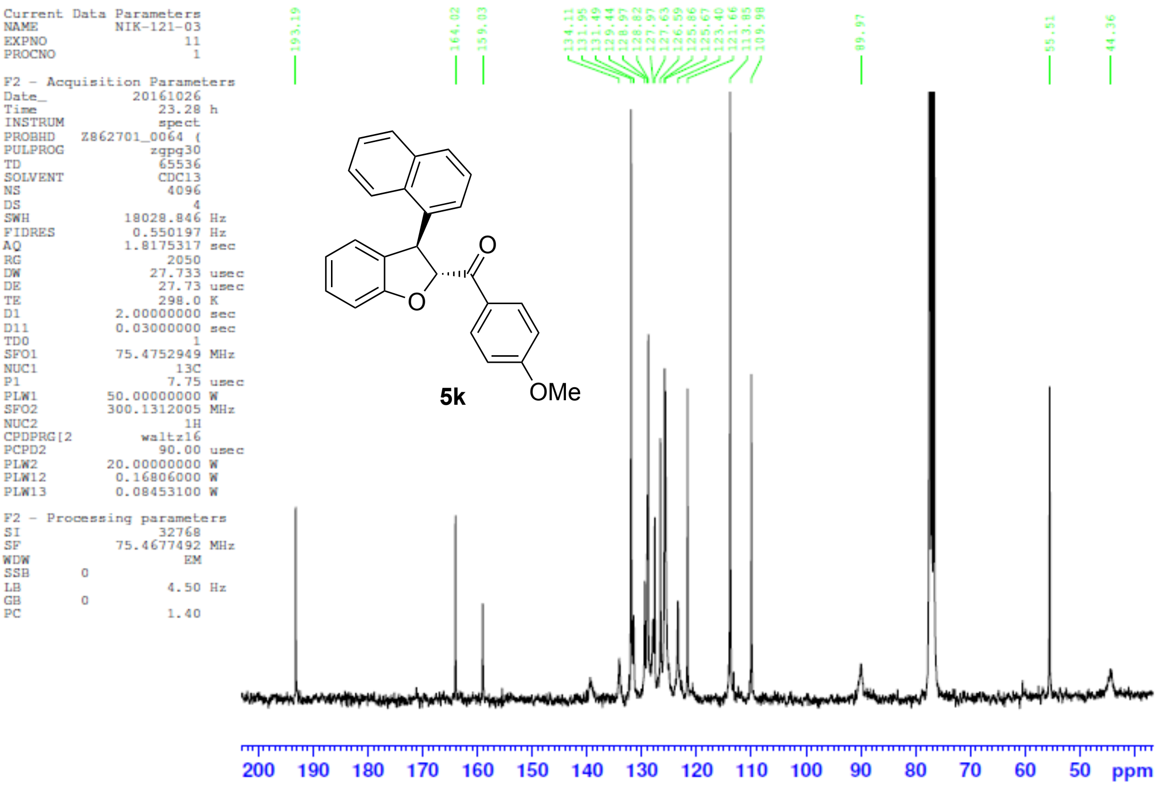
Current Data Parameters
NAME NIK-121-03
EXPNO 11
PROCNO 1
F2 - Acquisition Parameters
Date_ 20161026
Time 23.28 h
INSTRUM spect
PROBHD Z862701_0064 (
PULPROG zgpg30
TD 65536
SOLVENT CDCl3
NS 4096
DS 4
SWH 18028.846 Hz
FIDRES 0.550197 Hz
AQ 1.8175317 sec
RG 2050
DW 27.733 usec
DE 27.73 usec
TE 298.0 K
D1 2.00000000 sec
D11 0.03000000 sec
TD0 1
SFO1 75.4752949 MHz
NUC1 13C
P1 7.75 usec
PLW1 50.00000000 W
SFO2 300.1312005 MHz
NUC2 1H
CPDPRG[2 waltz16
PCPD2 90.00 usec
PLW2 20.00000000 W
PLW12 0.16806000 W
PLW13 0.08453100 W
F2 - Processing parameters
SI 32768
SF 75.4677492 MHz
WDW EM
SSB 0
LB 4.50 Hz
GB 0
PC 1.40
193.19
164.02 159.03
134.11 131.95 131.49 129.44 128.97 128.82 127.97 127.65 126.59 125.86 125.67 123.40 121.66 113.85 109.98
89.97
55.51
44.36
O
O
5k
OMe
200 190 180 170 160 150 140 130 120 110 100 90 80 70 60 50 ppm

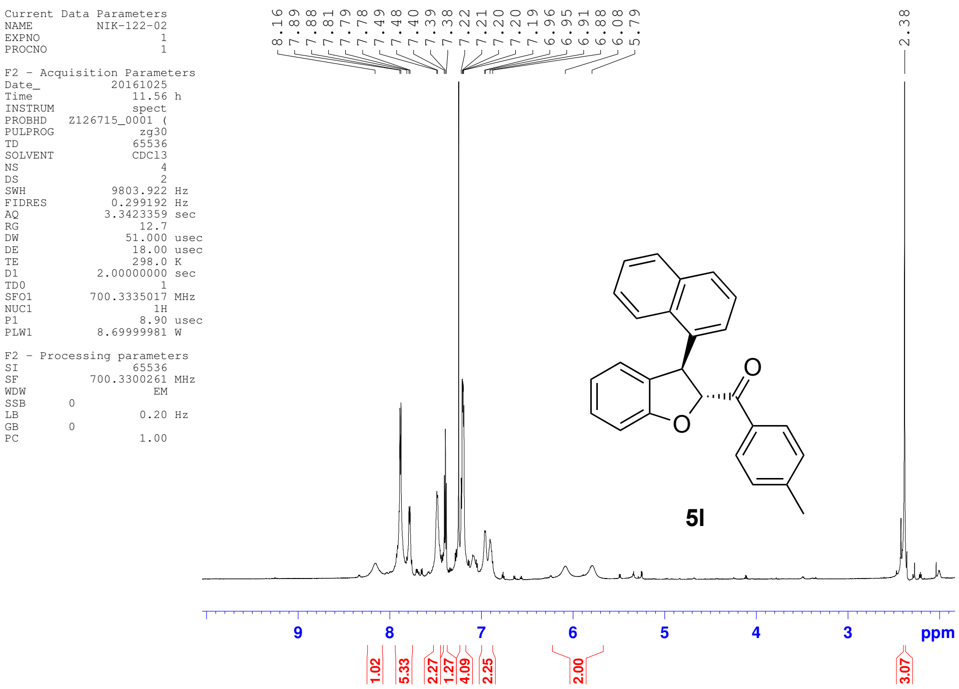
Current Data Parameters
NAME NIK-122-02
EXPNO 1
PROCNO 1
F2 - Acquisition Parameters
Date_ 20161025
Time 11.56 h
INSTRUM spect
PROBHD Z126715_0001 (
PULPROG zg30
TD 65536
SOLVENT CDCl3
NS 4
DS 2
SWH 9803.922 Hz
FIDRES 0.299192 Hz
AQ 3.3423359 sec
RG 12.7
DW 51.000 usec
DE 18.00 usec
TE 298.0 K
D1 2.00000000 sec
TD0 1
SFO1 700.3335017 MHz
NUC1 1H
P1 8.90 usec
PLW1 8.69999981 W
F2 - Processing parameters
SI 65536
SF 700.3300261 MHz
WDW EM
SSB 0
LB 0.20 Hz
GB 0
PC 1.00
8.16
7.89
7.88
7.81
7.79
7.78
7.49
7.48
7.40
7.39
7.38
7.22
7.21
7.20
7.20
7.19
6.96
6.95
6.91
6.88
6.08
5.79
2.38
O
O
5l
9
8
7
6
5
4
3
ppm
1.02
5.33
2.27
1.27
4.09
2.25
2.00
3.07

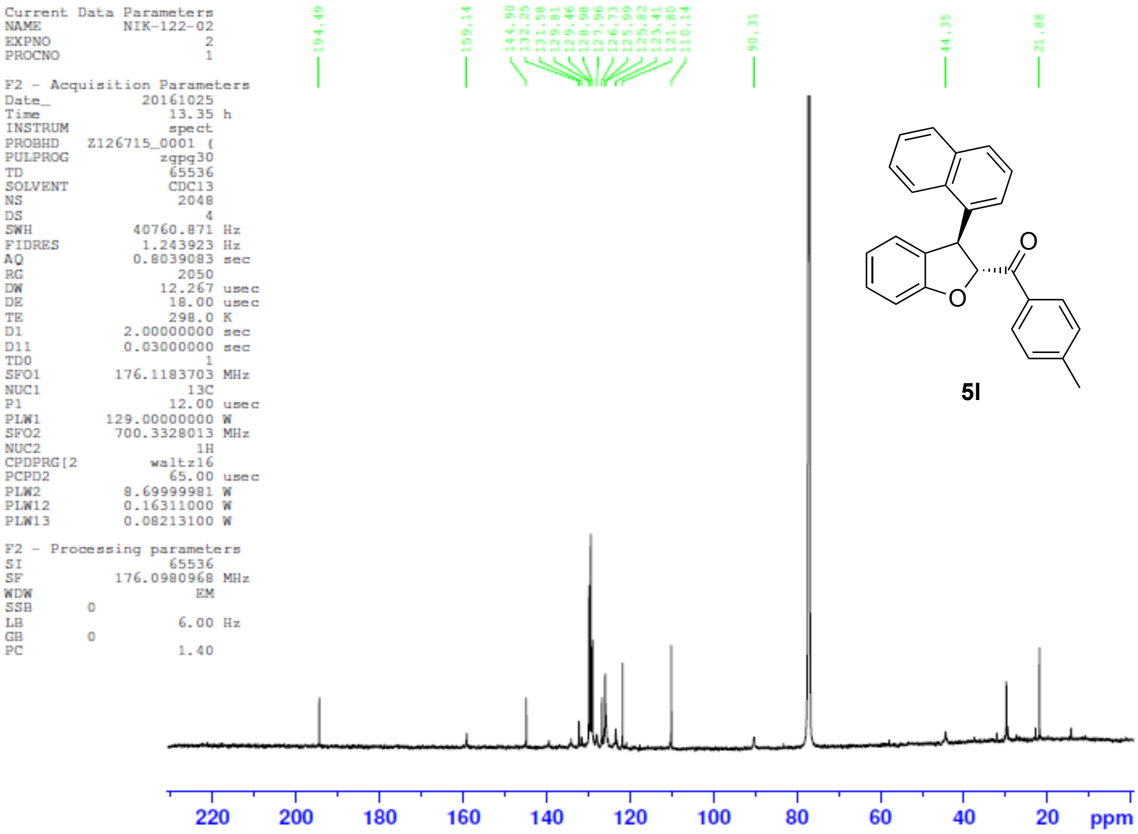
Current Data Parameters
NAME NIK-122-02
EXPNO 2
PROCNO 1
F2 - Acquisition Parameters
Date_ 20161025
Time 13.35 h
INSTRUM spect
PROBHD Z126715_0001 (
PULPROG zgpg30
TD 65536
SOLVENT CDCl3
NS 2048
DS 4
SWH 40760.871 Hz
FIDRES 1.243923 Hz
AQ 0.8039083 sec
RG 2050
DW 12.267 usec
DE 18.00 usec
TE 298.0 K
D1 2.00000000 sec
D11 0.03000000 sec
TD0 1
SFO1 176.1183703 MHz
NUC1 13C
P1 12.00 usec
PLW1 129.00000000 W
SFO2 700.3328013 MHz
NUC2 1H
CPDPRG[2 waltz16
PCPD2 65.00 usec
PLW2 8.69999981 W
PLW12 0.16311000 W
PLW13 0.08213100 W
F2 - Processing parameters
SI 65536
SF 176.0980968 MHz
WDW EM
SSB 0
LB 6.00 Hz
GB 0
PC 1.40
194.49
159.14
144.90
132.25
131.58
129.81
129.46
128.98
127.96
126.73
125.99
125.82
123.41
121.80
110.14
90.31
44.35
21.88
O
O
5l
220
200
180
160
140
120
100
80
60
40
20
ppm

```
Current Data Parameters
NAME          NIK-143-02
EXPNO                 10
PROCNO                 1

F2 - Acquisition Parameters
Date_           20160802
Time               13.41
INSTRUM            spect
PROBHD   5 mm PABBO BB-
PULPROG             zg30
TD                 65536
SOLVENT            CDCl3
NS                    16
DS                     2
SWH             6188.119 Hz
FIDRES          0.094423 Hz
AQ             5.2953086 sec
RG                   256
DW                80.800 usec
DE                  6.50 usec
TE                 298.0 K
D1            1.00000000 sec

======== CHANNEL f1 ========
NUC1                  1H
P1                  8.25 usec
PLW1         20.00000000 W
SFO1         300.1318534 MHz

F2 - Processing parameters
SI                 65536
SF           300.1300515 MHz
WDW                   EM
SSB      0
LB                  0.30 Hz
GB       0
PC                  1.00
```

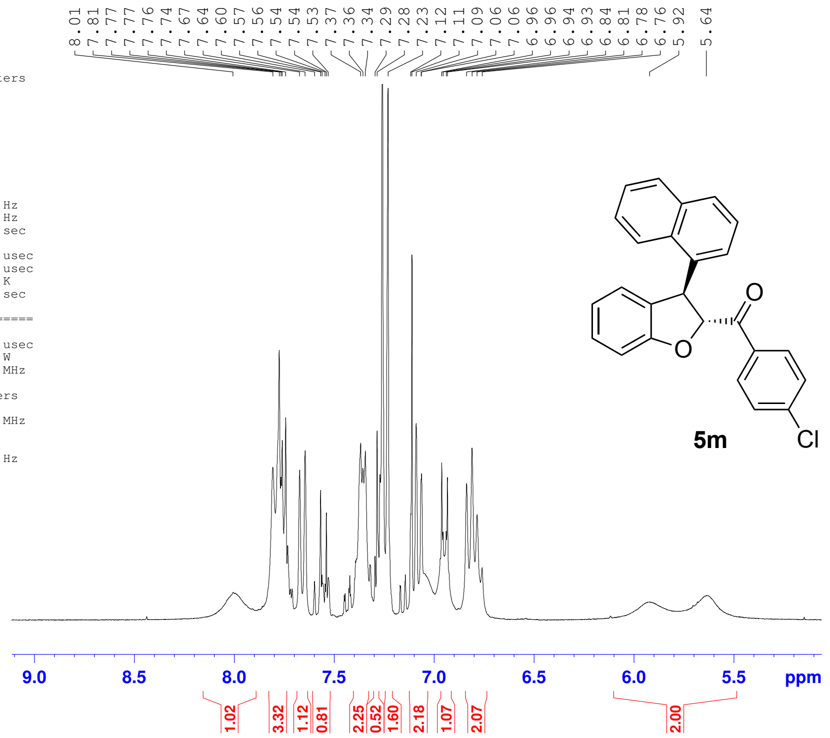


```
Current Data Parameters
NAME          NIK-143-02
EXPNO                 11
PROCNO                 1

F2 - Acquisition Parameters
Date_           20161027
Time                3.57 h
INSTRUM            spect
PROBHD   Z862701_0064 (
PULPROG          zgpg30
TD                 65536
SOLVENT            CDCl3
NS                  4096
DS                     4
SWH            18028.846 Hz
FIDRES          0.550197 Hz
AQ             1.8175317 sec
RG                  2050
DW                27.733 usec
DE                 27.73 usec
TE                 298.0 K
D1            2.00000000 sec
D11           0.03000000 sec
TD0                    1
SFO1          75.4752949 MHz
NUC1                 13C
P1                  7.75 usec
PLW1         50.00000000 W
SFO2         300.1312005 MHz
NUC2                  1H
CPDPRG[2         waltz16
PCPD2              90.00 usec
PLW2         20.00000000 W
PLW12         0.16806000 W
PLW13         0.08453100 W

F2 - Processing parameters
SI                 32768
SF            75.4677471 MHz
WDW                   EM
SSB      0
LB                  5.50 Hz
GB       0
PC                  1.40
```

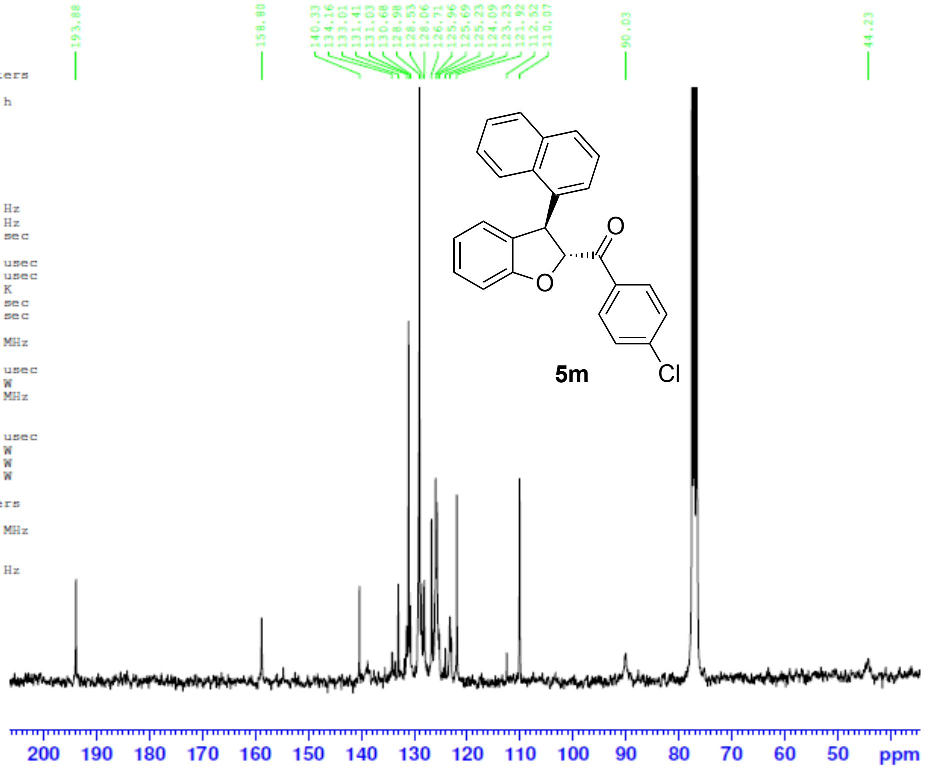

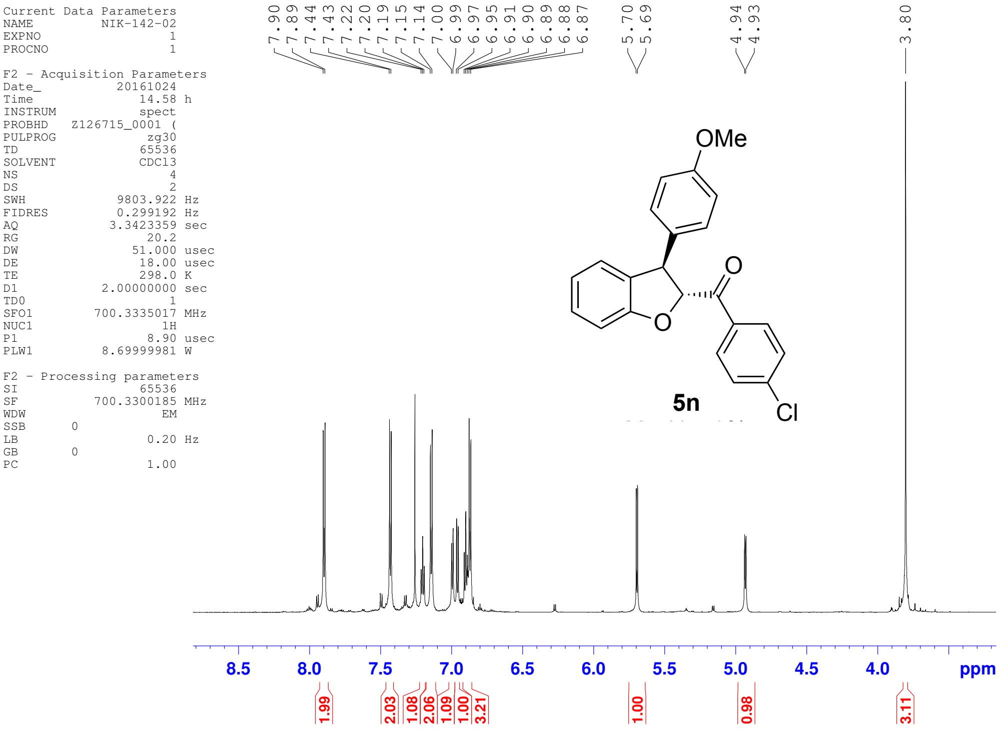
Current Data Parameters
NAME NIK-142-02
EXPNO 1
PROCNO 1
F2 - Acquisition Parameters
Date_ 20161024
Time 14.58 h
INSTRUM spect
PROBHD Z126715_0001 (
PULPROG zg30
TD 65536
SOLVENT CDCl3
NS 4
DS 2
SWH 9803.922 Hz
FIDRES 0.299192 Hz
AQ 3.3423359 sec
RG 20.2
DW 51.000 usec
DE 18.00 usec
TE 298.0 K
D1 2.00000000 sec
TD0 1
SFO1 700.3335017 MHz
NUC1 1H
P1 8.90 usec
PLW1 8.69999981 W
F2 - Processing parameters
SI 65536
SF 700.3300185 MHz
WDW EM
SSB 0
LB 0.20 Hz
GB 0
PC 1.00
7.90
7.89
7.44
7.43
7.22
7.20
7.19
7.15
7.14
7.00
6.99
6.97
6.95
6.91
6.90
6.89
6.88
6.87
5.70
5.69
4.94
4.93
3.80
OMe
O
O
5n
Cl
8.5
8.0
7.5
7.0
6.5
6.0
5.5
5.0
4.5
4.0
ppm
1.99
2.03
1.08
2.06
1.09
1.00
3.21
1.00
0.98
3.11

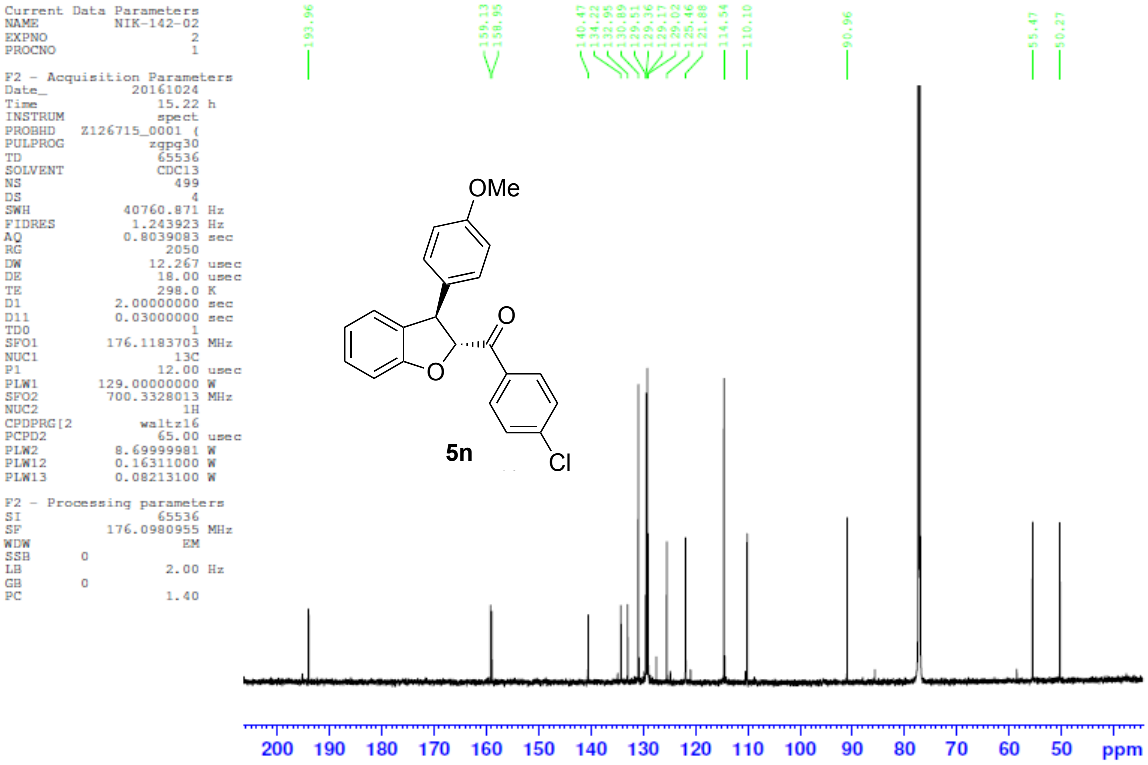
Current Data Parameters
NAME NIK-142-02
EXPNO 2
PROCNO 1
F2 - Acquisition Parameters
Date_ 20161024
Time 15.22 h
INSTRUM spect
PROBHD Z126715_0001 (
PULPROG zgpg30
TD 65536
SOLVENT CDCl3
NS 499
DS 4
SWH 40760.871 Hz
FIDRES 1.243923 Hz
AQ 0.8039083 sec
RG 2050
DW 12.267 usec
DE 18.00 usec
TE 298.0 K
D1 2.00000000 sec
D11 0.03000000 sec
TD0 1
SFO1 176.1183703 MHz
NUC1 13C
P1 12.00 usec
PLW1 129.00000000 W
SFO2 700.3328013 MHz
NUC2 1H
CPDPRG[2 waltz16
PCPD2 65.00 usec
PLW2 8.69999981 W
PLW12 0.16311000 W
PLW13 0.08213100 W
F2 - Processing parameters
SI 65536
SF 176.0980955 MHz
WDW EM
SSB 0
LB 2.00 Hz
GB 0
PC 1.40
193.96
159.13
158.95
140.47
134.22
132.95
130.89
129.51
129.36
129.17
129.02
125.46
121.88
114.54
110.10
90.96
55.47
50.27
OMe
O
O
5n
Cl
200
190
180
170
160
150
140
130
120
110
100
90
80
70
60
50
ppm

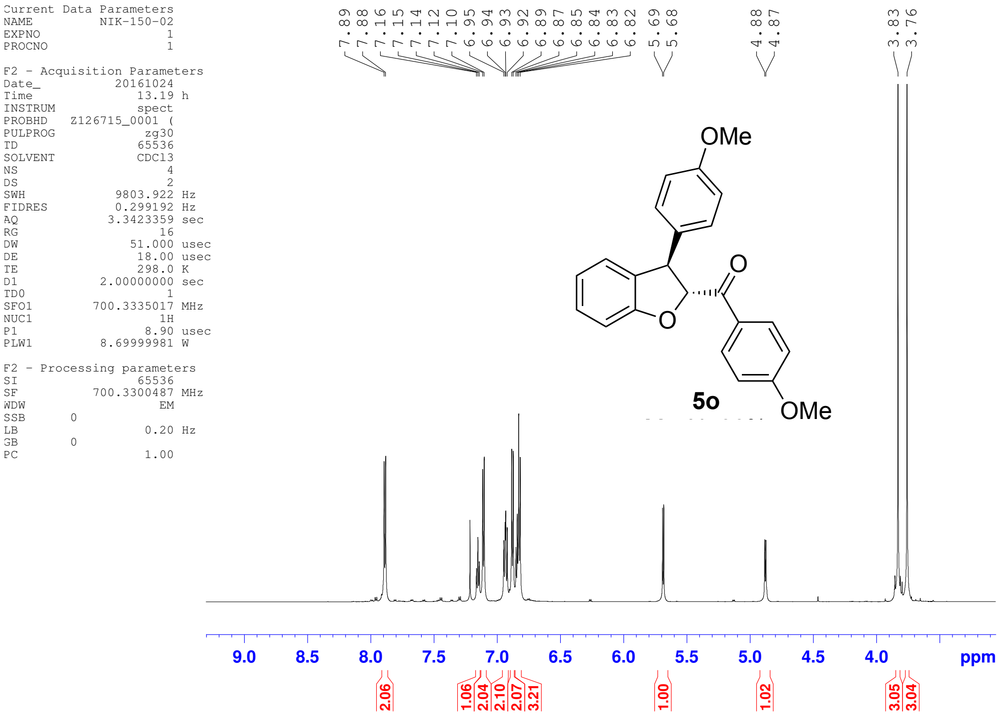
Current Data Parameters
NAME NIK-150-02
EXPNO 1
PROCNO 1
F2 - Acquisition Parameters
Date_ 20161024
Time 13.19 h
INSTRUM spect
PROBHD Z126715_0001 (
PULPROG zg30
TD 65536
SOLVENT CDCl3
NS 4
DS 2
SWH 9803.922 Hz
FIDRES 0.299192 Hz
AQ 3.3423359 sec
RG 16
DW 51.000 usec
DE 18.00 usec
TE 298.0 K
D1 2.00000000 sec
TD0 1
SFO1 700.3335017 MHz
NUC1 1H
P1 8.90 usec
PLW1 8.69999981 W
F2 - Processing parameters
SI 65536
SF 700.3300487 MHz
WDW EM
SSB 0
LB 0.20 Hz
GB 0
PC 1.00
7.89 7.88 7.16 7.15 7.14 7.12 7.10 6.95 6.94 6.93 6.92 6.89 6.87 6.85 6.84 6.83 6.82 5.69 5.68 4.88 4.87 3.83 3.76
OMe
O
O
5o
OMe
9.0 8.5 8.0 7.5 7.0 6.5 6.0 5.5 5.0 4.5 4.0 ppm
2.06 1.06 2.04 2.10 2.07 3.21 1.00 1.02 3.05 3.04

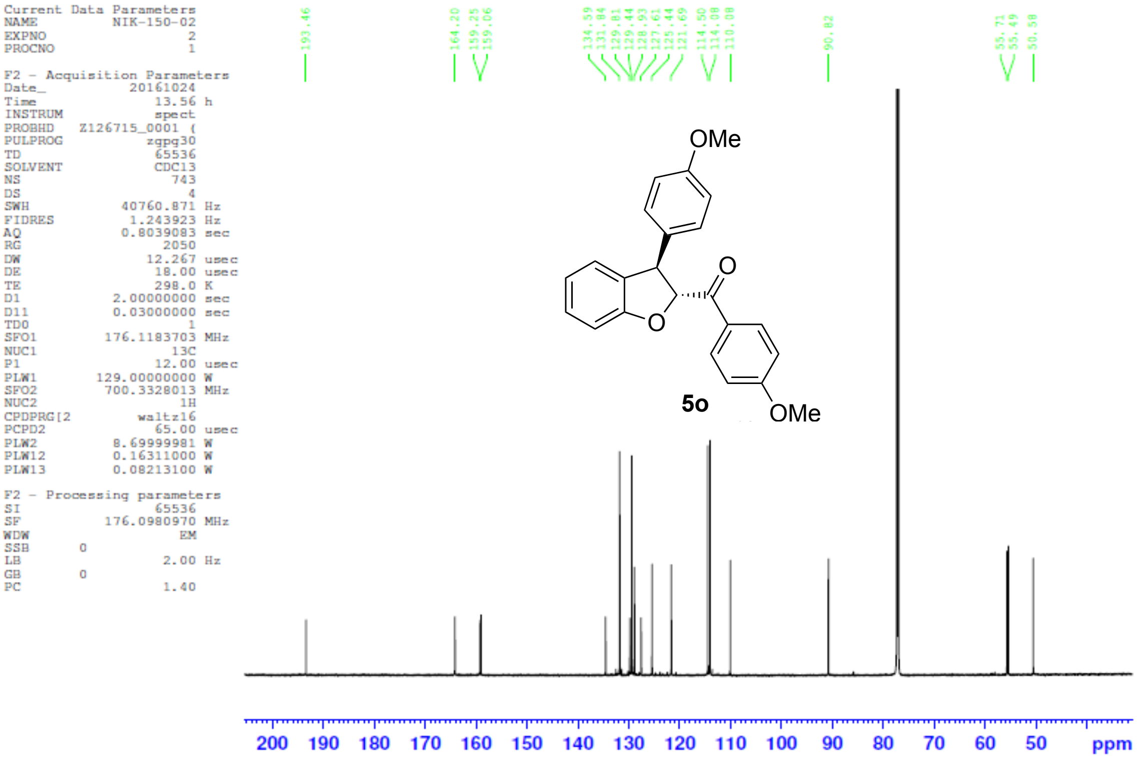
Current Data Parameters
NAME NIK-150-02
EXPNO 2
PROCNO 1
F2 - Acquisition Parameters
Date_ 20161024
Time 13.56 h
INSTRUM spect
PROBHD Z126715_0001 (
PULPROG zgpg30
TD 65536
SOLVENT CDCl3
NS 743
DS 4
SWH 40760.871 Hz
FIDRES 1.243923 Hz
AQ 0.8039083 sec
RG 2050
DW 12.267 usec
DE 18.00 usec
TE 298.0 K
D1 2.00000000 sec
D11 0.03000000 sec
TD0 1
SFO1 176.1183703 MHz
NUC1 13C
P1 12.00 usec
PLW1 129.00000000 W
SFO2 700.3328013 MHz
NUC2 1H
CPDPRG[2 waltz16
PCPD2 65.00 usec
PLW2 8.69999981 W
PLW12 0.16311000 W
PLW13 0.08213100 W
F2 - Processing parameters
SI 65536
SF 176.0980970 MHz
WDW EM
SSB 0
LB 2.00 Hz
GB 0
PC 1.40
193.46 164.20 159.25 159.06 134.59 131.84 129.81 129.44 128.93 127.61 125.44 121.69 114.50 114.09 110.08 90.82 55.71 55.49 50.58
OMe
O
O
5o
OMe
200 190 180 170 160 150 140 130 120 110 100 90 80 70 60 50 ppm

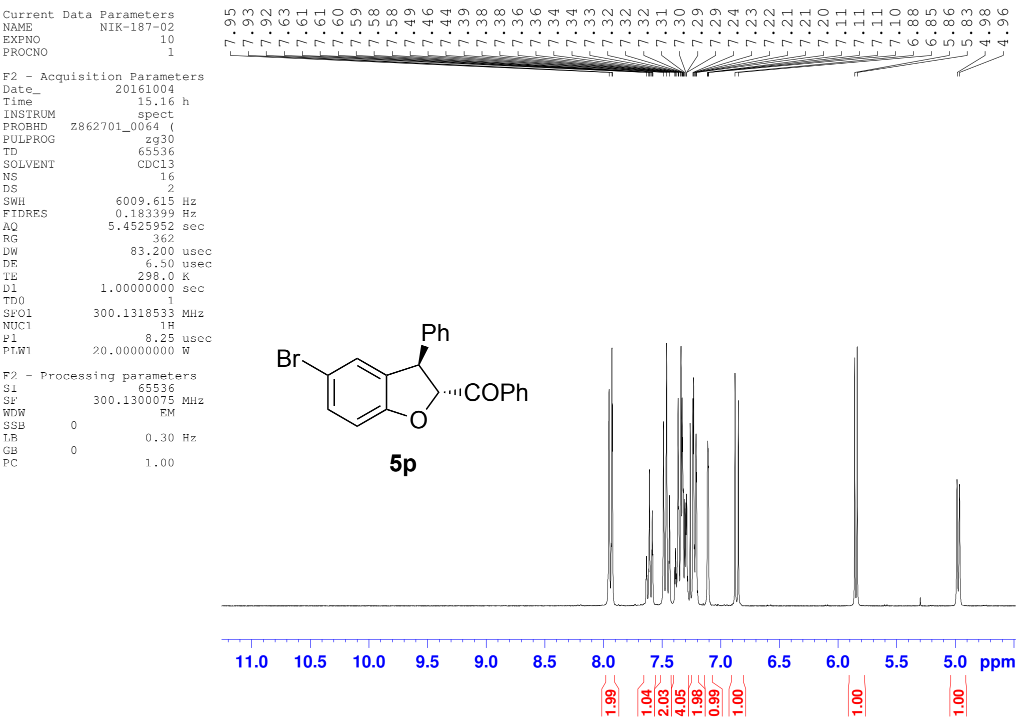
Current Data Parameters
NAME NIK-187-02
EXPNO 10
PROCNO 1
F2 - Acquisition Parameters
Date_ 20161004
Time 15.16 h
INSTRUM spect
PROBHD Z862701_0064 (
PULPROG zg30
TD 65536
SOLVENT CDCl3
NS 16
DS 2
SWH 6009.615 Hz
FIDRES 0.183399 Hz
AQ 5.4525952 sec
RG 362
DW 83.200 usec
DE 6.50 usec
TE 298.0 K
D1 1.00000000 sec
TD0 1
SFO1 300.1318533 MHz
NUC1 1H
P1 8.25 usec
PLW1 20.00000000 W
F2 - Processing parameters
SI 65536
SF 300.1300075 MHz
WDW EM
SSB 0
LB 0.30 Hz
GB 0
PC 1.00
7.95 7.93 7.92 7.63 7.61 7.61 7.60 7.59 7.58 7.58 7.49 7.46 7.44 7.39 7.38 7.38 7.36 7.36 7.34 7.34 7.33 7.32 7.32 7.32 7.31 7.30 7.29 7.29 7.24 7.23 7.22 7.21 7.21 7.20 7.11 7.11 7.11 7.10 6.88 6.85 5.86 5.83 4.98 4.96
Br
Ph
COPh
O
5p
11.0 10.5 10.0 9.5 9.0 8.5 8.0 7.5 7.0 6.5 6.0 5.5 5.0 ppm
1.99 1.04 2.03 4.05 1.98 0.99 1.00 1.00 1.00

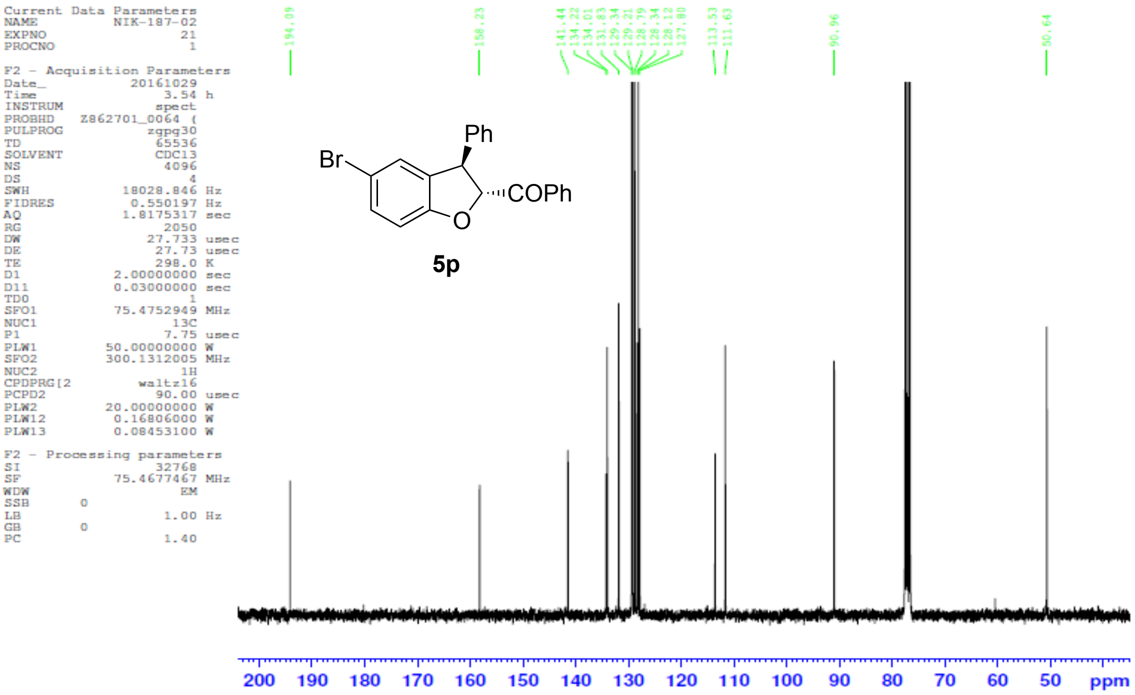
Current Data Parameters
NAME NIK-187-02
EXPNO 21
PROCNO 1
F2 - Acquisition Parameters
Date_ 20161029
Time 3.54 h
INSTRUM spect
PROBHD Z862701_0064 (
PULPROG zgpg30
TD 65536
SOLVENT CDCl3
NS 4096
DS 4
SWH 18028.846 Hz
FIDRES 0.550197 Hz
AQ 1.8175317 sec
RG 2050
DW 27.733 usec
DE 27.73 usec
TE 298.0 K
D1 2.00000000 sec
D11 0.03000000 sec
TD0 1
SFO1 75.4752949 MHz
NUC1 13C
P1 7.75 usec
PLW1 50.00000000 W
SFO2 300.1312005 MHz
NUC2 1H
CPDPRG[2 waltz16
PCPD2 90.00 usec
PLW2 20.00000000 W
PLW12 0.16806000 W
PLW13 0.08453100 W
F2 - Processing parameters
SI 32768
SF 75.4677467 MHz
WDW EM
SSB 0
LB 1.00 Hz
GB 0
PC 1.40
194.99 158.23 141.44 134.22 134.01 131.85 129.34 129.21 128.79 128.34 128.12 127.80 113.53 111.63 90.96 50.64
Br
Ph
COPh
O
5p
200 190 180 170 160 150 140 130 120 110 100 90 80 70 60 50 ppm

```
Current Data Parameters
NAME            NIK-171-02
EXPNO                    1
PROCNO                   1

F2 - Acquisition Parameters
Date_             20161024
Time                 15.28 h
INSTRUM              spect
PROBHD    Z126715_0001 (
PULPROG               zg30
TD                   65536
SOLVENT              CDCl3
NS                       4
DS                       2
SWH               9803.922 Hz
FIDRES            0.299192 Hz
AQ               3.3423359 sec
RG                       9
DW                  51.000 usec
DE                   18.00 usec
TE                   298.0 K
D1              2.00000000 sec
TD0                      1
SFO1          700.3335017 MHz
NUC1                    1H
P1                    8.90 usec
PLW1            8.69999981 W

F2 - Processing parameters
SI                   65536
SF            700.3300185 MHz
WDW                     EM
SSB       0
LB                    0.20 Hz
GB        0
PC                    1.00
```

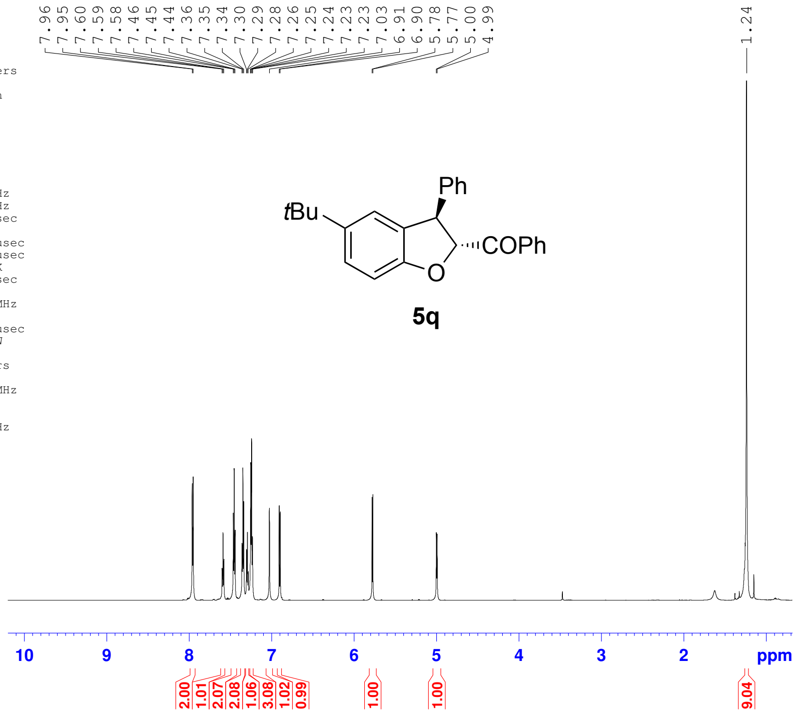


```
Current Data Parameters
NAME            NIK-171-02
EXPNO                    2
PROCNO                   1

F2 - Acquisition Parameters
Date_             20161024
Time                 16.39 h
INSTRUM              spect
PROBHD    Z126715_0001 (
PULPROG             zgpg30
TD                   65536
SOLVENT              CDCl3
NS                    1472
DS                       4
SWH              40760.871 Hz
FIDRES            1.243923 Hz
AQ               0.8039083 sec
RG                    2050
DW                  12.267 usec
DE                   18.00 usec
TE                   298.0 K
D1              2.00000000 sec
D11             0.03000000 sec
TD0                      1
SFO1          176.1183703 MHz
NUC1                   13C
P1                   12.00 usec
PLW1          129.00000000 W
SFO2          700.3328013 MHz
NUC2                    1H
CPDPRG[2           waltz16
PCPD2                65.00 usec
PLW2            8.69999981 W
PLW12           0.16311000 W
PLW13           0.08213100 W

F2 - Processing parameters
SI                   65536
SF            176.0980993 MHz
WDW                     EM
SSB       0
LB                    2.00 Hz
GB        0
PC                    1.40
```

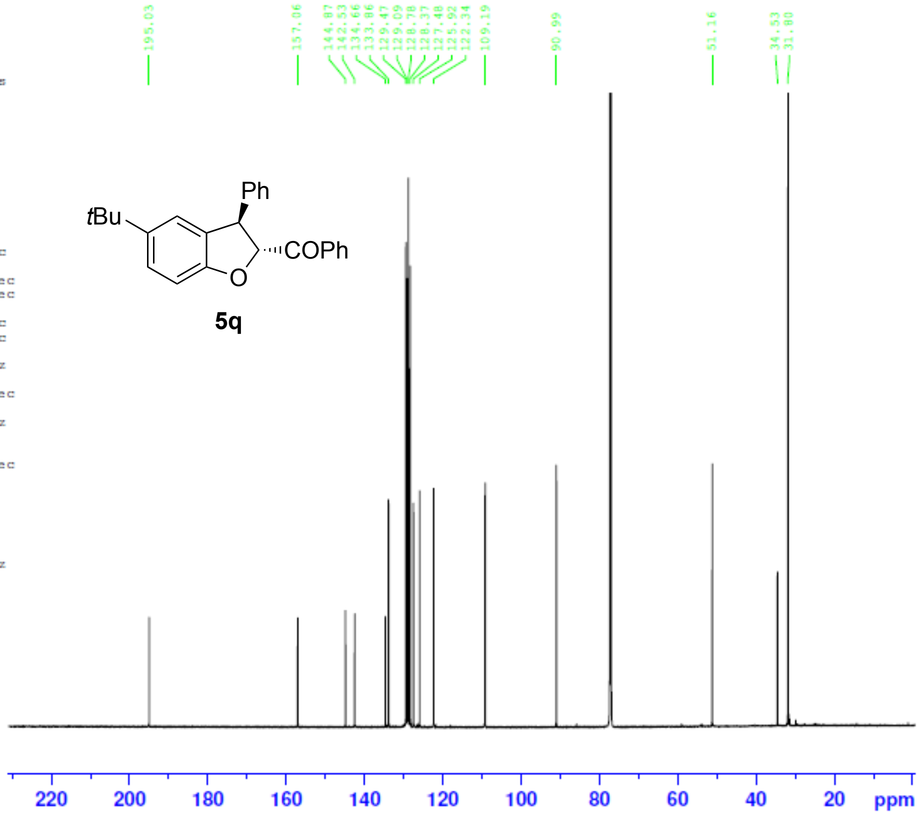

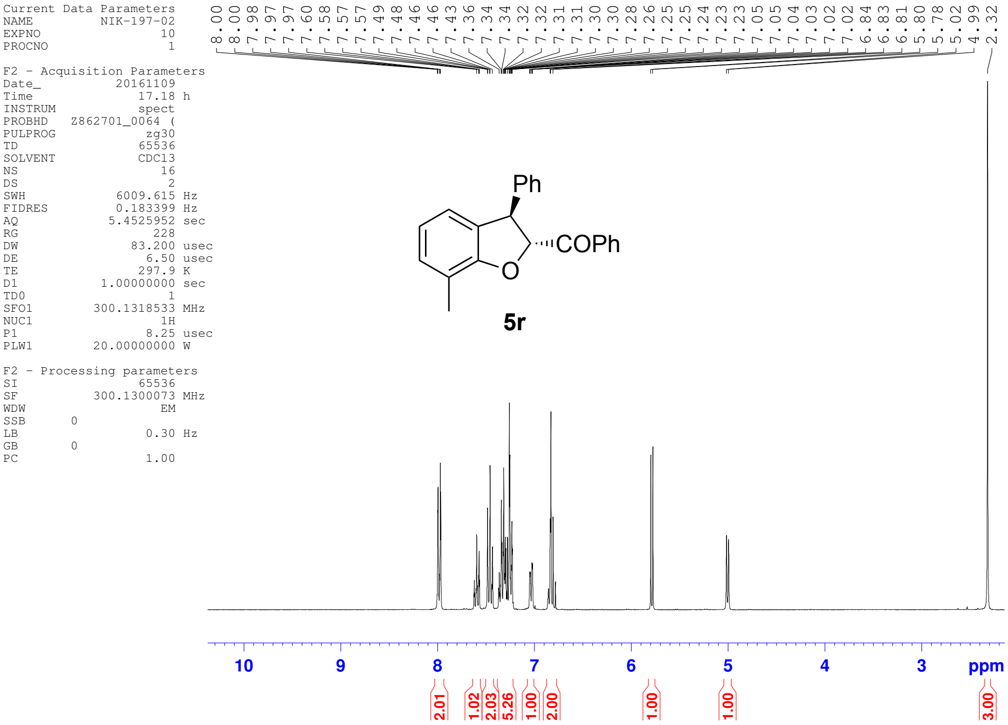
Current Data Parameters
NAME NIK-197-02
EXPNO 10
PROCNO 1
F2 - Acquisition Parameters
Date_ 20161109
Time 17.18 h
INSTRUM spect
PROBHD Z862701_0064 (
PULPROG zg30
TD 65536
SOLVENT CDCl3
NS 16
DS 2
SWH 6009.615 Hz
FIDRES 0.183399 Hz
AQ 5.4525952 sec
RG 228
DW 83.200 usec
DE 6.50 usec
TE 297.9 K
D1 1.00000000 sec
TD0 1
SFO1 300.1318533 MHz
NUC1 1H
P1 8.25 usec
PLW1 20.00000000 W
F2 - Processing parameters
SI 65536
SF 300.1300073 MHz
WDW EM
SSB 0
LB 0.30 Hz
GB 0
PC 1.00
8.00 8.00 7.98 7.97 7.97 7.60 7.58 7.57 7.57 7.49 7.48 7.46 7.46 7.43 7.36 7.34 7.34 7.32 7.32 7.31 7.31 7.30 7.30 7.28 7.26 7.25 7.25 7.24 7.23 7.23 7.05 7.05 7.04 7.03 7.02 7.02 6.84 6.83 6.81 5.80 5.78 5.02 4.99 2.32
Ph
COPh
O
5r
10 9 8 7 6 5 4 3 ppm
2.01 1.02 2.03 5.26 1.00 2.00 1.00 1.00 3.00

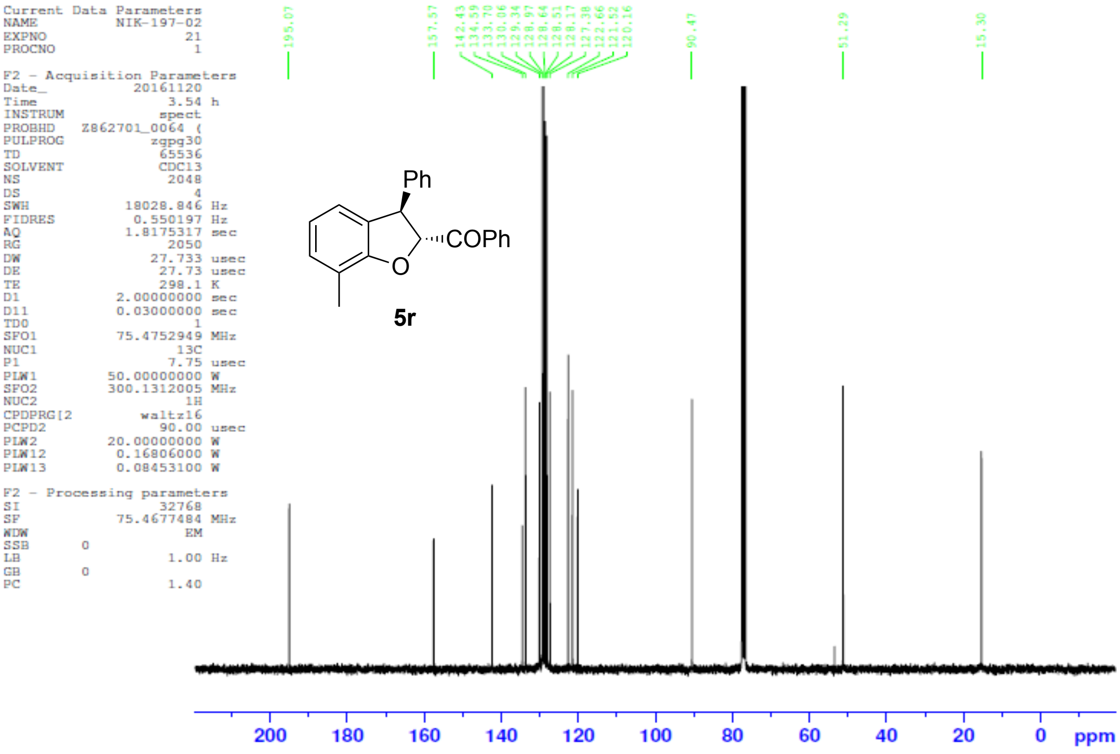
Current Data Parameters
NAME NIK-197-02
EXPNO 21
PROCNO 1
F2 - Acquisition Parameters
Date_ 20161120
Time 3.54 h
INSTRUM spect
PROBHD Z862701_0064 (
PULPROG zgpg30
TD 65536
SOLVENT CDCl3
NS 2048
DS 4
SWH 18028.846 Hz
FIDRES 0.550197 Hz
AQ 1.8175317 sec
RG 2050
DW 27.733 usec
DE 27.73 usec
TE 298.1 K
D1 2.00000000 sec
D11 0.03000000 sec
TD0 1
SFO1 75.4752949 MHz
NUC1 13C
P1 7.75 usec
PLW1 50.00000000 W
SFO2 300.1312005 MHz
NUC2 1H
CPDPRG[2 waltz16
PCPD2 90.00 usec
PLW2 20.00000000 W
PLW12 0.16806000 W
PLW13 0.08453100 W
F2 - Processing parameters
SI 32768
SF 75.4677484 MHz
WDW EM
SSB 0
LB 1.00 Hz
GB 0
PC 1.40
195.07 157.57 142.43 134.59 133.70 130.06 129.34 128.97 128.64 128.51 128.17 127.38 122.66 121.52 120.16 90.47 51.29 15.30
Ph
COPh
O
5r
200 180 160 140 120 100 80 60 40 20 0 ppm

```
Current Data Parameters
NAME              NIK-191-02
EXPNO                     10
PROCNO                     1

F2 - Acquisition Parameters
Date_               20161012
Time                   18.17 h
INSTRUM                spect
PROBHD    Z862701_0064 (
PULPROG                 zg30
TD                     65536
SOLVENT                CDCl3
NS                        16
DS                         2
SWH                 6009.615 Hz
FIDRES              0.183399 Hz
AQ                 5.4525952 sec
RG                       256
DW                    83.200 usec
DE                      6.50 usec
TE                     298.0 K
D1                1.00000000 sec
TD0                        1
SFO1             300.1318533 MHz
NUC1                      1H
P1                      8.25 usec
PLW1             20.00000000 W

F2 - Processing parameters
SI                     65536
SF               300.1300196 MHz
WDW                       EM
SSB      0
LB                      0.30 Hz
GB       0
PC                      1.00
```

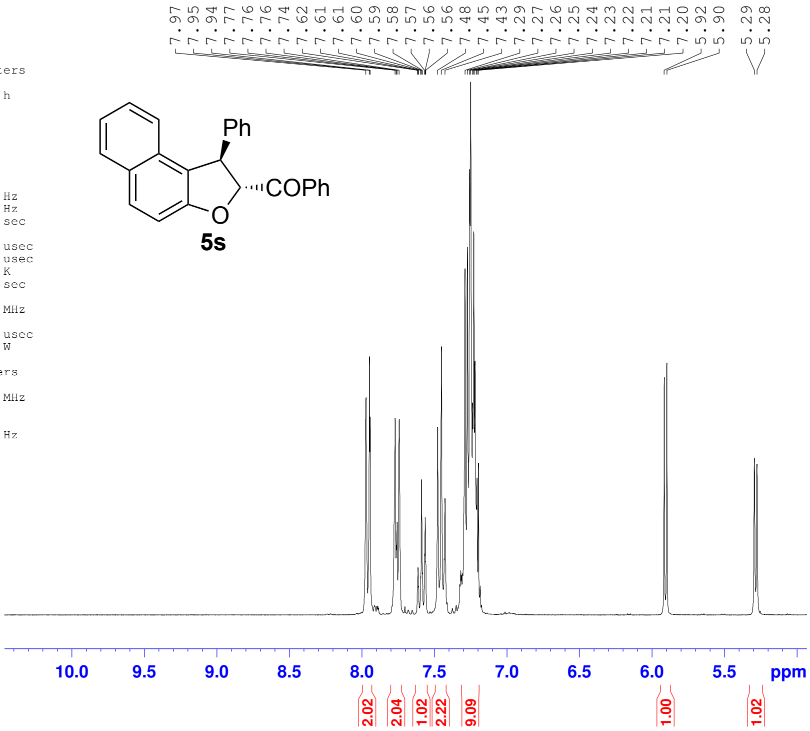


```
Current Data Parameters
NAME              NIK-191-02
EXPNO                     21
PROCNO                     1

F2 - Acquisition Parameters
Date_               20161027
Time                   23.29 h
INSTRUM                spect
PROBHD    Z862701_0064 (
PULPROG               zgpg30
TD                     65536
SOLVENT                CDCl3
NS                      4096
DS                         4
SWH                18028.846 Hz
FIDRES              0.550197 Hz
AQ                 1.8175317 sec
RG                      2050
DW                    27.733 usec
DE                     27.73 usec
TE                     297.9 K
D1                2.00000000 sec
D11               0.03000000 sec
TD0                        1
SFO1              75.4752949 MHz
NUC1                     13C
P1                      7.75 usec
PLW1             50.00000000 W
SFO2             300.1312005 MHz
NUC2                      1H
CPDPRG[2             waltz16
PCPD2                  90.00 usec
PLW2             20.00000000 W
PLW12             0.16806000 W
PLW13             0.08453100 W

F2 - Processing parameters
SI                     32768
SF                75.4677485 MHz
WDW                       EM
SSB      0
LB                      1.00 Hz
GB       0
PC                      1.40
```

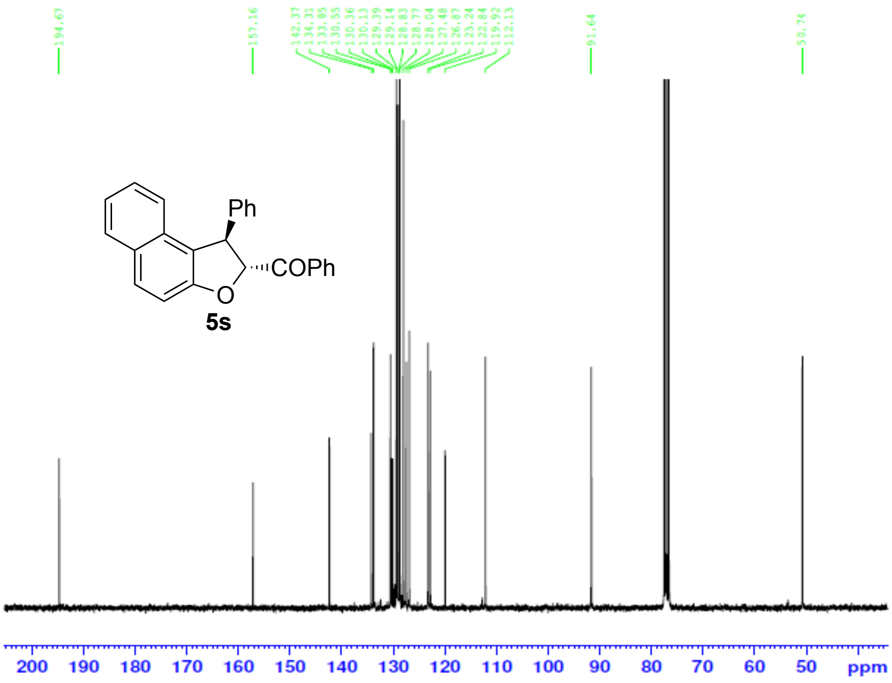

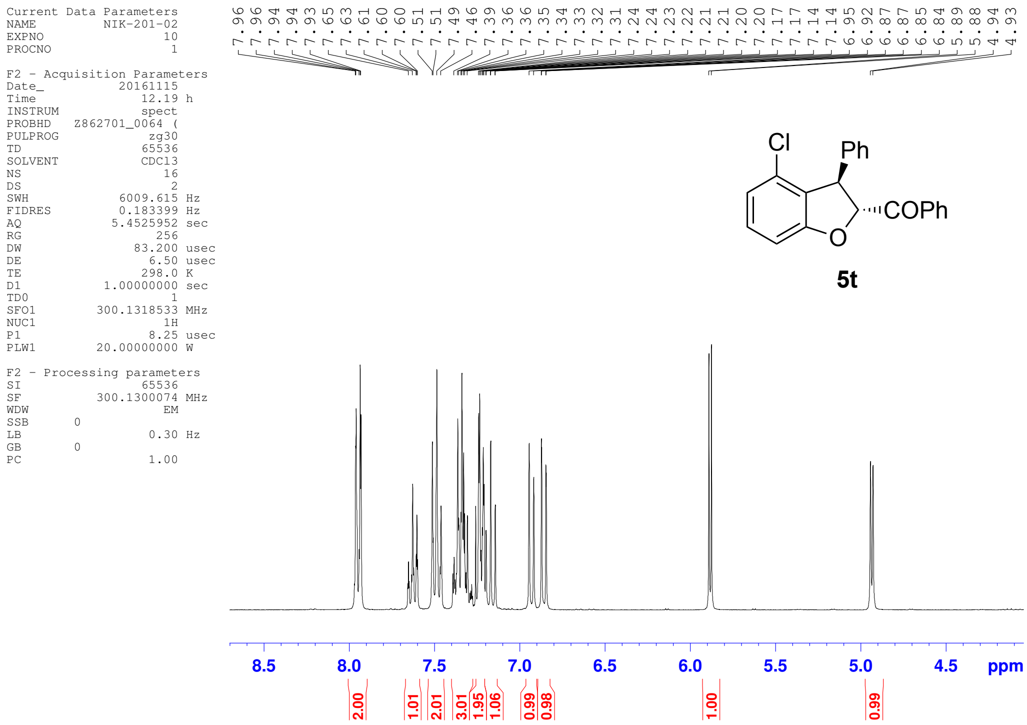
Current Data Parameters
NAME NIK-201-02
EXPNO 10
PROCNO 1
F2 - Acquisition Parameters
Date_ 20161115
Time 12.19 h
INSTRUM spect
PROBHD Z862701_0064 (
PULPROG zg30
TD 65536
SOLVENT CDCl3
NS 16
DS 2
SWH 6009.615 Hz
FIDRES 0.183399 Hz
AQ 5.4525952 sec
RG 256
DW 83.200 usec
DE 6.50 usec
TE 298.0 K
D1 1.00000000 sec
TD0 1
SFO1 300.1318533 MHz
NUC1 1H
P1 8.25 usec
PLW1 20.00000000 W
F2 - Processing parameters
SI 65536
SF 300.1300074 MHz
WDW EM
SSB 0
LB 0.30 Hz
GB 0
PC 1.00
7.96 7.96 7.94 7.94 7.93 7.65 7.63 7.61 7.60 7.51 7.51 7.49 7.46 7.39 7.36 7.36 7.35 7.34 7.33 7.32 7.31 7.24 7.24 7.23 7.22 7.21 7.21 7.20 7.20 7.17 7.17 7.14 7.14 6.95 6.92 6.87 6.87 6.85 6.84 5.89 5.88 4.94 4.93
Cl
Ph
COPh
O
5t
8.5 8.0 7.5 7.0 6.5 6.0 5.5 5.0 4.5 ppm
2.00 1.01 2.01 3.01 1.95 1.06 0.99 0.98 1.00 0.99

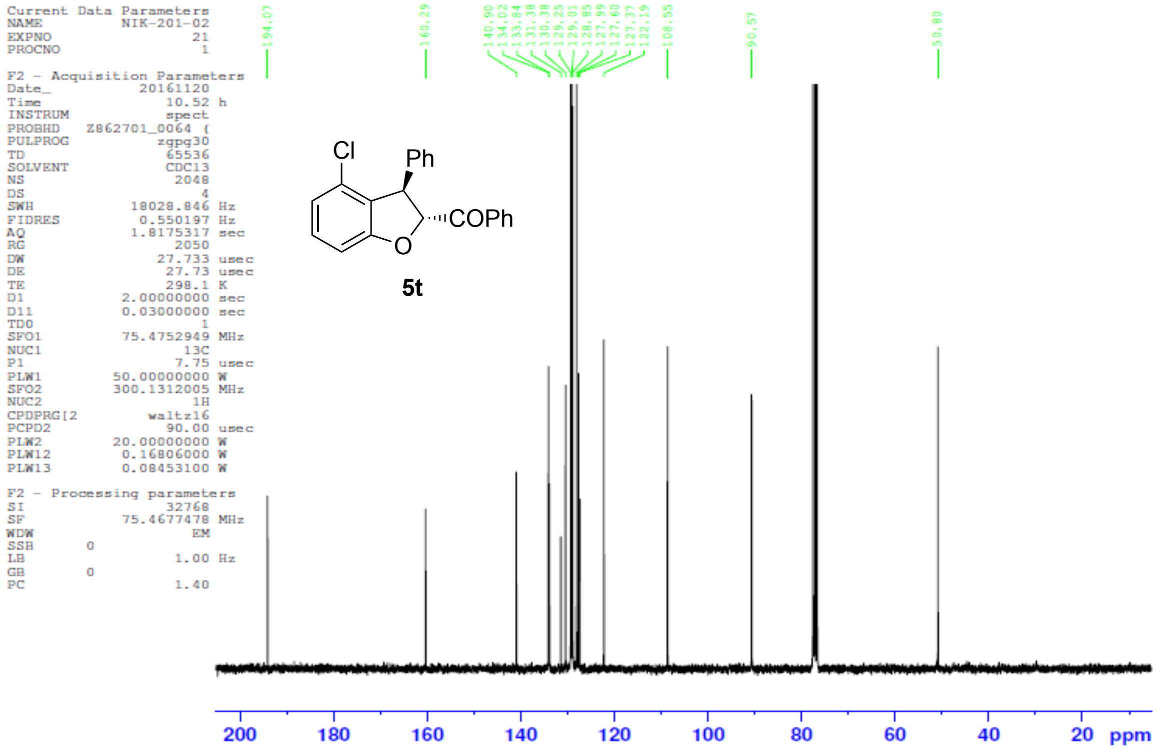
Current Data Parameters
NAME NIK-201-02
EXPNO 21
PROCNO 1
F2 - Acquisition Parameters
Date_ 20161120
Time 10.52 h
INSTRUM spect
PROBHD Z862701_0064 (
PULPROG zgpg30
TD 65536
SOLVENT CDCl3
NS 2048
DS 4
SWH 18028.846 Hz
FIDRES 0.550197 Hz
AQ 1.8175317 sec
RG 2050
DW 27.733 usec
DE 27.73 usec
TE 298.1 K
D1 2.00000000 sec
D11 0.03000000 sec
TD0 1
SFO1 75.4752949 MHz
NUC1 13C
P1 7.75 usec
PLW1 50.00000000 W
SFO2 300.1312005 MHz
NUC2 1H
CPDPRG[2 waltz16
PCPD2 90.00 usec
PLW2 20.00000000 W
PLW12 0.16806000 W
PLW13 0.08453100 W
F2 - Processing parameters
SI 32768
SF 75.4677478 MHz
WDW EM
SSB 0
LB 1.00 Hz
GB 0
PC 1.40
194.07 160.29 140.90 134.02 133.84 131.39 130.39 129.25 129.01 128.85 127.99 127.60 127.37 122.19 108.55 90.57 50.80
Cl
Ph
COPh
O
5t
200 180 160 140 120 100 80 60 40 20 ppm

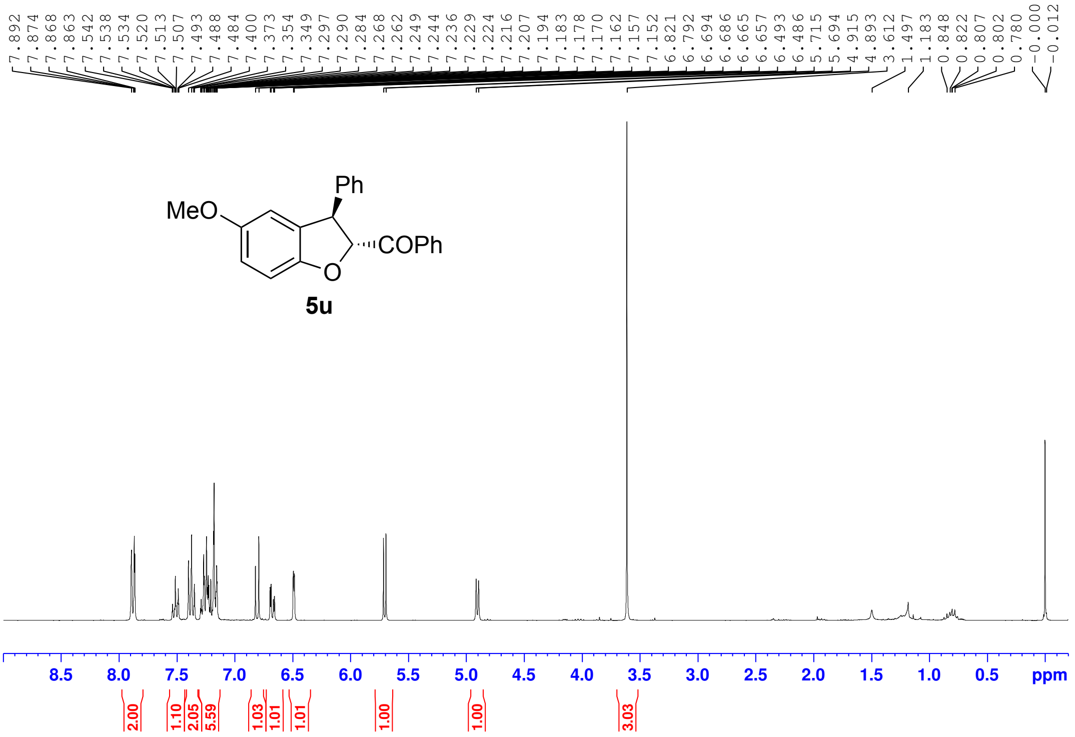

7.892
7.874
7.868
7.863
7.542
7.538
7.534
7.520
7.513
7.507
7.493
7.488
7.484
7.400
7.373
7.354
7.349
7.297
7.290
7.284
7.268
7.262
7.249
7.244
7.236
7.229
7.224
7.216
7.207
7.194
7.183
7.178
7.170
7.162
7.157
7.152
6.821
6.792
6.694
6.686
6.665
6.657
6.493
6.486
5.715
5.694
4.915
4.893
3.612
1.497
1.183
0.848
0.822
0.807
0.802
0.780
-0.000
-0.012
MeO
Ph
COPh
O
5u
8.5 8.0 7.5 7.0 6.5 6.0 5.5 5.0 4.5 4.0 3.5 3.0 2.5 2.0 1.5 1.0 0.5 ppm
2.00
1.10
2.05
5.59
1.03
1.01
1.01
1.00
1.00
3.03

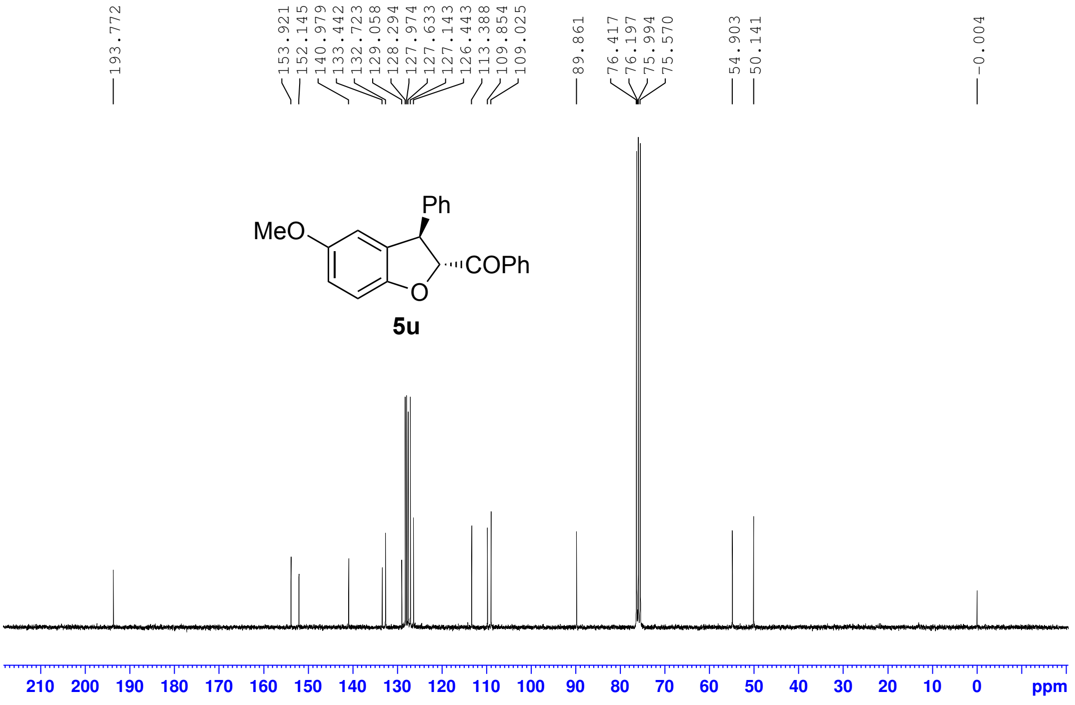

193.772
153.921
152.145
140.979
133.442
132.723
129.058
128.294
127.974
127.633
127.143
126.443
113.388
109.854
109.025
89.861
76.417
76.197
75.994
75.570
54.903
50.141
-0.004
MeO
Ph
COPh
O
5u
210 200 190 180 170 160 150 140 130 120 110 100 90 80 70 60 50 40 30 20 10 0 ppm

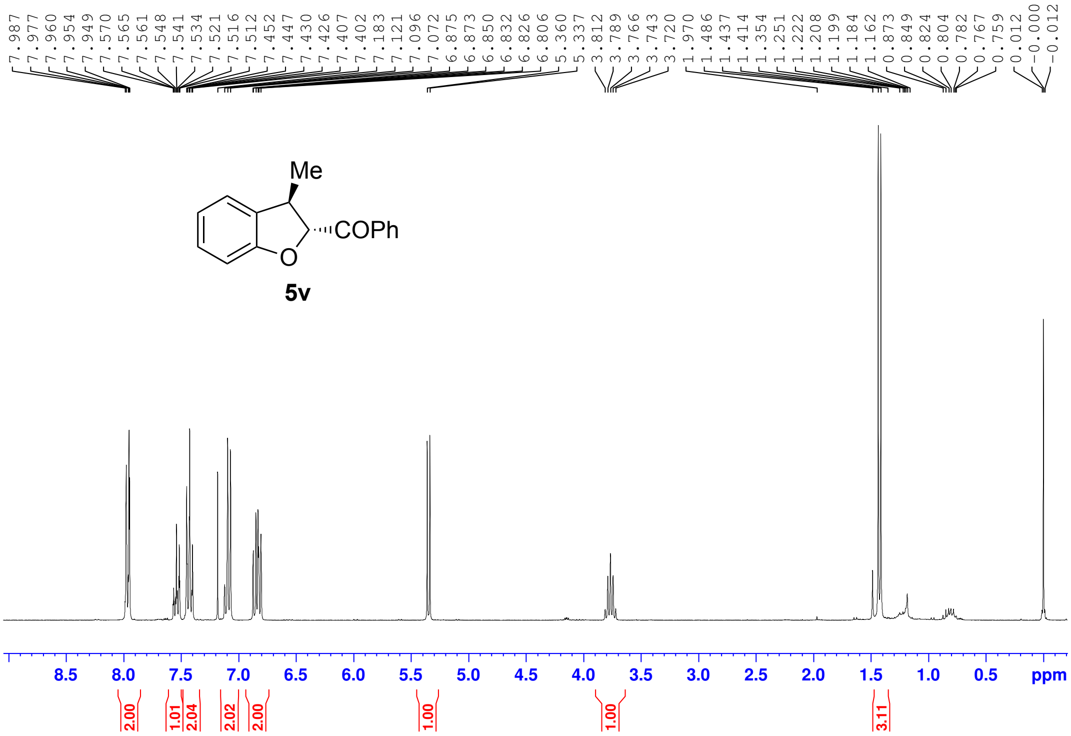
Me
COPh
O
5v
8.5
8.0
7.5
7.0
6.5
6.0
5.5
5.0
4.5
4.0
3.5
3.0
2.5
2.0
1.5
1.0
0.5
ppm
2.00
1.01
2.04
2.02
2.00
1.00
1.00
3.11

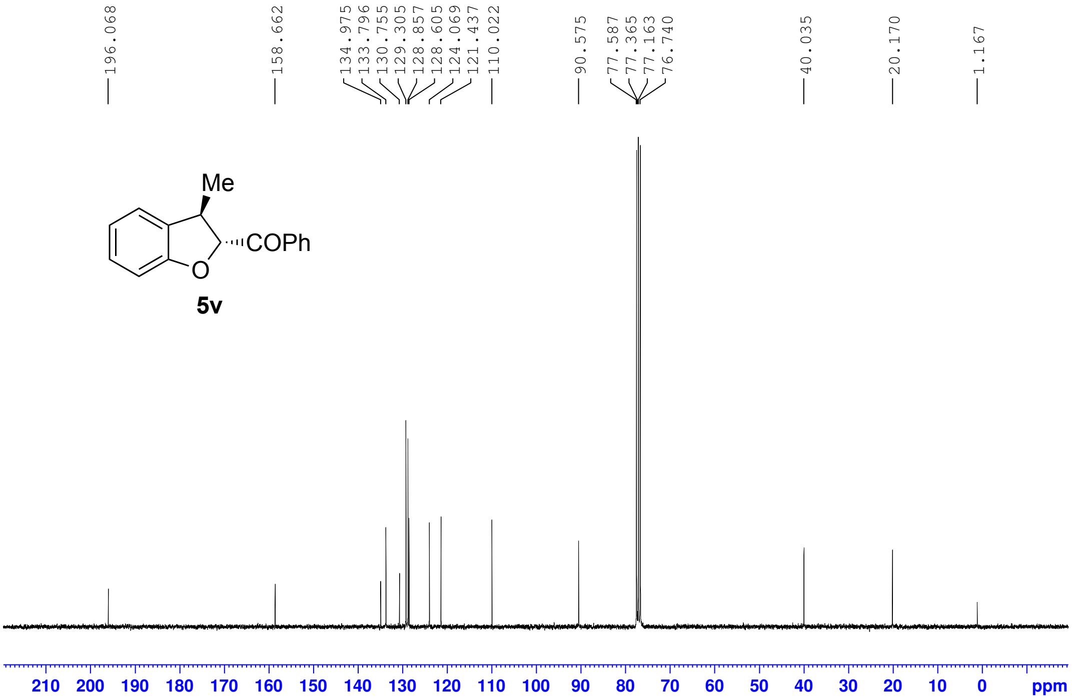
196.068
158.662
134.975
133.796
130.755
129.305
128.857
128.605
124.069
121.437
110.022
90.575
77.587
77.365
77.163
76.740
40.035
20.170
1.167
Me
COPh
O
5v
210
200
190
180
170
160
150
140
130
120
110
100
90
80
70
60
50
40
30
20
10
0
ppm
